# Taxonomic revision of the *Pheidole sikorae* species group (Hymenoptera, Formicidae) from Madagascar

**DOI:** 10.3897/zookeys.949.51269

**Published:** 2020-07-15

**Authors:** Sebastian Salata, Brian L. Fisher

**Affiliations:** 1 Department of Entomology, California Academy of Sciences, San Francisco, CA 94118, USA Department of Entomology, California Academy of Sciences San Francisco United States of America

**Keywords:** endemic species, Malagasy region, Myrmicinae, taxonomy

## Abstract

The present study represents a taxonomic revision of the *Pheidole sikorae* species group from Madagascar. Forty-four members of this group are recognised and described, and an illustrated identification key to this group is also presented. One species is raised to species level: *P. litigiosa* Forel, 1892 **stat. nov.***Pheidole veteratrix angustinoda* Forel, 1892 **syn. nov.** is proposed as a junior synonym of *Pheidole veteratrix* Forel, 1891. Worker castes are also described and lectotypes designated for *P. litigiosa* Forel, 1892, *P. sikorae* Forel, 1891, and *P. veteratrix* Forel, 1891. The following 41 new species are described: *P. alina***sp. nov.**, *P. ambohimanga***sp. nov.**, *P. analavelona***sp. nov.**, *P. andohahela***sp. nov.**, *P. anomala***sp. nov.**, *P. anosyenne***sp. nov.**, *P. antranohofa***sp. nov.**, *P. beanka***sp. nov.**, *P. befotaka***sp. nov.**, *P. dasos***sp. nov.**, *P. flavominuta***sp. nov.**, *P. gracilis***sp. nov.**, *P. haboka***sp. nov.**, *P. havoana***sp. nov.**, *P. hazo***sp. nov.**, *P. itremo***sp. nov.**, *P. joffreville***sp. nov.**, *P. kely***sp. nov.**, *P. lavasoa***sp. nov.**, *P. mahamavo***sp. nov.**, *P. mainty***sp. nov.**, *P. mamiratra***sp. nov.**, *P. manantenina***sp. nov.**, *P. masoandro***sp. nov.**, *P. mavohavoana***sp. nov.**, *P. midongy***sp. nov.**, *P. mikros***sp. nov.**, *P. mivory***sp. nov.**, *P. nitidobruna***sp. nov.**, *P. parvula***sp. nov.**, *P. parvulogibba***sp. nov.**, *P. renirano***sp. nov.**, *P. sava***sp. nov.**, *P. sofia***sp. nov.**, *P. sparsa***sp. nov.**, *P. tampony***sp. nov.**, *P. trichotos***sp. nov.**, *P. tsaravoniana***sp. nov.**, *P. vadum***sp. nov.**, *P. volontany***sp. nov.**, and *P. vony***sp. nov.** At present, there are 109 valid species and subspecies of *Pheidole* known from Madagascar, but this number is expected to increase with upcoming taxonomic revisions of the species groups not revised in this study.

## Introduction

The hyper-diverse ant genus *Pheidole* Westwood, 1839, with 1,095 species and 132 subspecies, is the second most species-rich ant genus in the world (Bolton 2020). Its centre of distribution is located in the Neotropical region, which hosts nearly 50% of known *Pheidole* taxa. The other zoogeographical regions hold distinctly fewer number of species: Oceania – 20, Palearctic – 22, Nearctic – 79, Australasia – 110, Afrotropic – 139, and Indomalaya – 146 (Bolton 2020). However, knowledge of the taxonomy and diversity of *Pheidole* is far from complete and a significant increase in the number of taxa can be expected in a poorly studied region such as the Afrotropic (Fischer et al. 2012).

The Malagasy *Pheidole* had been neglected for decades until recent papers published by Fischer and Fisher (2013) and Salata and Fisher (2020). Fischer and Fisher (2013) provided the first comprehensive review of *Pheidole* known from the small Malagasy Region islands. They summarised hitherto known data on their diversity and described the presence of 13 species, seven of which were considered as endemic. However, their work did not cover Madagascar, the largest Malagasy island, recognised by Fisher and Peeters (2019) as having exceptionally diverse and abundant *Pheidole* fauna.

Taxonomic research published by Salata and Fisher (2020) ended a 100 year-long break in studies on this genus from Madagascar. They proposed the first species-group division, revised eleven species groups, redescribed six species, and described 46 species new to science. Their study increased the number of known *Pheidole* from 20 to 69, of which 67 are considered endemic. However, they estimated that the number of species will increase with taxonomic work on the following five species groups: *sikorae*, *bessonii*, *fervens*, *megacephala*, and *lucida*.

In this study, we present a revision of the *sikorae* species group, which appears to be the most species-rich on Madagascar. As mentioned in Salata and Fisher 2020, our species-group division is based on morphological similarities and their monophyly will be addressed in further studies. The *sikorae* group contains 44 species and its members are known exclusively from the island. Three species, *P. litigiosa* Forel, *P. sikorae* Forel, and *P. veteratrix* Forel, are already described members of the group. An additional 41 taxa are described here as new to science. This work increases the total number of species of Madagascar *Pheidole* to 109 (106 species endemic to the island) and ranks this genus as the second most species-rich on the island. The number of valid *Pheidole* taxa from the Malagasy region is estimated at 119, making this bioregion the fourth most species-rich globally.

## Materials and methods

The majority of the material was collected by BLF and members of the Madagascar Biodiversity Centre from across Madagascar between 1991 and 2018. We included also material deposited in the Museum d’Historie Naturelle, Geneva, Switzerland.

**Figure 1.**
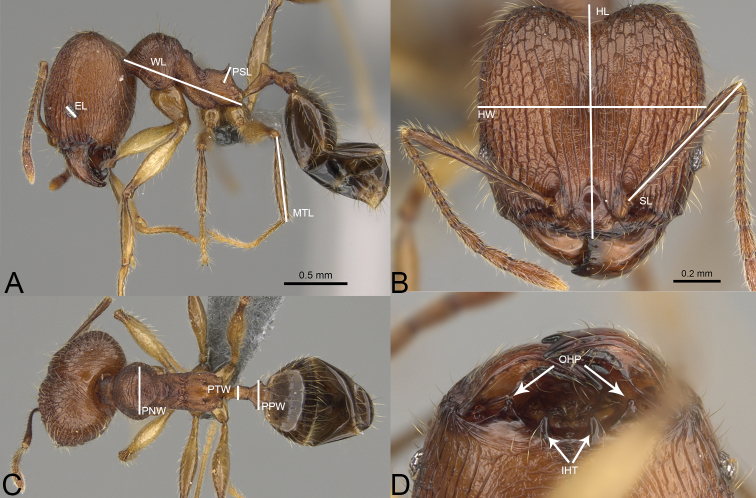
*Pheidole tsaravoniana* sp. nov., illustrations of measurements (**A–C**). **A** Profile **B** full-face view **C** dorsal view **D** inner hypostomal teeth (IHT) and outer hypostomal teeth (OHT).

Repositories. Collections are referred to by the following acronyms:

**CASC**California Academy of Sciences, San Francisco, California, USA;

**MHNG**Muséum d’Historie Naturelle, Geneva, Switzerland;

**PBZT**Parc Botanique et Zoologique de Tsimbazaza, Antananarivo, Madagascar.

All observations and measurements were taken using a pin-holding stage, permitting rotations around the X, Y, and Z axes at magnifications from 32× to 100× with a Leica MZ12.5 microscope and an orthogonal crosshair micrometre, at an accuracy of 0.01 mm to approximately 0.005 mm. All measurements are presented in mm units as minimum and maximum values, with the arithmetic mean in parentheses. Photographs were taken using a JVC KY-75 or Leica DFC450 digital camera with a Leica Z16 APO microscope and Leica Application Suite software (v3.8). Unless stated otherwise, photographs were taken by Michele Esposito. Images of specimens and data of all pinned specimens examined in the present contribution are available online on AntWeb (www.AntWeb.org) and accessible using the unique CASENT identifying specimen code. Measurements and indices are in line with Salata and Fisher (2020) and are predominantly the same as in Longino (2009, 2019) and several other revisions (Eguchi 2008; Fischer et al. 2012; Fischer and Fisher 2013; Wang et al. 2018). The general morphological terminology follows Wilson (2003) and Longino (2009, 2019). Additionally, in both majors and queens of *Pheidole sikorae*, we observed an impressed and smooth concavity present lateral to the antennal socket and the tentorial pit (Figs 14E, 53B, D). The concavity is separated from the antennal socket by a distinct ridge. We cannot consider these structures as reduced antennal scrobes because: a) they do not accommodate the basal part of the scape, which is always distinctly convex, and b) they extend anteriorly towards the posterior margin of clypeus. To our knowledge, this morphological feature is found exclusively in majors and queens of *P. sikorae*.

As older taxa are often insufficiently characterised by their original describers, diagnoses are provided in the redescriptions for *P. litigiosa* Forel, 1892, *P. sikorae* Forel, 1891, and *P. veteratrix* Forel, 1891 to make identifications easier.

Our recognition of species follows the biological species concept and species boundaries are based on comparative morphology and known geographic distributions of investigated taxa. Where sympatric populations exhibit consistently different phenotypes, they are considered different species. Species described based on the single nest sample exhibit distinct and unique set of morphological features allowing their separation from other Madagascan *Pheidole* species.

Pilosity inclination degree follows that used in Wilson (1955). Appressed (0–5°) hairs run parallel or nearly parallel to the body surface. Decumbent hairs stand 10–40°, subdecumbent hair stand ~ 45° from the surface, suberect hairs bend about 10°–20° from vertical, and erect hairs stand vertical or nearly vertical.

Maps were generated using tmap v2.2 package on R v3.5. R Core Team (2018).

### Measurements and indices

**Measurements**

**EL** eye length; measured along the maximum vertical diameter of the eye;

**HL** maximum distance from the midpoint of the anterior clypeal margin to the midpoint of the posterior margin of the head, measured in full-face view; in majors from midpoint of tangent between anteriormost position of clypeus to midpoint of tangent between posteriormost projection of the vertex;

**HW** head width; measured in full-face view, at widest point of the head, directly above the eyes;

**MTL** metatibia length; straight line length of the metatibia measured from the constriction immediately before its proximal insertion to its distalmost point, excluding the bristles or spines;

**PNW** pronotum width; maximum width of promesonotum measured in dorsal view;

**PPW** postpetiole width; maximum width of postpetiole in dorsal view;

**PSL** propodeal spine length; measured from the centre of the propodeal spiracle to the tip of the propodeal spine in lateral view;

**PTW** petiole width; maximum width of petiole in dorsal view;

**SL** scape length; maximum straight-line length of scape excluding the basal condylar bulb;

**WL** mesosoma length (Weber’s length); diagonal length of mesosoma in lateral view from the anterior point of the pronotal slope and excluding the neck, to the posteroventral margin of the propodeum.

**Indices**

**CI** cephalic index: HW / HL * 100;

**MTI** tibia index: MTL / HW * 100;

**SI** scape index: SL / HW * 100;

**PNI** pronotum index: PNW / HW * 100;

**PPI** postpetiole width index: PPW / PTW * 100;

**PSLI** propodeal spine index: PSL / HW * 100.

### Abbreviations

**m**.–male; **q**.–gyne; **s**–major worker; **w**.–minor worker.

## Synopsis of species of the *Pheidole sikorae* group

*Pheidole alina* **sp. nov.**

*Pheidole ambohimanga* **sp. nov.**

*Pheidole analavelona* **sp. nov.**

*Pheidole andohahela* **sp. nov.**

*Pheidole anomala* **sp. nov.**

*Pheidole anosyenne* **sp. nov.**

*Pheidole antranohofa* **sp. nov.**

*Pheidole beanka* **sp. nov.**

*Pheidole befotaka* **sp. nov.**

*Pheidole dasos* **sp. nov.**

*Pheidole flavominuta* **sp. nov.**

*Pheidole gracilis* **sp. nov.**

*Pheidole haboka* **sp. nov.**

*Pheidole havoana* **sp. nov.**

*Pheidole hazo* **sp. nov.**

*Pheidole itremo* **sp. nov.**

*Pheidole joffreville* **sp. nov.**

*Pheidole kely* **sp. nov.**

*Pheidole lavasoa* **sp. nov.**

*Pheidole litigiosa* Forel **stat. nov.**

*Pheidole mahamavo* **sp. nov.**

*Pheidole mainty* **sp. nov.**

*Pheidole mamiratra* **sp. nov.**

*Pheidole manantenina* **sp. nov.**

*Pheidole masoandro* **sp. nov.**

*Pheidole mavohavoana* **sp. nov.**

*Pheidole midongy* **sp. nov.**

*Pheidole mikros* **sp. nov.**

*Pheidole mivory* **sp. nov.**

*Pheidole nitidobruna* **sp. nov.**

*Pheidole parvula* **sp. nov.**

*Pheidole parvulogibba* **sp. nov.**

*Pheidole renirano* **sp. nov.**

*Pheidole sava* **sp. nov.**

*Pheidole sikorae* Forel

*Pheidole sofia* **sp. nov.**

*Pheidole sparsa* **sp. nov.**

*Pheidole tampony* **sp. nov.**

*Pheidole trichotos* **sp. nov.**

*Pheidole tsaravoniana* **sp. nov.**

*Pheidole vadum* **sp. nov.**

*Pheidole veteratrix* Forel

=*Pheidole veteratrix angustinoda* Forel **syn. nov.**

*Pheidole volontany* **sp. nov.**

*Pheidole vony* **sp. nov.**

## Taxonomy

### Species accounts

Repetitive characters occurring in the majority of species have been omitted. Unless stated otherwise, the following descriptions apply to all species treated here.

**Major workers.** Dorsal face of head in lateral view not depressed posteriorly; antennal sockets shallow; frontal lobes absent; head in full-face view with distinct median concavity; antenna 12-segmented, with 3-segmented club; masticatory margin of mandible with large, stout apical and preapical teeth, followed by a long diastema and then a short and crenulate tooth just before the rounded basal angle; outer surface of mandible mostly smooth and shining, sometimes with weak and sparse foveolae; antennal scrobes absent; promesonotum strongly convex, well above the level of propodeum; petiolar peduncle with small horizontal lobes on its basal part; postpetiole short with slightly convex dorsum; petiolar peduncle without horizontal lobes on its basal part; body unicolourous.

**Minor workers.** Antennal sockets shallow; frontal lobes absent; occipital carina absent; head in full-face view oval, posterior and anterior of eyes convex; antenna 12-segmented, with 3-segmented club; humeral area not developed; clypeus smooth and shiny, its anterior margin regularly convex; promesonotum well above the level of propodeum; petiole smooth, with node moderately low, triangular, and small, with few short, erect setae; petiolar peduncle with ventral face slightly convex; postpetiole smooth, short, low, and slightly convex, with few short, erect setae; gaster smooth and shiny; body unicolourous.

### Revision of the *Pheidole sikorae* group

**Diagnosis. Major workers.** Postpetiole in profile without conspicuous ventral convexity; antennal sockets shallow; frontal lobes absent or indistinct; propodeal spines small to moderate, never very long and narrow; head in full-face view sub-oval with lateral sides from relatively straight to convex (sometimes when sides of head are relatively straight head appears sub-rectangular), often head slightly widening posteriorly; head in lateral view sub-oval with distinctly convex sides; occipital lobes never with transverse rugae and in most cases entirely sculptured (except *P. tampony* sp. nov., *P. gracilis* sp. nov., *P. litigiosa*, and *P. masoandro* sp. nov.); frons entirely sculptured, medially with predominantly longitudinal to irregular rugae, lateral sides with rugae more irregular or longitudinally irregular; antennal scrobes absent; rugae on head always thick and distinct; promesonotum short, angular, and moderately high; postpetiole in dorsal view with lateral margins medially with two dentate, small to moderate projections; gaster most often smooth (except *P. mikros* sp. nov., *P. beanka* sp. nov., *P. trichotos* sp. nov., *P. renirano* sp. nov., *P. tsaravoniana* sp. nov., *P. mivory* sp. nov.).

**Minor workers.** Postpetiole in profile without conspicuous ventral convexity; antennal sockets shallow; frontal lobes absent or indistinct; propodeal spines minute to moderate, never very long and narrow; promesonotum in lateral view never box-like and posterior mesonotum never steep; promesonotal groove absent or distinct but never very deep; posterior region of the head never elongated into short to long neck.

**Comments.** Members of the *sikorae* group are predominantly distributed across the central highlands, evergreen rainforest, and Sambirano rainforest. Very few records of these species are from arid and dry biomes. Also, Antsiranana prefecture emerges as a region characterised by the highest diversity of the group. Species of the *sikorae* group, especially major workers, are most similar to some representatives of the *P. lutea* group (*P. ranohirensis* Salata & Fisher, *P. lutea* Salata & Fisher, and *P. voasara* Salata & Fisher) and members of the *P. ferruginea* group. Majors of the *sikorae* group can be easily distinguished from those of the *P. ferruginea* group based on absence of antennal scrobes, lower promesonotum in lateral view, shorter and thicker propodeal spines and smooth to indistinctly shagreened first gastral tergite; from members of the *P. lutea* group they differ in lack of antennal scrobes, longer scape which exceeds midlength of the head, and thicker head sculpture, especially on its posterior part. Minor workers can be more confusing and species determinations should be always made based on nest samples consisting of major and minor workers. Overall, minors of the *sikorae* group can be distinguished from others based on the combination of the following characters: body yellow to black; promesonotum, in lateral view, from short and arched to long and low but never box-like and never with steep posterior mesonotum; propodeal spines most often minute to small, never long and sharp; head in full-face view sub-rectangular to sub-oval but never elongated.

### Key to species of the *Pheidole sikorae* group

**Table:** 

1	Minute species. Major workers with HL < 1.1 mm and WL < 0.9 mm and minor workers with HL < 0.5 mm and WL < 0.6 mm. All four measurements must fit those requirements; if at least one is higher than stated limits then go to couplet 13	**2**
–	Moderately large species. Major workers with HL > 1.1 mm and WL > 0.9 mm and minor workers with HL > 0.55 mm and WL > 0.7 mm	**13**
2	Major workers. Head in full–face view sub–oval, body dark brown, and sides of head with very dense, relatively short to short, suberect pilosity (Fig. 2A, F). Minor workers. Scape, when laid back, surpassing the posterior head margin by two-fifths of its length, SI > 130.0; propodeal spines reduced to small lobes, head elongate and oval and body yellowish brown to dark brown (Fig. 4A, F)	**3**
–	Major workers. Head, in full-face view elongate, if sub-oval then body orange to yellow or body dark brown with sides of the head with dense, very long, suberect to erect pilosity (Fig. 2 B–E, G–L). Minor workers. Scape, when laid back, reaching the posterior head margin or surpassing it by one-fifth of its length, SI < 115.0; propodeal spines minute to moderately large but always developed; if propodeal spines reduced to small lobes then head more rectangular in shape (Fig. 4 B–E, G–L)	**4**
3	Major workers. Sides of the head with very dense, short, suberect to erect pilosity, inner hypostomal teeth triangular with rounded apex directed upward, outer hypostomal teeth with dentate sharp apex directed slightly outward (Fig. 2F, N). Minor workers. Head sculpture shiny and smooth but frons with sparse, short, and longitudinal rugulae, mesosoma shiny and smooth, body yellowish brown (Figs 4F, 5F)	***Pheidole lavasoa* sp. nov.**
–	Major workers. Sides of the head with sparser and longer, suberect pilosity, inner hypostomal teeth with sharp apex directed upward, outer hypostomal teeth lobe-like with apex directed distinctly outward (Fig. 2A, M). Minor workers. Head sculpture shiny and smooth but vertex and frons with sparse, short, and transverse rugulae, anterolateral sides of propodeum and anepisternum with indistinct and sparse rugoreticulae, body dark brown (Figs 4A, 5A)	***Pheidole andohahela* sp. nov.**
4	Major workers. Head in full-face view elongate, not widening posteriorly, with anterior and posterior sides slightly convex (Fig. 2B, C, H, I). Minor workers. Head predominantly smooth and in full–face view rectangular with anterolateral sides relatively straight or indistinctly convex, body yellow to brown (Figs 4B, C, H, I, 5B, C, H, I)	**5**
–	Major workers. Head in full-face view sub-oval, slightly widening posteriorly, with anterior and posterior sides convex (Fig. 2D, E, G, J, K, L). Minor workers. Head foveolate; if predominantly smooth, then in full-face view oval with anterolateral sides distinctly convex or body dark brown (Figs 4D, E, G, J, K, L, 5D, E, G, J, K, L)	**8**
5	Major worker. Body yellow, head in lateral view sub-oval and elongate with slightly convex dorsal and ventral sides, base of first gastral tergite smooth (Figs 2C, 3C). Minor worker. Scape, when laid back, reaching the posterior head margin, SI < 96.0, body yellow (Figs 4C, 5C)	***Pheidole flavominuta* sp. nov.**
–	Major worker. Body brown to orange, head in lateral view sub-oval and short with strongly convex dorsal and ventral sides, base of first gastral tergite shagreened (Figs 2B, H, I, 3B, H, I). Minor workers. Scape, when laid back, exceeding the posterior head margin by one-fifth of its length, body yellowish brown or bright yellow, SI > 98.0 (Figs 4B, H, I, 5B, H, I)	**6**
6	Major workers. Medial part of frons with thick, longitudinal, and moderately dense rugae and smooth to indistinctly rugulate interspaces, occipital lobes with dense, irregular rugae, and indistinctly rugoreticulate interspaces, lateral sides of promesonotum with smooth notches (Figs 2I, 3I). Minor workers. Vertex and frons smooth (Fig. 4I)	***Pheidole nitidobruna* sp. nov.**
–	Major workers. Medial part of frons with thick, longitudinal, and dense rugae and distinctly to indistinctly rugulate interspaces, occipital lobes with irregular rugae and distinctly foveolate or rugoreticulae interspaces, lateral sides of promesonotum entirely sculptured (Figs 2B, H, 3B, H). Minor workers. Vertex and/or frons with additional sculpture (Fig. 4B, H)	**7**
7	Major workers. Medial part of frons with thick, longitudinal, and dense rugae and indistinctly rugulate interspaces, propodeal spines moderately long (Figs 2H, 3H). Minor workers. Lateral sides of frons with fine, sparse, and irregular rugulae with smooth interspaces, mesosoma with sparse and moderately thick network of irregular rugulae, mesonotum and katepisternum smooth, body yellowish brown, propodeal spines small, triangular (Figs 4H, 5H)	***Pheidole mikros* sp. nov.**
–	Major workers. Medial part of frons with thick, longitudinal, and dense rugae and distinctly rugulate interspaces, propodeal spines very small (Figs 2B, 3B). Minor workers. Lateral sides of frons smooth, mesosoma smooth, propodeal spines reduced to small tubercles, body yellow (Figs 4B, 5B)	***Pheidole beanka* sp. nov.**
8	Major workers. Body dark orange, medial part of frons with longitudinal rugae, rugae in posteromedial part more irregular, interspaces shiny with dense and distinct irregular rugulae, pronotal dorsum and lateral sides of propodeum with reduced sculpture (Figs 2D, 3D). Minor workers. Head foveolate, frons with additional indistinct longitudinal and interrupted rugae in medial part, area posterolateral from eyes smooth (Fig. 4D)	***Pheidole havoana* sp. nov.**
–	Major workers. Body dark brown; if body orange then medial part of frons with longitudinal and dense rugae and smooth to indistinctly rugulate interspaces or pronotum distinctly foveolate with additional irregular rugae on dorsum and propodeum with fine rugulae and smooth interspaces (Figs 2E, G, J, K, L, 3E, G, J, K, L). Minor workers. Frons and vertex at least partly smooth (Fig. 4E, G, J, K, L)	**9** ^*^
9	Major and minor workers. Bright body colouration, orange to yellow (Figs 2E, J, K, 3E, J, K, 4E, J, K, 5E, J, K)	**10**
–	At least major workers brown, minor workers brown to dark yellow (Figs 2G, L, 3G, L, 4G, L, 5G, L)	**12**
10	Major workers. Medial part of frons with thick, dense, longitudinal and interrupted rugae and smooth to indistinctly rugulate interspaces, mesosoma with fine and sparse rugofoveolae, pronotum with rugofoveolae reduced and smooth notches on medial parts of dorsum and its lateral sides (Figs 2E, 3E). Minor workers. Frons and vertex distinctly foveolate (Fig. 4E)	***Pheidole kely* sp. nov.**
–	Major workers. Medial part of frons with thick and dense rugae and distinctly rugulate interspaces; if interspaces smooth or indistinctly regulate, then promesonotum foveolate with sparse and thick to moderately thick transverse to irregular rugae on dorsum, smooth notches absent (Figs 2J, K, 3J, K). Minor workers. Frons and vertex never distinctly foveolae, predominantly smooth (Fig. 4J, K)	**11**
11	Major workers. Medial part of frons with thick and dense rugae and distinctly rugulate interspaces (Fig. 2J). Minor workers. Medial frons smooth, lateral sides of frons and vertex with fine, sparse, and irregular rugulae with smooth interspaces (Fig. 4J)	***Pheidole parvula* sp. nov.**
–	Major workers. Medial part of frons with thick, dense rugae and smooth to indistinctly rugulate interspaces (Fig. 2K). Minor workers. Frons and vertex smooth (Fig. 4K)	***Pheidole parvulogibba* sp. nov.**
12	Major workers. Medial part of frons with moderately dense, thick, longitudinal and interrupted rugae, interspaces shiny and smooth, sides of the head with dense, very long, suberect to erect pilosity (Fig. 2L). Minor workers. Body dark brown and head predominantly smooth (Figs 4L, 5L)	***Pheidole volontany* sp. nov.**
–	Major workers. Medial part of frons with thick, longitudinal and dense rugae and distinctly rugulate interspaces, sides of the head with moderately dense, moderately short, suberect pilosity (Fig. 2G). Minor workers. Frons with sparse and moderately thick, short and predominantly longitudinal rugulae, vertex with transverse, sparse, and moderately thick, short rugulae (Figs 4G, 5G)	***Pheidole midongy* sp. nov.**
13	Major workers. Posterior part of head with distinctly reduced sculpture, occipital lobes entirely or predominantly smooth, area posterolateral from eyes entirely smooth and shiny or with reduced sculpture and smooth notches (Fig. 6). Minor workers. Body orange to yellow, head at least medially foveolate, promesonotal groove present but sometimes indistinct, mesosoma predominantly or entirely smooth and promesonotum short or mesosoma predominantly foveolate and promesonotum low and long (Fig. 7)	**14**
–	Major workers. Entire head distinctly sculptured, sometimes sculpture weakening posteriorly but always visible, smooth notches, if present, occur only on the posteriormost part of lateral sides of head (Figs 8, 14, 16, 17). Minor workers. Character combination different (Figs 9, 15, 18)	**17** ^*^
14	Major workers. Body brown, occipital lobes predominantly smooth, area posterolateral from eyes predominantly with dense and thick longitudinal rugae with rugulate interspaces and reduced sculpture and predominantly smooth posteriormost parts (Fig. 6D, H). Minor workers. Promesonotum low and moderately long, mesosoma foveolate, area posterolateral from eyes smooth, body bright brown (Fig. 7D, H)	***Pheidole tampony* sp. nov.**
–	Major workers. Body yellow to yellowish orange, at least occipital lobes and posteriormost parts of area posterolateral from eyes smooth or occipital lobes predominantly smooth, area posterolateral from eyes with indistinct, dense, thin, and longitudinal rugae and smooth on the posteriormost parts (Fig. 6A–C, D–G). Minor workers. Promesonotum moderately low and short and mesosoma predominantly smooth; if promesonotum low and moderately long and mesosoma foveolate then body yellow and area posterolateral from eyes always with indistinct sculpture (Fig. 7A–C, D–G)	**15**
15	Major workers. Medial part of frons with moderately thick, longitudinal, interrupted, and moderately dense rugae, occipital lobes predominantly smooth, only anterior part with indistinct, dense, thin, and longitudinal rugae, promesonotum distinctly foveolate (Fig. 6A, E). Minor workers. Promesonotum low and moderately long, mesosoma foveolate (Fig. 7A, E)	***Pheidole gracilis* sp. nov.**
–	Major workers. Medial part of frons with thick, longitudinal, interrupted, and moderately dense rugae, occipital lobes smooth, promesonotum predominantly smooth (Fig. 6B, C, F, G). Minor workers. Promesonotum moderately low and short, mesosoma predominantly smooth (Fig. 7B, C, F, G)	**16**
16	Major workers. Medial part of frons with thick, longitudinal, interrupted, and moderately dense rugae, interspaces rugulate, sides of the head with moderately dense, short, subdecumbent to suberect pilosity (Fig. 6B, F). Minor workers. Head with foveolate frons and no additional rugae, promesonotal groove indistinct, propodeal spines small, triangular (Fig. 7B, F)	***Pheidole litigiosa* Forel**
–	Major workers. Medial part of frons with moderately dense, thick, interrupted longitudinal rugae, interspaces smooth, sides of the head with very dense, long, erect pilosity (Fig. 6C, G). Minor workers. Head with foveolate frons with additional rugae, promesonotal groove distinct, propodeal spines minute (Fig. 7C, G)	***Pheidole masoandro* sp. nov.**
17	Major workers. Body black or brownish black (Fig. 8). Minor workers. Head and mesosoma black or head and mesosoma brown to dark brown with dense foveolae and no smooth notches (Fig. 9)	**18**
–	Major workers. Body yellow to dark brown (Figs 14, 16, 17). Minor workers. Body yellow to orange; if head and mesosoma dark brown then sculpture reduced and never entirely foveolae (Figs 15, 18)	**20**
18	Major workers. Body brownish black. Medial part of frons with thick, interrupted, dense, and longitudinally irregular rugae with indistinctly to distinctly rugulate interspaces, base of first gastral tergite shagreened (Fig. 8C). Minor workers. Body brown to brownish black, head and mesosoma entirely foveolae, smooth notches absent, scape, when laid back, exceeding the posterior head margin by two-fifths of its length, SI < 153.0 (Fig. 9C, F)	***Pheidole trichotos* sp. nov.**
–	Major workers. Medial part of frons with thick, interrupted, dense, and longitudinal rugae with smooth to indistinctly rugulate interspaces, base of first gastral tergite smooth (Fig. 8A, B, F, G). Minor workers. Body black to brownish black, head and mesosoma foveolae with smooth notches, if smooth notches absent then scape, when laid back, exceeding the posterior head margin by one-third of its length SI > 163.0 (Fig. 9A, B, D, E)	**19**
19	Major workers. Sides of the head with moderately dense, long, suberect to erect pilosity, inner hypostomal teeth indistinct, low, and wide, closely spaced, bulge-like, propodeal spines moderately long, narrow and with acute apex (Fig. 8A, D, F). Minor workers. Head foveolate, median frons with short and indistinct longitudinal rugulae, area posterolateral from eyes with slightly to distinctly weaker sculpture, mesosoma foveolate, katepisternum with reduced sculpture or with smooth notch (Fig. 9A, D)	***Pheidole alina* sp. nov.**
–	Major workers. Sides of the head with very dense, moderately long, suberect to erect pilosity, inner hypostomal teeth distinct, moderately large and narrow, closely spaced, triangular with apex directed slightly inward, propodeal spines moderately long, wide, and with acute apex (Fig. 8B, E, G). Minor workers. Head foveolate, vertex with fading foveolae, frons with distinct and sparse, longitudinal rugae, area posterolateral from eyes smooth, mesosoma foveolate, promesonotal dorsum, katepisternum, and propodeum predominantly smooth (Fig. 9B, E)	***Pheidole mainty* sp. nov.**
20	Major workers. Medial frons predominantly with dense, irregular, and thick rugae, sometimes longitudinal to longitudinally irregular rugae occur on anterior part of frons but posterior part is always distinctly irregular (Figs 10A–C, 11A–C, 12A–C, 13A–C). Minor workers. Promesonotum low and long, dorsal and lateral sides of pronotum distinctly rugulate with no smooth notches, if smooth notches present then propodeum with indistinct to distinct foveolae or head always with foveolae, at least on frons, promesonotum short and moderately high, surpassing the posterior head margin by one-fifth of its length and mesosoma predominantly foveolate or head with additional thick longitudinal rugae on frons or promesonotal groove absent or indistinct (Figs 10D–I, 11D–F, 12D–F, 13G–I)	**21**
–	Major workers. Medial frons predominantly with sparser, longitudinal, and sometimes interrupted, rugae, interspaces sometimes with irregular but distinctly thinner rugae (Figs 14, 16). Minor workers. Promesonotum low and long, dorsal and lateral sides of pronotum with reduced sculpture and smooth notches and propodeum with distinct foveolae or predominantly smooth or head predominantly smooth, if foveolae occur and promesonotum short and moderately high then promesonotum predominantly smooth or frons without additional, thick rugae and promesonotal groove very distinct (Figs 15, 18)	**32** ^*^
21	Major workers. Head in full–face view oval, not widening posteriorly, with strongly convex sides, medial part of frons with thick, moderately sparse, irregular rugae or with moderately dense, thin, longitudinal anteriorly to irregular posteriorly interrupted rugae and very shallow occipital cleft (Fig. 10A–C). Minor workers. Body orange to brown, never yellow, promesonotum short and moderately high, head with additional moderately thick to thick longitudinal and interrupted rugae or head foveolate with no additional sculpture (Fig. 10D–I)	**22**
–	Major workers. Head in full-face view sub–oval, most often slightly to distinctly widening posteriorly with convex sides, medial part of frons with thick, dense, irregular rugae, sometimes anterior part with rugae more longitudinal (Figs 11A–C, 12A–C, 13A–C). Minor workers. Body yellow, if orange to brown then promesonotum low and moderately long or promesonotum short and moderately high, and head predominantly foveolate with rugae very thin and indistinct (Figs 11D–F, 12D–F, 13G–I)	**24**
22	Major workers. Medial part of frons with moderately dense, thin, longitudinal anteriorly to irregular posteriorly interrupted rugae and very shallow occipital cleft (Fig. 10C). Minor workers. Head predominantly foveolate with no additional sculpture (Fig. 10F)	***Pheidole vadum* sp. nov.**
–	Major workers. Medial part of frons with thick, moderately sparse, irregular rugae, occipital cleft deeper (Fig. 10A, B). Minor workers. Head with additional moderately thick to thick longitudinal and interrupted rugae (Fig. 10D, E)	**23**
23	Major workers. Frons with thick, irregular rugae, interspaces shiny with distinct and sparse rugoreticulae (Fig. 10B). Minor workers. Head sculpture foveolate, medial frons with foveolae sparser, area posterolateral from eyes with reduced sculpture and smooth notches, mesonotum, anepisternum, katepisternum, and propodeum with sparse and indistinct rugofoveolae (Fig. 10E, H)	***Pheidole analavelona* sp. nov.**
–	Major workers. Frons with thick, irregular rugae, interspaces smooth and sometimes with indistinct rugulae (Fig. 10A). Minor workers. Head with sparse foveolae, frons with foveolae reduced to absent medially, area posterolateral from eyes with reduced sculpture, predominantly smooth, mesonotum, anepisternum, katepisternum and propodeum predominantly smooth, with sparse and indistinct foveolae on anterolateral and anterior sides (Fig. 10D, G)	***Pheidole ambohimanga* sp. nov.**
24	Major and minor workers. Body uniformly yellow or minors yellow and majors orange (Fig. 11)	**25**
–	At least major workers with darker with legs and antenna distinctly brighter (Figs 12, 13)	**27**
25	Major workers. Body orange, lateral sides of head slightly convex, lateral sides of frons with distinctly irregular rugae, large species HL > 1.1, WL > 1.05 (Fig. 11A). Minor workers. Head predominantly foveolate, vertex and lateral sides of frons with additional, irregular to arcing rugae, large species HL > 0.65, WL > 0.8 (Fig. 11D)	***Pheidole befotaka* sp. nov.**
–	Major workers. Body yellow, lateral sides of head relatively straight, lateral sides of frons distinctly longitudinally irregular, smaller species HL < 1.05, WL < 1.05 (Fig. 11B, C). Minor workers. Head predominantly smooth, if foveolae then without additional rugae, smaller species HL < 0.6, WL < 0.75 (Fig. 11E, F)	**26**
26	Major workers. Medial part of frons with moderately dense and thin rugae, inner hypostomal teeth large, triangular, with rounded apex directed outward and outer hypostomal teeth lobe-like, lower, and more narrow than inner hypostomal teeth (Fig. 11C, H). Minor workers. Head foveolate, only area posterolateral from eyes smooth (Fig. 11F)	***Pheidole vony* sp. nov.**
–	Major workers. Medial part of frons with moderately sparse and thick rugae, inner hypostomal teeth small, triangular, with rounded apex directed upward and outer hypostomal teeth lobe-like, wider and higher than inner hypostomal teeth (Fig. 11B, G). Minor workers. Head foveolate, medial part of frons and vertex with strongly reduced sculpture and predominantly smooth, area posterolateral from eyes smooth (Fig. 11E)	***Pheidole mamiratra* sp. nov.**
27	Major workers. Medial part of frons with thick, interrupted, dense, and irregular to longitudinally irregular rugae with indistinctly to distinctly rugulate interspaces, base of first gastral tergite smooth to shagreened (Fig. 12A–C, G, H). Minor workers. Mesosoma foveolate, sometimes with smooth notch on katepisternum, propodeal spines minute (Fig. 12D–F)	**28**
–	Major workers. Anteromedial part of frons with thick, interrupted, dense to sparse and predominantly longitudinal rugae with smooth to rugulate interspaces, posteromedial frons with rugae irregular, base of first gastral tergite always smooth (Fig. 13A–C). Minor workers. Mesosoma with smooth areas more expanded, if only katepisternum with smooth notch then propodeal spines well developed (Fig. 13G–I)	**30**
28	Major workers. Body ferruginous, frons with distinctly rugulate interspaces (Fig. 12A). Minor workers. Body yellow, katepisternum entirely foveolate (Fig. 12D)	***Pheidole anomala* sp. nov.**
–	Major workers. Body brown to blackish brown, frons with interspaces predominantly smooth or indistinctly rugulate (Fig. 12B, C). Minor workers. Body brown to dark brown, if yellowish brown then katepisternum with smooth notch (Fig. 12E, F)	**29**
29	Major workers. Body brownish black, base of first gastral tergite shagreened (Fig. 12B, G). Minor workers. Mesosoma distinctly foveolate, body dark brown (Fig. 12E)	***Pheidole trichotos* sp. nov.**
–	Major workers. Body brown to dark brown, base of first gastral tergite smooth (Fig. 12C, H). Minor workers. Body yellowish brown to brown, if dark brown then katepisternum at least with smooth notch (Fig. 12F)	***Pheidole veteratrix* Forel**
30	Major workers. Sides of the head with moderately dense, short, decumbent to subdecumbent pilosity, lateral sides of pronotum and katepisternum with smooth notches, body ferruginous (Fig. 13A, D). Minor workers. Body dark orange, mesosomal dorsum rugofoveolate, lateral sides of pronotum foveolate, anepisternum and katepisternum smooth, lateral sides of propodeum with smooth notches (13G)	***Pheidole anosyenne* sp. nov.**
–	Major workers. Sides of the head with sparse to moderately sparse, moderately long to long, decumbent to erect pilosity, body brown, mesosoma with no smooth notches (Fig. 13B, C, E, F). Minor workers. Body brown to bright brown, mesosoma entirely foveolate with smooth notch on katepisternum or mesosoma with sparse foveolae and dorsal promesonotum and medial parts of lateral sides of pronotum, propodeum and katepisternum with smooth notches (Fig. 13H, I)	**31**
31	Major workers. Frons with interspaces distinctly rugofoveolate, rugae on posteromedial part directed outward, lateral sides of frons with thick, sparse, and irregular rugae with distinctly rugofoveolate interspaces (Fig. 13B, E). Minor workers. Mesosoma foveolate, katepisternum with big smooth notch (Fig. 13H)	***Pheidole joffreville* sp. nov.**
–	Major workers. Frons with interspaces with sparse rugofoveolae, lateral sides of frons with dense, thick, predominantly irregular rugae with few distinct longitudinal rugae, interspaces with sparse rugulae, gaster with slightly shagreened base of first tergite (Fig. 13C, F). Minor workers. Mesosoma with sparse foveolae, dorsal promesonotum and medial parts of lateral sides of pronotum, propodeum, and katepisternum with smooth notches (Fig. 13I)	***Pheidole mivory* sp. nov.**
32	Major workers. Impressed and smooth concavity placed lateral to antennal socket and tentorial pit present (Fig. 14E). Minor workers. Body yellow to orange, head always with reduced sculpture, mesosomal dorsum with few transverse, thick rugulae and propodeum with indistinct and sparse foveolae, propodeal spines very small, triangular, promesonotal groove present, promesonotum moderately high, short (15E, J)	***Pheidole sikorae* Forel**
–	Major workers. Impressed and smooth concavity placed lateral to antennal socket and tentorial pit absent (Figs 14A–D, 16). Minor workers. Combination of characters different (Figs 15A–D, F–I, 18)	**33**
33	Major workers. Head in full-face view widening posteriorly, with lateral sides distinctly convex (Fig. 14A–D). Minor workers. Body brown to dark brown, head predominantly foveolate, mesosoma predominantly smooth and propodeal spines minute or body yellow to orange, promesonotal groove deep, mesosoma predominantly or entirely smooth (Fig. 15A–D, F–I)	**34**
–	Major workers. Head in full-face view not widening posteriorly, with lateral sides relatively straight or slightly convex (Fig. 16). Minor workers. Body brown to dark brown, head predominantly foveolate and mesosoma foveolate with smooth notch on katepisternum or mesosoma predominantly smooth and propodeal spines moderate or body yellow to orange, promesonotal groove absent or indistinct, if present then mesosoma with distinct foveolae (Fig. 18)	**37**
34	Major and minor workers. Body colouration bright yellow to orange (Figs 14A, C, 15A, C, F, H)	**35**
–	Major and minor workers. Body colouration darker, brown to dark brown. Sometimes minors yellowish brown (Figs 14B, J, D, K, B, G, D, I)	**36**
35	Major workers. Sides of the head with dense, short, suberect pilosity, lateral sides of frons with longitudinally irregular and thick rugae with distinctly rugofoveolate interspaces, gaster with sparse pilosity, outer hypostomal teeth distinct and wide (Fig. 14A, F, H). Minor workers. Head foveolate with additional indistinct rugulae on frons and vertex, area posterolateral from eyes with reduced sculpture, posteriormost parts predominantly smooth, promesonotum moderately high and short (Fig. 15A, F)	***Pheidole antranohofa* sp. nov.**
–	Major workers. Sides of the head with very dense, short, suberect to erect pilosity, lateral sides of frons with thick, dense, and irregular rugae with sparsely rugulate interspaces, gaster with very dense pilosity, outer hypostomal teeth reduced (Fig. 14C, G, I). Minor workers. Head smooth, lateral sides of frons with longitudinal, short, and thick rugae, vertex with very short, sparse, and transverse rugae, promesonotum moderately low and moderately long (Fig. 15C, H)	***Pheidole mavohavoana* sp. nov.**
36	Major workers. Sides of the head with dense, short, suberect pilosity, interspaces on medial frons with sparse and distinct rugulae, dorsal and lateral sides of pronotum with smooth notches (Fig. 14D, K). Minor workers. Head foveolate with additional indistinct longitudinal rugulae on medial frons, area posterolateral from eyes smooth, mesosoma predominantly smooth, only promesonotal dorsum with arched rugae and indistinct foveolae, mesonotum and lateral sides of propodeum with indistinct and sparse foveolae (Fig. 15D, I)	***Pheidole sava* sp. nov.**
–	Major workers. Sides of the head with moderately dense, moderately long, suberect to erect pilosity, interspaces on medial frons smooth, pronotum never with smooth notches (Fig. 14B, J). Minor workers. Head predominantly smooth with posterolateral sides of frons with sparse and indistinct foveolae, mesosoma smooth (Fig. 15B, G)	***Pheidole itremo* sp. nov.**
37	Major workers. Head in full-face view sub-oval and elongate with lateral sides slightly convex and narrowing posteriorly, medial frons with sparse, interrupted, and longitudinal rugae with predominantly smooth interspaces, lateral sides of frons with dense, irregular rugae with distinctly foveolate interspaces (Fig. 16E, G). Minor workers. Promesonotal groove present but indistinct, body yellowish brown and head and mesosoma entirely or predominantly smooth or body brown and head with sparse foveolae and smooth notches on frons and mesosoma with sparse and indistinct foveolae with smooth notches on dorsum and anepisternum and katepisternum (Fig. 18E, G, N, P)	**38**
–	Major workers. Head in full-face view sub-oval but not elongate with lateral sides relatively straight or slightly convex but never narrowing posteriorly, medial frons with dense, sometimes interrupted and longitudinal rugae with predominantly sculptured interspaces, lateral sides of frons with dense to sparse longitudinal to longitudinally irregular rugae with interspaces smooth to rugoreticulate or rugofoveolate (Fig. 16A–D, F, H, I). Body yellow, if yellowish brown then head and mesosoma predominantly with dense foveolae, if brown then head and mesosoma predominantly with dense foveolae or mesosoma almost entirely smooth and promesonotal groove absent (Fig. 18A–-D, F, H, I, J–M, O, Q, R)	**39**
38	Major workers. Medial part of frons with interspaces finely foveolate, lateral sides with more longitudinally irregular rugae, body ferruginous, promesonotum never with reduced sculpture (Figs 16G, 17F). Minor workers. Head predominantly smooth or foveolae with foveola sparse and fading on medial frons and area posterolateral from eyes; vertex with sparse and short rugulae; frons with very sparse, short, and irregular rugae; mesosoma with sparse rugofoveolae; promesonotal dorsum and katepisternum with smooth notches or smooth; pronotum with additional sparse, short, and transverse rugulae (Fig. 18G, P)	***Pheidole sofia* sp. nov.**
–	Major workers. Medial part of frons with smooth or indistinct rugofoveolae, lateral sides of frons with rugae predominantly irregular, body brownish to dark orange, promesonotum with reduced sculpture (Figs 16E, 17E). Minor workers. Head predominantly smooth; frons with longitudinal, short, and thick rugae and sometimes foveolate interspaces, promesonotum predominantly smooth (Fig. 18E, N)	***Pheidole manantenina* sp. nov.**
39	Major workers. Base of first gastral tergite shagreened (Fig. 17H, I). Minor workers. Scape, when laid back, exceeding the posterior head margin by one-third of its length, SI > 157.0, promesonotal groove present, promesonotum predominantly sculptured, vertex without arching rugae (Fig. 18F, I, O, R)	**40**
–	Major workers. Base of first gastral tergite smooth. Minor workers. Scape, when laid back, exceeding the posterior head margin by one- to two-fifths of its length, SI < 157.0, if exceeds by one-third of its length then promesonotal groove absent and promesonotum predominantly smooth or promesonotal groove present and vertex with distinct, arching rugae (Fig. 18A–-D, H, J–M, Q)	**41**
40	Major workers. Body yellow, promesonotal dorsum with short, transverse rugae with smooth to indistinctly foveolate interspaces (Fig. 17J). Minor workers. Body yellowish brown, dorsal side of promesonotum and lateral sides of pronotum with reduced sculpture and smooth notches on medial parts (Fig. 18F, 0)	***Pheidole renirano* sp. nov.**
–	Major workers. Body ferruginous, promesonotal dorsum with short, transverse, and more irregular rugae with distinctly rugofoveolate interspaces (Fig. 17K). Minor workers. Body brown, dorsal side of promesonotum and lateral sides of pronotum with sparse foveolae but never with smooth notches (Fig. 18I, R)	***Pheidole tsaravoniana* sp. nov.**
41	Major workers. Body brown, lateral sides of pronotum with distinct sculpture (Fig. 17D). Minor workers. Head with distinct, arcing rugae on vertex, body brown (Fig. 18D, M)	***Pheidole mahamavo* sp. nov.**
–	Major workers. Body yellow to orange, lateral sides of pronotum with smooth notches or sparse sculpture with smooth interspaces (Fig. 17A–C, G). Minor workers. Head never with distinct, arcing rugae on vertex, body yellow (Fig. 18A–-C, H, J–L, Q)	**42**
42	Major workers. Area posterolateral from eyes with sculpture weakening posteriorly and smooth notch (Fig. 17B). Minor workers. Head and mesosoma predominantly smooth and never foveolate (Fig. 18B, K)	***Pheidole haboka* sp. nov.**
–	Major workers. Area posterolateral from eyes with sculpture well developed and never with smooth notch (Fig. 17A, C, G). Minor workers. At least head predominantly foveolate, mesosoma at least partially foveolate (Fig. 18A, C, H, J, L, Q)	**43**
43	Major workers. Medial part of frons with moderately sparse rugae, interspaces with sparse and indistinct rugofoveolae, promesonotum predominantly smooth, with indistinct rugofoveolae on lateral sides (Figs 16H, 17G). Minor workers. Body dark yellow, scape, when laid back, surpassing the posterior head margin by one-fifth of its length, SI < 123.0, pronotum with very indistinct and sparse foveolae; mesonotum, anepisternum, katepisternum, and propodeum smooth (Fig. 18H, Q)	***Pheidole sparsa* sp. nov.**
–	Major workers. Medial part of frons with moderately dense to dense rugae, interspaces with distinct rugofoveolae or indistinct foveolae, promesonotum entirely rugofoveolate and sometimes with smooth notches on lateral sides (Figs 16A, C, 17A, C). Minor workers. Body brown and scape, when laid back, exceeding the posterior head margin by one-third of its length, SI > 153.0, or body yellow, scape, when laid back, surpassing the posterior head margin by one-fifth of its length, SI < 123.0, and mesosoma distinctly foveolate and lateral sides of propodeum, katepisternum, and anepisternum with smooth notches (Fig. 18A, C, J, L)	**44**
44	Major workers. Interspaces on medial frons distinctly rugofoveolate, propodeal spines moderately long and relatively narrow, pronotum with no smooth notches (Figs 16C, 17C). Minor workers. Scape, when laid back, surpassing the posterior head margin by one-fifth of its length, SI < 123.0, mesosoma with thick foveolae; lateral sides of propodeum, katepisternum, and anepisternum with smooth notches, body yellow (Fig. 18C, L)	***Pheidole hazo* sp. nov.**
–	Major workers. Interspaces on frons indistinctly foveolate, propodeal spines short, with wide base, lateral sides of pronotum with smooth notches (Figs 16A, 17A). Minor workers. Scape, when laid back, exceeding the posterior head margin by one-third of its length, SI < 153.0, mesosoma predominantly smooth, lateral sides with very sparse, indistinct, and thin rugulae, body brown (Fig. 18A, J)	***Pheidole dasos* sp. nov.**

**Figure 2.**
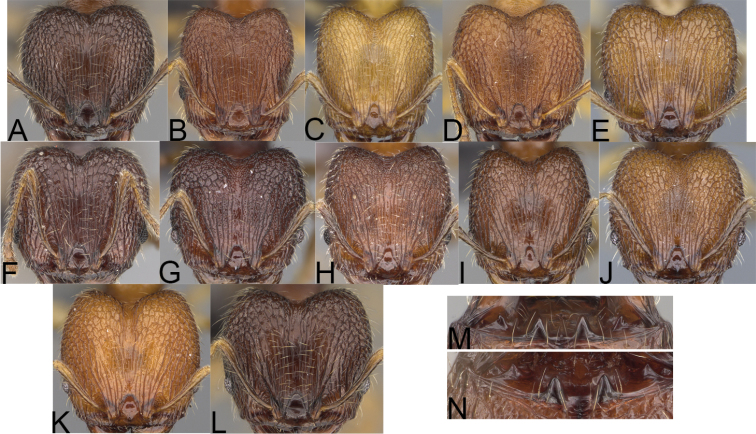
Major worker, head in full-face view. *Pheidole andohahela* sp. nov. (**A**). *Pheidole beanka* sp. nov. (**B**). *Pheidole flavominuta* sp. nov. (**C**). *Pheidole havoana* sp. nov. (**D**). *Pheidole kely* sp. nov. (**E**). *Pheidole lavasoa* sp. nov. (**F**). *Pheidole midongy* sp. nov. (**G**). *Pheidole mikros* sp. nov. (**H**). *Pheidole nitidobruna* sp. nov. (**I**). *Pheidole parvula* sp. nov. (**J**). *Pheidole parvulogibba* sp. nov. (**K**). *Pheidole volontany* sp. nov. (**L**). Major workers, hypostomal teeth. *Pheidole andohahela* sp. nov. (**M**). *Pheidole lavasoa* sp. nov. (**N**).

**Figure 3.**
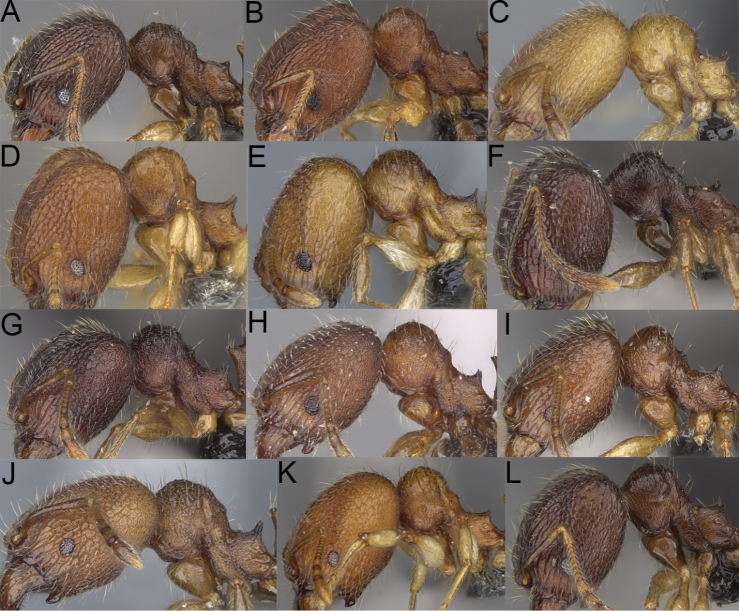
Major worker, profile. *Pheidole andohahela* sp. nov. (**A**). *Pheidole beanka* sp. nov. (**B**). *Pheidole flavominuta* sp. nov. (**C**). *Pheidole havoana* sp. nov. (**D**). *Pheidole kely* sp. nov. (**E**). *Pheidole lavasoa* sp. nov. (**F**). *Pheidole midongy* sp. nov. (**G**). *Pheidole mikros* sp. nov. (**H**). *Pheidole nitidobruna* sp. nov. (**I**). *Pheidole parvula* sp. nov. (**J**). *Pheidole parvulogibba* sp. nov. (**K**). *Pheidole volontany* sp. nov. (**L**).

**Figure 4.**
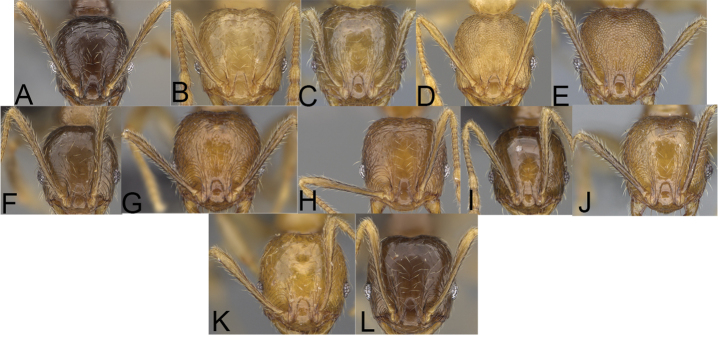
Minor worker, head in full-face view. *Pheidole andohahela* sp. nov. (**A**). *Pheidole beanka* sp. nov. (**B**). *Pheidole flavominuta* sp. nov. (**C**). *Pheidole havoana* sp. nov. (**D**). *Pheidole kely* sp. nov. (**E**). *Pheidole lavasoa* sp. nov. (**F**). *Pheidole midongy* sp. nov. (**G**). *Pheidole mikros* sp. nov. (**H**). *Pheidole nitidobruna* sp. nov. (**I**). *Pheidole parvula* sp. nov. (**J**). *Pheidole parvulogibba* sp. nov. (**K**). *Pheidole volontany* sp. nov. (**L**).

**Figure 5.**
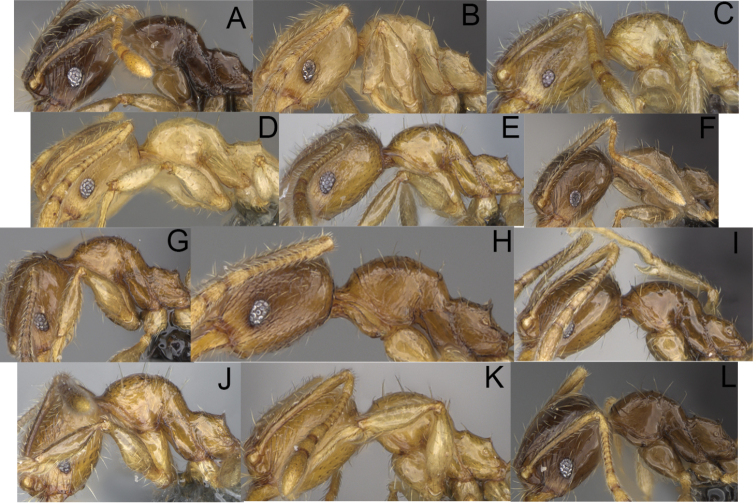
Minor worker, profile. *Pheidole andohahela* sp. nov. (**A**). *Pheidole beanka* sp. nov. (**B**). *Pheidole flavominuta* sp. nov. (**C**). *Pheidole havoana* sp. nov. (**D**). *Pheidole kely* sp. nov. (**E**). *Pheidole lavasoa* sp. nov. (**F**). *Pheidole midongy* sp. nov. (**G**). *Pheidole mikros* sp. nov. (**H**). *Pheidole nitidobruna* sp. nov. (**I**). *Pheidole parvula* sp. nov. (**J**). *Pheidole parvulogibba* sp. nov. (**K**). *Pheidole volontany* sp. nov. (**L**).

**Figure 6.**
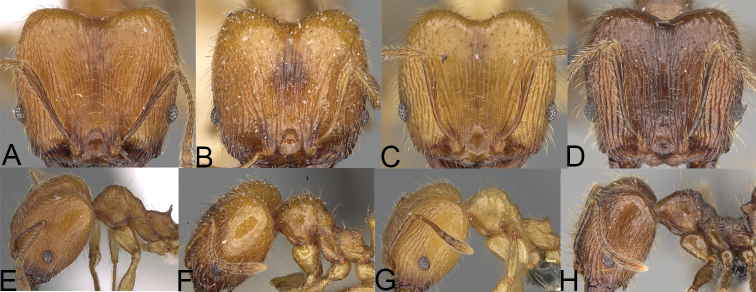
Major worker. *Pheidole gracilis* sp. nov., head (**A**), profile (**E**). *Pheidole litigiosa* Forel, head (**B**), profile (**F**). *Pheidole masoandro* sp. nov., head (**C**), profile (**G**). *Pheidole tampony* sp. nov., head (**D**), profile (**H**).

**Figure 7.**
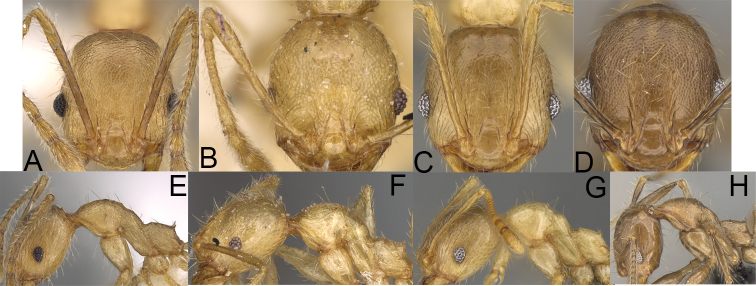
Minor worker. *Pheidole gracilis* sp. nov., head (**A**), profile (**E**). *Pheidole litigiosa* Forel, head (**B**), profile (**F**). *Pheidole masoandro* sp. nov., head (**C**), profile (**G**). *Pheidole tampony* sp. nov., head (**D**), profile (**H**).

**Figure 8.**
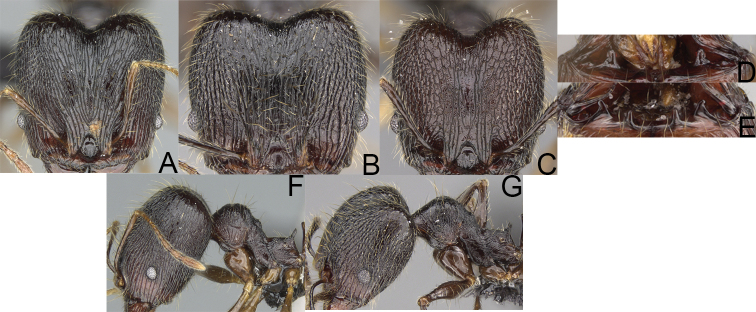
Major worker. *Pheidole alina* sp. nov., head (**A**), hypostomal teeth (**D**), profile (**F**). *Pheidole mainty* sp. nov., head (**B**), hypostomal teeth (**E**), profile (**G**). *Pheidole trichotos* sp. nov., head (**C**).

**Figure 9.**
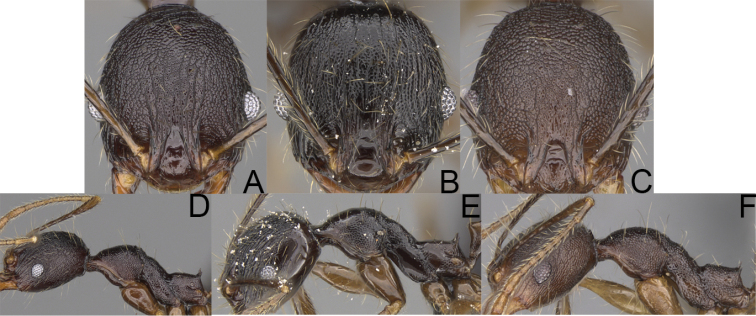
Minor worker. *Pheidole alina* sp. nov., head (**A**), profile (**D**). *Pheidole mainty* sp. nov., head (**B**), profile (**E**). *Pheidole trichotos* sp. nov., head (**C**), profile (**F**).

**Figure 10.**
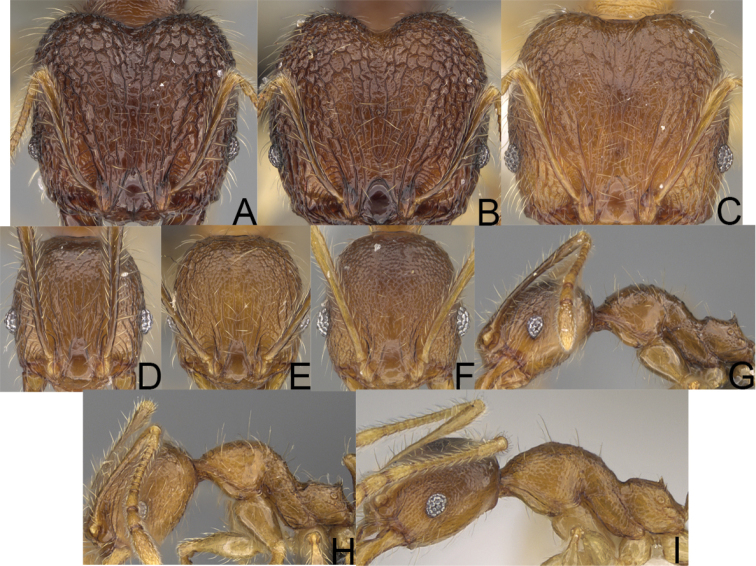
*Pheidole ambohimanga* sp. nov. Major worker, head (**A**). Minor worker, head (**D**), profile (**G**). *Pheidole analavelona* sp. nov. Major worker, head (**B**). Minor worker, head (**E**), profile (**H**). *Pheidole vadum* sp. nov. Major worker, head (**C**). Minor worker, head (**F**), profile (**I**).

**Figure 11.**
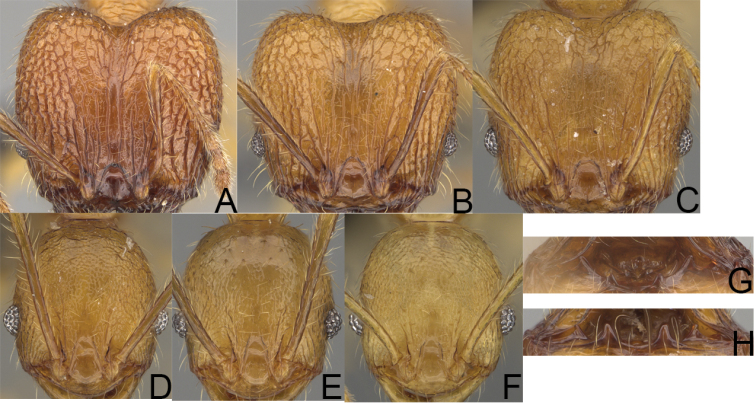
*Pheidole befotaka* sp. nov. Major worker, head (**A**). Minor worker, head (**D**). *Pheidole mamiratra* sp. nov. Major worker, head (**B**), hypostomal teeth (**G**). Minor worker, head (**E**). *Pheidole vony* sp. nov. Major worker, head (**C**), hypostomal teeth (**H**). Minor worker, head (**F**).

**Figure 12.**
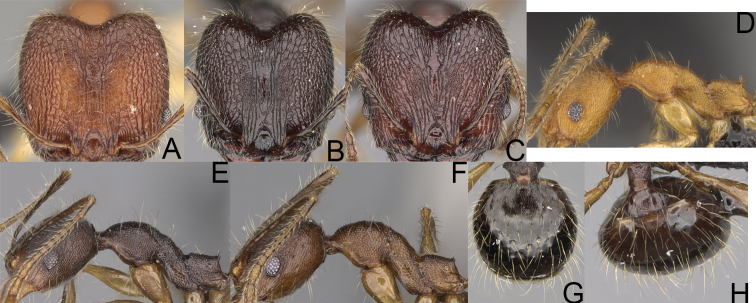
*Pheidole anomala* sp. nov. Major worker, head (**A**). Minor worker, profile (**D**). *Pheidole trichotos* sp. nov. Major worker, head (**B**), gaster (**G**). Minor worker, profile (**E**). *Pheidole veteratrix* Forel. Major worker, head (**C**), gaster (**H**). Minor worker, profile (**F**).

**Figure 13.**
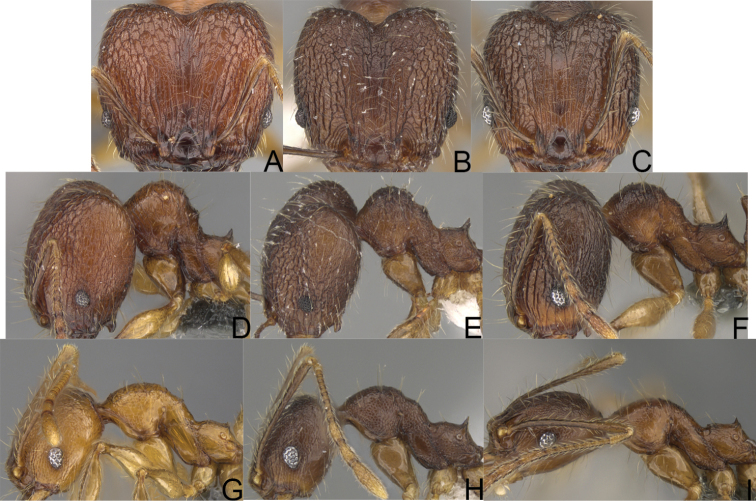
*Pheidole anosyenne* sp. nov. Major worker, head (**A**), profile (**D**). Minor worker, profile (**G**). *Pheidole joffreville* sp. nov. Major worker, head (**B**), profile (**E**). Minor worker, profile (**H**). *Pheidole mivory* sp. nov. Major worker, head (**C**), profile (**F**). Minor worker, profile (**I**).

**Figure 14.**
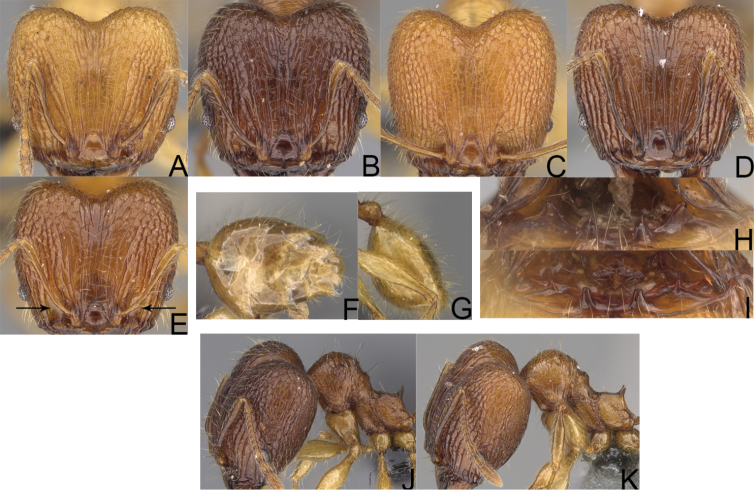
Major worker. *Pheidole antranohofa* sp. nov., head (**A**), gaster (**F**), hypostomal teeth (**H**). *Pheidole itremo* sp. nov., head (**B**), profile (**J**). *Pheidole mavohavoana* sp. nov., head (**C**), gaster (**G**), hypostomal teeth (**I**). *Pheidole sava* sp. nov., head (**D**), profile (**K**). *Pheidole sikorae* Forel, head (**E**).

**Figure 15.**
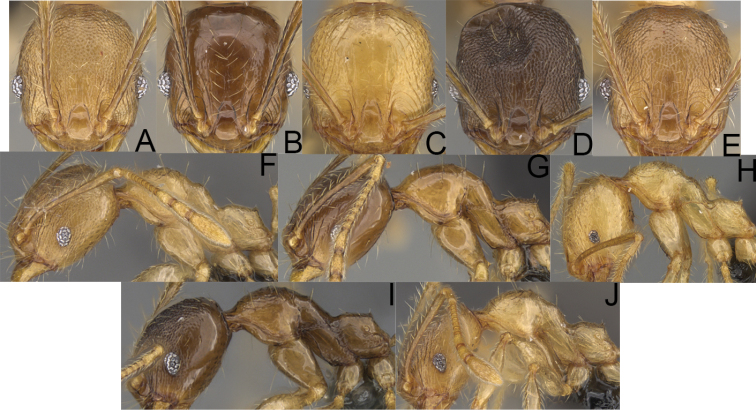
Minor worker. *Pheidole antranohofa* sp. nov., head (**A**), profile (**F**). *Pheidole itremo* sp. nov., head (**B**), profile (**G**). *Pheidole mavohavoana* sp. nov., head (**C**), profile (**H**). *Pheidole sava* sp. nov., head (**D**), profile (**I**). *Pheidole sikorae* Forel, head (**E**), profile (**J**).

**Figure 16.**
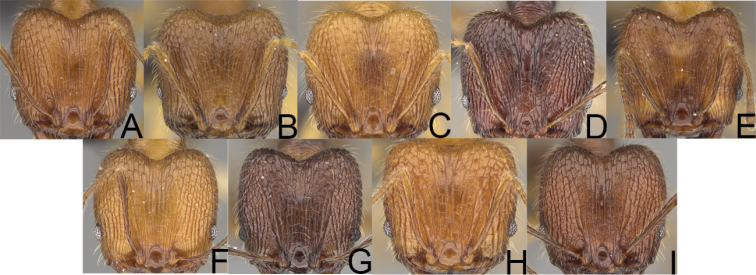
Major worker, head. *Pheidole dasos* sp. nov. (**A**). *Pheidole haboka* sp. nov. (**B**). *Pheidole hazo* sp. nov. (**C**). *Pheidole mahamavo* sp. nov. (**D**). *Pheidole manantenina* sp. nov. (**E**). *Pheidole renirano* sp. nov. (**F**). *Pheidole sofia* sp. nov. (**G**). *Pheidole sparsa* sp. nov. (**H**). *Pheidole tsaravoniana* sp. nov. (**I**).

**Figure 17.**
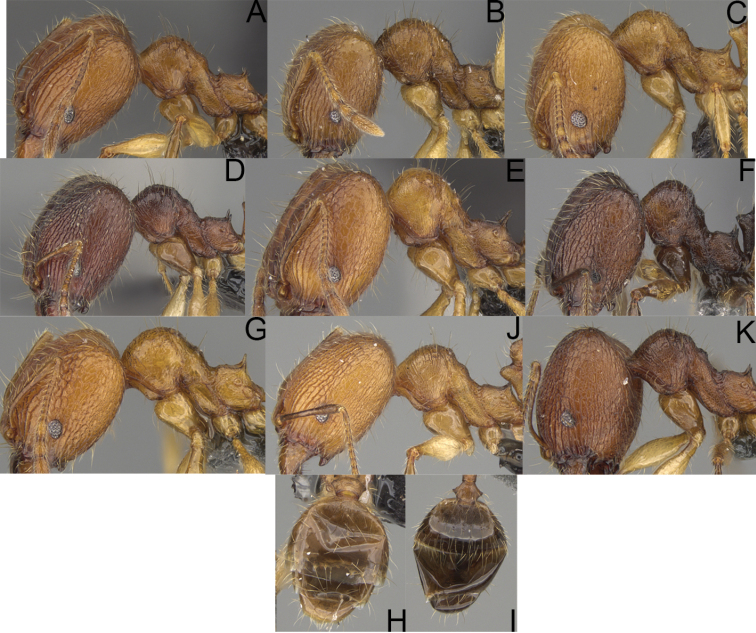
Major worker. *Pheidole dasos* sp. nov., profile (**A**). *Pheidole haboka* sp. nov., profile (**B**). *Pheidole hazo* sp. nov., profile (**C**). *Pheidole mahamavo* sp. nov., profile (**D**). *Pheidole manantenina* sp. nov., profile (**E**). *Pheidole renirano* sp. nov., gaster (**H**), profile (**J**). *Pheidole sofia* sp. nov., profile (**F**). *Pheidole sparsa* sp. nov., profile (**G**). *Pheidole tsaravoniana* sp. nov., gaster (**I**), profile (**K**).

**Figure 18.**
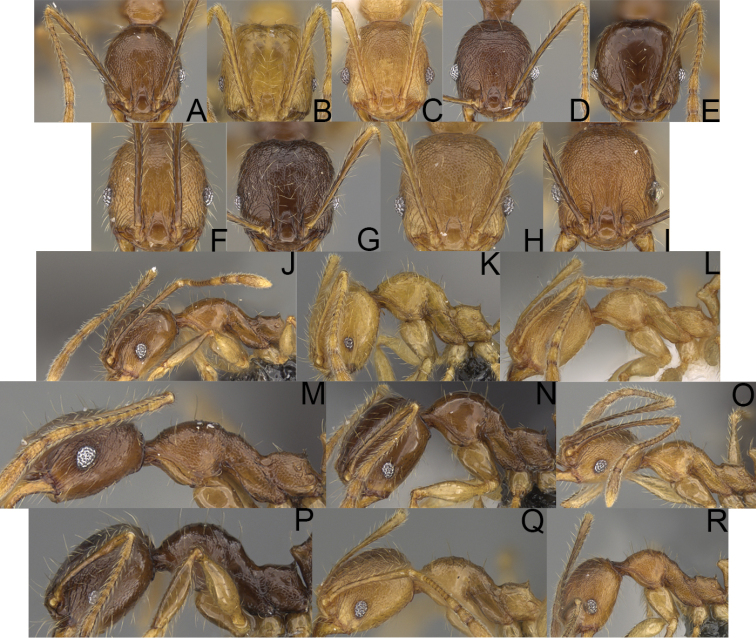
Minor worker. *Pheidole dasos* sp. nov., head (**A**), profile (**J**). *Pheidole haboka* sp. nov., head (**B**), profile (**K**). *Pheidole hazo* sp. nov., head (**C**), profile (**L**). *Pheidole mahamavo* sp. nov., head (**D**), profile (**M**). *Pheidole manantenina* sp. nov., head (**E**), profile (**N**). *Pheidole renirano* sp. nov., head (**F**), profile (**O**). *Pheidole sofia* sp. nov., head (**G**), profile (**P**). *Pheidole sparsa* sp. nov., head (**H**), profile (**Q**). *Pheidole tsaravoniana* sp. nov., head (**I**), profile (**R**).

#### Pheidole alina sp. nov.

Taxon classificationAnimalia

B887B712-8256-56B6-A23F-16BE6BBF0F33

http://zoobank.org/760CE832-A353-4B5A-986F-550C89D6197E

**Figure 19.**
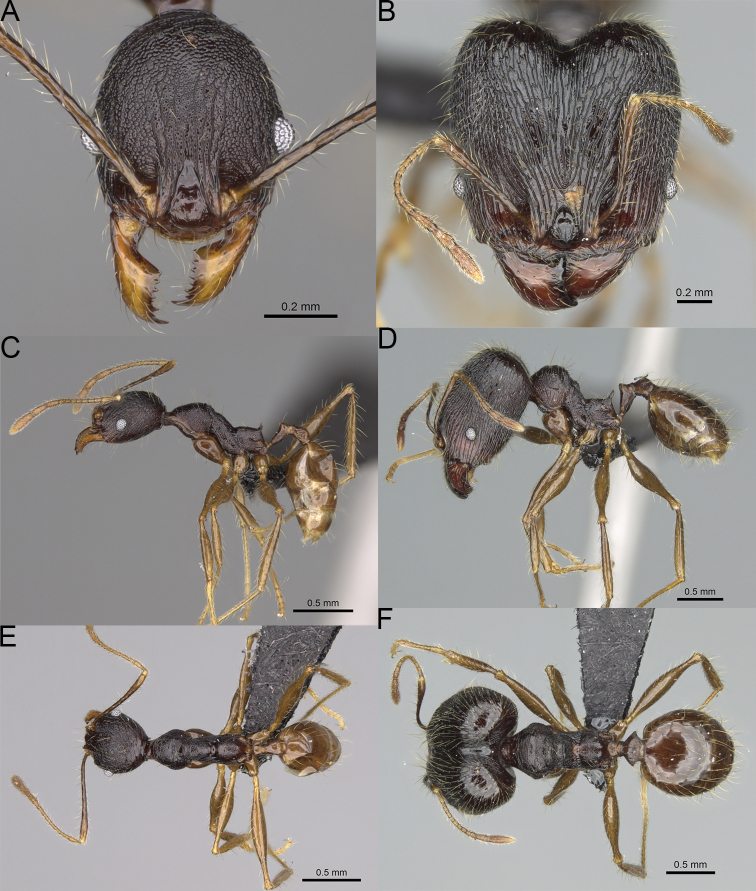
*Pheidole alina* sp. nov., full-face view (**A**), profile (**C**), and dorsal view (**E**) of paratype minor worker (CASENT0487334) and full-face view (**B**), profile (**D**), and dorsal view (**F**) of holotype major worker (CASENT0923257).

Figs 19A–F , 63A , 65A

##### Type material.

***Holotype.*** Madagascar. • 1 major worker; Antsiranana; Parc National de Marojejy, 25.7 km 32°NNE Andapa, 10.3 km 314°NW Manantenina; -14.445, 49.74167; alt. 1575 m; 22 Nov 2003; B. L. Fisher leg.; montane rainforest, ex rotten log; BLF09287; CASENT0923257, top specimen on the pin (CASC). ***Paratypes.*** • 6w., 2s.; same data as for holotype, CASENT0487334, CASENT0487336, CASENT0487335 (CASC, MHNG, PBZT).

##### Other material.

Madagascar. –**Antsiranana**: • 6w., 3s.; Parc National de Marojejy, 25.4 km 30°NNE Andapa, 10.9 km 311°NW Manantenina; -14.445, 49.735; alt. 2000 m; 23 Nov 2003; B. L. Fisher leg.; montane shrubland, under moss, on ground; BLF09330 (CASC). •1w.; Parc National de Marojejy, 25.7 km 32°NNE Andapa, 10.3 km 314°NW Manantenina; -14.445, 49.74167; alt. 1575 m; 21 Nov 2003; B. L. Fisher leg.; montane rainforest; sifted litter (leaf mold, rotten wood); BLF09242 (CASC). • 6w.; Parc National de Marojejy, 25.7 km 32°NNE Andapa, 10.3 km 314°NW Manantenina; -14.445, 49.74167; alt. 1575 m; 22 Nov 2003; B. L. Fisher leg.; montane rainforest, under moss, above ground; BLF09264 (CASC). • 6w., 3s.; Parc National de Marojejy, 25.7 km 32°NNE Andapa, 10.3 km 314°NW Manantenina; -14.445, 49.74167; alt. 1575 m; 22 Nov 2003; B. L. Fisher leg.; montane rainforest, ex rotten log; BLF09302 (CASC). • 3w.; Parc National de Marojejy, 25.7 km 32°NNE Andapa, 10.3 km 314°NW Manantenina; -14.445, 49.74167; alt. 1575 m; 22 Nov 2003; B. L. Fisher leg.; montane rainforest, ex Melastomataceae; BLF09318 (CASC). • 3w.; Parc National de Marojejy, Antranohofa, 26.6 km 31°NNE Andapa, 10.7 km 318°NW Manantenina; -14.44333, 49.74333; alt. 1325 m; 19 Nov 2003; B. L. Fisher leg.; montane rainforest, under moss, above ground; BLF09142 (CASC). • 1w.; Prov. Antsiranana R.S. Manongarivo 17.3 km 218°SW Antanambao; -14.02167, 48.41833; alt. 1580 m; 27 Oct 1998; B. L. Fisher leg.; montane rainforest, sifted litter (leaf mold, rotten wood); BLF01970 (CASC). • 3w.; Prov. Antsiranana R.S. Manongarivo 17.3 km 218°SW Antanambao; -14.02167, 48.41833; alt. 1580 m; 27 Oct 1998; B. L. Fisher leg.; montane rainforest; BLF01972 (CASC). • 1w.; R.S. Manongarivo, 20.4 km 219°SW Antanambao; -14.04667, 48.40167; alt. 1860 m; 3 Nov 1998; B. L. Fisher leg.; montane rainforest; BLF01989 (CASC). –**Mahajanga**: • 10w.; Region Sofia, Ampotsidia; -14.40775, 48.70201; alt. 1625 m; 10 Jan 2017; B. L. Fisher leg.; montane rainforest, sifted litter; BLF39733 (CASC).

##### Diagnosis.

Moderately large species. ***Major workers.*** Head in full-face view sub-oval and slightly widening posteriorly, with anterior and posterior sides slightly convex, in lateral view sub-oval; ventral and dorsal faces convex; sides of the head with moderately dense, long, suberect to erect pilosity; medial part of frons with thick, interrupted, dense, and longitudinal rugae with smooth to indistinctly rugulate interspaces; occipital lobes and area posterolateral from eyes without smooth notches; scape, when laid back, exceeding the midlength of head by one-fifth of its length; inner hypostomal teeth indistinct, low, and wide, closely spaced, bulge-like; outer hypostomal teeth lobe-like, wider and higher than inner teeth, apex directed upward; inner and outer hypostomal teeth closely spaced and not connected by concavity; mesosoma rugofoveolate; pronotum with additional thin, moderately dense, and transverse rugae; katepisternum with reduced sculpture and sometimes with smooth notch; gaster smooth; body black. ***Minor workers.*** Head foveolate; median frons with short, indistinct, and longitudinal rugulae; area posterolateral from eyes with slightly to distinctly weaker sculpture; scape, when laid back, exceeding the posterior head margin by one-third of its length; promesonotum low and moderately long; promesonotal groove present; propodeal spines small and triangular; mesosoma foveolate; katepisternum with reduced sculpture or with smooth notch; body black to dark brown.

##### Description.

**Major workers.** Measurements (*N* = 9): HL: 1.3–1.79 (1.61); HW: 1.28–1.73 (1.56); SL: 0.88–1.05 (0.97); EL: 0.18–0.22 (0.19); WL: 1.22–1.48 (1.36); PSL: 0.22–0.28 (0.26); MTL: 0.91–1.07 (1.01); PNW: 0.54–0.7 (0.61); PTW: 0.16–0.22 (0.2); PPW: 0.35–0.53 (0.44); CI: 100.7–104.7 (102.9); SI: 57.5–70.4 (62.9); PSLI: 14.9–17.7 (16.1); PPI: 41.6–49.1 (45.1); PNI: 36.3–43.7 (39.4); MTI: 60.3–73.4 (65.4).

***Head.*** In full-face view sub-oval, slightly widening posteriorly, with anterior and posterior sides convex (Fig. 19B). In lateral view sub-oval; ventral and dorsal faces convex; inner hypostomal teeth visible. Sides of the head with moderately dense, long, suberect to erect pilosity; whole head with dense, long, decumbent to erect pilosity. Medial part of frons with thick, interrupted, dense, and longitudinal rugae with smooth to indistinctly rugulate interspaces; lateral sides of frons with denser, thick, and longitudinal rugae, interspaces with dense and moderately thick rugulae. Occipital lobes with longitudinal and thinner rugae and indistinctly rugulate to smooth interspaces. Area posterolateral from eyes with dense, thick, and longitudinal rugae with distinctly rugulate interspaces, sculpture fading posteriorly. Gena with relatively sparse, thick, and longitudinal rugae with indistinctly rugulate interspaces. Centre of clypeus smooth and shiny, lateral sides with indistinct rugulae; median notch present, moderately wide, and shallow; median longitudinal carina present; lateral longitudinal carinae absent. Scape, when laid back, exceeding the midlength of head by one-fifth of its length; pilosity subdecumbent to erect (Fig. 19B, D). Inner hypostomal teeth indistinct, low, and wide, closely spaced, bulge-like; outer hypostomal teeth lobe-like, wider, and higher than inner teeth, apex directed upward; inner and outer hypostomal teeth closely spaced and not connected by concavity (Fig. 63A). ***Mesosoma.*** In lateral view, promesonotum short, angular, and moderately low, posterior mesonotum moderately steep, mesonotal process indistinct, tubercle-like; promesonotal groove absent; metanotal groove absent; propodeal spines moderately long, narrow, and with acute apex; humeral area weakly produced (Fig. 19D). Surface shiny and rugofoveolate; pronotum with additional thin, moderately dense, and transverse rugae; katepisternum with reduced sculpture and sometimes with smooth notch. Pilosity moderately dense, long, and erect (Fig. 19D, F). ***Petiole.*** Shiny with sparse foveolae; node finely foveolate to smooth, triangular, with rounded and thick apex, in rear view node dorsoventrally slightly convex; pilosity moderately sparse and erect (Fig. 19D, F). ***Postpetiole.*** Shiny and foveolate; dorsum with reduced sculpture and smooth notch; in dorsal view oval, lateral margins medially with two wide and dentate projections; pilosity long, moderately sparse, and erect (Fig. 19D, F). ***Gaster.*** Shiny and smooth; pilosity moderately sparse, long, and erect (Fig. 19D, F). ***Colour.*** Black, gena and posterior parts of lateral sides of promesonotum reddish, gaster, antenna, and legs yellowish (Fig. 19D, F).

**Minor workers.** Measurements (*N* = 10): HL: 0.65–0.85 (0.78); HW: 0.5–0.65 (0.61); SL: 0.87–1.07 (1.04); EL: 0.12–0.16 (0.14); WL: 0.89–1.12 (1.07); PSL: 0.11–0.17 (0.15); MTL: 0.72–0.89 (0.86); PNW: 0.37–0.47 (0.44); PTW: 0.08–0.13 (0.11); PPW: 0.15–0.21 (0.18); CI: 123.6–131.1 (127.5); SI: 155.1–172.1 (163.4); PSLI: 16.8–21.0 (19.1); PPI: 45.9–69.9 (58.8); PNI: 69.0–75.0 (71.8); MTI: 136.3–143.7 (139.7).

***Head.*** Cephalic margin slightly convex (Fig. 19A). Pilosity relatively sparse, short, and subdecumbent. Sculpture shiny and foveolate; median frons with short and indistinct longitudinal rugulae; area posterolateral from eyes with slightly to distinctly weaker sculpture; antennal sockets with few indistinct, curved outward rugae and foveolate interspaces. Clypeus with median longitudinal carina absent; two lateral longitudinal carinae absent. Scape, when laid back, exceeding the posterior head margin by one-third of its length; pilosity dense, subdecumbent to erect (Fig. 19A, C). ***Mesosoma.*** In lateral view, promesonotum low and moderately long, arched; promesonotal groove indistinct; metanotal groove distinct; propodeal spines small and triangular (Fig. 19C). Sculpture shiny and foveolate; katepisternum with reduced sculpture or with smooth notch. Pilosity sparse, moderately long, and erect (Fig. 19C, E). ***Gaster.*** With sparse, erect pilosity (Fig. 19C, E). ***Colour.*** Black to dark brown, legs, gaster, and antenna yellowish (Fig. 19C, E).

##### Etymology.

Malagasy for night in reference to dark body colouration.

##### Biology.

The species was collected between 1270–2000 m in elevation, in montane rainforest and montane shrubland. Nests were located in rotten logs, under moss above and on the ground, and in a Melastomataceae twig.

##### Comments.

*Pheidole alina* sp. nov. belongs to the group of taxa characterised by dark body colouration, in major workers from brownish black to black and in minor workers from black (head and mesosoma) to dark brown with body predominantly foveolate (in dark brown specimens entirely). The group consists of three species: *P. alina* sp. nov., *P. trichotos* sp. nov., and *P. mainty* sp. nov. All members of this group are sympatric and their distribution is limited to northernmost parts of the island, predominantly in Antsiranana prefecture. Major workers of *Pheidole alina* sp. nov. differ from *P. trichotos* sp. nov. by medial part of frons with thick, interrupted, dense, and longitudinal rugae with smooth to indistinctly rugulate interspaces, base of first gastral tergite smooth and indistinct, bulge-like inner hypostomal teeth; from *P. mainty* sp. nov. in sides of the head with moderately dense, long, suberect to erect pilosity, indistinct, bulge-like inner hypostomal teeth, and narrow and moderately long propodeal spines. Minor workers of *P. alina* sp. nov. differ from *P. trichotos* sp. nov. in larger body size and black body colouration; from *P. mainty* sp. nov. in never entirely smooth area posterolateral from eyes and distinctly foveolate mesosoma with reduced or no foveolae only on the katepisternum.

#### Pheidole ambohimanga sp. nov.

Taxon classificationAnimalia

C721C07E-BB6F-5CCF-BF44-BE17731C04F4

http://zoobank.org/CAEC5D0E-D815-4524-BB3E-4B42E443B3A6

**Figure 20.**
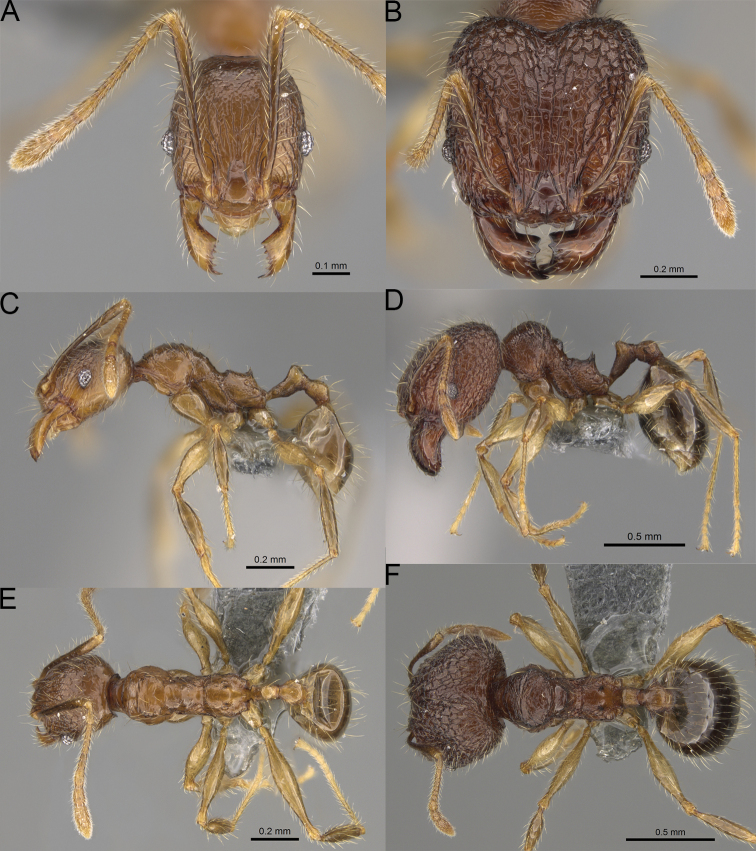
*Pheidole ambohimanga* sp. nov., full-face view (**A**), profile (**C**), and dorsal view (**E**) of paratype minor worker (CASENT0923279) and full-face view (**B**), profile (**D**), and dorsal view (**F**) of holotype major worker (CASENT0720924).

Figs 20A–F , 63B , 65B

##### Type material.

***Holotype.*** Madagascar. • 1 major worker; Antananarivo; Ambohimanga; -18.76036, 47.56372; alt. 1294 m; 8 Sep 2015; B.L. Fisher et al. leg.; montane rainforest, ground nest; BLF37302; CASENT0720924 (CASC). ***Paratypes.*** • 2w., 1s.; same data as for holotype; CASENT0923279, CASENT0720925 (CASC, MHNG).

##### Other material.

Madagascar. –**Antananarivo**: • 2w., 1s.; Ambohitrangano; -18.72801, 47.57878; alt. 1383 m; 3 Oct 2015; B.L. Fisher et al. leg.; disturbed montane rainforest, under root mat, litter on rock; BLF37326 (CASC). • 1w., 1s.; Station Forestière Angavokely; -18.92207, 47.74157; alt. 1460 m; 9 Mar 2013; B.L. Fisher et al. leg.; rainforest, under root mat, litter on rock; BLF31333 (CASC). • 1w., 1s.; Tsimbazaza; -18.928, 47.527; alt. 1300 m; 16 Dec 2006; B.L. Fisher et al. leg.; park/garden, ex rotten log; BLF16240 (CASC).

##### Diagnosis.

Moderately large species. ***Major workers.*** Head in full-face view oval, not widening posteriorly, with anterior and posterior sides convex, in lateral view sub-oval, ventral and dorsal faces convex, occipital cleft moderately deep; sides of the head with dense, very long, erect pilosity; medial part of frons with thick, moderately sparse, and irregular rugae, interspaces predominantly smooth and sometimes with indistinct rugulae; lateral sides of head, area posterolateral from eyes and occipital lobes with relatively dense to dense, thick, and irregular rugae; interspaces predominantly smooth or indistinctly rugulate; scape, when laid back, exceeding the midlength of head by two-fifths of its length; inner hypostomal teeth distinct, large, closely spaced, and triangular, with rounded apex directed inward; outer hypostomal teeth lobe-like, slightly wider than inner hypostomal teeth and approximately the same height, apex directed outward; inner and outer hypostomal teeth closely spaced and not connected by concavity; lateral sides of pronotum, mesonotum, katepisternum, anepisternum, and propodeum rugofoveolate, pronotal dorsum with thick to thin, irregular to transverse rugae with smooth to indistinctly rugofoveolate interspaces; katepisternum with smooth notch; body dark brown. ***Minor workers.*** Head with sparse foveolae; frons with foveolae reduced medially with moderately thick, longitudinal, and interrupted rugae; area posterolateral from eyes with reduced sculpture, predominantly smooth; scape, when laid back, surpassing the posterior head margin by two-fifths of its length; promesonotum moderately high and short; promesonotal groove absent; propodeal spines minute and triangular; propodeum predominantly smooth, its dorsum with sparse, short, and transverse rugae and indistinct foveolae; mesonotum, anepisternum, katepisternum, and propodeum predominantly smooth, with sparse and indistinct foveolae on anterolateral and anterior sides; body brown.

##### Description.

**Major workers.** Measurements (*N* = 4): HL: 0.95–1.03 (0.98); HW: 0.93–1.02 (0.96); SL: 0.61–0.63 (0.62); EL: 0.13–0.13 (0.13); WL: 0.84–0.92 (0.88); PSL: 0.14–0.16 (0.15); MTL: 0.55–0.59 (0.57); PNW: 0.45–0.5 (0.47); PTW: 0.14–0.16 (0.15); PPW: 0.3–0.35 (0.31); CI: 97.5–104.4 (101.5); SI: 62.0–67.0 (64.6); PSLI: 13.2–15.9 (15.0); PPI: 41.1–49.8 (47.2); PNI: 46.2–50.9 (48.8); MTI: 55.6–61.6 (58.9).

***Head.*** In full-face view oval, not widening posteriorly, with anterior and posterior sides convex (Fig. 20B). In lateral view sub-oval; ventral and dorsal faces convex; inner hypostomal teeth visible. Sides of the head with dense, very long, erect pilosity; whole head with dense, long, decumbent to erect pilosity. Medial part of frons with thick, moderately sparse, irregular rugae, interspaces predominantly smooth and sometimes with indistinct rugulae; lateral sides of head, area posterolateral from eyes, and occipital lobes with relatively dense to dense, thick, and irregular rugae; interspaces predominantly smooth or indistinctly rugulate. Gena with relatively dense, thick, and longitudinal rugae and smooth interspaces. Centre of clypeus smooth and shiny, lateral sides with indistinct rugulae; median notch present, moderately wide, and shallow; median longitudinal carina present; lateral longitudinal carinae absent. Scape, when laid back, exceeding the midlength of head by two-fifths of its length; pilosity subdecumbent to erect (Fig. 20B, D). Inner hypostomal teeth distinct, large, closely spaced, triangular, with rounded apex directed inward; outer hypostomal teeth lobe-like, slightly wider than inner hypostomal teeth and approximately the same height, apex directed outward; inner and outer hypostomal teeth closely spaced and not connected by concavity (Fig. 63B). ***Mesosoma.*** In lateral view, promesonotum short, angular, and moderately low, posterior mesonotum moderately steep, mesonotal process distinct, tubercle-like; promesonotal groove absent; metanotal groove absent; propodeal spines moderately short, with wide base and acute apex; humeral area laterally weakly produced (Fig. 20D). Surface shiny; lateral sides of pronotum, mesonotum, katepisternum, anepisternum, and propodeum rugofoveolate, pronotal dorsum with thick to thin, irregular to transverse rugae with smooth to indistinctly rugofoveolate interspaces; katepisternum with smooth notch. Pilosity moderately dense, long, and erect (Fig. 20D, F). ***Petiole.*** Shiny with fine foveolae; node smooth to finely foveolate, low, triangular, with rounded and thin apex, in rear view node dorsoventrally straight to slightly convex; pilosity moderately sparse and erect (Fig. 20D, F). ***Postpetiole.*** Shiny and foveolate; dorsum with reduced sculpture and smooth notch; in dorsal view oval, lateral margins medially with two dentate projections; pilosity long, moderately sparse, and erect (Fig. 20D, F). ***Gaster.*** Shiny and smooth; pilosity dense, long, and erect (Fig. 20D, F). ***Colour.*** Dark brown; legs and antennae yellow (Fig. 20D, F).

**Minor workers.** Measurements (*N* = 7): HL: 0.55–0.6 (0.56); HW: 0.46–0.51 (0.47); SL: 0.58–0.62 (0.6); EL: 0.09–0.11 (0.1); WL: 0.67–0.76 (0.7); PSL: 0.08–0.1 (0.09); MTL: 0.44–0.48 (0.46); PNW: 0.32–0.38 (0.33); PTW: 0.09–0.1 (0.1); PPW: 0.13–0.14 (0.14); CI: 116.0–123.7 (119.4); SI: 119.5–130.0 (126.6); PSLI: 14.4–17.8 (15.7); PPI: 64.7–72.5 (69.0); PNI: 67.9–73.9 (70.0); MTI: 93.6–101.7 (96.6).

***Head.*** Cephalic margin indistinctly concave or straight (Fig. 20A). Pilosity relatively sparse, long, decumbent to suberect. Sculpture shiny with sparse foveolae; frons with foveolae reduced medially with moderately thick, longitudinal, and interrupted rugae; area posterolateral from eyes with reduced sculpture, predominantly smooth; antennal sockets with few thick, curved outward rugae and indistinctly foveolate interspaces. Clypeus with median longitudinal carina absent; two lateral longitudinal carinae absent. Scape, when laid back, surpassing the posterior head margin by two-fifths of its length; pilosity dense, subdecumbent to erect (Fig. 20A, C). ***Mesosoma.*** In lateral view, promesonotum moderately high and short, arched; promesonotal groove absent; metanotal groove distinct; propodeal spines minute and triangular (Fig. 20C). Sculpture shiny; propodeum predominantly smooth, its dorsum with sparse, short, transverse rugae and indistinct foveolae; mesonotum, anepisternum, katepisternum, and propodeum predominantly smooth, with sparse and indistinct foveolae on anterolateral and anterior sides. Pilosity moderately dense, long, and erect (Fig. 20C, E). ***Gaster.*** With sparse, erect pilosity (Fig. 20C, E). ***Colour.*** Brown, legs slightly brighter (Fig. 20C, E).

##### Etymology.

From the type locality.

##### Biology.

The species was collected between 1294–1460 m in elevation, in rainforest, montane rainforest and park. Nests were located under rootmat on rock, in the soil, and in rotten logs.

##### Comments.

*Pheidole ambohimanga* sp. nov. is a member of the group of species characterised by major workers with head in full-face view oval and not widening posteriorly and medial part of frons with thick, moderately sparse, irregular rugae or medial frons with moderately dense, thin, longitudinal anteriorly to irregular posteriorly, interrupted rugae and very shallow occipital cleft. Minor workers of this group have short and moderately high promesonotum and dark body colouration ranging from orange to brown. The group consists of three species: *P. vadum* sp. nov., *P. analavelona* sp. nov., and *P. ambohimanga* sp. nov. *Pheidole ambohimanga* sp. nov. is sympatric with *P. vadum* sp. nov. but morphologically is most similar to *P. analavelona* sp. nov. It can be easily separated from *P. vadum* sp. nov. based on thicker and exclusively irregular rugae on frons, deeper occipital cleft in majors, and presence of additional rugae on head in minors. Majors of *P. ambohimanga* sp. nov. can be distinguished from *P. analavelona* sp. nov. based on smooth to very indistinctly rugoreticulate interspaces on head, while minor workers have head sculpture foveolae strongly reduced to absent on frons, predominantly smooth area posterolateral from eyes, and mesonotum, anepisternum, katepisternum, and propodeum predominantly smooth, with sparse and indistinct foveolae on anterolateral and anterior sides. Minors of *P. ambohimanga* sp. nov. can be easily separated from other members of the *sikorae* group based on short and moderately high promesonotum and additional, moderately thick to thick, longitudinal, and interrupted rugae on head.

#### Pheidole analavelona sp. nov.

Taxon classificationAnimalia

2DA61EF8-ABA4-5A0B-B374-D03E1E8A4F18

http://zoobank.org/DC4E9F6D-96E6-42F5-8076-02D067506117

**Figure 21.**
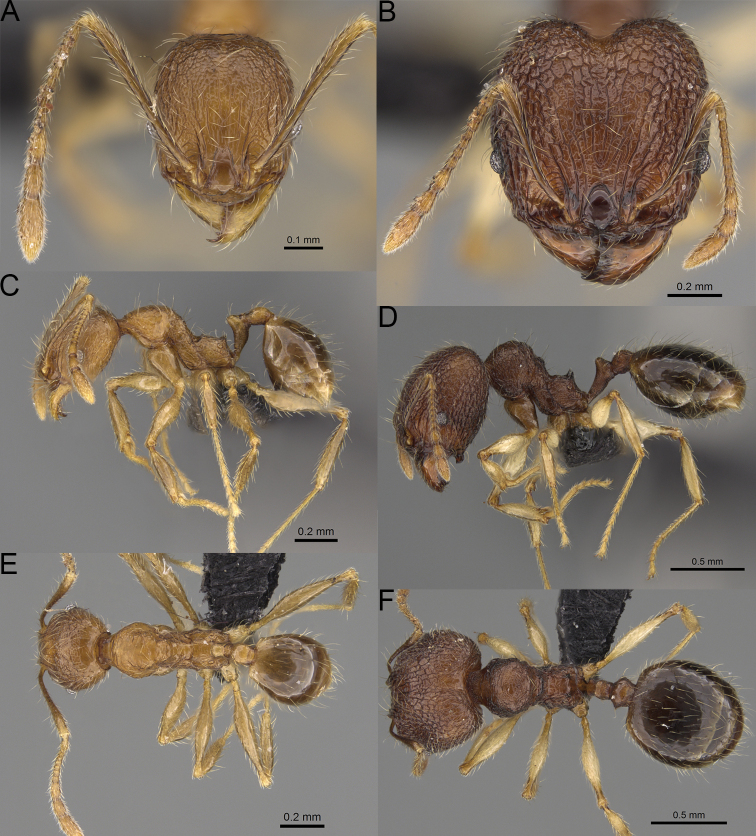
*Pheidole analavelona* sp. nov., full-face view (**A**), profile (**C**), and dorsal view (**E**) of paratype minor worker (CASENT0491846) and full-face view (**B**), profile (**D**), and dorsal view (**F**) of holotype major worker (CASENT0491849).

Figs 21A–F , 63C , 65C

##### Type material.

***Holotype.*** Madagascar. • 1 major worker; Toliara; Forêt Classée d’Analavelona, 33.2 km 344°NNW Mahaboboka; -22.64333, 44.17167; alt. 1300 m; 22 Feb 2003; Fisher et al. leg.; montane rainforest, ex rotten log; BLF07970; CASENT0491849, top specimen on the pin (CASC). ***Paratypes.*** • 9w., 6s., 2m.; same data as for holotype; CASENT0491847, CASENT0491846, CASENT0872248, CASENT0491850, CASENT0491845, CASENT0491844, CASENT0491848 (CASC, MHNG, PBZT).

##### Other material.

Madagascar. –**Toliara**: • 3w.; Forêt Classée d’Analavelona, 29.2 km 343°NNW Mahaboboka; -22.675, 44.19; alt. 1100 m; 18 Feb 2003; Fisher et al. leg.; montane rainforest, ex rotten log; BLF07833 (CASC). • 3w., 3s.; ibid.; BLF07835 (CASC). • 3w.; ibid.; BLF07844 (CASC). • 3w., 3s.; ibid.; BLF07857 (CASC). •3w.; ibid.; BLF07887 (CASC). • 3s.; ibid.; under stone; BLF07853 (CASC). • 9w., 3s.; Forêt Classée d’Analavelona, 33.2 km 344°NNW Mahaboboka; -22.64333, 44.17167; alt. 1300 m; 22 Feb 2003; Fisher et al. leg.; montane rainforest, ex rotten log; BLF07953 (CASC). • 1w., 1s.; ibid.; BLF07966 (CASC). • 3., 3s.; ibid.; BLF07971 (CASC). • 6w., 1s., 1q., 6m.; ibid.; BLF07989 (CASC).

##### Diagnosis.

Moderately large species. ***Major workers.*** Head in full-face view oval, not widening posteriorly, with anterior and posterior sides convex, in lateral view sub-oval, ventral and dorsal faces convex, occipital cleft moderately deep; sides of the head with dense, very long, suberect to erect pilosity; frons with thick, irregular rugae, moderately dense on lateral sides and sparser on medial part; interspaces shiny with distinct and sparse rugoreticulae; occipital lobes, area posterolateral from eyes never smooth; scape, when laid back, exceeding the midlength of head by one-fifth of its length; inner hypostomal teeth distinct, large, closely spaced, triangular, with rounded apex directed inward; outer hypostomal teeth lobe-like, wider than inner hypostomal teeth and approximately the same height, apex directed outward; inner and outer hypostomal teeth not closely spaced and not connected by concavity; mesosoma with dense rugofoveolae; pronotum, mesonotum, katepisternum, and anepisternum with additional thick to thin, irregular to transverse rugae; body dark brown. ***Minor workers.*** Head foveolate; medial frons with foveolae sparser and with moderately thick longitudinal and interrupted rugae; lateral sides of frons and vertex with additional moderately thick rugae; area posterolateral from eyes with reduced sculpture and smooth notches; scape, when laid back, surpassing the posterior head margin by two-fifths of its length; promesonotum moderately high and short; promesonotal groove absent; propodeal spines minute and triangular; propodeum predominantly smooth with indistinct rugofoveolae on lateral sides; mesonotum, anepisternum, katepisternum, and propodeum with sparse and indistinct rugofoveolae; body dark orange.

##### Description.

**Major workers.** Measurements (*N* = 10): HL: 0.97–1.02 (1.0); HW: 0.95–1.0 (0.98); SL: 0.64–0.67 (0.66); EL: 0.11–0.13 (0.12); WL: 0.88–0.95 (0.92); PSL: 0.13–0.15 (0.14); MTL: 0.57–0.6 (0.58); PNW: 0.44–0.48 (0.46); PTW: 0.13–0.17 (0.14); PPW: 0.26–0.29 (0.28); CI: 101.0–104.4 (102.0); SI: 65.3–69.3 (67.1); PSLI: 13.1–15.4 (14.1); PPI: 44.8–57.2 (52.2); PNI: 46.2–48.8 (47.4); MTI: 57.0–61.8 (59.3).

***Head.*** In full-face view oval, not widening posteriorly, with anterior and posterior sides convex (Fig. 21B). In lateral view sub-oval; ventral and dorsal faces convex; inner hypostomal teeth visible. Sides of the head with dense, very long, suberect to erect pilosity; whole head with dense, long, decumbent to erect pilosity. Frons with thick, irregular rugae, moderately dense on lateral sides and sparser on medial part; interspaces shiny with distinct and sparse rugoreticulae. Occipital lobes with relatively dense, thick, and irregular rugae; interspaces rugoreticulate. Gena with relatively dense, thick, and longitudinal rugae and rugoreticulate interspaces. Area posterolateral from eyes with moderately dense, thick, and irregular rugae; interspaces shiny with distinct and sparse rugoreticulae. Centre of clypeus smooth and shiny, lateral sides with indistinct rugulae; median notch present, moderately wide, and shallow; median longitudinal carina present; lateral longitudinal carinae absent. Scape, when laid back, exceeding the midlength of head by one-fifth of its length; pilosity subdecumbent to erect (Fig. 21B, D). Inner hypostomal teeth distinct, large, closely spaced, triangular, with rounded apex directed inward; outer hypostomal teeth lobe-like, wider than inner hypostomal teeth and approximately the same height, apex directed outward; inner and outer hypostomal teeth not closely spaced and not connected by concavity (Fig. 63C). ***Mesosoma.*** In lateral view, promesonotum short, angular, and moderately low, posterior mesonotum moderately steep, mesonotal process indistinct, tubercle-like; promesonotal groove absent; metanotal groove indistinct; propodeal spines moderately short, with wide base and acute apex; humeral area laterally weakly produced (Fig. 21D). Surface shiny with dense rugofoveolae; pronotum, mesonotum, katepisternum, and anepisternum with additional thick to thin, irregular to transverse rugae. Pilosity moderately dense, long, and erect (Fig. 21D, F). ***Petiole.*** Shiny with fine foveolae; node smooth to finely foveolate, low, triangular, with rounded and thin apex, in rear view node dorsoventrally straight to slightly convex; pilosity moderately sparse and erect (Fig. 21D, F). ***Postpetiole.*** Shiny and foveolate; dorsum with reduced sculpture and smooth notch; in dorsal view oval, lateral margins medially with two dentate projections; pilosity long, moderately sparse and erect (Fig. 21D, F). ***Gaster.*** Shiny and smooth; pilosity dense, long, and erect (Fig. 21D, F). ***Colour.*** Dark brown; legs and antennae yellow (Fig. 21D, F).

**Minor workers.** Measurements (*N* = 10): HL: 0.55–0.59 (0.57); HW: 0.46–0.48 (0.47); SL: 0.59–0.63 (0.62); EL: 0.09–0.11 (0.1); WL: 0.69–0.73 (0.71); PSL: 0.06–0.09 (0.08); MTL: 0.46–0.49 (0.47); PNW: 0.32–0.35 (0.33); PTW: 0.07–0.1 (0.08); PPW: 0.12–0.14 (0.13); CI: 116.6–124.7 (120.7); SI: 125.9–133.5 (130.5); PSLI: 10.6–15.1 (14.0); PPI: 55.0–81.5 (66.0); PNI: 67.4–72.0 (69.8); MTI: 97.3–102.3 (99.4).

***Head.*** Cephalic margin indistinctly concave or straight (Fig. 21A). Pilosity relatively dense, long, decumbent to suberect. Sculpture foveolate; medial frons with foveolae sparser and with moderately thick, longitudinal, and interrupted rugae; lateral sides of frons and vertex with additional moderately thick rugae; area posterolateral from eyes with reduced sculpture and smooth notches; antennal sockets with few thick, curved outward rugae and foveolate interspaces. Clypeus with median longitudinal carina absent; two lateral longitudinal carinae absent. Scape, when laid back, surpassing the posterior head margin by two-fifths of its length; pilosity dense, subdecumbent to erect (Fig. 21A, C). ***Mesosoma.*** In lateral view, promesonotum moderately high and short, arched; promesonotal groove absent; metanotal groove distinct; propodeal spines minute, triangular (Fig. 21C). Sculpture shiny; propodeum predominantly smooth with indistinct rugofoveolae on lateral sides; mesonotum, anepisternum, katepisternum, and propodeum with sparse and indistinct rugofoveolae. Pilosity moderately dense, long, and erect (Fig. 21C, E). ***Gaster.*** With sparse, erect pilosity (Fig. 21C, E). ***Colour.*** Dark orange, gaster slightly darker (Fig. 21C, E).

##### Etymology.

From the type locality.

##### Biology.

The species was collected between 1100–1300 m in elevation, in montane rainforest. Nests were located in rotten logs and under stones.

##### Comments.

*Pheidole analavelona* sp. nov. is a member of the group of species characterised by major workers with head in full-face view oval and not widening posteriorly and medial part of frons with thick, moderately sparse, and irregular rugae or medial frons with moderately dense, thin, longitudinal anteriorly to irregular posteriorly, and interrupted rugae and very shallow occipital cleft. Minor workers of this group have short and moderately high promesonotum and dark body colouration ranging from orange to brown. The group consists of three species: *P. vadum* sp. nov., *P. analavelona* sp. nov., and *P. ambohimanga* sp. nov. *Pheidole analavelona* sp. nov. is known from Forêt Classée d’Analavelona in Toliara and its distribution does not overlap with that of the two remaining members of the group known from the vicinity of Antananarivo. Morphologically both minor and major workers of *P. analavelona* sp. nov. are most similar to *P. ambohimanga* sp. nov. Majors of *P. analavelona* sp. nov. can be distinguished based on distinctly rugoreticulate interspaces on head while minor workers have head sculpture foveolate with foveolae sparser on frons and area posterolateral from eyes, and sparse but never absent rugofoveolae on mesonotum, anepisternum, katepisternum, and propodeum. Minors of *P. analavelona* sp. nov. can be easily separated from other members of the *sikorae* group based on short and moderately high promesonotum and additional, moderately thick to thick, longitudinal, and interrupted rugae on head.

#### Pheidole andohahela sp. nov.

Taxon classificationAnimalia

6CB24270-55C6-516F-9808-25736C3921CC

http://zoobank.org/AD047203-0321-4E69-B9BB-EF246CA61BFC

**Figure 22.**
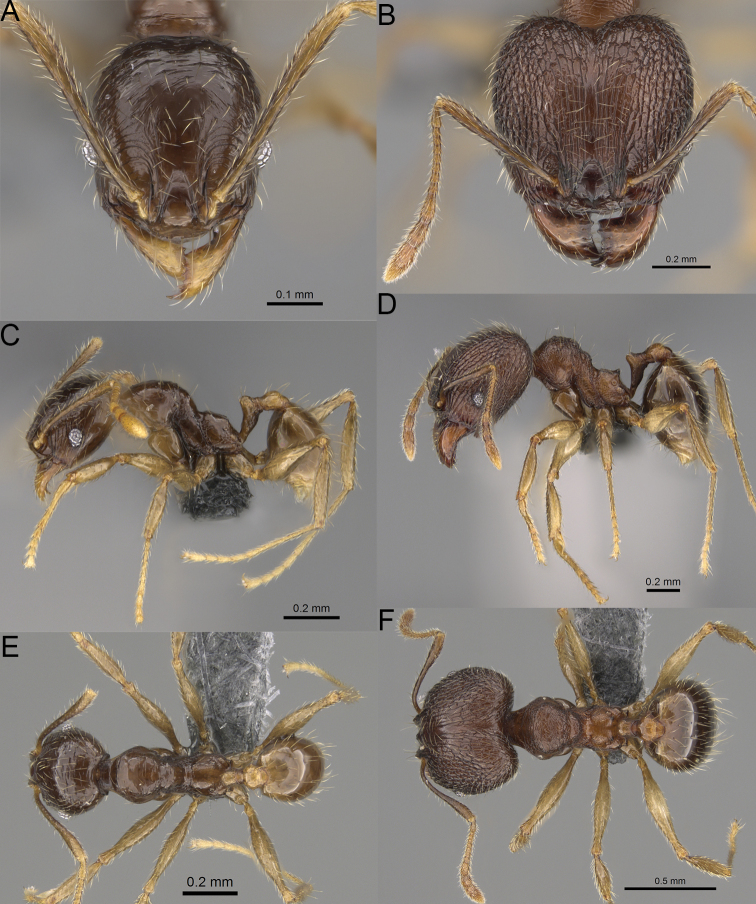
*Pheidole andohahela* sp. nov., full-face view (**A**), profile (**C**), and dorsal view (**E**) of paratype minor worker (CASENT0923281) and full-face view (**B**), profile (**D**), and dorsal view (**F**) of holotype major worker (CASENT0700917).

Figs 22A–F , 63D , 65D

##### Type material.

***Holotype.*** Madagascar. • 1 major worker; Toliara; Anosy Region, Parc National Andohahela, Col de Tanatana; -24.74969, 46.84949; alt. 400 m; 9 Mar 2015; B. L. Fisher et al. leg.; rainforest, ex rotten log; BLF36798; CASENT0700917 (CASC). ***Paratype.*** • 1w.; same data as for holotype, CASENT0923281 (CASC).

##### Other material.

Madagascar. –**Toliara**: • 1w., 1s.; Anosy Region, Parc National Andohahela, Col de Tanatana; -24.74969, 46.84949; alt. 400 m; 9 Mar 2015; B. L. Fisher et al. leg.; rainforest, ex rotten log; BLF36803 (CASC).

##### Diagnosis.

Minute species. ***Major workers.***HL < 0.95 mm and WL < 0.8 mm; head in full-face view sub-oval; body dark brown; sides of head with very dense, relatively short, suberect pilosity; entire head distinctly sculptured; scape, when laid back, exceeding the midlength of head by two-fifths of its length; inner hypostomal teeth distinct, large, with sharp apex; outer hypostomal teeth lobe-like with apex directed distinctly outward; inner and outer hypostomal teeth not closely spaced and not connected by concavity. ***Minor workers.***HL < 0.5 mm and WL < 0.6 mm, scape, when laid back, surpassing the posterior head margin by two-fifths of its length, propodeal spines reduced to small lobes, head elongate and oval and body dark brown; head sculpture shiny and predominantly smooth, vertex and frons with sparse, short, and transverse rugulae; anterolateral sides of propodeum and anepisternum with indistinct and sparse rugoreticulae.

##### Description.

**Major workers.** Measurements (*N* = 2): HL: 0.87, 0.92; HW: 0.87, 0.92; SL: 0.64, 0.63; EL: 0.12, 0.11; WL: 0.77, 0.79; PSL: 0.1, 0.11; MTL: 0.53, 0.56; PNW: 0.36, 0.39; PTW: 0.11, 0.12; PPW: 0.28, 0.29; CI: 100.0, 99.9; SI: 72.9, 67.8; PSLI: 11.9, 12.4; PPI: 40.1, 40.7; PNI: 41.3, 42.0; MTI: 60.8, 60.5.

***Head.*** In full-face view sub-oval, slightly widening posteriorly, with anterior and posterior sides convex (Fig. 22B). In lateral view sub-oval; ventral and dorsal faces convex; inner hypostomal teeth visible. Sides of the head with very dense, relatively short, suberect pilosity; whole head with dense, long, decumbent to erect pilosity. Medial part of frons with thick, dense, longitudinal, and interrupted rugae, interspaces smooth; lateral sides with thick, dense, and irregular rugae, interspaces shiny with dense and distinct rugoreticulae. Occipital lobes with thick, irregular rugae and smooth to rugoreticulate interspaces. Area posterolateral from eyes rugoreticulate to rugofoveolate with additional longitudinal and thick rugae. Gena with relatively dense, thick, and longitudinal rugae and rugoreticulate interspaces. Centre of clypeus smooth and shiny, lateral sides with indistinct rugulae; median notch present, moderately wide, and shallow; median longitudinal carina present; lateral longitudinal carinae absent. Scape, when laid back, exceeding the midlength of head by two-fifths of its length; pilosity subdecumbent to erect (Fig. B, D). Inner hypostomal teeth distinct, large, closely spaced, triangular, with sharp apex directed upward; outer hypostomal teeth lobe-like, more narrow and lower than inner hypostomal teeth, apex directed outward; inner and outer hypostomal teeth not closely spaced and not connected by concavity (Fig. 63D). ***Mesosoma.*** In lateral view, promesonotum short, angular, and moderately low, posterior mesonotum moderately steep, mesonotal process indistinct, tubercle-like; promesonotal groove absent; metanotal groove absent; propodeal spines short, with moderately wide base and acute apex; humeral area laterally weakly produced (Fig. 22D). Surface shiny with fine rugofoveolae; anterolateral sides of pronotum with smooth notches. Pilosity moderately dense, long, and erect (Fig. 22D, F). ***Petiole.*** Shiny with fine foveolae;node smooth to finely foveolate, low, triangular, with rounded and thin apex, in rear view node dorsoventrally straight to slightly convex; pilosity moderately sparse and erect (Fig. 22D, F). ***Postpetiole.*** Shiny and foveolate; dorsum with reduced sculpture and smooth notch; in dorsal view oval, lateral margins medially with two dentate projections; pilosity long, moderately sparse, and erect (Fig. 22D, F). ***Gaster.*** Shiny and smooth; pilosity dense, long, and erect (Fig. D, F). ***Colour.*** Dark brown; legs, gaster and antennae yellowish brown (Fig. 22D, F).

**Minor workers.** Measurements (*N* = 2): HL: 0.48, 0.49; HW: 0.39, 0.43; SL: 0.55, 0.57; EL: 0.09, 0.09; WL: 0.58, 0.58; PSL: 0.05, 0.05; MTL: 0.4, 0.42; PNW: 0.28, 0.28; PTW: 0.07, 0.08; PPW: 0.12, 0.14; CI: 122.4, 115.0; SI: 140.3, 132.8; PSLI: 10.8, 9.4; PPI: 55.6, 55.6; PNI: 70.9, 66.0; MTI: 102.0, 98.4.

***Head.*** Cephalic margin indistinctly convex or straight (Fig. 22A). Pilosity relatively dense, long, decumbent to suberect. Sculpture shiny and smooth; vertex and frons with sparse, short, and transverse rugulae; antennal sockets with few thick, curved outward rugae and smooth interspaces. Clypeus with median longitudinal carina absent; two lateral longitudinal carinae absent. Scape, when laid back, surpassing the posterior head margin by two-fifths of its length; pilosity dense, subdecumbent to erect (Fig. 22A, C). ***Mesosoma.*** In lateral view, promesonotum moderately high and short, arched; promesonotal groove absent; metanotal groove distinct; propodeal spines reduce to small tubercles (Fig. 22C). Sculpture shiny and smooth; anterolateral sides of propodeum and anepisternum with indistinct and sparse rugoreticulae. Pilosity moderately sparse, long, and erect (Fig. 22C, E). ***Gaster.*** With sparse, erect pilosity (Fig. 22C, E). ***Colour.*** Dark brown, legs and antenna yellowish brown (Fig. 22C, E).

##### Etymology.

From the type locality.

##### Biology.

The species was collected between 400–775 m in elevation, in rainforest. Nests were located in rotten logs and rotten sticks on the ground.

##### Comments.

*Pheidole andohahela* sp. nov. is known only from its terra typica: Col de Tanatana in Parc National Andohahela, Toliara and is most similar to parapatric *P. lavasoa* sp. nov. recorded so far only from Grand Lavasoa, Toliara. Major workers of both taxa are extremely similar and can be distinguished based on slight differences in the setosity of lateral sides of head (which is sparser and longer in *P. andohahela* sp. nov.) and shape of the hypostomal teeth (*P. andohahela* sp. nov. has inner hypostomal teeth with sharp apices and outer hypostomal teeth that are lobe-like and distinctly directed outward). Minor workers are a better resource for species separation. Minor workers of *P. andohahela* sp. nov. have transverse rugulae on vertex and frons, while anterolateral sides of their propodeum, and anepisternum have indistinct and sparse rugoreticulae; body colour is dark brown.

#### Pheidole anomala sp. nov.

Taxon classificationAnimalia

475FAEB4-E99A-5A22-A5DF-46D3D21DE39B

http://zoobank.org/7E2C359F-AA66-43D9-A591-8D5B8D2C788F

**Figure 23.**
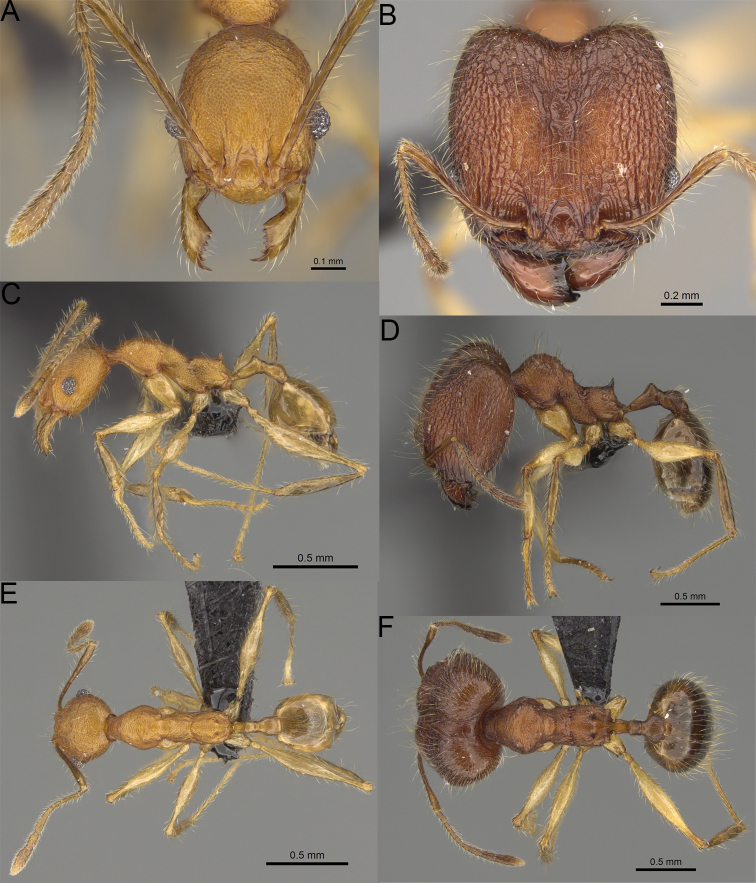
*Pheidole anomala* sp. nov., full-face view (**A**), profile (**C**), and dorsal view (**E**) of paratype minor worker (CASENT0235124) and full-face view (**B**), profile (**D**), and dorsal view (**F**) of holotype major worker (CASENT0134498).

Figs 23A–F , 63E , 65E

##### Type material.

***Holotype.*** Madagascar. • 1 major worker; Antsiranana; Parc National Montagne d’Ambre, Mahasarika; -12.53176, 49.17662; alt. 1135 m; 17 Nov 2007; B. L. Fisher et al. leg.; montane rainforest, ex rotten log; BLF18461; CASENT0134498 (CASC). ***Paratype.*** • 1w.; same data as for holotype, CASENT0235124 (CASC).

##### Diagnosis.

Moderately large species. ***Major workers.*** Head in full-face view sub-oval and slightly widening posteriorly, with anterior and posterior sides slightly convex, in lateral view sub-oval; ventral and dorsal faces convex; sides of the head with dense, long, suberect to erect pilosity; medial part of frons with irregular and dense rugae, interspaces distinctly rugulate; occipital lobes, and area posterolateral from eyes without smooth notches; scape, when laid back, exceeding the midlength of head by one-fifth of its length; inner hypostomal teeth distinct, large, closely spaced, triangular, with rounded apex directed inward; outer hypostomal teeth lobe-like, wider than and approximately as high as inner teeth; inner and outer hypostomal teeth closely spaced and not connected by concavity; mesosoma rugofoveolate; gaster smooth; body ferruginous. ***Minor workers.*** Head foveolate; vertex with a few arcing, sparse, and moderately thick rugae; scape, when laid back, exceeding the posterior head margin by one-third of its length; promesonotum very low and moderately long; promesonotal groove absent; propodeal spines small, triangular; mesosoma foveolate; body yellow.

##### Description.

**Major workers.** Measurements (*N* = 1): HL: 1.42; HW: 1.37; SL: 0.87; EL: 0.18; WL: 1.25; PSL: 0.21; MTL: 0.87; PNW: 0.57; PTW: 0.16; PPW: 0.34; CI: 103.0; SI: 63.2; PSLI: 14.8; PPI: 47.8; PNI: 41.6; MTI: 63.2.

***Head.*** In full-face sub-oval, widening posteriorly, with anterior and posterior sides convex (Fig. 23B). In lateral view sub-oval; ventral and dorsal faces convex; inner hypostomal teeth visible. Sides of the head with dense, long, suberect to erect pilosity; whole head with dense, long, decumbent to erect pilosity. Frons with thick, irregular, and dense rugae, interspaces distinctly rugulate. Occipital lobes with slightly thinner, dense, and irregular rugae and rugulate interspaces. Area posterolateral from eyes with dense rugoreticulae. Gena with relatively sparse and thick, longitudinal rugae with distinctly rugofoveolate interspaces. Centre of clypeus with sparse foveolae and shiny, lateral sides with indistinct rugulae; median notch present, moderately wide, and shallow; median longitudinal carina present; lateral longitudinal carinae absent. Scape, when laid back, exceeding the midlength of head by one-fifth of its length; pilosity subdecumbent to erect (Fig. 23B, D). Inner hypostomal teeth distinct, large, closely spaced, triangular, with rounded apex directed inward; outer hypostomal teeth lobe-like, wider than and approximately as high as inner teeth; inner and outer hypostomal teeth closely spaced and not connected by concavity (Fig. 63E). ***Mesosoma.*** In lateral view, promesonotum short, angular, and moderately low, posterior mesonotum moderately steep, mesonotal process indistinct, tubercle-like; promesonotal groove absent; metanotal groove absent; propodeal spines moderate, with wide base and acute apex; humeral area produced (Fig. 23D). Surface shiny and rugofoveolate. Pilosity moderately dense, long, and erect (Fig. 23D, F). ***Petiole.*** Shiny with dense foveolae; node finely foveolate, triangular, with rounded and thick apex, in rear view node dorsoventrally slightly convex; pilosity moderately sparse and erect (Fig. 23D, F). ***Postpetiole.*** Shiny and foveolate; dorsum with reduced sculpture and smooth notch; in dorsal view oval, lateral margins medially with two dentate projections; pilosity long, moderately sparse, and erect (Fig. 23D, F). ***Gaster.*** Shiny and smooth; pilosity moderately dense, long, and erect (Fig. D, F). ***Colour.*** Ferruginous with yellowish legs (Fig. 23D, F).

**Minor workers.** Measurements (*N* = 1): HL: 0.66; HW: 0.52; SL: 0.82; EL: 0.15; WL: 0.89; PSL: 0.12; MTL: 0.7; PNW: 0.36; PTW: 0.1; PPW: 0.16; CI: 128.2; SI: 158.8; PSLI: 17.4; PPI: 62.7; PNI: 69.5; MTI: 136.7.

***Head.*** Cephalic margin slightly convex (Fig. 23A). Pilosity relatively sparse, moderately long, subdecumbent to erect. Sculpture shiny and foveolate; vertex with a few arcing, sparse, and moderately thick rugae; antennal sockets with few indistinct, curved outward rugae and foveolate interspaces. Clypeus with median longitudinal carina absent; two lateral longitudinal carinae absent. Scape, when laid back, exceeding the posterior head margin by one-third of its length; pilosity dense, subdecumbent to erect (Fig. 23A, C). ***Mesosoma.*** In lateral view, promesonotum very low and moderately long, arched; promesonotal groove absent; metanotal groove distinct; propodeal spines small, triangular (Fig. 23C). Sculpture shiny and foveolate. Pilosity very sparse, moderately long, and erect (Fig. 23C, E). ***Gaster.*** With sparse, erect pilosity (Fig. 23C, E). ***Colour.*** Yellow, legs brighter (Fig. 23C, E).

##### Etymology.

Latin for irregular in reference to strongly irregular rugae on frons of major workers.

##### Biology.

The species was collected at 1135 m in elevation, in montane rainforest. Nest was located in a rotten log.

##### Comments.

*Pheidole anomala* sp. nov. is most similar to *P. veteratrix* Forel, a widespread species known from Antananarivo north to Taolagnaro, and sympatric *P. trichotos* sp. nov. Major workers of *P. anomala* sp. nov. differ from both taxa in ferruginous body colouration and distinctly rugulate interspaces on frons; minor workers are easy to distinguish based on their bright yellow body colouration and entirely foveolate katepisternum.

#### Pheidole anosyenne sp. nov.

Taxon classificationAnimalia

DDD18706-E1D5-5A18-9D72-CE989F30B09F

http://zoobank.org/F6512E6B-6D15-48AB-A3E7-05878764E0EF

**Figure 24.**
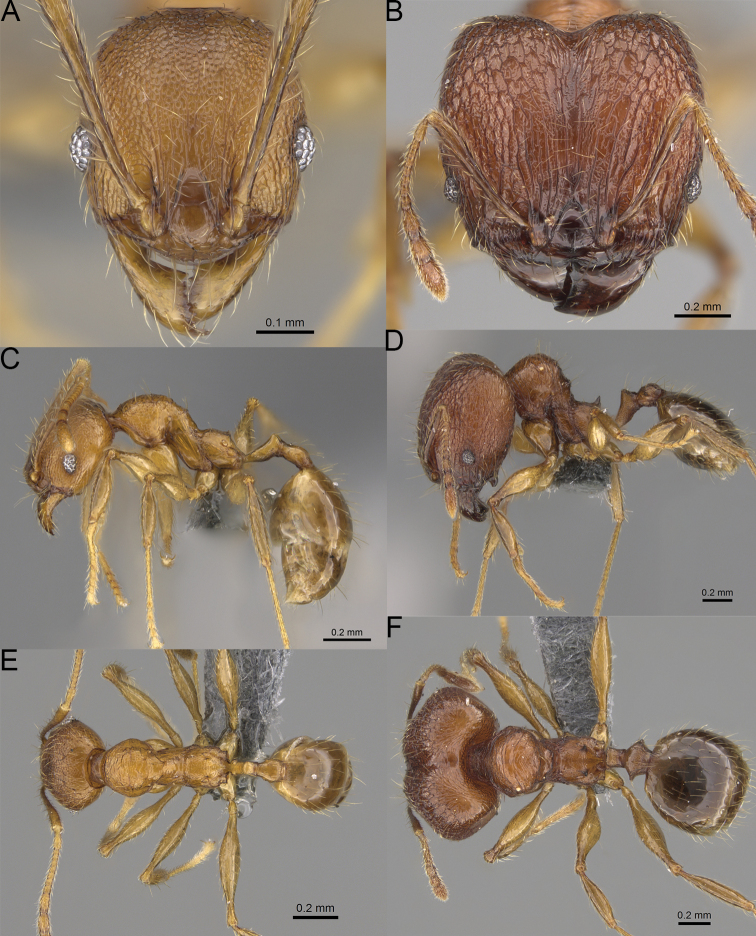
*Pheidole anosyenne* sp. nov., full-face view (**A**), profile (**C**), and dorsal view (**E**) of paratype minor worker (CASENT0704255) and full-face view (**B**), profile (**D**), and dorsal view (**F**) of holotype major worker (CASENT0923297).

Figs 24A–F , 63F , 65F

##### Type material.

***Holotype.*** Madagascar. • 1 major worker; Toliara; Anosy Region, Anosyenne Mts, 31.2 km NW Manantenina; -24.13894, 47.06804; 1125 m; 26 Feb 2015; B. L. Fisher et al. leg.; rainforest, ex rotten log; BLF36544; CASENT0923297 (CASC). ***Paratype.*** • 1w.; same data as for holotype; CASENT0704255 (CASC).

##### Diagnosis.

Moderately large species. ***Major workers.*** Head in full-face view sub-oval and slightly widening posteriorly, with anterior and posterior sides slightly convex, in lateral view sub-oval; ventral and dorsal faces convex; sides of the head with moderately dense, short, decumbent to subdecumbent pilosity; anteromedial part of frons with moderately dense and thick, longitudinal rugae, posteromedial part with irregular rugae, interspaces with sparse and distinct rugofoveolae; occipital lobes, and area posterolateral from eyes without smooth notches; scape, when laid back, exceeding the midlength of head by approximately one-fifth of its length; inner hypostomal teeth distinct, moderate, closely spaced, triangular, with rounded apex and relatively narrow base; outer hypostomal teeth lobe-like, wider, and higher than inner hypostomal teeth, apex directed forward; inner and outer hypostomal teeth closely spaced and connected by indistinct concavity; mesosoma with fine rugofoveolae, dorsal and lateral sides of pronotum with additional transverse and thin rugae; gaster smooth; body ochreous. ***Minor workers.*** Head foveolate with additional indistinct longitudinal rugulae on medial frons, area posterolateral from eyes with reduced sculpture; scape, when laid back, surpassing the posterior head margin by two-fifths of its length; promesonotum moderately high and long, arched; promesonotal groove present; propodeal spines very small, triangular; mesosomal dorsum rugofoveolate; lateral sides of pronotum foveolate, anepisternum and katepisternum smooth, lateral sides of propodeum with smooth notches; body dark orange.

##### Description.

**Major workers.** Measurements (*N* = 1): HL: 1.13; HW: 1.18; SL: 0.69; EL: 0.14; WL: 1.0; PSL: 0.17; MTL: 0.63; PNW: 0.52; PTW: 0.14; PPW: 0.34; CI: 96.0; SI: 58.3; PSLI: 14.8; PPI: 40.2; PNI: 44.0; MTI: 53.2.

***Head.*** In full-face view sub-oval, widening posteriorly, with anterior and posterior sides convex (Fig. 24B). In lateral view sub-oval; ventral and dorsal faces convex; inner hypostomal teeth visible. Sides of the head with moderately dense, short, decumbent to subdecumbent pilosity; whole head with relatively dense, short, decumbent to erect pilosity. Anteromedial part of frons with moderately dense and thick, longitudinal rugae, posteromedial part with irregular rugae, interspaces with sparse and distinct rugofoveolae; lateral sides with irregular and thick rugae with distinctly rugofoveolate interspaces. Occipital lobes with thick, sparse, irregular rugae, interspaces smooth or with fine, indistinct rugulae. Gena with sparse and thick, longitudinal rugae and indistinctly rugofoveolate interspaces. Area posterolateral from eyes with dense and thin rugofoveolae. Centre of clypeus smooth and shiny, lateral sides with indistinct rugulae; median notch present, wide, and shallow; median longitudinal carina present; lateral longitudinal carinae absent. Scape, when laid back, exceeding the midlength of head by approximately one-fifth of its length; pilosity decumbent to suberect (Fig. 24B, D). Inner hypostomal teeth distinct, moderate, closely spaced, triangular, with rounded apex and relatively narrow base; outer hypostomal teeth lobe-like, wider and higher than inner hypostomal teeth, apex directed forward; inner and outer hypostomal teeth closely spaced and connected by indistinct concavity (Fig. 63F). ***Mesosoma.*** In lateral view, promesonotum short, angular, and moderately high, posterior mesonotum moderately steep, mesonotal process indistinct, tubercle-like; promesonotal groove absent; metanotal groove absent; propodeal spines moderate, relatively wide, with acute apex; humeral area laterally weakly produced (Fig. 24D). Surface shiny with fine rugofoveolae, dorsal and lateral sides of pronotum with additional transverse and thin rugae. Pilosity relatively dense, long, and erect (Fig. 24D, F). ***Petiole.*** Shiny with fine and dense foveolae; node foveolate, low, triangular, with rounded and thin apex, in rear view node dorsoventrally convex; pilosity moderately sparse and erect (Fig. 24D, F). ***Postpetiole.*** Shiny, with fine and sparse rugofoveolae; in dorsal view postpetiole oval, lateral margins medially with two dentate projections; pilosity long, moderately sparse, and erect (Fig. 24D, F). ***Gaster.*** Shiny and smooth; pilosity moderately dense, long, and erect (Fig. 24D, F). ***Colour.*** Ochreous; legs dark yellow, gaster and mandibles brown (Fig. 24D, F).

**Minor workers.** Measurements (*N* = 1): HL: 0.55; HW: 0.49; SL: 0.62; EL: 0.11; WL: 0.72; PSL: 0.07; MTL: 0.47; PNW: 0.34; PTW: 0.07; PPW: 0.13; CI: 113.8; SI: 126.7; PSLI: 12.8; PPI: 54.3; PNI: 70.4; MTI: 96.5.

***Head.*** Cephalic margin indistinctly convex (Fig. 24A). Pilosity relatively sparse, long, decumbent to subdecumbent. Head foveolate with additional indistinct longitudinal rugulae on medial frons, area posterolateral from eyes with reduced sculpture; antennal sockets with few thick, curved outward rugae and foveolate interspaces. Clypeus with median longitudinal carina absent; two lateral longitudinal carinae absent. Scape, when laid back, surpassing the posterior head margin by two-fifths of its length; pilosity dense, subdecumbent to erect (Fig. 24A, C). ***Mesosoma.*** In lateral view, promesonotum moderately high and long, arched; promesonotal groove present but indistinct; metanotal groove present and distinct; propodeal spines very small, triangular (Fig. 24C). Mesosomal dorsum rugofoveolate; lateral sides of pronotum foveolate, anepisternum and katepisternum smooth, lateral sides of propodeum with smooth notches. Pilosity sparse, moderately long, and erect (Fig. 24C, E). ***Petiole.*** Peduncle with ventral face relatively straight (Fig. 24C, E). ***Gaster.*** With sparse, erect pilosity (Fig. 24C, E). ***Colour.*** Dark orange (Fig. 24C, E).

##### Etymology.

From the type locality.

##### Biology.

The species was collected at 1125 m in elevation, in rainforest. Nest was located in rotten log.

##### Comments.

*Pheidole anosyenne* sp. nov., described from Anosyenne Mts. in Toliara, has major workers with dense and thick rugae that are anteromedially longitudinal and posteromedially irregular with sparsely and distinctly rugofoveolate interspaces, and ferruginous body colouration. Most similar are *P. joffreville* sp. nov. and *P. mivory* sp. nov., known from remote localities in Antsiranana. Major workers of *P. anosyenne* sp. nov. can be easily separated from both of those taxa based on sparse to moderately sparse, moderately long to long, decumbent to erect pilosity on sides of head, brighter body colouration and presence of smooth notches on lateral sides of pronotum and katepisternum; minor workers differ from *P. joffreville* sp. nov. in brighter body colouration, presence of smooth notches on lateral sides of propodeum, and entirely smooth anepisternum and katepisternum, from *P. mivory* sp. nov. in foveolate mesosomal dorsum, brighter body colouration, and less developed propodeal spines.

#### Pheidole antranohofa sp. nov.

Taxon classificationAnimalia

EE46802C-2951-5364-BE01-53EA8BC41EEE

http://zoobank.org/269DB81E-05EC-47B5-9DCC-F99B6E4E5007

**Figure 25.**
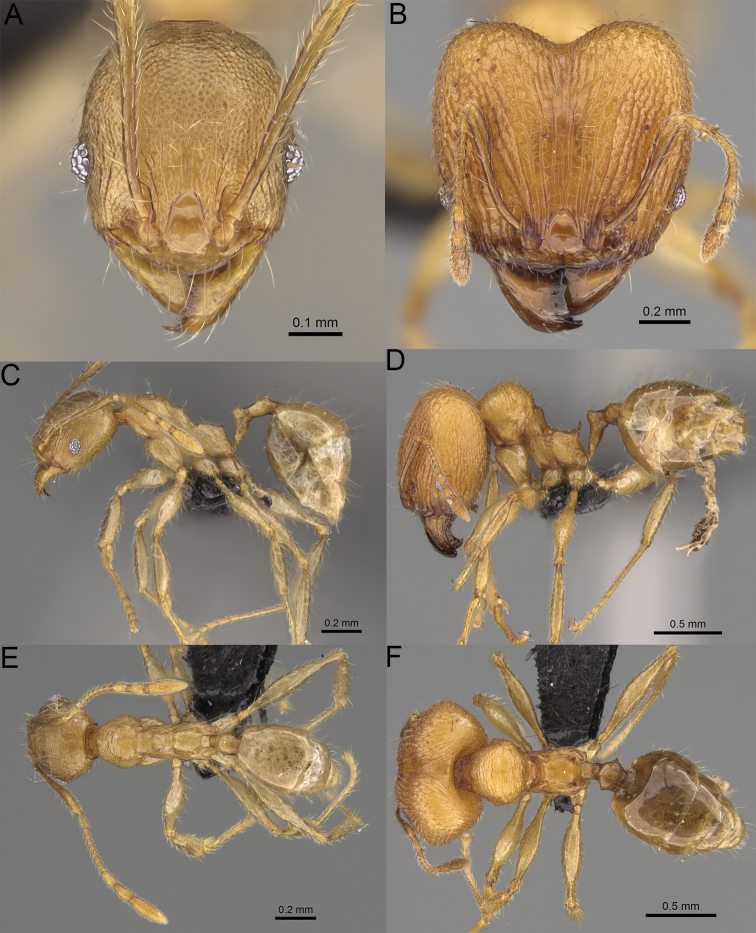
*Pheidole antranohofa* sp. nov., full-face view (**A**), profile (**C**), and dorsal view (**E**) of paratype minor worker (CASENT0923294) and full-face view (**B**), profile (**D**), and dorsal view (**F**) of holotype major worker (CASENT0923295).

Figs 25A–F , 63G , 65G

##### Type material.

***Holotype.*** Madagascar. • 1 major worker; Antsiranana; Parc National de Marojejy, Antranohofa, 26.6 km 31°NNE Andapa, 10.7 km 318°NW Manantenina; -14.44333, 49.74333; alt. 1325 m; 20 Nov 2003; B. L. Fisher et al. leg.; montane rainforest, ex root mat, ground layer; BLF09196; CASENT0923295, top specimen on the pin (CASC). ***Paratypes.*** • 3w., 2s; same data as for holotype; CASENT0923294, CASENT0499827CASENT0499829 (CASC, MHNG, PBZT).

##### Other material.

Madagascar. –**Antsiranana**: •1w., 1q.; Parc National de Marojejy, Antranohofa, 26.6 km 31°NNE Andapa, 10.7 km 318°NW Manantenina; -14.44333, 49.74333; alt. 1325 m; 20 Nov 2003; B. L. Fisher et al. leg.; montane rainforest, ex rotten tree stump; BLF09202 (CASC). •1w., 1s.; Parc National de Marojejy, Antranohofa, 26.6 km 31°NNE Andapa, 10.7 km 318°NW Manantenina; -14.44333, 49.74333; alt. 1325 m; 20 Nov 2003; B. L. Fisher et al. leg.; montane rainforest, ex rotten log; BLF09215 (CASC).

##### Diagnosis.

Moderately large species. ***Major workers.*** Head in full-face view sub-oval, widening posteriorly, with anterior and posterior sides convex, in lateral view sub-oval; ventral and dorsal faces convex; sides of the head with dense, short, suberect pilosity; anterior and medial parts of frons with moderately dense and thick, longitudinal and sometimes interrupted rugae with distinctly rugofoveolate interspaces; posterolateral sides with longitudinally irregular and thick rugae with distinctly rugofoveolate interspaces, area posterolateral from eyes without smooth notches; scape, when laid back, exceeding the midlength of head by approximately one-fifth of its length; inner hypostomal teeth distinct, moderately long, closely spaced, triangular, with rounded apex and wide base; outer hypostomal teeth lobe-like, wider and higher than inner hypostomal teeth, apex directed outward; inner and outer hypostomal teeth closely spaced and connected by indistinct concavity; promesonotum predominantly rugofoveolate, with additional sparse, thin, transverse rugae on pronotum; propodeum with fine rugofoveolae; gaster smooth; body yellowish orange. ***Minor workers.*** Head foveolate with additional indistinct rugulae on frons and vertex, area posterolateral from eyes with reduced sculpture, on posterior part predominantly smooth; scape, when laid back, surpassing the posterior head margin by two-fifths of its length; promesonotum moderately high and short; promesonotal groove present; propodeal spines indistinct, triangular; mesosoma smooth and shiny, only dorsum with few transverse, indistinct rugulae and propodeum with indistinct and sparse foveolae; body dark yellow.

##### Description.

**Major workers.** Measurements (*N* = 4): HL: 1.18–1.23 (1.21); HW: 1.21–1.27 (1.24); SL: 0.67–0.7 (0.69); EL: 0.14–0.16 (0.15); WL: 1.1–1.13 (1.12); PSL: 0.2–0.21 (0.2); MTL: 0.7–0.73 (0.72); PNW: 0.54–0.59 (0.57); PTW: 0.15–0.17 (0.16); PPW: 0.33–0.35 (0.34); CI: 96.7–97.8 (97.3); SI: 54.1–57.8 (55.4); PSLI: 16.3–17.7 (16.9); PPI: 43.4–50.5 (46.6); PNI: 43.9–46.9 (45.6); MTI: 56.7–58.8 (57.6).

***Head.*** In full-face view sub-oval, widening posteriorly, with anterior and posterior sides convex (Fig. 25B). In lateral view sub-oval; ventral and dorsal faces convex; inner hypostomal teeth invisible. Sides of the head with dense, short, suberect pilosity; whole head with relatively dense, short, decumbent to erect pilosity. Anterior and medial parts of frons with moderately dense and thick, longitudinal and sometimes interrupted rugae with distinctly rugofoveolate interspaces; posterolateral sides with longitudinally irregular and thick rugae with distinctly rugofoveolate interspaces. Occipital lobes with thick, sparse, irregular rugae, interspaces smooth or with fine, indistinct rugofoveolae. Gena with sparse and thick, longitudinal rugae and smooth interspaces. Area posterolateral from eyes with thin, longitudinal rugae with distinctly rugofoveolate interspaces. Centre of clypeus smooth and shiny, lateral sides with indistinct rugulae; median notch present, wide and shallow; median longitudinal carina absent; lateral longitudinal carinae absent. Scape, when laid back, exceeding the midlength of head by approximately one-fifth of its length; pilosity decumbent to suberect (Fig. 25B, D). Inner hypostomal teeth distinct, moderate, closely spaced, triangular, with rounded apex and wide base; outer hypostomal teeth lobe-like, wider and higher than inner hypostomal teeth, apex directed outward; inner and outer hypostomal teeth closely spaced and connected by indistinct concavity (Fig. 63G). ***Mesosoma.*** In lateral view, promesonotum short, angular, and moderately high, posterior mesonotum moderately steep, mesonotal process indistinct, tubercle-like; promesonotal groove absent; metanotal groove absent; propodeal spines moderate, thin, with acute apex; humeral area laterally weakly produced (Fig. 25D). Surface shiny, promesonotum predominantly rugofoveolate, with additional sparse, thin, transverse rugae on pronotum; propodeum with fine rugofoveolae. Pilosity sparse, moderately long, and erect (Fig. 25D, F). ***Petiole.*** Shiny with fine and sparse rugofoveolae; node smooth to indistinctly rugulose, low, triangular, with rounded apex, in rear view node dorsoventrally convex; pilosity moderately sparse and erect (Fig. 25D, F). ***Postpetiole.*** Shiny, with fine and sparse rugulae; in dorsal view postpetiole oval, lateral margins medially with two dentate projections; pilosity long, moderately sparse, and erect (Fig. 25D, F). ***Gaster.*** Shiny and smooth; pilosity sparse, long, and erect (Fig. 25D, F). ***Colour.*** Yellowish orange; mandibles and anterior part of head slightly darker (Fig. 25D, F).

**Minor workers.** Measurements (*N* = 9): HL: 0.57–0.64 (0.59); HW: 0.47–0.53 (0.5); SL: 0.59–0.66 (0.62); EL: 0.09–0.11 (0.1); WL: 0.7–0.79 (0.74); PSL: 0.06–0.08 (0.07); MTL: 0.48–0.56 (0.52); PNW: 0.3–0.38 (0.33); PTW: 0.07–0.1 (0.09); PPW: 0.13–0.16 (0.14); CI: 112.2–122.3 (119.5); SI: 121.6–130.3 (125.5); PSLI: 10.8–12.6 (11.8); PPI: 51.1–70.3 (61.2); PNI: 64.0–70.2 (66.2); MTI: 101.9–108.0 (104.8).

***Head.*** Cephalic margin indistinctly convex (Fig. 25A). Pilosity relatively sparse, long, and subdecumbent. Head foveolate with additional indistinct rugulae on frons and vertex, area posterolateral from eyes with reduced sculpture and partially smooth; antennal sockets with few thick, curved outward rugae and foveolate interspaces. Clypeus with median longitudinal carina absent; two lateral longitudinal carinae absent. Scape, when laid back, surpassing the posterior head margin by two-fifths of its length; pilosity dense, subdecumbent to erect (Fig. 25A, C). ***Mesosoma.*** In lateral view, promesonotum moderately high, short, arched; promesonotal groove present, distinct; metanotal groove present and distinct; propodeal spines indistinct, triangular, apex acute (Fig. 25C). Sculpture smooth and shiny, only dorsum with few transverse, indistinct rugulae and propodeum with indistinct and sparse foveolae. Pilosity sparse, moderately long, and erect (Fig. 25C, E). ***Petiole.*** Peduncle with ventral face relatively straight; node low, knobbed, and small; with few short, erect setae (Fig. 25C, E). ***Gaster.*** With sparse, erect pilosity (Fig. 25C, E). ***Colour.*** Dark yellow (Fig. 25C, E).

##### Etymology.

From the type locality.

##### Biology.

The species was collected at 1325 m in elevation, in montane rainforest. Nests were located in rotten logs, root mat and rotten tree stump.

##### Comments.

*Pheidole antranohofa* sp. nov. is known only from its type locality: Parc National de Marojejy in Antsiranana. Morphologically it is most similar to *P. sikorae* Forel which is widely distributed from Forêt Ambohidena in Toamasina to Andrambovato in Fianarantsoa. Major workers of *P. antranohofa* sp. nov. can be distinguished based on developed sculpture of mesosoma, absence of impressed and smooth concavity placed lateral to antennal socket and tentorial pit and distinctly rugofoveolate interspaces on frons. However, minors of *P. antranohofa* sp. nov. and *P. sikorae* are indistinguishable.

#### Pheidole beanka sp. nov.

Taxon classificationAnimalia

B6307494-8C6A-5F26-9BAA-673F0A21EAEF

http://zoobank.org/4465D6B7-A7E3-4C86-A4DA-45D197B2731F

**Figure 26.**
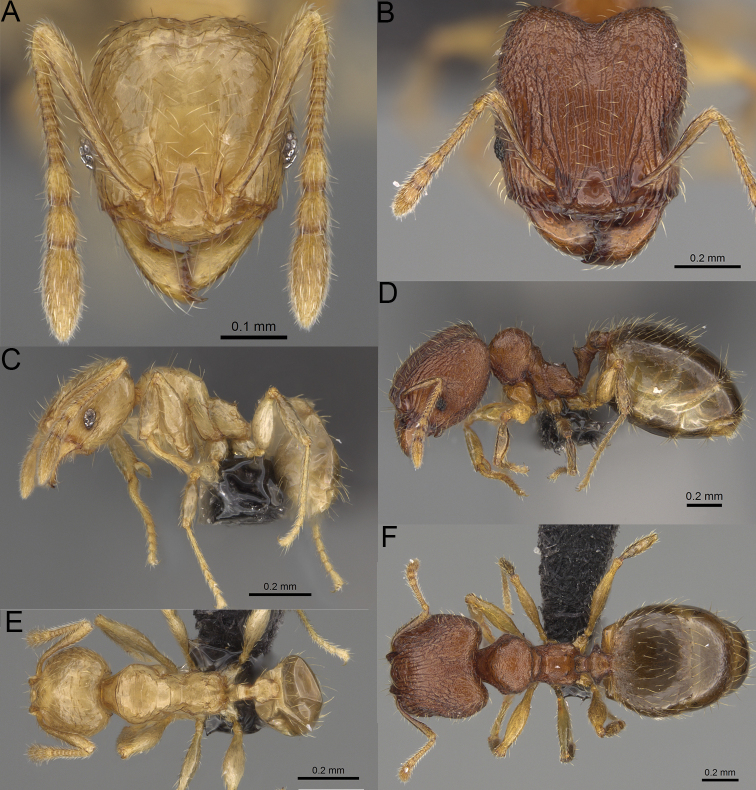
*Pheidole beanka* sp. nov., full-face view (**A**), profile (**C**), and dorsal view (**E**) of paratype minor worker (CASENT0923276) and full-face view (**B**), profile (**D**), and dorsal view (**F**) of holotype major worker (CASENT0156684).

Figs 26A–F , 63H , 65H

##### Type material.

***Holotype.*** Madagascar. • 1 major worker; Mahajanga; Réserve forestière Beanka, 53.6 km E Maintirano; -18.04014, 44.53394; alt. 272 m; 25 Oct 2009; B. L. Fisher et al. leg.; tropical dry forest on tsingy, ex rotten log; BLF22970; CASENT0156684 (CASC). ***Paratype.*** • 1w.; same data as for holotype, CASENT0923276 (CASC).

##### Diagnosis.

Minute species. ***Major workers.***HL < 0.9 mm and WL < 0.7 mm; head in full-face view elongate, not widening posteriorly with posterior sides slightly convex, in lateral view sub-oval and short with convex dorsal and ventral sides; body ocherous; sides of head with moderately dense, moderately short, suberect to erect pilosity; entire head distinctly sculptured, medial part of frons with thick and longitudinal rugae and distinctly rugulate and never smooth interspaces, occipital lobes with dense and irregular rugae with distinctly rugoreticulate interspaces; scape, when laid back, exceeding the midlength of head by one-fifth of its length; lateral sides of promesonotum distinctly foveolate without additional sparse and indistinct rugulae; inner hypostomal teeth distinct, large, closely spaced, triangular, with rounded apex directed upward; outer hypostomal teeth lobe-like, approximately the same height and width as inner teeth; inner and outer hypostomal teeth closely spaced and not connected by concavity; base of first gastral tergite shagreened. ***Minor workers.***HL < 0.45 mm and WL < 0.5 mm, scape, when laid back, surpassing the posterior head margin by one-fifth of its length; propodeal spines reduced to small tubercles; head relatively rectangular; body yellowish brown; lateral sides of frons smooth; vertex with transverse, sparse, and moderately thick, short rugulae; mesosoma smooth.

##### Description.

**Major workers.** Measurements (*N* = 1): HL: 0.86; HW: 0.74; SL: 0.45; EL: 0.1; WL: 0.7; PSL: 0.09; MTL: 0.38; PNW: 0.39; PTW: 0.12; PPW: 0.3; CI: 115.3; SI: 60.3; PSLI: 10.5; PPI: 40.4; PNI: 51.9; MTI: 51.1.

***Head.*** In full-face view elongate, not widening posteriorly, with anterior and posterior sides slightly convex (Fig. 26B). In lateral view sub-oval; ventral and dorsal faces convex; inner hypostomal teeth visible. Sides of the head with moderately dense, moderately short, suberect to erect pilosity; whole head with dense, long, decumbent to erect pilosity. Medial part of frons with thick, longitudinal, and dense rugae and distinctly rugulate interspaces, rugae more irregular and with denser sculpture of interspaces on posteromedial part; lateral sides with thick, dense and longitudinal rugae with distinctly rugofoveolate interspaces, rugae more irregular on posterolateral parts. Occipital lobes with dense, irregular rugae and distinctly rugoreticulate interspaces. Area posterolateral from eyes with dense rugofoveolae. Gena with relatively sparse and thick, longitudinal rugae and indistinctly rugoreticulate interspaces. Centre of clypeus smooth and shiny, lateral sides with indistinct rugulae; median notch present, moderately wide, and shallow; median longitudinal carina absent; lateral longitudinal carinae absent. Scape, when laid back, exceeding the midlength of head by one-fifth of its length; pilosity subdecumbent to erect (Fig. 26B, D). Inner hypostomal teeth distinct, large, closely spaced, triangular, with rounded apex directed upward; outer hypostomal teeth lobe-like, approximately the same height and width as inner teeth; inner and outer hypostomal teeth closely spaced and not connected by concavity (Fig. 63H). ***Mesosoma.*** In lateral view, promesonotum short, angular, and moderately high, posterior mesonotum moderately steep, mesonotal process very indistinct, tubercle-like; promesonotal groove absent; metanotal groove absent; propodeal spines very small, with wide base and acute apex; humeral area produced (Fig. 26D). Surface shiny and distinctly foveolate; katepisternum with sparser foveolae. Pilosity sparse, moderately long, and erect (Fig. 26D, F). ***Petiole.*** Shiny with dense foveolae; node finely foveolate, triangular, with rounded and thin apex, in rear view node dorsoventrally concave; pilosity moderately sparse and erect (Fig. 26D, F). ***Postpetiole.*** Shiny and foveolate; dorsum with reduced sculpture; in dorsal view oval, lateral margins medially with two distinct dentate projections; pilosity long, moderately sparse, and erect (Fig. 26D, F). ***Gaster.*** Shiny and smooth with shagreened base; pilosity moderately dense, moderately short, and erect (Fig. 26D, F). ***Colour.*** Ochreous with yellow legs and antenna (Fig. 26D, F).

**Minor workers.** Measurements (*N* = 1): HL: 0.44; HW: 0.39; SL: 0.39; EL: 0.1; WL: 0.48; PSL: 0.05; MTL: 0.3; PNW: 0.25; PTW: 0.06; PPW: 0.11; CI: 112.8; SI: 98.7; PSLI: 10.9; PPI: 48.7; PNI: 65.0; MTI: 77.0.

***Head.*** Cephalic margin slightly concave (Fig. 26A). Pilosity relatively sparse, moderately long, decumbent to subdecumbent. Sculpture shiny and smooth; vertex with transverse, sparse, and moderately thick, short rugulae; antennal sockets with few thick, curved outward rugae and smooth interspaces. Clypeus with median longitudinal carina absent; two lateral longitudinal carinae absent. Scape, when laid back, exceeding the posterior head margin by one-fifth of its length; pilosity dense, subdecumbent to erect (Fig. 26A, C). ***Mesosoma.*** In lateral view, promesonotum moderately high and short, box-like; promesonotal groove absent; metanotal groove distinct; propodeal spines reduced to small tubercles (Fig. 26C). Sculpture shiny and smooth. Pilosity sparse, moderately long, and erect (Fig. 26C, E). ***Gaster.*** With sparse, erect pilosity (Fig. 26C, E). ***Colour.*** Yellow (Fig. 26C, E).

##### Etymology.

From the type locality.

##### Biology.

The species was collected at 272 m in elevation, in tropical dry forest. Nest was located in rotten log.

##### Comments.

*Pheidole beanka* sp. nov. belongs to the group of species characterised by small body size (major workers: HL < 1.05 mm, WL < 0.9 mm and minor workers HL < 0.5 mm, WL < 0.6 mm), elongate and not widening posteriorly head in major workers, and minor workers with predominantly smooth and relatively rectangular head and yellow to brown body colouration. The group includes five species: *P. flavominuta* sp. nov., *P. nitidobruna* sp. nov., *P. mikros* sp. nov., and *P. beanka* sp. nov. *Pheidole beanka* sp. nov., described from Réserve forestière Beanka, is the only member of this group known from the Mahajanga and its distribution doesn’t overlap with the remaining four taxa. However, morphology of *P. beanka* sp. nov. is most similar to *P. mikros* sp. nov., which is distributed on the southernmost part of the Antsiranana prefecture. Major workers of *Pheidole beanka* sp. nov. differ from *P. mikros* sp. nov. in the combination of the following characters: medial part of frons with distinctly rugulate interspaces and very small propodeal spines; minor workers can be separated based on smooth lateral sides of frons and mesosoma, yellow body, and propodeal spines reduced to small tubercles.

#### Pheidole befotaka sp. nov.

Taxon classificationAnimalia

33D35FE1-AAA8-5B28-A864-18BD17B337D1

http://zoobank.org/E3B4F08F-A5CF-409A-AB97-EA9951ACA186

**Figure 27.**
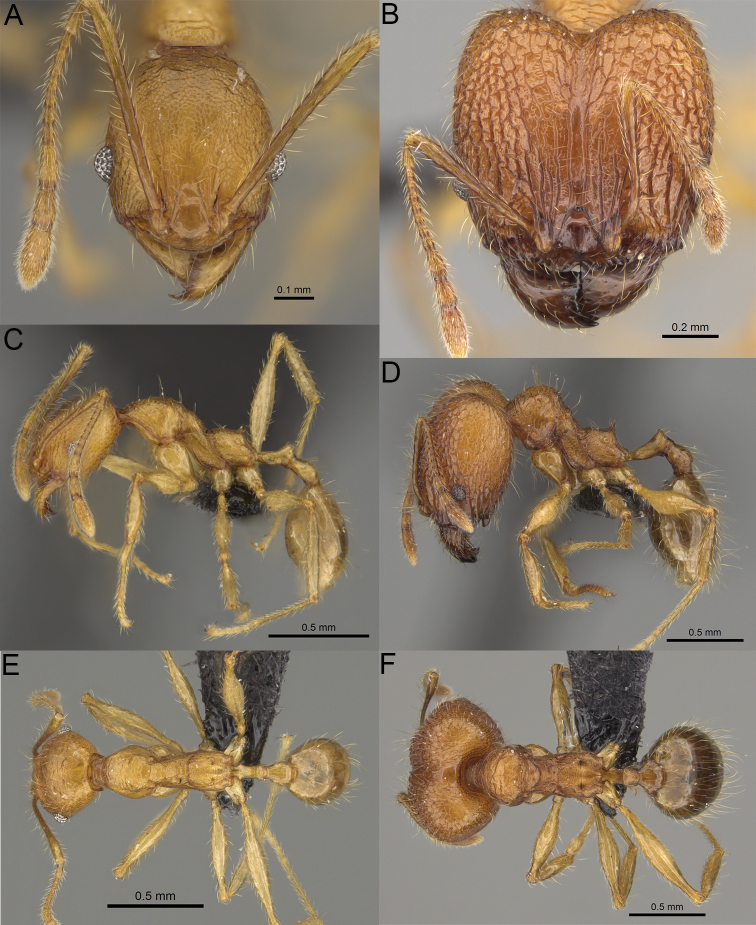
*Pheidole befotaka* sp. nov., full-face view (**A**), profile (**C**), and dorsal view (**E**) of paratype minor worker (CASENT0235125) and full-face view (**B**), profile (**D**), and dorsal view (**F**) of holotype major worker (CASENT0119590).

Figs 27A–F , 63I , 65I

##### Type material.

***Holotype.*** Madagascar. • 1 major worker; Fianarantsoa; Parc National Befotaka-Midongy, Papango 28.5km S Midongy-Sud, Mount Papango; -23.84083, 46.9575; alt. 1250 m; 17 Nov 2006; B. L. Fisher et al. leg.; montane rainforest, ex rotten log; BLF14974; CASENT0119590 (CASC). ***Paratype.*** • 1w.; same data as for holotype; CASENT0235125 (CASC).

##### Other material.

Madagascar. –**Fianarantsoa**: •1w., 1s.; Parc National Befotaka-Midongy, Papango 27.7km S Midongy-Sud, Mount Papango; -23.83517, 46.96367; alt. 940 m; 16 Nov 2006; B. L. Fisher et al. leg.; rainforest, ex dead twig above ground; BLF14931 (CASC).

##### Diagnosis.

Moderately large species. ***Major workers.*** Head in full-face view sub-oval, slightly widening posteriorly, with anterior and posterior sides convex, in lateral view sub-oval, ventral and dorsal faces convex; sides of the head with dense, moderately long, erect pilosity; medial part of frons with moderately dense and thick rugae, anteriorly rugae longitudinal and interrupted, posteromedially rugae irregular, interspaces shiny with sparse and indistinct irregular rugulae; lateral sides with irregular, thick, and relatively sparse rugae with indistinctly rugoreticulate interspaces; occipital lobes, and area posterolateral from eyes never smooth; scape, when laid back, exceeding the midlength of head by two-fifths of its length; inner hypostomal teeth distinct, large, closely spaced, triangular, with rounded apex directed upward; outer hypostomal teeth lobe-like, wider than inner hypostomal teeth and approximately the same height, apex directed outward; inner and outer hypostomal teeth closely spaced and connected by indistinct concavity; pronotum and mesonotum with fine rugoreticulae and additional transverse to irregular, thick rugae; anepisternum, katepisternum and propodeum with fine rugoreticulae; body orange. ***Minor workers.*** Head foveolate; medial part of frons with smooth notch; vertex and lateral sides of frons with additional, irregular to arcing rugae, area posterolateral from eyes smooth; scape, when laid back, surpassing the posterior head margin by two-fifths of its length; promesonotum moderately low and moderately short; promesonotal groove indistinct; propodeal spines small and triangular; mesosoma with very indistinct and sparse rugofoveolae; dorsal pronotum and medial parts of lateral sides of pronotum, katepisternum, and anepisternum smooth; body yellow.

##### Description.

**Major workers.** Measurements (*N* = 2): HL: 1.18, 1.12; HW: 1.18, 1.1; SL: 0.71, 0.73; EL: 0.16, 0.16; WL: 1.06, 1.05; PSL: 0.19, 0.17; MTL: 0.73, 0.72; PNW: 0.51, 0.48; PTW: 0.17, 0.14; PPW: 0.31, 0.31; CI: 100.5, 101.8; SI: 60.6, 66.3; PSLI: 16.0, 15.2; PPI: 56.9, 45.4; PNI: 43.0, 44.0; MTI: 62.2, 65.7.

***Head.*** In full-face view sub-oval, slightly widening posteriorly, with anterior and posterior sides convex (Fig. 27B). In lateral view sub-oval; ventral and dorsal faces convex; inner hypostomal teeth visible. Sides of the head with dense, moderately long, erect pilosity; whole head with dense, long, decumbent to erect pilosity. Medial part of frons with moderately dense and thick rugae, anteriorly rugae longitudinal and interrupted, posteromedially rugae irregular, interspaces shiny with sparse and indistinct irregular rugulae; lateral sides with irregular, thick, and relatively sparse rugae with indistinctly rugoreticulate interspaces. Occipital lobes with sparse, thick, and irregular rugae; interspaces smooth. Gena with relatively dense and thick longitudinal rugae and smooth interspaces. Area posterolateral from eyes with thin and dense rugofoveolae, sculpture weakening posteriorly. Centre of clypeus smooth and shiny, lateral sides with indistinct rugulae; median notch present, moderately wide, and shallow; median longitudinal carina present; lateral longitudinal carinae absent. Scape, when laid back, exceeding the midlength of head by two-fifths of its length; pilosity subdecumbent to erect (Fig. 27B, D). Inner hypostomal teeth distinct, large, closely spaced, triangular, with rounded apex directed upward; outer hypostomal teeth lobe-like, wider than inner hypostomal teeth and approximately the same height, apex directed outward; inner and outer hypostomal teeth closely spaced and connected by indistinct concavity (Fig. 63I). ***Mesosoma.*** In lateral view, promesonotum short, angular, and moderately low, posterior mesonotum moderately steep, mesonotal process indistinct, tubercle-like; promesonotal groove absent; metanotal groove indistinct; propodeal spines moderately long, moderately wide, with acute apex; humeral area laterally weakly produced (Fig. 27D). Surface shiny; pronotum and mesonotum with fine rugoreticulae and additional transverse to irregular, thick rugae; anepisternum, katepisternum, and propodeum with fine rugoreticulae. Pilosity relatively dense, long, and erect (Fig. 27D, F). ***Petiole.*** Shiny with fine and dense rugofoveolae; node smooth, low, triangular, with rounded and thin apex, in rear view node dorsoventrally slightly convex; pilosity moderately sparse and erect (Fig. 27D, F). ***Postpetiole.*** Shiny and smooth; in dorsal view oval, lateral margins medially with two dentate projections; pilosity long, moderately sparse, and erect (Fig. 27D, F). ***Gaster.*** Shiny and smooth; pilosity moderately dense, long, and erect (Fig. 27D, F). ***Colour.*** Orange; mandibles and gaster slightly darker; legs yellowish (Fig. 27D, F).

**Minor workers.** Measurements (*N* = 2): HL: 0.66, 0.64; HW: 0.54, 0.52; SL: 0.76, 0.74; EL: 0.13, 0.13; WL: 0.85, 0.86; PSL: 0.09, 0.1; MTL: 0.61, 0.61; PNW: 0.36, 0.36; PTW: 0.1, 0.08; PPW: 0.17, 0.15; CI: 121.2, 123.2; SI: 140.0, 141.0; PSLI: 13.5, 14.9; PPI: 58.2, 55.0; PNI: 66.4, 69.2; MTI: 112.0, 117.0.

***Head.*** Cephalic margin indistinctly convex or straight (Fig. 27A). Pilosity relatively sparse, long, decumbent to suberect. Sculpture foveolate; medial part of frons with smooth notch; vertex and lateral sides of frons with additional, irregular to arcing rugae; area posterolateral from eyes smooth. Clypeus with median longitudinal carina absent; two lateral longitudinal carinae absent. Scape, when laid back, surpassing the posterior head margin by two-fifths of its length; pilosity dense, subdecumbent to erect (Fig. 27A, C). ***Mesosoma.*** In lateral view, promesonotum moderately low and moderately short, arched; promesonotal groove absent; metanotal groove indistinct; propodeal spines small and triangular (Fig. 27C). Sculpture with very indistinct and sparse rugofoveolae; dorsal pronotum and medial parts of lateral sides of pronotum, katepisternum, and anepisternum smooth. Pilosity very sparse, moderately long, and erect (Fig. 27C, E). ***Postpetiole.*** Short, low, and relatively flat; with few short, erect setae (Fig. 27C, E). ***Gaster.*** With sparse, erect pilosity (Fig. 27C, E). ***Colour.*** Yellow, vertex slightly darker (Fig. 27C, E).

##### Etymology.

From the type locality.

##### Biology.

The species was collected between 940–1250 m in elevation, in rainforest and montane rainforest. Nests were located in rotten logs and dead twigs above ground.

##### Comments.

*Pheidole befotaka* sp. nov. is a member of a group of species characterised by body coloration that is bright, yellow to orange in majors and yellow in minors, head sub-oval, not widening posteriorly with sides not convex or convex indistinctly, and medial part of frons with longitudinal and interrupted rugae anteriorly and distinctly irregular rugae posteriorly. The group includes three taxa: *P. vony* sp. nov., *P. befotaka* sp. nov., and *P. mamiratra* sp. nov. *Pheidole befotaka* sp. nov. is the only member of the group known from Parc National Befotaka-Midongy in Fianarantsoa and can be easily separated based on orange body of major workers with slightly convex lateral sides of head and lateral sides of frons with distinctly irregular rugae. Minor workers of *P. befotaka* sp. nov. can be separated from *P. vony* sp. nov. and *P. mamiratra* sp. nov. based on presence of additional, irregular to arcing rugae on vertex and lateral sides of frons. Additionally, minors of *P. befotaka* sp. nov. can be confused with minors of sympatric *P. veteratrix* from which they differ in reduced sculpture of mesosoma and higher promesonotum.

#### Pheidole dasos sp. nov.

Taxon classificationAnimalia

306B1012-E807-5133-80E1-A0347C50C614

http://zoobank.org/CAD761EC-FFAC-4EC6-B80E-F4C0720AF757

**Figure 28.**
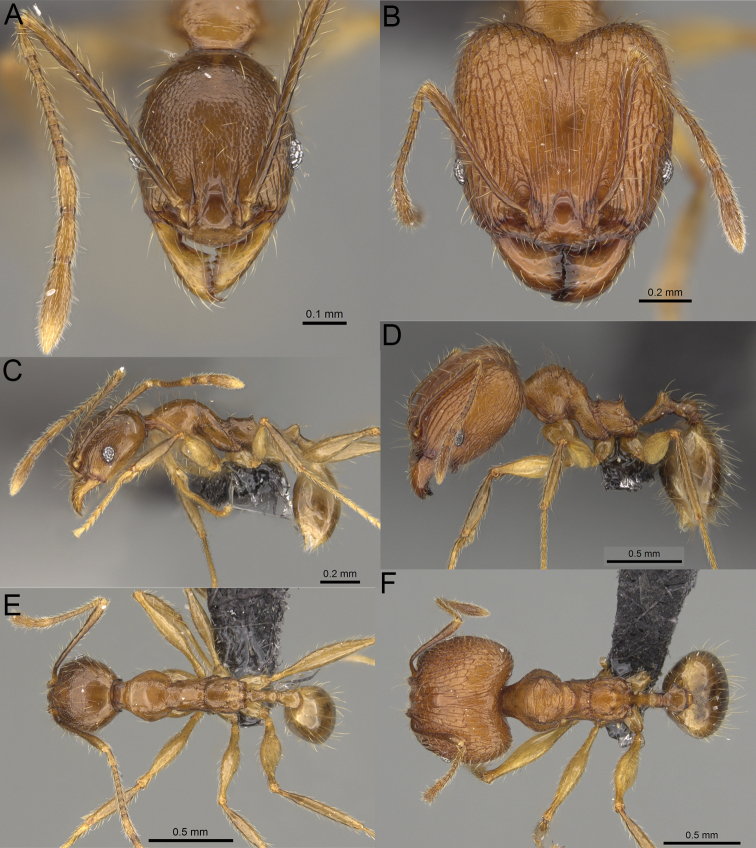
*Pheidole dasos* sp. nov., full-face view (**A**), profile (**C**), and dorsal view (**E**) of paratype minor worker (CASENT0923284) and full-face view (**B**), profile (**D**), and dorsal view (**F**) of holotype major worker (CASENT0212441).

Figs 28A–F , 63J , 65J

##### Type material.

***Holotype.*** Madagascar. • 1 major worker; Antsiranana; Makirovana forest; -14.104, 50.03574; alt. 225 m; 4 May 2011; B. L. Fisher et al. leg.; rainforest, ex rotten log; BLF27046; CASENT0212441 (CASC). ***Paratype.*** • 1w.; same data as for holotype, CASENT0923284 (CASC).

##### Other material.

Madagascar. –**Antsiranana**: •1w., 1s.; Makirovana forest; -14.17066, 49.95409; alt. 415 m; 29 Apr 2011; B. L. Fisher et al. leg.; rainforest, ex rotten log; BLF26619 (CASC).

##### Diagnosis.

Moderately large species. ***Major workers.*** Head in full-face view sub-oval, not widening posteriorly, with anterior and posterior sides relatively straight, in lateral view sub-oval; ventral and dorsal faces convex; sides of the head with moderately dense, moderately long, subdecumbent to suberect pilosity; medial part of frons with thick, interrupted, sparse, and longitudinal rugae with indistinctly foveolate interspaces; anterolateral sides of frons with sparse, thick, and longitudinal rugae, posterolateral sides with rugae more irregular, interspaces with sparse foveolae; occipital lobes, and area posterolateral from eyes without smooth notches; scape, when laid back, exceeding the midlength of head by two-fifths of its length; inner hypostomal teeth distinct, large, closely spaced, triangular, with rounded apex directed upward; outer hypostomal teeth lobe-like, narrower, and approximately as high as inner teeth; inner and outer hypostomal teeth closely spaced and not connected by concavity; mesosoma rugofoveolate; dorsal promesonotum with additional thick and transverse rugae; lateral sides of pronotum with smooth notches; gaster smooth; body orange. ***Minor workers.*** Head foveolate; vertex and median frons with fading sculpture; area posterolateral from eyes smooth; antennal sockets with few indistinct, curved outward rugae and foveolate interspaces; scape, when laid back, exceeding the posterior head margin by one-third of its length; promesonotum low and moderately long; promesonotal groove absent; propodeal spines small and triangular; mesosoma predominantly smooth; lateral sides with very sparse and indistinct, thin rugulae; body brown.

##### Description.

**Major workers.** Measurements (*N* = 2): HL: 1.1, 1.14; HW: 1.04, 1.12; SL: 0.79, 0.81; EL: 0.16, 0.14; WL: 0.98, 1.05; PSL: 0.14, 0.15; MTL: 0.72, 0.77; PNW: 0.4, 0.45; PTW: 0.13, 0.13; PPW: 0.29, 0.31; CI: 105.9, 101.4; SI: 76.2, 72.6; PSLI: 12.4, 13.5; PPI: 45.8, 41.7; PNI: 38.8, 39.9; MTI: 68.8, 68.6.

***Head.*** In full-face view sub-oval, not widening posteriorly, with anterior and posterior sides relatively straight (Fig. 28B). In lateral view sub-oval; ventral and dorsal faces convex; inner hypostomal teeth visible. Sides of the head with moderately dense, moderately long, subdecumbent to suberect pilosity; whole head with dense, long, decumbent to erect pilosity. Medial part of frons with thick, interrupted, sparse, and longitudinal rugae with indistinctly foveolate interspaces; anterolateral sides of frons with sparse, thick, and longitudinal rugae, posterolateral sides with rugae more irregular, interspaces with sparse foveolae. Occipital lobes with slightly thinner, sparse, and irregular rugae and smooth to indistinctly foveolate interspaces. Area posterolateral from eyes with denser and thinner longitudinal rugae with rugofoveolate interspaces. Gena with relatively sparse and thick, longitudinal rugae with distinctly rugofoveolate interspaces. Centre of clypeus smooth and shiny, lateral sides with indistinct rugulae; median notch present, moderately wide, and shallow; median longitudinal carina absent; lateral longitudinal carinae absent. Scape, when laid back, exceeding the midlength of head by two-fifths of its length; pilosity subdecumbent to erect (Fig. 28B, D). Inner hypostomal teeth distinct, large, closely spaced, triangular, with rounded apex directed upward; outer hypostomal teeth lobe-like, narrower than and approximately as high as inner teeth; inner and outer hypostomal teeth closely spaced and not connected by concavity (Fig. 63J). ***Mesosoma.*** In lateral view, promesonotum short, angular and moderately low, posterior mesonotum moderately steep, mesonotal process indistinct, tubercle-like; promesonotal groove absent; metanotal groove absent; propodeal spines short, with wide base and acute apex; humeral area produced (Fig. 28D). Surface shiny and rugofoveolate; dorsal promesonotum with additional thick and transverse rugae; lateral sides of pronotum with smooth notches. Pilosity moderately dense, long, and erect (Fig. 28D, F). ***Petiole.*** Shiny with dense foveolae; node finely foveolate, triangular, with rounded and thick apex, in rear view node dorsoventrally slightly convex; pilosity moderately sparse and erect (Fig. 28D, F). ***Postpetiole.*** Shiny and foveolate; dorsum with reduced sculpture and smooth notch; in dorsal view oval, lateral margins medially with two dentate projections; pilosity long, moderately sparse and erect (Fig. 28D, F). ***Gaster.*** Shiny and smooth; pilosity moderately dense, long and erect (Fig. 28D, F). ***Colour.*** Orange with yellowish legs (Fig. 28D, F).

##### Description.

**Minor workers.** Measurements (*N* = 2): HL: 0.56, 0.61; HW: 0.46, 0.49; SL: 0.71, 0.74; EL: 0.1, 0.1; WL: 0.74, 0.79; PSL: 0.08, 0.09; MTL: 0.57, 0.59; PNW: 0.31, 0.32; PTW: 0.09, 0.07; PPW: 0.12, 0.14; CI: 122.0, 122.7; SI: 153.6, 150.9; PSLI: 13.8, 15.4; PPI: 74.3, 52.9; PNI: 67.5, 65.7; MTI: 123.3, 120.5.

***Head.*** Cephalic margin slightly convex (Fig. 28A). Pilosity relatively sparse, moderately long, subdecumbent to erect. Sculpture shiny and foveolate; vertex and median frons with fading sculpture; area posterolateral from eyes smooth; antennal sockets with few indistinct, curved outward rugae and foveolate interspaces. Clypeus with median longitudinal carina absent; two lateral longitudinal carinae absent. Scape, when laid back, exceeding the posterior head margin by one-third of its length; pilosity dense, subdecumbent to erect (Fig. 28A, C). ***Mesosoma.*** In lateral view, promesonotum low and moderately long, arched; promesonotal groove absent; metanotal groove distinct; propodeal spines small, triangular (Fig. 28C). Sculpture shiny and predominantly smooth; lateral sides with very sparse and indistinct, thin rugulae. Pilosity very sparse, moderately long, and erect (Fig. 28C, E). ***Gaster.*** With sparse, erect pilosity (Fig. 28C, E). ***Colour.*** Brown, legs and gaster brighter (Fig. 28C, E).

##### Etymology.

Greek for forest in reference to habitat preferences of the species.

##### Biology.

The species was collected between 225–415 m in elevation, in rainforest. Nests were located in rotten logs.

##### Comments.

*Pheidole dasos* sp. nov., described from Makirovana forest in Antsiranana, is most similar to *P. hazo* sp. nov., known from the vicinity of Andranoma in Antananarivo, and *P. sparsa* sp. nov., recorded so far only from Bemanevika in Mahajanga. Majors of *P. dasos* sp. nov. can be distinguished from both taxa by medial part of frons with moderately dense rugae with interspaces with indistinct foveolae, and pronotum predominantly sculptured with smooth notches on lateral sides; minors of *P. dasos* sp. nov. can be distinguished from *P. sparsa* sp. nov. and *P. hazo* sp. nov. by brown body colouration, longer scape exceeding the posterior head margin by one-third of its length, and predominantly smooth mesosoma with very sparse and indistinct, thin rugulae on lateral sides.

#### Pheidole flavominuta sp. nov.

Taxon classificationAnimalia

C4C1340F-83DE-5456-8B67-7A4731BC6DFD

http://zoobank.org/59199444-7C41-420D-8302-7EFBE5525DF0

**Figure 29.**
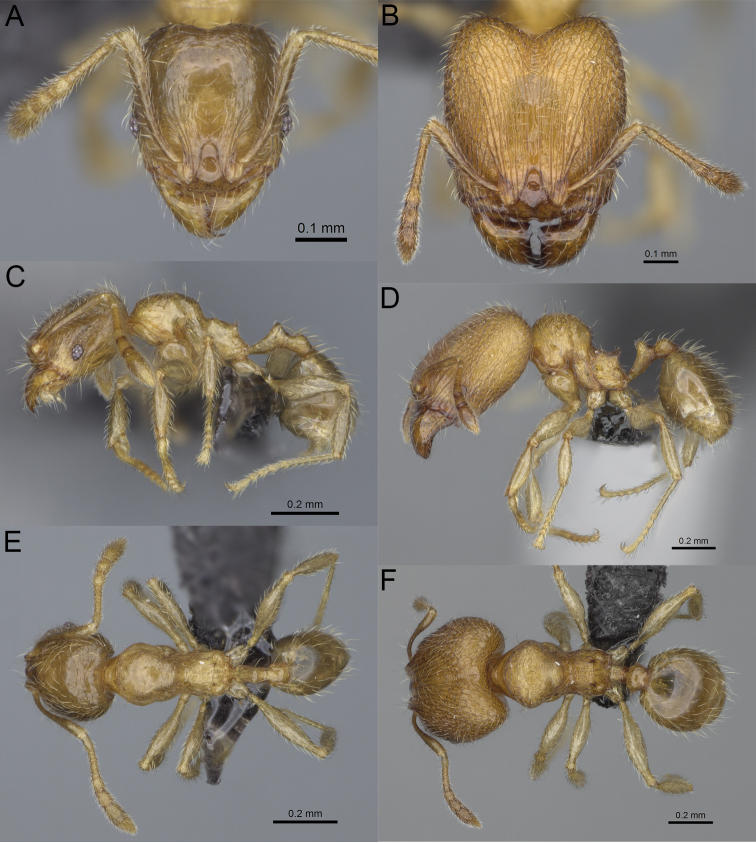
*Pheidole flavominuta* sp. nov., full-face view (**A**), profile (**C**), and dorsal view (**E**) of paratype minor worker (CASENT0162186) and full-face view (**B**), profile (**D**), and dorsal view (**F**) of holotype major worker (CASENT0923272).

Figs 29A–F , 63K , 65K

##### Type material.

***Holotype.*** Madagascar. • 1 major worker; Toamasina; Réserve Spéciale Ambatovaky, Sandrangato river; -16.81745, 49.2925; alt. 400 m; 26 Feb 2010; B. L. Fisher et al. leg.; rainforest, under root mat on ground; BLF24911; CASENT0923272 (CASC). ***Paratype.*** • 1w.; same data as for holotype, CASENT0162186 (CASC).

##### Other material.

Madagascar. –**Toamasina**: • 1w., 1s.; Reserve Betampona, Camp Vohitsivalana, 37.1 km 338° Toamasina; -17.88667, 49.2025; alt. 520 m; 1 Dec 2005; B. L. Fisher et al. leg.; rainforest, ex rotten log; BLF13262 (CASC). • 1w., 1s.; Réserve Spéciale Ambatovaky, Sandrangato river; -16.77468, 49.26551; alt. 355 m; 21 Feb 2010; B. L. Fisher et al. leg.; rainforest along river, under rootmat, litter on rock; BLF24505 (CASC). • 1w., 1s.; Réserve Spéciale Ambatovaky, Sandrangato river; -16.81745, 49.2925; alt. 400 m; 26 Feb 2010; B. L. Fisher et al. leg.; rainforest, ex rotten log; BLF24913 (CASC). • 2w.; Réserve Spéciale Ambatovaky, Sandrangato river; -16.81745, 49.2925; alt. 400 m; 26 Feb 2010; B. L. Fisher et al. leg.; rainforest, ex rotten log; BLF24943 (CASC). • 1w., 1s.; Réserve Spéciale Ambatovaky, Sandrangato river; -16.81745, 49.2925; alt. 400 m; 26 Feb 2010; B. L. Fisher et al. leg.; rainforest, ex rotten log; BLF24958 (CASC).

##### Diagnosis.

Minute species. ***Major workers.***HL < 0.85 mm and WL < 0.65 mm; head in full-face view elongate, not widening posteriorly with posterior sides slightly convex, in lateral view sub-oval and elongate with slightly convex dorsal and ventral sides; body yellow; sides of head with moderately dense, short, suberect to erect pilosity; entire head distinctly sculptured but with smooth interspaces; scape, when laid back, reaching the midlength of head; inner hypostomal teeth distinct, moderate, closely spaced, triangular, with rounded apex directed upward; outer hypostomal teeth lobe-like, approximately as high and wide as inner teeth; inner and outer hypostomal teeth moderately closely spaced and not connected by concavity; base of first gastral tergite smooth. ***Minor workers.***HL < 0.45 mm and WL < 0.5 mm, scape, when laid back, reaching the posterior head margin, propodeal spines small and triangular; head relatively rectangular; body yellow; head predominantly smooth; mesosoma smooth.

##### Description.

**Major workers.** Measurements (*N* = 5): HL: 0.76–0.83 (0.8); HW: 0.68–0.73 (0.71); SL: 0.39–0.41 (0.4); EL: 0.08–0.08 (0.08); WL: 0.61–0.66 (0.64); PSL: 0.1–0.13 (0.12); MTL: 0.33–0.36 (0.35); PNW: 0.36–0.4 (0.37); PTW: 0.09–0.1 (0.1); PPW: 0.17–0.19 (0.18); CI: 111.3–115.3 (113.0); SI: 53.7–58.7 (56.2); PSLI: 12.8–15.5 (14.5); PPI: 47.4–60.8 (54.6); PNI: 51.4–53.9 (52.6); MTI: 48.2–49.6 (49.0).

***Head.*** In full-face view elongate, not widening posteriorly, with anterior and posterior sides slightly convex (Fig. 29B). In lateral view sub-oval and elongate; ventral and dorsal faces convex; inner hypostomal teeth visible. Sides of the head with moderately dense, short, suberect to erect pilosity; whole head with dense, long, decumbent to erect pilosity. Medial part of frons with thick, longitudinal, and dense rugae and smooth to indistinctly rugulate interspaces, rugae directed slightly outward on posteromedial part; lateral sides with thick, dense, and irregular rugae, interspaces shiny and distinctly rugoreticulate. Occipital lobes with dense, irregular rugae and distinctly rugoreticulate interspaces. Area posterolateral from eyes with weaker sculpture, rugoreticulate. Gena with relatively sparse and thick longitudinal rugae and smooth interspaces. Centre of clypeus smooth and shiny, lateral sides with indistinct rugulae; median notch present, moderately wide, and shallow; median longitudinal carina absent; lateral longitudinal carinae absent. Scape, when laid back, reaching the midlength of head; pilosity subdecumbent to erect (Fig. 29B, D). Inner hypostomal teeth distinct, moderate, closely spaced, triangular, with rounded apex directed upward; outer hypostomal teeth lobe-like, approximately as high and wide as inner teeth; inner and outer hypostomal teeth moderately closely spaced and not connected by concavity (Fig. 63K). ***Mesosoma.*** In lateral view, promesonotum short, angular, and moderately low, posterior mesonotum steep, mesonotal process very indistinct, bulge-like; promesonotal groove absent; metanotal groove absent; propodeal spines moderately long, with wide base and acute apex; humeral area laterally weakly produced (Fig. 29D). Surface shiny; promesonotum predominantly smooth with very fine network of rugoreticulae; anepisternum, katepisternum, and propodeum with rugoreticulae denser and thicker. Pilosity moderately dense, moderately long, and erect (Fig. 29D, F). ***Petiole.*** Shiny with dense foveolae; node smooth to finely foveolate, triangular, with rounded and thick apex, in rear view node dorsoventrally convex; pilosity moderately sparse and erect (Fig. 29D, F). ***Postpetiole.*** Shiny and foveolate; dorsum with reduced sculpture and smooth notch; in dorsal view oval, lateral margins medially with two dentate projections; pilosity long, moderately sparse, and erect (Fig. 29D, F). ***Gaster.*** Shiny and smooth; pilosity moderately dense, moderately short, and erect (Fig. 29D, F). ***Colour.*** Yellow (Fig. 29D, F).

**Minor workers.** Measurements (*N* = 5): HL: 0.42–0.44 (0.43); HW: 0.37–0.39 (0.38); SL: 0.34–0.36 (0.35); EL: 0.06–0.07 (0.06); WL: 0.46–0.49 (0.47); PSL: 0.05–0.07 (0.06); MTL: 0.25–0.28 (0.26); PNW: 0.23–0.26 (0.25); PTW: 0.05–0.07 (0.06); PPW: 0.09–0.11 (0.1); CI: 108.4–117.2 (114.8); SI: 88.2–95.7 (93.2); PSLI: 12.2–16.9 (14.6); PPI: 47.7–70.7 (63.8); PNI: 59.6–69.4 (66.4); MTI: 67.7–70.3 (69.0).

***Head.*** Cephalic margin indistinctly concave to straight (Fig. 29A). Pilosity relatively dense, moderately long, decumbent to suberect. Sculpture shiny and smooth; lateral sides of frons with fine, sparse, longitudinal, and interrupted rugulae; vertex with indistinct, sparse, and transverse rugulae; antennal sockets with few thick, curved outward rugae and smooth interspaces. Clypeus with median longitudinal carina absent; two lateral longitudinal carinae absent. Scape, when laid back, reaching the posterior head margin; pilosity dense, subdecumbent to erect (Fig. 29A, C). ***Mesosoma.*** In lateral view, promesonotum moderately high and short, arched; promesonotal groove absent; metanotal groove distinct; propodeal spines small and triangular (Fig. 29C). Sculpture shiny and smooth. Pilosity moderately sparse, moderately long, and erect (Fig. 29C, E). ***Gaster.*** With sparse, erect pilosity (Fig. 29C, E). ***Colour.*** Yellow, head and gaster slightly darker (Fig. 29C, E).

##### Etymology.

Latin for bright and small in reference to small body size and bright colouration.

##### Biology.

The species was collected between 355–520 m in elevation, in rainforest. Nests were located in rotten logs, and under rootmats on the ground.

##### Comments.

*Pheidole flavominuta* sp. nov. belongs to the group of species characterised by small body size (major workers: HL < 1.05 mm, WL < 0.9 mm and minor workers HL < 0.5 mm, WL < 0.6 mm), head in major workers elongate and not widening posteriorly, minor workers with predominantly smooth and relatively rectangular head, and yellow to brown body colouration. The group includes four species: *P. flavominuta* sp. nov., *P. nitidobruna* sp. nov., *P. mikros* sp. nov., and *P. beanka* sp. nov. Distribution of *P. flavominuta* sp. nov. covers area north from Toamasina up to Antenina and doesn’t overlap with other members of this group. *Pheidole flavominuta* sp. nov. strongly differs from other members of the group by having distinctly yellow body colouration in major and minor workers. Additionally, major workers have head, in lateral view, sub-oval and elongate with slightly convex dorsal and ventral sides, and first gastral tergite is entirely smooth. Minor workers of this species can be also separated by a short antennal scape which doesn’t exceed the posterior head margin.

#### Pheidole gracilis sp. nov.

Taxon classificationAnimalia

4DCC0AC3-56F0-542C-8E5B-42FC62D1E037

http://zoobank.org/687877F4-A954-4004-94E3-135722F8442A

**Figure 30.**
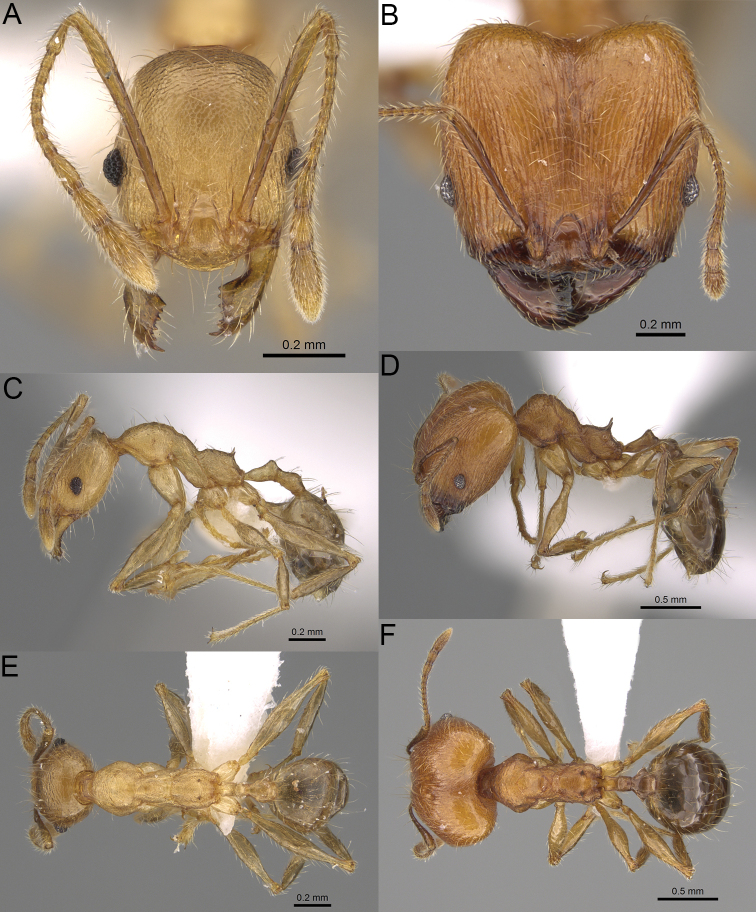
*Pheidole gracilis* sp. nov., full-face view (**A**), profile (**C**), and dorsal view (**E**) of paratype minor worker (CASENT0403309) and full-face view (**B**), profile (**D**), and dorsal view (**F**) of holotype major worker (CASENT0403308).

Figs 30A–F , 63L , 65L

##### Type material.

***Holotype.*** Madagascar. • 1 major worker; Antsiranana; Parc National Montagne d’Ambre, 12.2 km 211°SSW Joffreville; -12.59639, 49.1595; alt. 1300 m; 2 Feb 2001; Fisher et al. leg.; montane rainforest, ex dead branch above ground; BLF02825; CASENT0403308, middle specimen on the pin (CASC). ***Paratypes.*** • 6w., 4s.; same data as for holotype, CASENT0403307, CASENT0872252, CASENT0403309, CASENT0403310 (CASC, MHNG, PBZT).

##### Other material.

Madagascar. –**Antsiranana**: •2w., 3s.; Parc National Montagne d’Ambre, 12.2 km 211°SSW Joffreville; -12.59639, 49.1595; alt. 1300 m; 2 Feb 2001; Fisher et al. leg.; montane rainforest, ex dead branch above ground; BLF02844 (CASC).

##### Diagnosis.

Moderately large species. ***Major workers.*** Head in full-face view sub-oval and not widening posteriorly, with anterior and posterior sides slightly convex, in lateral view sub-oval; ventral and dorsal faces convex; sides of the head with dense, short, decumbent to suberect pilosity; medial part of frons with moderately thick, longitudinal, interrupted, and moderately dense rugae, interspaces rugulate, rugae directed slightly outward on posteromedial part; occipital lobes predominantly smooth, only anterior part with indistinct, dense, thin, and longitudinal rugae; area posterolateral from eyes with indistinct, dense, thin, and longitudinal rugae, posteriormost part smooth; scape, when laid back, exceeding the midlength of head by approximately two-fifths of its length; inner hypostomal teeth distinct, low, closely spaced, triangular, with rounded apex directed upward; outer hypostomal teeth dentate, wider and higher then inner teeth; inner and outer hypostomal teeth closely spaced and connected by indistinct concavity; mesosoma distinctly foveolate; promesonotum with additional sparse and moderately thick, irregular to transverse rugae on dorsum; katepisternum with smooth notch; body yellowish orange. ***Minor workers.*** Head foveolate; vertex with additional, short, and transverse rugulae; area posterolateral from eyes with weaker sculpture that appears smooth; scape, when laid back, exceeding the posterior head margin by two-fifths of its length; promesonotum low and moderately long; promesonotal groove present; propodeal spines moderately large with narrow base; mesosoma foveolate; katepisternum with smooth notch; body yellow to orange.

##### Description.

**Major workers.** Measurements (*N* = 8): HL: 1.22–1.32 (1.27); HW: 1.22–1.33 (1.27); SL: 0.72–0.82 (0.77); EL: 0.16–0.19 (0.17); WL: 1.08–1.22 (1.15); PSL: 0.16–0.21 (0.18); MTL: 0.76–0.83 (0.79); PNW: 0.48–0.53 (0.51); PTW: 0.14–0.17 (0.16); PPW: 0.3–0.37 (0.33); CI: 97.3–103.5 (99.8); SI: 56.7–63.3 (60.7); PSLI: 12.8–16.9 (14.6); PPI: 43.1–55.8 (49.0); PNI: 38.8–41.4 (39.8); MTI: 59.3–65.7 (62.1).

***Head.*** In full-face sub-oval, not widening posteriorly, with anterior and posterior sides slightly convex (Fig. 30B). In lateral view sub-oval; ventral and dorsal faces convex; inner hypostomal teeth visible. Sides of the head with dense, short, decumbent to suberect pilosity; whole head with dense, long, decumbent to erect pilosity. Medial part of frons with moderately thick, longitudinal, interrupted, and moderately dense rugae, interspaces rugulate, rugae directed slightly outward on posteromedial part; lateral sides with thick, dense, and longitudinal rugae with distinctly rugulate interspaces. Occipital lobes predominantly smooth, only anterior part with indistinct, dense, thin, and longitudinal rugae. Area posterolateral from eyes with indistinct, dense, thin, and longitudinal rugae, posteriormost part smooth and shiny. Gena with relatively sparse and thick longitudinal rugae and smooth to indistinctly rugulate interspaces. Centre of clypeus smooth and shiny, lateral sides with indistinct rugulae; median notch present, moderately wide, and shallow; median longitudinal carina absent; lateral longitudinal carinae absent. Scape, when laid back, exceeding the midlength of head by two-fifths of its length; pilosity subdecumbent to erect (Fig. 30B, D). Inner hypostomal teeth distinct, low, closely spaced, triangular, with rounded apex directed upward; outer hypostomal teeth dentate, wider and higher than inner teeth; inner and outer hypostomal teeth closely spaced and connected by indistinct concavity (Fig. 63L). ***Mesosoma.*** In lateral view, promesonotum short, angular, and moderately low, posterior mesonotum moderately steep, mesonotal process indistinct, tubercle-like; promesonotal groove absent; metanotal groove absent; propodeal spines moderately long, with narrow base and acute apex; humeral area weakly produced (Fig. 30D). Surface shiny and distinctly foveolate; promesonotum with additional sparse and moderately thick, irregular to transverse rugae on dorsum; katepisternum with smooth notch. Pilosity sparse, long, and erect (Fig. 30D, F). ***Petiole.*** Shiny with sparse foveolae; node finely foveolate, triangular, with rounded and thick apex, in rear view node dorsoventrally slightly concave; pilosity moderately sparse and erect (Fig. 30D, F). ***Postpetiole.*** Shiny and foveolate; dorsum with reduced sculpture and smooth notch; in dorsal view oval, lateral margins medially with two wide and dentate projections; pilosity long, moderately sparse, and erect (Fig. 30D, F). ***Gaster.*** Shiny and smooth; pilosity moderately sparse, moderately long, and erect (Fig. 30D, F). ***Colour.*** Yellowish orange with yellow legs (Fig. 30D, F).

**Minor workers.** Measurements (*N* = 10): HL: 0.65–0.71 (0.69); HW: 0.55–0.58 (0.56); SL: 0.69–0.74 (0.71); EL: 0.12–0.14 (0.13); WL: 0.84–0.89 (0.86); PSL: 0.1–0.12 (0.11); MTL: 0.58–0.66 (0.61); PNW: 0.36–0.4 (0.38); PTW: 0.07–0.1 (0.09); PPW: 0.15–0.19 (0.16); CI: 118.3–123.9 (121.5); SI: 123.7–132.8 (126.8); PSLI: 15.0–17.8 (16.3); PPI: 49.7–66.0 (57.0); PNI: 65.1–69.6 (67.4); MTI: 103.8–112.7 (108.9).

***Head.*** Cephalic margin slightly convex (Fig. 30A). Pilosity relatively sparse, moderately long, decumbent to subdecumbent. Sculpture shiny and foveolate; vertex with additional, short, and transverse rugulae; area posterolateral from eyes with weaker sculpture that appears smooth; antennal sockets with few thick, curved outward rugae and foveolate interspaces. Clypeus with median longitudinal carina absent; two lateral longitudinal carinae absent. Scape, when laid back, exceeding the posterior head margin by two-fifths of its length; pilosity dense, suberect to erect (Fig. 30A, C). ***Mesosoma.*** In lateral view, promesonotum low and moderately long, arched; promesonotal groove present; metanotal groove distinct; propodeal spines moderately large with narrow base, triangular (Fig. 30C). Sculpture shiny and foveolate; katepisternum with smooth notch. Pilosity very sparse, moderately long, and erect (Fig. 30C, E). ***Gaster.*** With sparse, erect pilosity (Fig. 30C, E). ***Colour.*** Orange to yellow (Fig. 30C, E).

##### Etymology.

Latin for slender in reference to body sculpture.

##### Biology.

The species was collected at 1300 m in elevation, in montane rainforest. Nests were located in dead branches above ground.

##### Comments.

*Pheidole gracilis* sp. nov. is a member of a group of species characterised by distinctly reduced head sculpture in major workers with occipital lobes entirely or predominantly smooth, area posterolateral from eyes partially or entirely smooth and shiny or with reduced sculpture and smooth notches. The group consists of four species: *P. litigiosa*, *P. masoandro* sp. nov., *P. gracilis* sp. nov., and *P. tampony* sp. nov. *Pheidole gracilis* sp. nov. is known only from Parc National Montagne d’Ambre in Antsiranana and its distribution doesn’t overlap with other members of this group. Morphologically the species is most similar to *P. litigiosa*, known from the vicinity of Antananarivo, and *P. masoandro* sp. nov., described from Anosyenne Mts. in Toliara. Its major workers can be easily separated from both of those taxa based on more developed head sculpture with occipital lobes anteriorly covered by indistinct, dense, thin, and longitudinal rugae and distinctly foveolate promesonotum; minor workers can be separated based on foveolate mesosoma and low and long promesonotum. Minor workers of *P. gracilis* sp. nov. are similar to minors of *P. tampony* sp. nov. and *P. befotaka* sp. nov. but can be separated based on more developed head sculpture with additional, short, and transverse rugulae on vertex and area posterolateral from eyes with weak but distinct sculpture and lacking smooth notches.

#### Pheidole haboka sp. nov.

Taxon classificationAnimalia

4047961A-DD30-5D3D-9506-662A1C6AABB2

http://zoobank.org/6176EC69-C18F-4B38-8FCB-D0543E48598C

**Figure 31.**
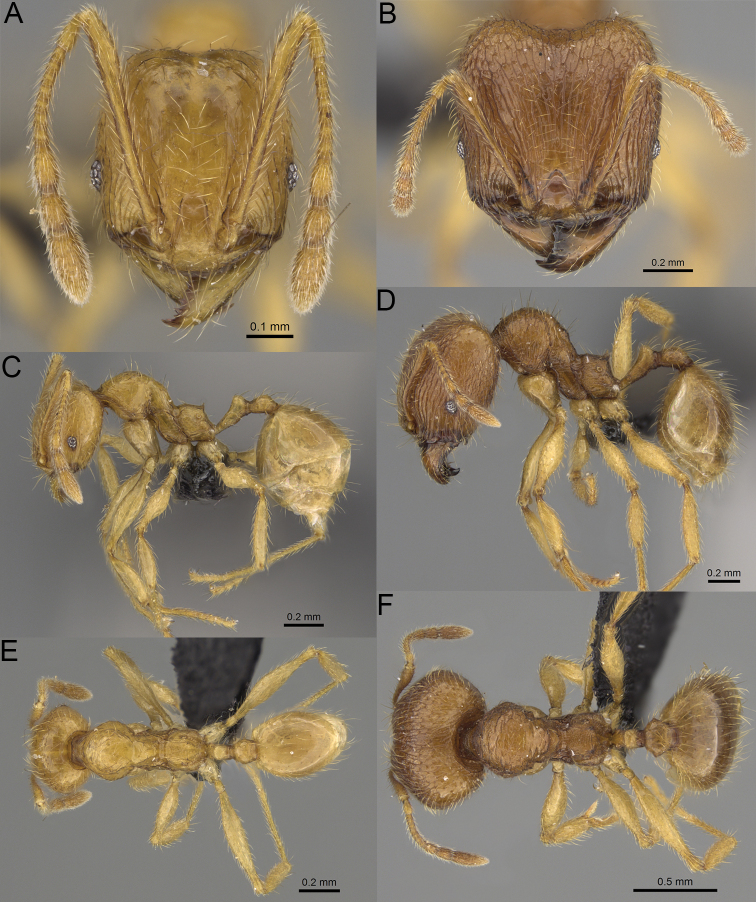
*Pheidole haboka* sp. nov., full-face view (**A**), profile (**C**), and dorsal view (**E**) of paratype minor worker (CASENT0491601) and full-face view (**B**), profile (**D**), and dorsal view (**F**) of holotype major worker (CASENT0491605).

Figs 31A–F , 63M , 65M

##### Type material.

***Holotype.*** Madagascar. • 1 major worker; Fianarantsoa; Forêt d’Atsirakambiaty, 7.6 km 285°WNW Itremo; -20.59333, 46.56333; alt. 1550 m; 22 Jan 2003; Fisher et al. leg.; montane rainforest, ex rotten log; BLF07262; CASENT0491605, bottom specimen on the pin (CASC). ***Paratypes.*** • 3w., 7s.; same data as for holotype, CASENT0491603, CASENT0491604, CASENT0872253, CASENT0491601 (CASC, MHNG, PBZT).

##### Other material.

Madagascar. –**Fianarantsoa**: • 3w., 3s.; Forêt d’Atsirakambiaty, 7.6 km 285°WNW Itremo; -20.59333, 46.56333; alt. 1550 m; 22 Jan 2003; Fisher et al. leg.; montane rainforest, ex rotten log; BLF07170 (CASC). • 1w.; Forêt d’Atsirakambiaty, 7.6 km 285°WNW Itremo; -20.59333, 46.56333; alt. 1550 m; 22 Jan 2003; Fisher et al. leg.; montane rainforest, ex rotten log; BLF07182 (CASC). • 3w.; Forêt d’Atsirakambiaty, 7.6 km 285°WNW Itremo; -20.59333, 46.56333; alt. 1550 m; 22 Jan 2003; Fisher et al. leg.; montane rainforest, under stone; BLF07184 (CASC). • 1w.; Forêt d’Atsirakambiaty, 7.6 km 285°WNW Itremo; -20.59333, 46.56333; alt. 1550 m; 22 Jan 2003; Fisher et al. leg.; montane rainforest, ex rotten log; BLF07249 (CASC). • 3w.; Forêt d’Atsirakambiaty, 7.6 km 285°WNW Itremo; -20.59333, 46.56333; alt. 1550 m; 22 Jan 2003; Fisher et al. leg.; montane rainforest, ex rotten log; BLF07250 (CASC).

##### Diagnosis.

Moderately large species. ***Major workers.*** Head in full-face view sub-oval, not widening posteriorly, with anterior and posterior sides relatively straight, in lateral view sub-oval; ventral and dorsal faces convex; sides of the head with dense, moderately long, suberect to erect pilosity; medial part of frons with thick, longitudinal, and dense rugae, interspaces predominantly smooth to indistinctly rugulate, rugae more irregular and directed slightly outward on posteromedial part; lateral sides with thick, dense, and longitudinal rugae with sparsely rugulate interspaces, rugae more irregular on posterolateral parts; occipital lobes without smooth notches; area posterolateral from eyes with sculpture weakening posteriorly and smooth notches; scape, when laid back, exceeding the midlength of head by two-fifths of its length; inner hypostomal teeth distinct, small, closely spaced, triangular, with rounded apex directed upward; outer hypostomal teeth lobe-like, wider than inner teeth, and approximately the same height; inner and outer hypostomal teeth closely spaced and not connected by concavity; mesosoma foveolate; promesonotum with additional thin, moderately sparse, transverse to irregular rugae; katepisternum with smooth notch; gaster smooth; body orange. ***Minor workers.*** Head smooth; lateral sides of frons with longitudinal, short, and thick rugae; vertex with very short, sparse, and transverse to arcing rugae; scape, when laid back, exceeding the posterior head margin by two-fifths of its length; promesonotum moderately low and short, arched; promesonotal groove absent; propodeal spines very small, triangular; mesosoma smooth; dorsal promesonotum with very sparse and irregular rugae; propodeum with sparse, irregular, and indistinct rugulae; body yellow.

##### Description.

**Major workers.** Measurements (*N* = 8): HL: 0.94–1.13 (1.06); HW: 0.97–1.13 (1.06); SL: 0.66–0.72 (0.68); EL: 0.12–0.14 (0.13); WL: 1.05–1.13 (1.08); PSL: 0.15–0.19 (0.17); MTL: 0.65–0.7 (0.67); PNW: 0.52–0.58 (0.55); PTW: 0.14–0.17 (0.15); PPW: 0.29–0.34 (0.31); CI: 97.0–101.9 (99.6); SI: 59.5–69.1 (64.5); PSLI: 13.7–19.3 (16.0); PPI: 46.5–53.6 (50.1); PNI: 48.2–58.5 (52.2); MTI: 59.4–70.8 (63.7).

***Head.*** In full-face view elongate, not widening posteriorly, with anterior and posterior sides relatively straight (Fig. 31B). In lateral view sub-oval; ventral and dorsal faces convex; inner hypostomal teeth visible. Sides of the head with dense, moderately long, suberect to erect pilosity; whole head with dense, long, decumbent to erect pilosity. Medial part of frons with thick, longitudinal, and dense rugae, interspaces predominantly smooth to indistinctly rugulate, rugae more irregular and directed slightly outward on posteromedial part; lateral sides with thick, dense, and longitudinal rugae with sparsely rugulate interspaces, rugae more irregular on posterolateral parts. Occipital lobes with dense, irregular rugae and sparsely rugulate interspaces. Area posterolateral from eyes with weaker and more irregular sculpture, sculpture weakening posteriorly and smooth on the posteriormost part. Gena with relatively sparse and thick longitudinal rugae and distinctly rugulate interspaces. Centre of clypeus smooth and shiny, lateral sides with indistinct rugulae; median notch present, moderately wide, and shallow; median longitudinal carina absent; lateral longitudinal carinae absent. Scape, when laid back, exceeding the midlength of head by two-fifths of its length; pilosity subdecumbent to erect (Fig. 31B, D). Inner hypostomal teeth distinct, small, closely spaced, triangular, with rounded apex directed upward; outer hypostomal teeth lobe-like, wider than inner teeth and approximately the same height; inner and outer hypostomal teeth closely spaced and not connected by concavity (Fig. 63M). ***Mesosoma.*** In lateral view, promesonotum short, angular, and moderately low, posterior mesonotum moderately steep, mesonotal process indistinct, tubercle-like; promesonotal groove absent; metanotal groove indistinct; propodeal spines short, with wide base and acute apex; humeral area weakly produced (Fig. 31D). Surface shiny and foveolate; promesonotum with additional thin, moderately sparse, transverse to irregular rugae; katepisternum with smooth notch. Pilosity dense, long, and erect (Fig. 31D, F). ***Petiole.*** Shiny with sparse foveolae; node finely foveolate, triangular, with rounded and thick apex, in rear view node dorsoventrally convex; pilosity moderately sparse and erect (Fig. 31D, F). ***Postpetiole.*** Shiny and foveolate; dorsum smooth; in dorsal view oval, lateral margins medially with two dentate projections; pilosity long, moderately sparse, and erect (Fig. 31D, F). ***Gaster.*** Shiny and smooth; pilosity very dense, moderately long, and erect (Fig. 31D, F). ***Colour.*** Orange with yellow legs (Fig. 31D, F).

**Minor workers.** Measurements (*N* = 3): HL: 0.61–0.64 (0.62); HW: 0.53–0.55 (0.54); SL: 0.59–0.63 (0.61); EL: 0.08–0.09 (0.09); WL: 0.78–0.81 (0.79); PSL: 0.08–0.1 (0.08); MTL: 0.52–0.56 (0.54); PNW: 0.37–0.39 (0.38); PTW: 0.09–0.1 (0.09); PPW: 0.16–0.16 (0.16); CI: 113.3–117.9 (115.4); SI: 111.9–115.5 (113.1); PSLI: 12.2–15.4 (13.6); PPI: 56.1–61.0 (58.5); PNI: 70.1–70.7 (70.4); MTI: 98.5–101.4 (99.6).

***Head.*** Cephalic margin slightly concave (Fig. 31A). Pilosity relatively dense, moderately long, decumbent to subdecumbent. Sculpture shiny and smooth; lateral sides of frons with longitudinal, short and thick rugae; vertex with very short, sparse, and transverse to arcing rugae; antennal sockets with few thick, curved outward rugae and smooth interspaces. Clypeus with median longitudinal carina absent; two lateral longitudinal carinae absent. Scape, when laid back, exceeding the posterior head margin by two-fifths of its length; pilosity dense, suberect to erect (Fig. 31A, C). ***Mesosoma.*** In lateral view, promesonotum moderately low and short, arched; promesonotal groove absent; metanotal groove distinct; propodeal spines very small, triangular (Fig. 31C). Sculpture shiny and smooth; dorsal promesonotum with very sparse and irregular rugae; propodeum with sparse, irregular, and indistinct rugulae. Pilosity sparse, moderately long, and erect (Fig. 31C, E). ***Gaster.*** With moderately dense, erect pilosity (Fig. 31C, E). ***Colour.*** Yellow (Fig. 31C, E).

##### Etymology.

Malagasy for orange in reference to body colouration.

##### Biology.

The species was collected at 1550 m in elevation, in montane rainforest. Nests were located in rotten logs and under stones.

##### Comments.

*Pheidole haboka* sp. nov., described from Forêt d’Atsirakambiaty in Fianarantsoa, is most similar to *P. sparsa* sp. nov. and *P. dasos* sp. nov., both known from the northern part of the island, and *P. hazo* sp. nov., recorded so far only from the vicinity of Andranoma in Antananarivo. Majors of *P. haboka* sp. nov. can be easily distinguished from all taxa mentioned above by area posterolateral from eyes with sculpture weakening posteriorly and smooth notches and very dense pilosity on gaster; minors can be separated based on body sculpture predominantly smooth and lacking foveolae.

#### Pheidole havoana sp. nov.

Taxon classificationAnimalia

C6B109B1-3C44-5A4B-AA2C-BDC5CD3F17EE

http://zoobank.org/5C216771-3A0C-4352-8EEB-BC0816C6CFF5

**Figure 32.**
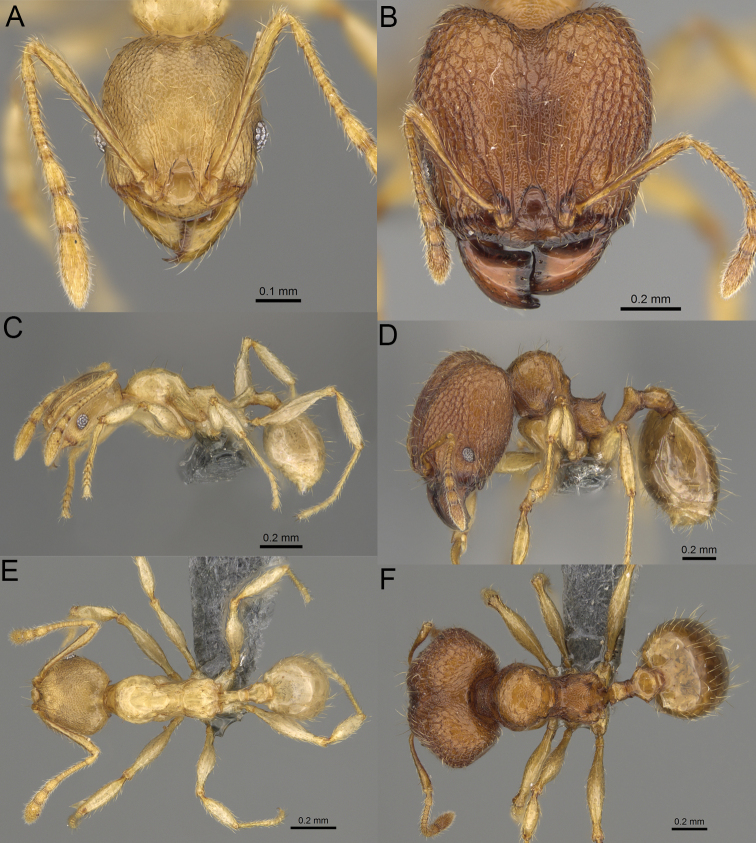
*Pheidole havoana* sp. nov., full-face view (**A**), profile (**C**), and dorsal view (**E**) of paratype minor worker (CASENT0923290) and full-face view (**B**), profile (**D**), and dorsal view (**F**) of holotype major worker (CASENT0704280).

Figs 32A–F , 63N , 65N

##### Type material.

***Holotype.*** Madagascar. • 1 major worker; Toliara; Anosy Region, Anosyenne Mts, 31.2 km NW Manantenina; -24.13894, 47.06804; alt. 1125 m; 26 Feb 2015; B. L. Fisher et al. leg.; rainforest, ex root mat; BLF36492; CASENT0704280 (CASC). ***Paratypes.*** • 2w., 1m.; same data as for holotype; CASENT0923290, CASENT0704279 (CASC, MHNG).

##### Other material.

Madagascar. –**Toliara**: • 2w., 1s., 1m.; Anosy Region, Anosyenne Mts, 31.2 km NW Manantenina; -24.13894, 47.06804; alt. 1125 m; 26 Feb 2015; B. L. Fisher et al. leg.; rainforest, under stone; BLF36463 (CASC). • 2w.,1s., 1q.; Anosy Region, Anosyenne Mts, 31.2 km NW Manantenina; -24.13894, 47.06804; alt. 1125 m; 26 Feb 2015; B. L. Fisher et al. leg.; rainforest, under root mat on rock; BLF36477 (CASC). •2 w., 1s., 1m.; Anosy Region, Anosyenne Mts, 31.2 km NW Manantenina; -24.13894, 47.06804; alt. 1125 m; 26 Feb 2015; B. L. Fisher et al. leg.; rainforest, ex root mat; BLF36496 (CASC). • 1w., 1s.; Anosy Region, Anosyenne Mts, 31.2 km NW Manantenina; -24.13894, 47.06804; alt. 1125 m; 26 Feb 2015; B. L. Fisher et al. leg.; rainforest, ex rotten log; BLF36561 (CASC).

##### Diagnosis.

Minute species. ***Major workers.***HL < 1.1 mm and WL < 0.9 mm; head, in full-face view, sub-oval, slightly widening posteriorly, with anterior and posterior sides convex, in lateral view sub-oval; ventral and dorsal faces convex; body dark orange; sides of head with moderately dense, moderately long, subdecumbent to suberect pilosity; entire head distinctly sculptured, medial part of frons with longitudinal rugae, rugae in posteromedial part more irregular, interspaces shiny with dense, distinct, and irregular rugulae; scape, when laid back, slightly exceeding the midlength of head; mesosoma predominantly rugofoveolate, pronotal dorsum and lateral sides of propodeum with reduced sculpture; inner hypostomal teeth distinct, moderately high, closely spaced, triangular, with rounded apex directed upward; outer hypostomal teeth lobe-like, wider and higher than inner hypostomal teeth, apex directed outward; inner and outer hypostomal teeth closely spaced, connected by indistinct concavity; base of first gastral tergite smooth. ***Minor workers.***HL < 0.5 mm and WL < 0.6 mm, scape, when laid back, surpassing the posterior head margin by one-fifth of its length; propodeal spines very small, triangular; head relatively rectangular; body yellow; head foveolate, frons with additional indistinct longitudinal and interrupted rugae in medial part, area posterolateral from eyes smooth; mesosoma smooth.

##### Description.

**Major workers.** Measurements (*N* = 4): HL: 0.99–1.06 (1.02); HW: 1.0–1.05 (1.0); SL: 0.55–0.56 (0.56); EL: 0.12–0.12 (0.12); WL: 0.85–0.91 (0.88); PSL: 0.18–0.2 (0.19); MTL: 0.53–0.55 (0.54); PNW: 0.46–0.48 (0.47); PTW: 0.11–0.16 (0.14); PPW: 0.24–0.31 (0.27); CI: 98.7–103.1 (101.2); SI: 53.4–56.7 (55.2); PSLI: 17.1–20.5 (18.6); PPI: 40.6–60.4 (52.2); PNI: 45.3–48.9 (46.3); MTI: 52.3–56.0 (53.7).

***Head.*** In full-face view sub-oval, slightly widening posteriorly, with anterior and posterior sides convex (Fig. 32B). In lateral view sub-oval; ventral and dorsal faces convex; inner hypostomal teeth visible. Sides of the head with moderately dense, moderately long, subdecumbent to suberect pilosity; whole head with dense, long, decumbent to erect pilosity. Medial part of frons with moderately dense, thick, longitudinal rugae, rugae in posteromedial part more irregular and directed outward, interspaces shiny with dense and distinct irregular rugulae; lateral sides with longitudinal to irregular and thick rugae with distinctly rugofoveolate interspaces. Occipital lobes with sparse, thick, and irregular rugae; interspaces smooth to indistinctly rugulate. Gena with relatively dense and thick longitudinal rugae and distinctly rugofoveolate interspaces. Area posterolateral from eyes with dense and thick rugofoveolae. Centre of clypeus smooth and shiny, lateral sides with indistinct rugulae; median notch present, moderately wide, and shallow; median longitudinal carina present; lateral longitudinal carinae absent. Scape, when laid back, slightly exceeding the midlength of head; pilosity decumbent to erect (Fig. 32B, D). Inner hypostomal teeth distinct, moderately high, closely spaced, triangular, with rounded apex directed upward; outer hypostomal teeth lobe-like, wider and higher than inner hypostomal teeth, apex directed outward; inner and outer hypostomal teeth closely spaced, connected by indistinct concavity (Fig. 63N). ***Mesosoma.*** In lateral view, promesonotum short, angular, and moderately high, posterior mesonotum moderately steep, mesonotal process indistinct, tubercle-like; promesonotal groove absent; metanotal groove absent; propodeal spines moderately long, moderately wide, with acute apex; humeral area laterally weakly produced (Fig. 32D). Surface shiny and predominantly rugofoveolate, pronotal dorsum and lateral sides of propodeum with reduced sculpture. Pilosity relatively dense, long, and erect (Fig. 32D, F). ***Petiole.*** Shiny with fine and dense rugofoveolae; node partially smooth, low, triangular, with rounded and thin apex, in rear view node dorsoventrally slightly concave; pilosity moderately sparse and erect (Fig. 32D, F). ***Postpetiole.*** Shiny and smooth; in dorsal view oval, lateral margins medially with two dentate projections; pilosity long, moderately sparse, and erect (Fig. 32D, F). ***Gaster.*** Shiny and smooth; pilosity moderately dense, long, and erect (Fig. 32D, F). ***Colour.*** Dark orange; legs yellow (Fig. 32D, F).

**Minor workers.** Measurements (*N* = 5): HL: 0.52–0.53 (0.52); HW: 0.44–0.47 (0.45); SL: 0.51–0.53 (0.52); EL: 0.09–0.11 (0.09); WL: 0.6–0.62 (0.61); PSL: 0.05–0.08 (0.07); MTL: 0.39–0.41 (0.4); PNW: 0.28–0.33 (0.3); PTW: 0.06–0.09 (0.07); PPW: 0.12–0.13 (0.12); CI: 112.2–118.6 (115.6); SI: 112.2–115.8 (114.1); PSLI: 10.3–14.3 (12.9); PPI: 50.4–70.5 (58.2); PNI: 63.4–69.3 (66.7); MTI: 87.2–90.2 (88.4).

***Head.*** Cephalic margin indistinctly concave or straight (Fig. 32A). Pilosity relatively sparse, long, decumbent to subdecumbent. Sculpture foveolate; frons with additional indistinct longitudinal and interrupted rugae in medial part; area posterolateral from eyes smooth. Clypeus with median longitudinal carina absent; two lateral longitudinal carinae absent. Scape, when laid back, surpassing the posterior head margin by one-fifth of its length; pilosity dense, subdecumbent to erect (Fig. 32A, C). ***Mesosoma.*** In lateral view, promesonotum moderately low and short, arched; promesonotal groove absent; metanotal groove present and distinct; propodeal spines very small and triangular (Fig. 32C). Sculpture smooth. Pilosity very sparse, moderately long, and erect (Fig. 32C, E). ***Postpetiole.*** Short, low, and relatively flat; with few short, erect setae (Fig. 32C, E). ***Gaster.*** With sparse, erect pilosity (Fig. 32C, E). ***Colour.*** Yellow, vertex slightly darker (Fig. 32C, E).

##### Etymology.

Malagasy for hill or mountain in reference to type locality.

##### Biology.

The species was collected at 1125 m in elevation, in rainforest. Nests were located in rotten logs under stone and in root mats.

##### Comments.

*Pheidole havoana* sp. nov. belongs to the group of species characterised by small body size (major workers: HL < 1.05 mm, WL < 0.9 mm and minor workers HL < 0.5 mm, WL < 0.6 mm), head sub-oval, slightly widening posteriorly with anterior and posterior sides convex in major workers, and minor workers with yellow to brown body colouration and head foveolate or predominantly smooth and relatively oval. The group includes six species: *P. havoana* sp. nov., *P. kely* sp. nov., *P. parvula* sp. nov., *P. parvulogibba* sp. nov., *P. volontany* sp. nov., and *P. midongy* sp. nov. Within this group, *P. havoana* sp. nov., described form Anosyenne Mts. in Toliara, is most similar to *P. kely* sp. nov., distributed in the northern part of the island, *P. parvula* sp. nov., known from two localities in the vicinity of Antananarivo, and sympatric *P. parvulogibba* sp. nov. Minor workers of *P. havoana* sp. nov. distinctly differ from those of *P. parvula* sp. nov. and *P. parvulogibba* sp. nov. by predominantly foveolate head and presence of additional indistinct longitudinal and interrupted rugae in medial part of frons. Minor workers of *P. havoana* sp. nov. and *P. kely* sp. nov. are indistinguishable. Major workers of *P. havoana* sp. nov. differ from *P. kely* sp. nov. and *P. parvulogibba* sp. nov. by medial part of frons with longitudinal rugae, rugae in posteromedial part more irregular, with densely and distinctly irregular rugulae in interspaces; and from *P. parvula* sp. nov. in reduced sculpture on pronotal dorsum and lateral sides of propodeum.

#### Pheidole hazo sp. nov.

Taxon classificationAnimalia

8A4178BF-D3C4-5D99-B1E6-77B0BB43D178

http://zoobank.org/A8CA690D-98D2-4DC9-8B8A-CB60BE22F0ED

**Figure 33.**
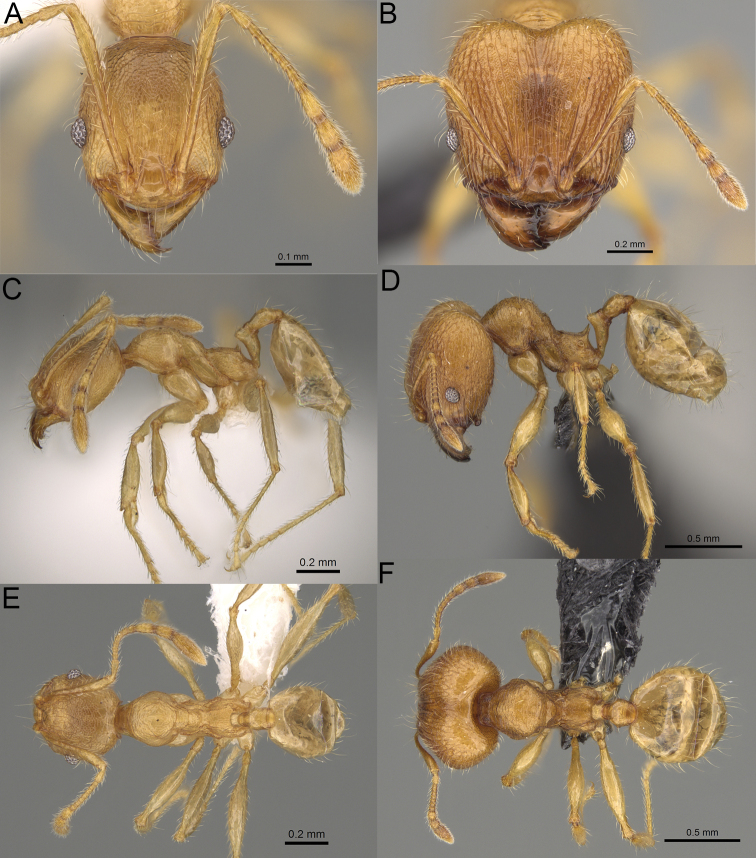
*Pheidole hazo* sp. nov., full-face view (**A**), profile (**C**), and dorsal view (**E**) of paratype minor worker (CASENT0427762) and full-face view (**B**), profile (**D**), and dorsal view (**F**) of holotype major worker (CASENT0427790).

Figs 33A–F , 63O , 65O

##### Type material.

***Holotype.*** Madagascar. • 1 major worker; Antananarivo; 3 km 41°NE Andranomay, 11.5 km 147°SSE Anjozorobe; -18.47333, 47.96; alt. 1300 m; 5 Dec 2000; Fisher et al. leg.; montane rainforest, ex dead branch above ground; BLF02441; CASENT0427790, bottom specimen on the pin (CASC). ***Paratypes.*** • 5w., 8s.; same data as for holotype; CASENT0427762, CASENT00872251, CASENT0413711, CASENT0413712, CASENT0427765 (CASC, MHNG, PBZT).

##### Diagnosis.

Moderately large species. ***Major workers.*** Head in full-face view sub-oval, not widening posteriorly, with anterior and posterior sides relatively straight, in lateral view sub-oval; ventral and dorsal faces convex; sides of the head with dense, long, suberect to erect pilosity; medial part of frons with moderately dense, thin, longitudinal rugae, rugae in posteromedial portion directed slightly outward, interspaces shiny with sparse and distinct rugofoveolae; lateral sides with longitudinal, thicker, and relatively sparser rugae; interspaces shiny with relatively dense and distinct rugofoveolae; occipital lobes, and area posterolateral from eyes without smooth notches; scape, when laid back, exceeding the midlength of head by two-fifths of its length; inner hypostomal teeth distinct, large, closely spaced, triangular, with rounded apex directed slightly outward; outer hypostomal teeth lobe-like, wider than inner hypostomal teeth and approximately the same height, apex directed outward; inner and outer hypostomal teeth closely spaced and connected by concavity; mesosoma with thick and sparse foveolae; pronotum with additional transverse to irregular, thin rugae; katepisternum with smooth notch; gaster smooth; body orange. ***Minor workers.*** Head foveolate with additional longitudinal to irregular, interrupted, and moderately thick rugae; area posterolateral from eyes with weaker foveolae and smooth notches; scape, when laid back, surpassing the posterior head margin by one-fifth of its length; promesonotum moderately low and moderately long, arched; promesonotal groove absent; propodeal spines minute and triangular; mesosoma with thick foveolae; lateral sides of propodeum, katepisternum, and anepisternum with smooth notches; body yellow.

##### Description.

**Major workers.** Measurements (*N* = 9): HL: 1.06–1.14 (1.09); HW: 1.02–1.13 (1.08); SL: 0.63–0.66 (0.64); EL: 0.14–0.17 (0.15); WL: 0.98–1.08 (1.03); PSL: 0.16–0.18 (0.17); MTL: 0.63–0.65 (0.64); PNW: 0.41–0.52 (0.47); PTW: 0.13–0.15 (0.14); PPW: 0.25–0.3 (0.27); CI: 97.6–104.3 (101.0); SI: 56.4–63.3 (59.7); PSLI: 15.1–17.1 (16.0); PPI: 47.3–55.8 (51.5); PNI: 39.2–47.0 (43.3); MTI: 57.1–63.9 (59.9).

***Head.*** In full-face view sub-oval, not widening posteriorly, with anterior and posterior sides relatively straight (Fig. 33B). In lateral view sub-oval; ventral and dorsal faces convex; inner hypostomal teeth visible. Sides of the head with dense, long, suberect to erect pilosity; whole head with dense, long, decumbent to erect pilosity. Medial part of frons with moderately dense, thin, longitudinal rugae, rugae in posteromedial directed slightly outward, interspaces shiny with sparse and distinct rugofoveolae; lateral sides with longitudinal, thicker, and relatively sparser rugae; interspaces shiny with relatively dense and distinct rugofoveolae. Occipital lobes with sparse, thick, and longitudinal to irregular rugae; interspaces rugofoveolate. Gena with relatively dense and thick, longitudinal rugae and rugofoveolate interspaces. Area posterolateral from eyes shiny, with dense, longitudinal rugofoveolae. Centre of clypeus smooth and shiny, lateral sides with indistinct rugulae; median notch present, moderately wide, and shallow; median longitudinal carina absent; lateral longitudinal carinae absent. Scape, when laid back, exceeding the midlength of head by two-fifths of its length; pilosity subdecumbent to erect (Fig. 33B, D). Inner hypostomal teeth distinct, large, closely spaced, triangular, with rounded apex directed slightly outward; outer hypostomal teeth lobe-like, wider than inner hypostomal teeth and approximately the same height, apex directed outward; inner and outer hypostomal teeth closely spaced and connected by concavity (Fig. 63O). ***Mesosoma.*** In lateral view, promesonotum short, angular, and moderately low, posterior mesonotum moderately steep, mesonotal process indistinct, tubercle-like; promesonotal groove absent; metanotal groove absent; propodeal spines moderately long, moderately narrow, with acute apex; humeral area laterally weakly produced (Fig. 33D). Surface shiny with thick and sparse foveolae; pronotum with additional transverse to irregular, thin rugae; katepisternum with smooth notch. Pilosity sparse, long, and erect (Fig. 33D, F). ***Petiole.*** Shiny with fine and sparse foveolae; node smooth to finely foveolate, low, triangular, with rounded and thin apex, in rear view node dorsoventrally straight to slightly convex; pilosity moderately sparse and erect (Fig. 33D, F). ***Postpetiole.*** Shiny and smooth; in dorsal view oval, lateral margins medially with two dentate projections; pilosity long, moderately sparse, and erect (Fig. 33D, F). ***Gaster.*** Shiny and smooth; pilosity moderately dense, long, and erect (Fig. 33D, F). ***Colour.*** Dark orange; mandibles slightly darker; legs yellow (Fig. 33D, F).

**Minor workers.** Measurements (*N* = 5): HL: 0.6–0.63 (0.61); HW: 0.5–0.53 (0.52); SL: 0.61–0.64 (0.62); EL: 0.11–0.12 (0.12); WL: 0.76–0.82 (0.79); PSL: 0.08–0.09 (0.09); MTL: 0.52–0.55 (0.54); PNW: 0.33–0.35 (0.34); PTW: 0.09–0.1 (0.09); PPW: 0.14–0.15 (0.15); CI: 114.8–121.9 (118.8); SI: 115.4–123.9 (120.9); PSLI: 12.9–14.6 (13.9); PPI: 57.3–70.4 (62.7); PNI: 63.4–67.4 (66.0); MTI: 101.5–106.2 (103.8).

***Head.*** Cephalic margin indistinctly convex or straight (Fig. 33A). Pilosity relatively sparse, long, decumbent to suberect. Sculpture foveolate with additional longitudinal to irregular, interrupted, and moderately thick rugae; area posterolateral from eyes with weaker foveolae and smooth notches. Clypeus with median longitudinal carina absent; two lateral longitudinal carinae absent. Scape, when laid back, surpassing the posterior head margin by one-fifth of its length; pilosity dense, subdecumbent to erect (Fig. 33A, C). ***Mesosoma.*** In lateral view, promesonotum moderately low and moderately long, arched; promesonotal groove absent; metanotal groove distinct; propodeal spines minute and triangular (Fig. 33C). Sculpture shiny and with thick foveolae; lateral sides of propodeum, katepisternum, and anepisternum with smooth notches. Pilosity very sparse, moderately long, and erect (Fig. 33C, E). ***Postpetiole.*** Short, low, and relatively flat; with few short, erect setae (Fig. 33C, E). ***Gaster.*** With sparse, erect pilosity (Fig. 33C, E). ***Colour.*** Yellow, vertex slightly darker (Fig. 33C, E).

##### Etymology.

Malagasy for tree in reference to arboreal nesting preferences of the species.

##### Biology.

The species was collected at 1300 m in elevation, in montane rainforest. Nest was located in dead branch above ground.

##### Comments.

*Pheidole hazo* sp. nov., described from the vicinity of Andranoma in Antananarivo, is most similar to *P. dasos* sp. nov., known from Makirovana forest in Antsiranana, and *P. sparsa* sp. nov., recorded so far only from Bemanevika in Mahajanga. Majors of *P. hazo* sp. nov. can be distinguished from both taxa by medial part of frons with moderately dense rugae with interspaces with distinct rugofoveolae, and pronotum lacking smooth notches; minors of *P. hazo* sp. nov. can be distinguished from *P. sparsa* sp. nov. by yellow body colouration, and mesosoma predominantly with dense foveolae, and from *P. dasos* sp. nov. by yellow body colouration, shorter scape surpassing the posterior head margin by one-fifth of its length, and pronotum with distinct and dense foveolae.

#### Pheidole itremo sp. nov.

Taxon classificationAnimalia

333BE617-A4F5-57CA-8050-326F1FBCB9F6

http://zoobank.org/3E990650-E613-475B-8C4B-E16C43898146

**Figure 34.**
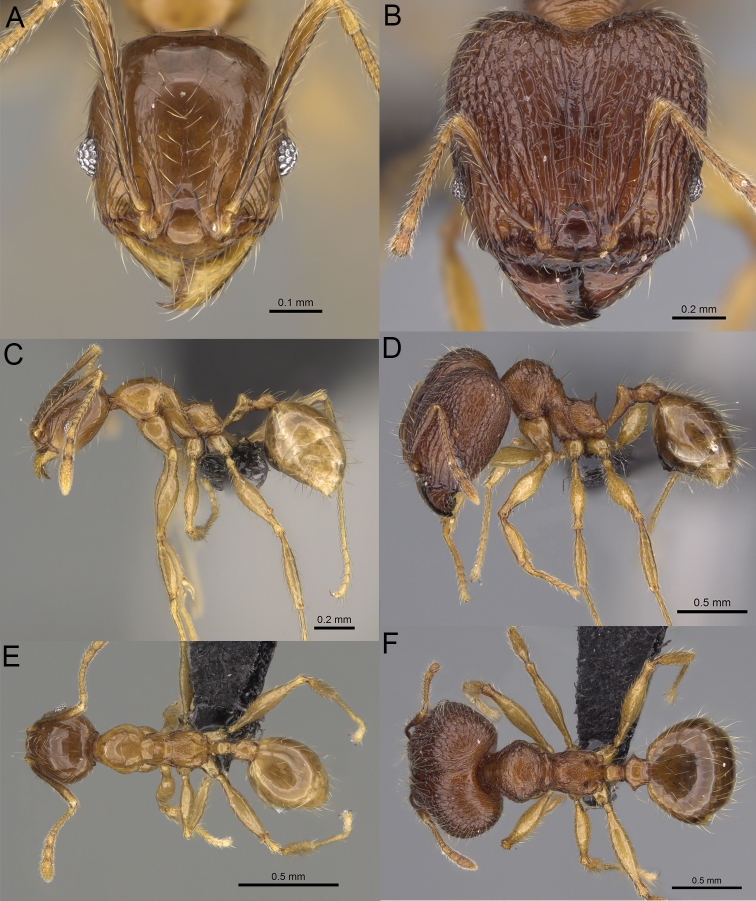
*Pheidole itremo* sp. nov., full-face view (**A**), profile (**C**), and dorsal view (**E**) of paratype minor worker (CASENT0491586) and full-face view (**B**), profile (**D**), and dorsal view (**F**) of holotype major worker (CASENT0491584).

Figs 34A–F , 63P , 65P

##### Type material.

***Holotype.*** Madagascar. • 1 major worker; Fianarantsoa; Forêt d’Atsirakambiaty, 7.6 km 285°WNW Itremo; -20.59333, 46.56333; alt. 1550 m; 22 Jan 2003; Fisher et al. leg.; montane rainforest, ex rotten log; BLF07251; CASENT0491584, top specimen on the pin (CASC). ***Paratypes.*** • 6w., 1s.; same data as for holotype, CASENT0872249, CASENT0491586, CASENT0491585 (CASC, MHNG, PBZT).

##### Other material.

Madagascar. –**Antananarivo**: • 1w., 2s.; Réserve Spéciale d’Ambohitantely, Forêt d Ambohitantely, 20.9 km 72°NE d Ankazobe; -18.22528, 47.28683; alt. 1410 m; 17 Apr 2001; Fisher et al. leg.; montane rainforest, ex root mat, ground layer; BLF03700 (CASC). • 3s.; Réserve Spéciale d’Ambohitantely, Forêt d Ambohitantely, Jardin Botanique, 24.1km 59°NE d Ankazobe; -18.17139, 47.28182; alt. 1620 m; 17 Apr 2001; Fisher et al. leg.; montane rainforest, sifted litter; BLF03720 (CASC). • 2w.; Réserve Speciale d’Ambohitantely; -18.18762, 47.28576; alt. 1580 m; 8 Mar 2012; Fisher et al. leg.; montane forest, ex rotten log; BLF28200 (CASC). • 3w., 1q.; Réserve Speciale d’Ambohitantely; -18.18762, 47.28576; alt. 1580 m; 8 Mar 2012; Fisher et al. leg.; montane forest, ex rotten log; BLF28206 (CASC). • 2w.; Réserve Speciale d’Ambohitantely; -18.18762, 47.28576; alt. 1580 m; 8 Mar 2012; Fisher et al. leg.; montane forest, ex rotten log; BLF28218 (CASC). • 3w., 1q.; Réserve Speciale d’Ambohitantely; -18.18762, 47.28576; alt. 1580 m; 8 Mar 2012; Fisher et al. leg.; montane forest, ex rotten log; BLF28227 (CASC).

##### Diagnosis.

Moderately large species. ***Major workers.*** Head in full-face view sub-oval, widening posteriorly, with anterior and posterior sides convex, in lateral view sub-oval; ventral and dorsal faces convex; sides of the head with moderately dense, moderately long, suberect to erect pilosity; medial part of frons with thick, dense, longitudinal, and interrupted rugae and smooth interspaces, posteromedial frons with more irregular rugae directed slightly outward; lateral sides with thick and dense rugae, interspaces shiny and smooth or indistinctly rugulate; rugae longitudinal on anterolateral sides and more irregular on posterolateral sides; area posterolateral from eyes without smooth notches; scape, when laid back, exceeding the midlength of head by one-fifth of its length; inner hypostomal teeth distinct, large, closely spaced, triangular, with rounded apex directed upward; outer hypostomal teeth lobe-like, wider than inner teeth and approximately the same height; inner and outer hypostomal teeth closely spaced and connected by indistinct concavity; mesosoma with fine rugofoveolae; pronotum with rugofoveolae sparser and additional thin and transverse rugae; gaster smooth; body brown. ***Minor workers.*** Head smooth; posterolateral sides of frons with sparse and indistinct foveolae; scape, when laid back, surpassing the posterior head margin by two-fifths of its length; promesonotum moderately high and short, arched; promesonotal groove absent; propodeal spines minute and triangular; mesosoma smooth and shiny; body yellowish brown.

##### Description.

**Major workers.** Measurements (*N* = 6): HL: 1.14–1.24 (1.18); HW: 1.16–1.21 (1.17); SL: 0.64–0.69 (0.66); EL: 0.12–0.14 (0.13); WL: 0.99–1.06 (1.03); PSL: 0.18–0.2 (0.19); MTL: 0.65–0.68 (0.66); PNW: 0.51–0.54 (0.52); PTW: 0.15–0.17 (0.16); PPW: 0.28–0.34 (0.31); CI: 98.3–103.3 (100.8); SI: 53.7–58.8 (56.0); PSLI: 15.5–16.9 (16.1); PPI: 43.0–56.6 (51.5); PNI: 42.7–46.2 (44.3); MTI: 54.6–57.7 (56.6).

***Head.*** In full-face view sub-oval, widening posteriorly, with anterior and posterior sides convex (Fig. 34B). In lateral view sub-oval; ventral and dorsal faces convex; inner hypostomal teeth visible. Sides of the head with moderately dense, moderately long, suberect to erect pilosity; whole head with dense, long, decumbent to erect pilosity. Medial part of frons with thick, dense, longitudinal, and interrupted rugae and smooth interspaces, posteromedial frons with rugae more irregular and directed slightly outward; lateral sides with thick and dense rugae, interspaces shiny and smooth or indistinctly rugulate; rugae longitudinal on anterolateral sides and more irregular on posterolateral sides. Occipital lobes with thinner, irregular rugae and smooth to indistinctly rugoreticulate interspaces. Area posterolateral from eyes with irregular to longitudinal, relatively thick to thin rugulae and rugoreticulate interspaces. Gena with relatively dense, thick, longitudinal rugae and smooth to indistinctly rugoreticulate interspaces. Centre of clypeus smooth and shiny, lateral sides with indistinct rugulae; median notch present, moderately wide, and shallow; median longitudinal carina present; lateral longitudinal carinae absent. Scape, when laid back, exceeding the midlength of head by one-fifth of its length; pilosity subdecumbent to erect (Fig. 34B, D). Inner hypostomal teeth distinct, large, closely spaced, triangular, with rounded apex directed upward; outer hypostomal teeth lobe-like, wider than inner teeth and approximately the same height; inner and outer hypostomal teeth closely spaced and connected by indistinct concavity (Fig. 63P). ***Mesosoma.*** In lateral view, promesonotum short, angular, and moderately low, posterior mesonotum moderately steep, mesonotal process very indistinct, tubercle-like; promesonotal groove absent; metanotal groove absent; propodeal spines moderately long, with moderately narrow base and acute apex; humeral area laterally weakly produced (Fig. 34D). Surface shiny with fine rugofoveolae; pronotum with rugofoveolae sparser and additional thin and transverse rugae. Pilosity moderately sparse, long, and erect (Fig. 34D, F). ***Petiole.*** Shiny with fine foveolae; node smooth to finely foveolate, triangular, with rounded and thick apex, in rear view node dorsoventrally straight to slightly convex; pilosity moderately sparse and erect (Fig. 34D, F). ***Postpetiole.*** Shiny and foveolate; dorsum with reduced sculpture and smooth notch; in dorsal view oval, lateral margins medially with two dentate projections; pilosity long, moderately sparse, and erect (Fig. 34D, F). ***Gaster.*** Shiny and smooth; pilosity dense, long, and erect (Fig. 34D, F). ***Colour.*** Brown; legs yellow (Fig. 34D, F).

**Minor workers.** Measurements (*N* = 10): HL: 0.54–0.59 (0.56); HW: 0.46–0.5 (0.48); SL: 0.59–0.63 (0.6); EL: 0.1–0.13 (0.11); WL: 0.68–0.74 (0.71); PSL: 0.06–0.1 (0.08); MTL: 0.45–0.5 (0.47); PNW: 0.3–0.35 (0.32); PTW: 0.08–0.1 (0.08); PPW: 0.13–0.16 (0.14); CI: 115.7–121.8 (117.9); SI: 120.4–131.4 (125.6); PSLI: 11.8–17.0 (14.0); PPI: 49.4–67.0 (58.5); PNI: 65.8–71.0 (68.2); MTI: 94.5–101.7 (99.2).

***Head.*** Cephalic margin indistinctly convex (Fig. 34A). Pilosity relatively sparse, long, decumbent to suberect. Sculpture shiny and smooth; posterolateral sides of frons with sparse and indistinct foveolae; antennal sockets with few thick, curved outward rugae and smooth interspaces. Clypeus with median longitudinal carina absent; two lateral longitudinal carinae absent. Scape, when laid back, surpassing the posterior head margin by two-fifths of its length; pilosity dense, subdecumbent to erect (Fig. 34A, C). ***Mesosoma.*** In lateral view, promesonotum moderately high and short, arched; promesonotal groove absent; metanotal groove distinct; propodeal spines minute and triangular (Fig. 34C). Sculpture shiny and smooth. Pilosity moderately sparse, long, and erect (Fig. 34C, E). ***Gaster.*** With sparse, erect pilosity (Fig. 34C, E). ***Colour.*** Yellowish brown (Fig. 34C, E).

##### Etymology.

From the type locality.

##### Biology.

The species was collected between 1410–1620 m in elevation, in montane forest and montane rainforest. Nests were located in rotten logs and root mats.

##### Comments.

*Pheidole itremo* sp. nov., known from Forêt d’Atsirakambiaty in Fianarantsoa and Forêt d’Ambohitantely in Antananarivo, is most similar to *P. sava* sp. nov., described from Parc National de Marojejy in Antsiranana. Major workers of *P. itremo* sp. nov. can be separated based on darker body colouration, presence of moderately dense, moderately long, suberect to erect pilosity on sides of the head, medial frons with smooth interspaces, finely rugofoveolae mesosoma lacking smooth notches; minor workers can be separated based on predominantly smooth head with posterolateral sides of frons with sparse and indistinct foveolae, and entirely smooth mesosoma.

#### Pheidole joffreville sp. nov.

Taxon classificationAnimalia

CEE3427E-42F3-52E1-9E83-35E0A879E507

http://zoobank.org/2290CAFF-30DF-4911-8EEC-7AF36C7A79CE

**Figure 35.**
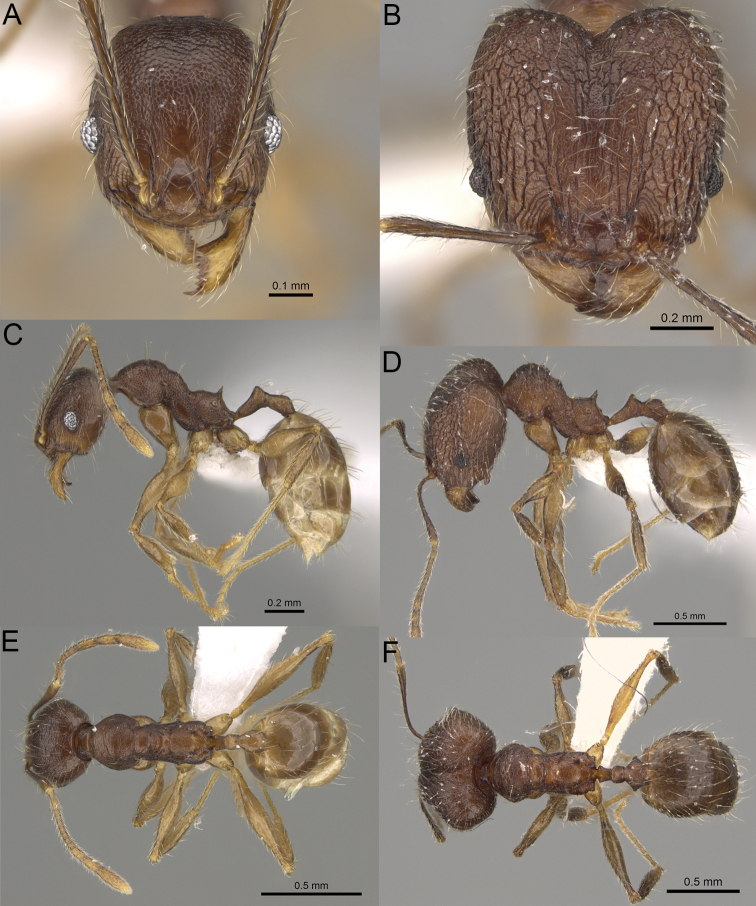
*Pheidole joffreville* sp. nov., full-face view (**A**), profile (**C**), and dorsal view (**E**) of paratype minor worker (CASENT0406352) and full-face view (**B**), profile (**D**), and dorsal view (**F**) of holotype major worker (CASENT0406355).

Figs 35A–F , 63Q , 65Q

##### Type material.

***Holotype.*** Madagascar. • 1 major worker; Antsiranana; Parc National Montagne d’Ambre, 3.6 km 235°SW Joffreville; -12.53444, 49.1795; alt. 925 m; 20 Jan 2001; Fisher et al. leg.; montane rainforest, ex rotten log; BLF02568; CASENT0406355 (CASC). ***Paratypes.*** • 4w., 2s.; same data as for holotype, CASENT0406358, CASENT0406356, CASENT0406352, CASENT0406354, CASENT0406350 (CASC, MHNG).

##### Diagnosis.

Moderately large species. ***Major workers.*** Head in full-face view sub-oval and widening slightly posteriorly, with anterior and posterior sides slightly convex, in lateral view sub-oval; ventral and dorsal faces convex; sides of the head with sparse, moderately long, suberect pilosity; anteromedial part of frons with thick, longitudinal, interrupted, and sparse rugae, posteromedial frons with rugae longitudinally irregular, interspaces distinctly rugofoveolate, rugae on posteromedial part directed outward; lateral sides with thick, sparse, and irregular rugae with distinctly rugofoveolate interspaces; occipital lobes, and area posterolateral from eyes without smooth notches; scape, when laid back, exceeding the midlength of head by one-fifth of its length; inner hypostomal teeth distinct, large, closely spaced, triangular, with rounded apex directed upward; outer hypostomal teeth lobe-like, narrower, and approximately as high as inner teeth; inner and outer hypostomal teeth closely spaced and not connected by concavity; mesosoma foveolate; promesonotum with additional sparse, thick, and transverse rugulae on dorsum; gaster smooth; body brown. ***Minor workers.*** Head foveolate; vertex with a few arcing, sparse, and moderately thick rugae; scape, when laid back, exceeding the posterior head margin by one-third of its length; promesonotum low and moderately long, arched; promesonotal groove present; propodeal spines moderate, triangular; mesosoma foveolate; katepisternum with big smooth notch; body brown.

##### Description.

**Major workers.** Measurements (*N* = 3): HL: 1.04–1.1 (1.06); HW: 1.03–1.16 (1.06); SL: 0.73–0.75 (0.74); EL: 0.14–0.15 (0.14); WL: 0.9–0.93 (0.92); PSL: 0.17–0.19 (0.17); MTL: 0.62–0.67 (0.64); PNW: 0.44–0.47 (0.45); PTW: 0.15–0.15 (0.15); PPW: 0.31–0.34 (0.32); CI: 98.6–101.0 (100.1); SI: 67.3–70.9 (69.3); PSLI: 15.4–17.9 (16.3); PPI: 44.4–47.3 (46.3); PNI: 42.2–43.0 (42.5); MTI: 59.7–60.1 (59.9).

***Head.*** In full-face view sub-oval, widening posteriorly, with anterior and posterior sides convex (Fig. 35B). In lateral view sub-oval; ventral and dorsal faces convex; inner hypostomal teeth visible. Sides of the head with sparse, moderately long, suberect pilosity; whole head with dense, long, decumbent to erect pilosity. Anteromedial part of frons with thick, longitudinal, interrupted and sparse rugae, posteromedial frons with rugae longitudinally irregular, interspaces distinctly rugofoveolate, rugae on posteromedial part directed outward; lateral sides with thick, sparse, and irregular rugae with distinctly rugofoveolate interspaces. Occipital lobes with thinner and denser irregular rugae and rugofoveolate interspaces. Area posterolateral from eyes with dense rugofoveolae and additional sparse rugae. Gena with relatively sparse, thick, longitudinal rugae and distinctly rugofoveolate interspaces. Centre of clypeus smooth and shiny, lateral sides with indistinct rugulae; median notch present, moderately wide, and shallow; median longitudinal carina present; lateral longitudinal carinae absent. Scape, when laid back, exceeding the midlength of head by one-fifth of its length; pilosity subdecumbent to erect (Fig. 35B, D). Inner hypostomal teeth distinct, large, closely spaced, triangular, with rounded apex directed upward; outer hypostomal teeth lobe-like, narrower, and approximately as high as inner teeth; inner and outer hypostomal teeth closely spaced and not connected by concavity (Fig. 63Q). ***Mesosoma.*** In lateral view, promesonotum short, angular, and moderately low, posterior mesonotum moderately steep, mesonotal process indistinct, tubercle-like; promesonotal groove absent; metanotal groove absent; propodeal spines moderate, with wide base and acute apex; humeral area produced (Fig. 35D). Surface shiny and foveolate; promesonotum with additional sparse, thick, and transverse rugulae on dorsum. Pilosity moderately sparse, long, and erect (Fig. 35D, F). ***Petiole.*** Shiny with dense foveolae; node finely foveolate, triangular, with rounded and thick apex, in rear view node dorsoventrally slightly convex; pilosity moderately sparse and erect (Fig. 35D, F). ***Postpetiole.*** Shiny and foveolate; dorsum with reduced sculpture and smooth notch; in dorsal view oval, lateral margins medially with two dentate projections; pilosity long, moderately sparse, and erect (Fig. 35D, F). ***Gaster.*** Shiny and smooth; pilosity moderately sparse, long, and erect (Fig. 35D, F). ***Colour.*** Brown with yellowish legs (Fig. 35D, F).

**Minor workers.** Measurements (*N* = 4): HL: 0.6–0.63 (0.62); HW: 0.52–0.54 (0.53); SL: 0.7–0.74 (0.72); EL: 0.13–0.13 (0.13); WL: 0.74–0.8 (0.76); PSL: 0.11–0.12 (0.11); MTL: 0.53–0.55 (0.54); PNW: 0.35–0.37 (0.36); PTW: 0.09–0.1 (0.09); PPW: 0.14–0.15 (0.15); CI: 116.0–120.5 (117.6); SI: 133.0–138.5 (136.2); PSLI: 17.6–18.9 (18.3); PPI: 58.6–67.8 (63.7); PNI: 66.9–70.2 (68.3); MTI: 99.3–105.6 (102.4).

***Head.*** Cephalic margin slightly convex (Fig. 35A). Pilosity relatively sparse, moderately long, subdecumbent to erect. Sculpture shiny and foveolate; vertex with a few arcing, sparse, and moderately thick rugae; antennal sockets with few thick, curved outward rugae and foveolate interspaces. Clypeus with median longitudinal carina absent; two lateral longitudinal carinae absent. Scape, when laid back, exceeding the posterior head margin by one-third of its length; pilosity dense, subdecumbent to erect (Fig. 35A, C). ***Mesosoma.*** In lateral view, promesonotum low and moderately long, arched; promesonotal groove present; metanotal groove distinct; propodeal spines moderate, triangular (Fig. 35C). Sculpture shiny and foveolate; katepisternum with big smooth notch. Pilosity very sparse, moderately long, and erect (Fig. 35C, E). ***Gaster.*** With sparse, erect pilosity (Fig. 35C, E). ***Colour.*** Brown, legs, antenna and gaster yellowish (Fig. 35C, E).

##### Etymology.

From the type locality.

##### Biology.

The species was collected at 925 m in elevation, in montane rainforest. Nest was located in rotten log.

##### Comments.

*Pheidole joffreville* sp. nov., described from Parc National Montagne d’Ambre in Antsiranana, has major workers with dense and thick rugae, anteromedially longitudinal and posteromedially irregular, with distinctly rugofoveolate interspaces, and brown body colouration. Morphologically is most similar to allopatric *P. mivory* sp. nov., known from Parc National de Marojejy in Antsiranana. Majors of both taxa are extremely similar and species separation should be supported or based exclusively on minors. Majors of *P. joffreville* sp. nov. differ from *P. mivory* sp. nov. in distinctly rugofoveolate interspaces on frons and lack of longitudinal rugae on lateral sides of frons and lack of shagreened sculpture on first gastral tergite; minor workers differ in distinctly foveolate mesosoma with only katepisternum with smooth notch.

#### Pheidole kely sp. nov.

Taxon classificationAnimalia

C55A1B83-3B4A-52E9-9196-1A7F8E08366C

http://zoobank.org/40D3BEC5-AB9B-4891-B58B-BF60B571341B

**Figure 36.**
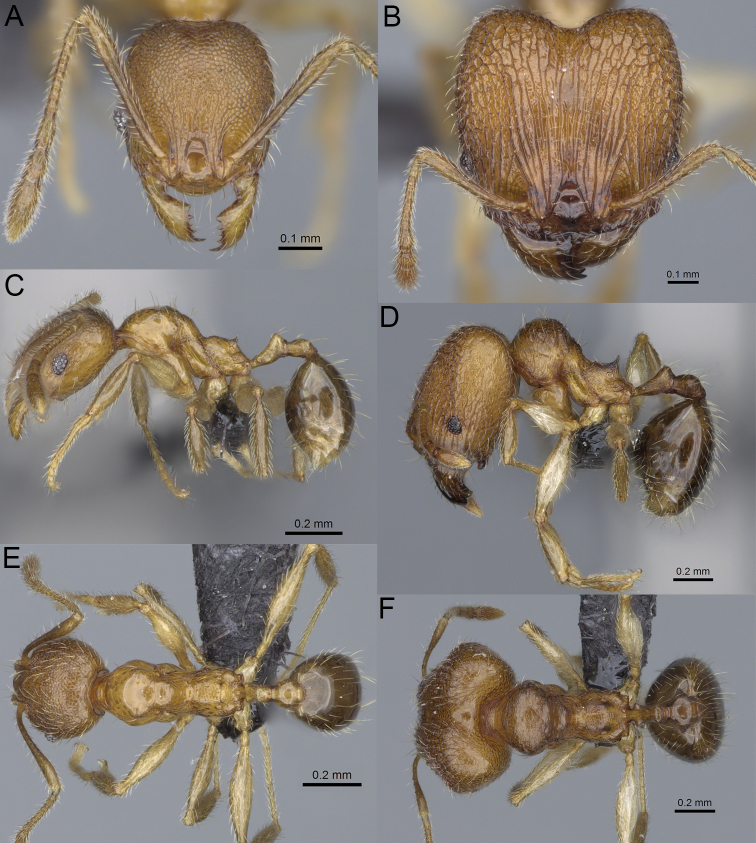
*Pheidole kely* sp. nov., full-face view (**A**), profile (**C**), and dorsal view (**E**) of paratype minor worker (CASENT0149839) and full-face view (**B**), profile (**D**), and dorsal view (**F**) of holotype major worker (CASENT0235022).

Figs 36A–F , 63R , 65R

##### Type material.

***Holotype.*** Madagascar. • 1 major worker; Toamasina; Parc National de Zahamena; -17.73359, 48.72625; alt. 950 m; 19 Feb 2009; B. L. Fisher et al. leg.; rainforest, ex rotten log; BLF22067; CASENT0235022 (CASC). ***Paratype.*** • 1w.; same data as for holotype, CASENT0149839 (CASC).

##### Other material.

Madagascar. –**Antsiranana**: • 2w., 2s.; Betaolana Forest, along Bekona River; -14.52996, 49.44039; alt. 880 m; 4 Mar 2009; B. L. Fisher et al. leg.; rainforest, ex rotten log; BLF22452 (CASC). • 2w., 2q.; Betaolana Forest, along Bekona River; -14.52996, 49.44039; alt. 880 m; 4 Mar 2009; B. L. Fisher et al. leg.; rainforest, ex rotten log; BLF22480 (CASC). • 2w., 2s.; Betaolana Forest, along Bekona River; -14.52996, 49.44039; alt. 880 m; 4 Mar 2009; B. L. Fisher et al. leg.; rainforest, ex rotten log; BLF22559 (CASC). • 1w., 1s.; Betaolana Forest, along Bekona River; -14.52996, 49.44039; alt. 880 m; 5 Mar 2009; B. L. Fisher et al. leg.; rainforest, ex rotten log; BLF22591 (CASC). • 1w., 1s.; Betaolana Forest, along Bekona River; -14.52996, 49.44039; alt. 880 m; 5 Mar 2009; B. L. Fisher et al. leg.; rainforest, ex rotten log; BLF22628 (CASC). • 2w., 2q.; Makirovana forest; -14.17066, 49.95409; alt. 415 m; 29 Apr 2011; B. L. Fisher et al. leg.; rainforest, ex rotten log; BLF26641 (CASC). • 2w.; Makirovana forest; -14.17066, 49.95409; alt. 415 m; 29 Apr 2011; B. L. Fisher et al. leg.; rainforest, ex root mat, ground layer; BLF26670 (CASC). • 2w.; Makirovana forest; -14.16044, 49.95216; alt. 550 m; 1 May 2011; B. L. Fisher et al. leg.; rainforest, under rootmat, litter on rock; BLF26911 (CASC). • 3w., 2s.; Parc National de Marojejy, Manantenina River, 27.6 km 35°NE Andapa, 9.6 km 327°NNW Manantenina; -14.435, 49.76; alt. 775 m; 15 Nov 2003; B. L. Fisher et al. leg.; rainforest, ex rotten log; BLF08904 (CASC). • 3w., 6s.; Parc National de Marojejy, Manantenina River, 27.6 km 35°NE Andapa, 9.6 km 327°NNW Manantenina; -14.435, 49.76; alt. 775 m; 15 Nov 2003; B. L. Fisher et al. leg.; rainforest, ex rotten log; BLF08928 (CASC). • 2w.; Parc National de Marojejy, Manantenina River, 27.6 km 35°NE Andapa, 9.6 km 327°NNW Manantenina; -14.435, 49.76; alt. 775 m; 16 Nov 2003; B. L. Fisher et al. leg.; rainforest, ex rotten log; BLF08943 (CASC). • 2w., 3s.; Parc National de Marojejy, Manantenina River, 28.0 km 38°NE Andapa, 8.2 km 333°NNW Manantenina; -14.43667, 49.775; alt. 450 m; 12 Nov 2003; B. L. Fisher et al. leg.; rainforest, ex rotten log; BLF08749 (CASC). • 3w., 1s., 1q.; Sava Region: Parc National de Marojejy, Manantenina River, 28.0 km 24.8°NE Andapa; -14.43461, 49.76074; alt. 780 m; 8 Feb 2018; B. L. Fisher et al. leg.; rainforest, ex rotten log; BLF40819 (CASC). • 3w., 1s., 1m.; Sava Region: Parc National de Marojejy, Manantenina River, 28.0 km 24.8°NE Andapa; -14.43461, 49.76074; alt. 780 m; 13 Feb 2018; B. L. Fisher et al. leg.; rainforest, ex rotten log; BLF41152 (CASC). • 2w., 1s., 1q.; Sava Region: Parc National de Marojejy, near Manantenina River; -14.43677, 49.77541; alt. 475 m; 7 Feb 2018; B. L. Fisher et al. leg.; rainforest, ex rotten log; BLF40761 (CASC).–**Fianarantsoa**: • 2w.; Parc National d’Isalo, Ambovo Springs, 29.3 km 4°N Ranohira; -22.29833, 45.35167; alt. 990 m; 9 Feb 2003; B. L. Fisher et al. leg.; Uapaca woodland, under stone; BLF07796 (CASC).–**Toamasina**: • 3w.; Montagne d’Akirindro 7.6 km 341°NNW Ambinanitelo; -15.28833, 49.54833; alt. 600 m; 17 Mar 2003; B. L. Fisher et al. leg.; rainforest, ex rotten log; BLF08293 (CASC). • 1w., 1s.; Parc National de Zahamena; -17.73359, 48.72625; alt. 950 m; 19 Feb 2009; B. L. Fisher et al. leg.; rainforest, ex rotten log; BLF22093 (CASC).

##### Diagnosis.

Minute species. ***Major workers.***HL < 1.0 mm and WL < 0.8 mm; head in full-face view sub-oval, widening slightly posteriorly, with anterior and posterior sides convex, in lateral view sub-oval; ventral and dorsal faces convex; body yellowish orange; sides of head with moderately dense, moderately long, suberect to erect pilosity; entire head distinctly sculptured, medial part of frons with thick, dense, longitudinal, and interrupted rugae and smooth to indistinctly rugulate interspaces; scape, when laid back, slightly exceeding the midlength of head; mesosoma with fine and sparse rugofoveolae, pronotum with rugofoveolae reduced and smooth notches on medial parts of dorsum and lateral sides; katepisternum with smooth notch; inner hypostomal teeth distinct, large, closely spaced, triangular, with rounded apex directed upward; outer hypostomal teeth lobe-like, lower than inner teeth and approximately the same weight; inner and outer hypostomal teeth moderately closely spaced and not connected by concavity; base of first gastral tergite smooth. ***Minor workers.***HL < 0.5 mm and WL < 0.6 mm, scape, when laid back, surpassing the posterior head margin by one-fifth of its length; propodeal spines small and triangular; head relatively rectangular; body dark yellow; head foveolate; medial side of frons with sparse, fine, and interrupted rugulae; area posterolateral from eyes smooth; mesosoma smooth.

##### Description.

**Major workers.** Measurements (*N* = 10): HL: 0.9–0.97 (0.93); HW: 0.87–0.95 (0.91); SL: 0.48–0.53 (0.5); EL: 0.1–0.13 (0.11); WL: 0.77–0.84 (0.8); PSL: 0.12–0.15 (0.14); MTL: 0.44–0.5 (0.47); PNW: 0.39–0.46 (0.43); PTW: 0.1–0.14 (0.12); PPW: 0.25–0.31 (0.28); CI: 100.2–104.6 (102.4); SI: 53.1–58.3 (55.0); PSLI: 13.2–15.7 (14.5); PPI: 38.7–48.6 (41.8); PNI: 43.0–49.2 (47.3); MTI: 49.3–54.1 (51.5).

***Head.*** In full-face view sub-oval, widening slightly posteriorly, with anterior and posterior sides convex (Fig. 36B). In lateral view sub-oval; ventral and dorsal faces convex; inner hypostomal teeth visible. Sides of the head with moderately dense, moderately long, suberect to erect pilosity; whole head with dense, long, decumbent to erect pilosity. Medial part of frons with thick, dense, longitudinal, and interrupted rugae and smooth to indistinctly rugulate interspaces; lateral sides with thick, dense, and irregular rugae, interspaces shiny and distinctly rugofoveolate; rugae more longitudinal on anterolateral sides. Occipital lobes with thinner, irregular rugae and smooth to indistinctly rugofoveolate interspaces. Area posterolateral from eyes with sculpture weaker and rugoreticulate; sculpture weakening posteriorly. Gena with relatively sparse, thick, longitudinal rugae and smooth to indistinctly rugoreticulate interspaces. Centre of clypeus smooth and shiny, lateral sides with indistinct rugulae; median notch present, moderately wide, and shallow; median longitudinal carina present; lateral longitudinal carinae absent. Scape, when laid back, slightly exceeding the midlength of head; pilosity subdecumbent to erect (Fig. 36B, D). Inner hypostomal teeth distinct, large, closely spaced, triangular, with rounded apex directed upward; outer hypostomal teeth lobe-like, lower than inner teeth and approximately the same weight; inner and outer hypostomal teeth moderately closely spaced and not connected by concavity (Fig. 63R). ***Mesosoma.*** In lateral view, promesonotum short, angular, and moderately low, posterior mesonotum moderately steep, mesonotal process very indistinct, tubercle-like or absent; promesonotal groove absent; metanotal groove absent; propodeal spines moderately long, with moderately wide base and acute apex; humeral area laterally weakly produced (Fig. 36D). Surface shiny with fine and sparse rugofoveolae; pronotum with rugofoveolae reduced and smooth notches on medial parts of dorsum and lateral sides; katepisternum with smooth notch. Pilosity moderately sparse, long, and erect (Fig. 36D, F). ***Petiole.*** Shiny with fine foveolae; node smooth to finely foveolate, triangular, with rounded and thick apex, in rear view node dorsoventrally straight to slightly convex; pilosity moderately sparse and erect (Fig. 36D, F). ***Postpetiole.*** Shiny and foveolate; dorsum with reduced sculpture and smooth notch; in dorsal view oval, lateral margins medially with two dentate projections; pilosity long, moderately sparse, and erect (Fig. 36D, F). ***Gaster.*** Shiny and smooth; pilosity dense, moderately long, and erect (Fig. 36D, F). ***Colour.*** Yellowish orange; mandibles and gaster brown (Fig. 36D, F).

**Minor workers.** Measurements (*N* = 10): HL: 0.47–0.52 (0.49); HW: 0.4–0.45 (0.42); SL: 0.43–0.48 (0.45); EL: 0.09–0.11 (0.1); WL: 0.53–0.61 (0.57); PSL: 0.04–0.06 (0.05); MTL: 0.33–0.41 (0.37); PNW: 0.27–0.3 (0.28); PTW: 0.05–0.08 (0.06); PPW: 0.1–0.14 (0.12); CI: 111.9–121.1 (117.0); SI: 103.9–108.0 (106.1); PSLI: 7.9–11.7 (10.3); PPI: 48.3–64.2 (55.5); PNI: 64.9–69.7 (67.0); MTI: 80.2–98.8 (85.4).

***Head.*** Cephalic margin indistinctly concave to straight (Fig. 36A). Pilosity relatively dense, moderately long, decumbent to suberect. Sculpture shiny and foveolate; medial side of frons with sparse, fine, and interrupted rugulae; area posterolateral from eyes smooth; antennal sockets with few thick, curved outward rugae and indistinctly foveolate interspaces. Clypeus with median longitudinal carina absent; two lateral longitudinal carinae absent. Scape, when laid back, surpassing the posterior head margin by one-fifth of its length; pilosity dense, subdecumbent to erect (Fig. 36A, C). ***Mesosoma.*** In lateral view, promesonotum moderately high and short, arched; promesonotal groove absent; metanotal groove distinct; propodeal spines small and triangular (Fig. 36C). Sculpture shiny and smooth. Pilosity moderately sparse, moderately long, and erect (Fig. 36C, E). ***Gaster.*** With sparse, erect pilosity (Fig. 36C, E). ***Colour.*** Dark yellow, gaster and vertex brown (Fig. 36C, E).

##### Etymology.

Malagasy for small in reference to body size.

##### Biology.

The species was collected between 325–990 m in elevation, in rainforest and Uapaca woodland. Nests were located in rotten logs, under rootmats, and under stones.

##### Comments.

*Pheidole kely* sp. nov. belongs to the group of species characterised by small body size (major workers: HL < 1.05 mm, WL < 0.9 mm and minor workers HL < 0.5 mm, WL < 0.6 mm), sub-oval, slightly widening posteriorly head with anterior and posterior sides convex in major workers, and minor workers with yellow to brown body colouration and foveolate head or head predominantly smooth and relatively oval. The group includes six species: *P. havoana* sp. nov., *P. kely* sp. nov., *P. parvula* sp. nov., *P. parvulogibba* sp. nov., *P. volontany* sp. nov., and *P. midongy* sp. nov. *Pheidole kely* sp. nov. is distributed from Toamasina north to Sambava and has a single record from Parc National d’Isalo in Fianarantsoa. Morphologically it is most similar to *P. havoana* sp. nov. and *P. parvulogibba* sp. nov., described from the Anosyenne Mts. in Toliara, and parapatric *P. parvula* sp. nov., known from two localities in the vicinity of Antananarivo. Minor workers of *P. kely* sp. nov. distinctly differ from those of *P. parvula* sp. nov. and *P. parvulogibba* sp. nov. in having a predominantly foveolate head. Minor workers of *P. havoana* sp. nov. and *P. kely* sp. nov. are indistinguishable. Major workers of *P. kely* sp. nov. differ from *P. havoana* sp. nov. and *P. parvula* sp. nov. in medial part of frons with thick, dense, longitudinal, and interrupted rugae and smooth to indistinctly rugulate interspaces; from *P. parvulogibba* sp. nov. in mesosoma with fine and sparse rugofoveolae, pronotum with rugofoveolae reduced, and smooth notches on medial parts of dorsum and its lateral sides.

#### Pheidole lavasoa sp. nov.

Taxon classificationAnimalia

E64A04E3-6A99-5430-AE3B-E8BEA130A88A

http://zoobank.org/603A3805-0A35-4BAA-9AAA-8BB0882809D3

**Figure 37.**
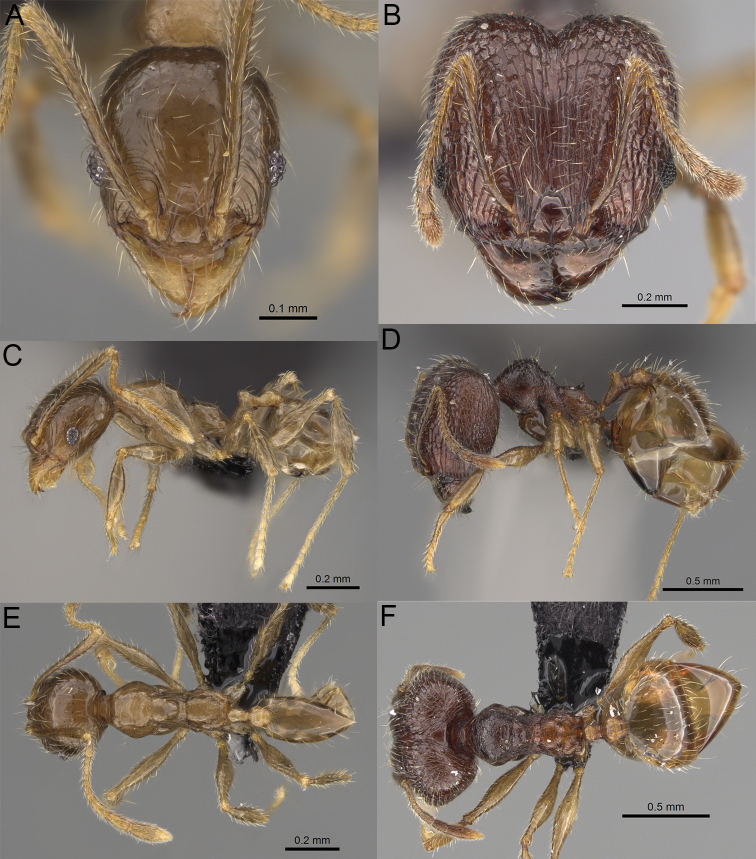
*Pheidole lavasoa* sp. nov., full-face view (**A**), profile (**C**), and dorsal view (**E**) of paratype minor worker (CASENT0923282) and full-face view (**B**), profile (**D**), and dorsal view (**F**) of holotype major worker (CASENT0122898).

Figs 37A–F , 63S , 65S

##### Type material.

***Holotype.*** Madagascar. •1 major worker; Toliara; Grand Lavasoa, 25.9km W Tolagnaro; -25.08767, 46.749; alt. 450 m; 30 Nov 2006; B. L. Fisher et al. leg.; rainforest, ex rotten log; BLF15392; CASENT0122898 (CASC). ***Paratype.*** •1w.; same data as for holotype, CASENT0923282 (CASC).

##### Diagnosis.

Minute species. ***Major workers.***HL < 0.95 mm and WL < 0.8 mm; head in full-face view sub-oval; body dark brown; sides of head with very dense, short, suberect pilosity; entire head distinctly sculptured; scape, when laid back, exceeding the midlength of head by two-fifths of its length; inner hypostomal teeth distinct, large, with rounded apex; outer hypostomal teeth dentate with apex directed indistinctly outward; inner and outer hypostomal teeth not closely spaced and not connected by concavity. ***Minor workers.***HL < 0.5 mm and WL < 0.6 mm, scape, when laid back, surpassing the posterior head margin by two-fifths of its length, propodeal spines reduced to small lobes, head elongate, and oval and body yellowish brown; head sculpture shiny and predominantly smooth, frons with sparse, short, and longitudinal rugulae; mesosoma shiny and smooth.

##### Description.

**Major workers.** Measurements (*N* = 1): HL: 0.94; HW: 0.98; SL: 0.66; EL: 0.12; WL: 0.83; PSL: 0.13; MTL: 0.57; PNW: 0.36; PTW: 0.13; PPW: 0.28; CI: 95.6; SI: 67.0; PSLI: 13.9; PPI: 44.0; PNI: 36.3; MTI: 57.9.

***Head.*** In full-face view sub-oval, slightly widening posteriorly, with anterior and posterior sides convex (Fig. 37B). In lateral view sub-oval; ventral and dorsal faces convex; inner hypostomal teeth visible. Sides of the head with very dense, short, suberect to erect pilosity; whole head with dense, long, decumbent to erect pilosity. Medial part of frons with thick, dense, longitudinal and interrupted rugae and smooth interspaces, posteromedial frons with rugae more irregular and directed slightly outward; lateral sides with thick, dense, and irregular rugae, interspaces shiny and smooth or indistinctly rugulate. Occipital lobes with thick, irregular rugae and smooth to rugoreticulate interspaces. Area posterolateral from eyes rugoreticulate to rugofoveolate with additional longitudinal thick rugae. Gena with relatively dense, thick, and longitudinal rugae and smooth to indistinctly rugoreticulate interspaces. Centre of clypeus smooth and shiny, lateral sides with indistinct rugulae; median notch present, moderately wide, and shallow; median longitudinal carina present; lateral longitudinal carinae absent. Scape, when laid back, exceeding the midlength of head by three-fifths of its length; pilosity subdecumbent to erect (Fig. 37B, D). Inner hypostomal teeth distinct, large, closely spaced, triangular, with rounded apex directed upward; outer hypostomal teeth dentate and lower than inner teeth with sharp apex directed slightly outward; inner and outer hypostomal teeth not closely spaced and not connected by concavity (Fig. 63S). ***Mesosoma.*** In lateral view, promesonotum short, angular, and moderately low, posterior mesonotum moderately steep, mesonotal process very indistinct, tubercle-like; promesonotal groove absent; metanotal groove absent; propodeal spines moderately short, with moderately wide base and acute apex; humeral area laterally weakly produced (Fig. 37D). Surface shiny with fine rugofoveolae; medial parts of lateral sides of pronotum with reduced sculpture. Pilosity moderately dense, long, and erect (Fig. 37D, F). ***Petiole.*** Shiny with fine foveolae; node smooth to finely foveolate, triangular, with rounded and thick apex, in rear view node dorsoventrally straight to slightly convex; pilosity moderately sparse and erect (Fig. 37D, F). ***Postpetiole.*** Shiny and foveolate; dorsum with reduced sculpture and smooth notch; in dorsal view oval, lateral margins medially with two dentate projections; pilosity long, moderately sparse, and erect (Fig. 37D, F). ***Gaster.*** Shiny and smooth; pilosity dense, long, and erect (Fig. 37D, F). ***Colour.*** Dark brown; legs, gaster and antennae yellowish brown (Fig. 37D, F).

**Minor workers.** Measurements (*N* = 1): HL: 0.48; HW: 0.42; SL: 0.54; EL: 0.1; WL: 0.58; PSL: 0.05; MTL: 0.4; PNW: 0.27; PTW: 0.07; PPW: 0.11; CI: 116.4; SI: 130.8; PSLI: 9.7; PPI: 65.5; PNI: 65.1; MTI: 95.4.

***Head.*** Cephalic margin indistinctly convex (Fig. 37A). Pilosity relatively dense, long, decumbent to suberect. Sculpture shiny and smooth; frons with sparse, short, and longitudinal rugulae; antennal sockets with few thick, curved outward rugae and smooth interspaces. Clypeus with median longitudinal carina absent; two lateral longitudinal carinae absent. Scape, when laid back, surpassing the posterior head margin by two-fifths of its length; pilosity dense, subdecumbent to erect (Fig. 37A, C). ***Mesosoma.*** In lateral view, promesonotum moderately high and short, arched; promesonotal groove absent; metanotal groove distinct; propodeal spines reduced to small lobes (Fig. 37C). Sculpture shiny and smooth. Pilosity moderately sparse, long, and erect (Fig. 37C, E). ***Gaster.*** With sparse, erect pilosity (Fig. 37C, E). ***Colour.*** Yellowish brown (Fig. 37C, E).

##### Etymology.

From the type locality.

##### Biology.

The species was collected at 450 m in elevation, in rainforest. Nest was located in a rotten log.

##### Comments.

*Pheidole lavasoa* sp. nov., described from Grand Lavasoa, Toliara, resembles the parapatric species *P. andohahela* sp. nov. known to date from Col de Tanatana in Parc National Andohahela, Toliara. Major workers of both taxa are very similar and can be separated based on slight difference in setosity of lateral sides of head (which is denser and shorter in *Pheidole lavasoa* sp. nov.) and shape of hypostomal teeth (*P. lavasoa* sp. nov. has inner hypostomal teeth with rounded apex and outer hypostomal teeth are dentate and indistinctly directed outward). A better resource for species separation are minor workers. Minor workers of *Pheidole lavasoa* sp. nov. lack transverse rugulae on vertex and frons and their frons have sparse, short, and longitudinal rugulae, mesosoma is entirely smooth, and body is yellowish brown.

#### Pheidole litigiosa

Taxon classificationAnimalia

Forel, 1892 stat. nov.

2A11C461-D95A-541C-A4E1-8A33708A0E86

**Figure 38.**
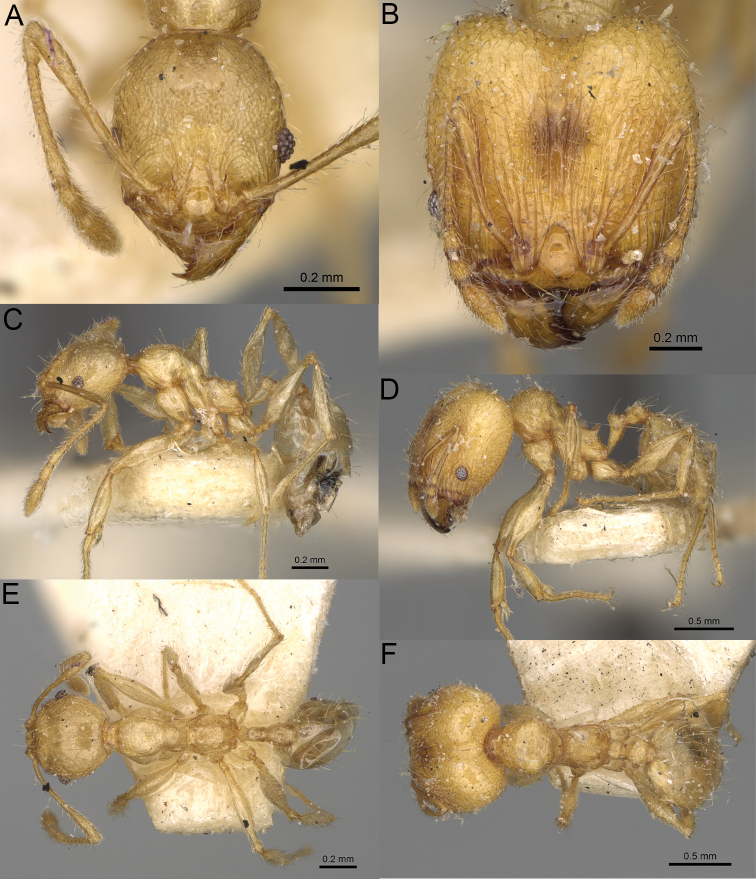
*Pheidole litigiosa* Forel, full-face view (**A**), profile (**C**), and dorsal view (**E**) of minor worker (CASENT0923196) and full-face view (**B**), profile (**D**), and dorsal view (**F**) of major worker (CASENT0923195).

Figs 38A–F , 63T , 65T

##### Type material.

Pheidole sikorae var. litigiosa Forel, 1892: 526 (s.w.q.). Lectotype [designated here]: major worker (top specimen, CASENT0101628): Madagascar, Antananarivo, Forêt d’Andrangoloaka, coll. Sikora (MHNG) [examined]. Paralectotypes: 1 major worker (CASENT0923211) (MHNG) [examined], 1 minor worker (CASENT0923210) (MHNG) [examined], 1 queen (CASENT0101935) (MHNG) [examined], 1 worker (CASENT0101638) (MHNG) [examined].

##### Other material.

Madagascar. –**Fianarantsoa**: • 1w.,1s.; Imerina; Sikora leg.; CASENT0923196, CASENT0923195; ANTC46557 (MHNG).

##### Diagnosis.

Moderately large species. ***Major workers.*** Head in full-face view sub-oval and not widening posteriorly, with anterior and posterior sides slightly convex, in lateral view sub-oval; ventral and dorsal faces convex; sides of the head with moderately dense, short, subdecumbent to suberect pilosity; medial part of frons with thick, longitudinal, interrupted, and moderately dense rugae, interspaces rugulate; occipital lobes smooth and shiny; area posterolateral from eyes smooth and shiny; scape, when laid back, exceeding the midlength of head by one-fifth of its length; inner hypostomal teeth distinct, large, closely spaced, triangular, with rounded apex directed upward; outer hypostomal teeth dentate, wider than and approximately as high as inner teeth; inner and outer hypostomal teeth closely spaced and connected by indistinct concavity; promesonotum predominantly smooth with sparse and moderately thick, irregular to transverse rugae on lateral sides; anepisternum, katepisternum, and propodeum finely rugofoveolate; katepisternum with smooth notch; body yellowish orange. ***Minor workers.*** Head foveolate; vertex, area posterolateral from eyes smooth; scape, when laid back, exceeding the posterior head margin by one-fifth of its length; promesonotum moderately low and short; promesonotal groove present; propodeal spines small, triangular; mesosoma smooth; propodeum and sometimes katepisternum with sparse foveolae; body orange to yellow.

##### Description.

**Major workers.** Measurements (*N* = 3): HL: 1.1–1.17 (1.12); HW: 1.02–1.11 (1.06); SL: 0.59–0.63 (0.61); EL: 0.12–0.13 (0.12); WL: 0.85–1.03 (0.95); PSL: 0.17–0.22 (0.19); MTL: 0.55–0.65 (0.6); PNW: 0.51–0.55 (0.53); PTW: 0.16–0.16 (0.16); PPW: 0.3–0.36 (0.33); CI: 105.0–107.2 (106.2); SI: 56.0–59.5 (57.7); PSLI: 15.7–18.6 (16.8); PPI: 42.9–52.4 (47.7); PNI: 49.0–52.4 (50.6); MTI: 52.6–58.5 (56.2).

***Head.*** In full-face view sub-oval, not widening posteriorly, with anterior and posterior sides slightly convex (Fig. 38B). In lateral view sub-oval; ventral and dorsal faces convex; inner hypostomal teeth visible. Sides of the head with moderately dense, short, subdecumbent to suberect pilosity; whole head with dense, long, decumbent to erect pilosity. Medial part of frons with thick, longitudinal, interrupted, and moderately dense rugae, interspaces rugulate, rugae directed slightly outward on posteromedial part; lateral sides with thick, dense, and longitudinal rugae with distinctly rugulate interspaces, rugae more irregular on the posterolateral parts. Occipital lobes smooth and shiny. Area posterolateral from eyes smooth and shiny. Gena with relatively sparse and thick longitudinal rugae and smooth to indistinctly rugulate interspaces. Centre of clypeus smooth and shiny, lateral sides with indistinct rugulae; median notch present, moderately wide, and shallow; median longitudinal carina absent; lateral longitudinal carinae absent. Scape, when laid back, exceeding the midlength of head by one-fifth of its length; pilosity subdecumbent to erect (Fig. 38B, D). Inner hypostomal teeth distinct, large, closely spaced, triangular, with rounded apex directed upward; outer hypostomal teeth dentate, wider than and approximately as high as inner teeth; inner and outer hypostomal teeth closely spaced and connected by indistinct concavity (Fig. 63T). ***Mesosoma.*** In lateral view, promesonotum short, angular, and moderately low, posterior mesonotum moderately steep, mesonotal process indistinct, tubercle-like; promesonotal groove absent; metanotal groove absent; propodeal spines moderate, with moderately narrow base and acute apex; humeral area weakly produced (Fig. 38D). Surface shiny; promesonotum predominantly smooth with sparse and moderately thick, irregular to transverse rugae on lateral sides; anepisternum, katepisternum, and propodeum finely rugofoveolate; katepisternum with smooth notch. Pilosity moderately dense, long, and erect (Fig. 38D, F). ***Petiole.*** Shiny with sparse foveolae; node finely foveolate, triangular, with rounded and thick apex, in rear view node dorsoventrally slightly concave; pilosity moderately sparse and erect (Fig. 38D, F). ***Postpetiole.*** Shiny and foveolate; dorsum with reduced sculpture and smooth notch; in dorsal view oval, lateral margins medially with two moderately large dentate projections; pilosity long, moderately sparse, and erect (Fig. 38D, F). ***Gaster.*** Shiny and smooth; pilosity moderately dense, moderately long, and erect (Fig. 38D, F). ***Colour.*** Yellowish orange with yellow legs (Fig. 38D, F).

**Minor workers.** Measurements (*N* = 3): HL: 0.54–0.6 (0.57); HW: 0.46–0.52 (0.5); SL: 0.57–0.62 (0.59); EL: 0.09–0.1 (0.09); WL: 0.68–0.8 (0.73); PSL: 0.09–0.11 (0.1); MTL: 0.46–0.56 (0.5); PNW: 0.28–0.33 (0.31); PTW: 0.08–0.11 (0.1); PPW: 0.13–0.17 (0.15); CI: 112.7–118.1 (115.4); SI: 111.3–124.4 (118.3); PSLI: 15.3–18.9 (17.6); PPI: 61.3–69.6 (64.5); PNI: 54.6–68.1 (61.9); MTI: 88.2–107.1 (100.7).

***Head.*** Cephalic margin relatively straight (Fig. 38A). Pilosity relatively sparse, moderately long, decumbent to subdecumbent. Sculpture shiny and foveolate; vertex, and area posterolateral from eyes smooth; antennal sockets with few thick, curved outward rugae and foveolate interspaces. Clypeus with median longitudinal carina absent; two lateral longitudinal carinae absent. Scape, when laid back, exceeding the posterior head margin by one-fifth of its length; pilosity dense, suberect to erect (Fig. 38A, C). ***Mesosoma.*** In lateral view, promesonotum moderately low and short, arched; promesonotal groove present; metanotal groove distinct; propodeal spines small, triangular (Fig. 38C). Sculpture shiny and smooth; propodeum and sometimes katepisternum with sparse foveolae. Pilosity very sparse, moderately long, and erect (Fig. 38C, E). ***Gaster.*** With sparse, erect pilosity (Fig. 38C, E). ***Colour.*** Orange to yellow (Fig. 38C, E).

##### Biology.

The species was collected between 1317–1409 m in elevation. Biology unknown.

##### Comments.

*Pheidole litigiosa* Forel is a member of a group of species characterised by distinctly reduced head sculpture in major workers with occipital lobes entirely or predominantly smooth, area posterolateral from eyes partially or entirely smooth and shiny or with reduced sculpture and smooth notches. The group consists of four species: *P. litigiosa*, *P. masoandro* sp. nov., *P. gracilis* sp. nov., and *P. tampony* sp. nov. *Pheidole litigiosa* is the only member of this group known from the Antananarivo prefecture. Morphologically *P. litigiosa* is most similar to *P. masoandro* sp. nov. known from Anosyenne Mts. in Toliara. Its major workers can be separated based on medial part of frons with thick, longitudinal, interrupted, and moderately dense rugae with rugulate interspaces, moderately dense, short, subdecumbent to suberect pilosity on sides of the head, and having outer hypostomal teeth dentate, wider than and approximately as high as inner teeth; minor workers have indistinct promesonotal groove, propodeal spines small and triangular, and frons lacking additional, short rugae. Despite strong morphological differences in major workers, minor workers of *P. litigiosa* are extremely similar to those of *P. sikorae* and *P. antranohofa* sp. nov. and can be separated based on lack of additional, short rugae.

#### Pheidole mahamavo sp. nov.

Taxon classificationAnimalia

1AC6E74F-3C5B-5D4F-B03E-07493737A0CC

http://zoobank.org/CAA38C9F-94E1-4929-8F02-3C172105D2C6

**Figure 39.**
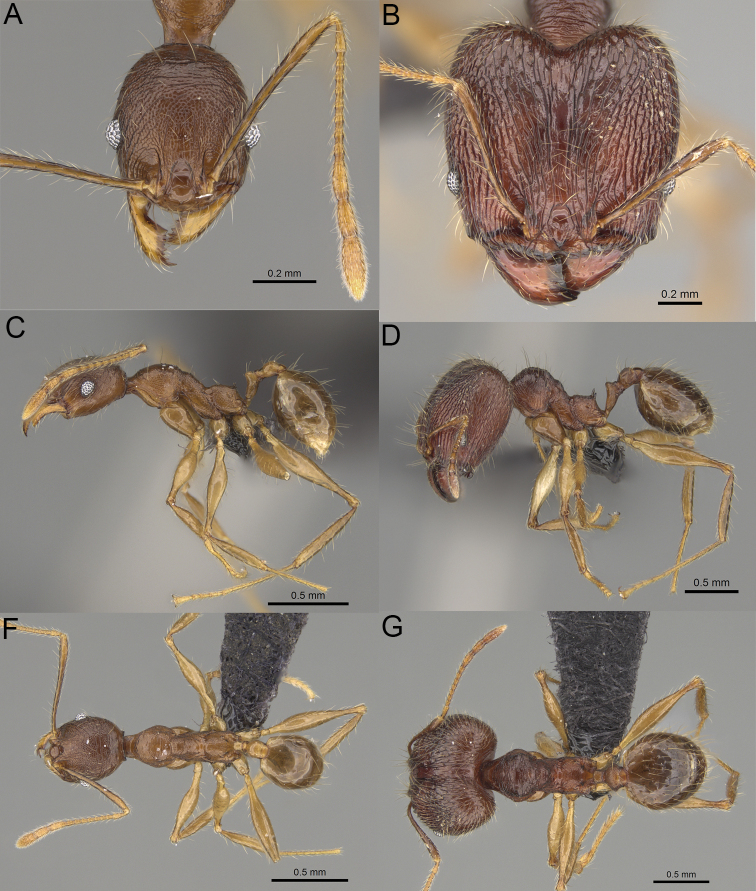
*Pheidole mahamavo* sp. nov., full-face view (**A**), profile (**C**), and dorsal view (**E**) of paratype minor worker (CASENT0455922) and full-face view (**B**), profile (**D**), and dorsal view (**F**) of holotype major worker (CASENT0923292).

Figs 39A–F , 63U , 66A

##### Type material.

***Holotype.*** Madagascar. • 1 major worker; Toliara; Parc National d’Andohahela, Col du Sedro, 3.8 km 113°ESE Mahamavo, 37.6 km 341°NNW Tolagnaro; -24.76389, 46.75167; alt. 900 m; 21 Jan 2002; Fisher et al. leg.; montane rainforest, canopy moss and leaf litter; BLF05135; CASENT0923292, bottom specimen on the pin (CASC). ***Paratypes.*** • 6w., 1s.; same data as for holotype, CASENT0455920, CASENT0455921, CASENT0455922 (CASC, MHNG, PBZT).

##### Diagnosis.

Moderately large species. ***Major workers.*** Head in full-face view sub-oval, not widening posteriorly, with anterior and posterior sides slightly convex, in lateral view sub-oval; ventral and dorsal faces convex; sides of the head with very dense, long, suberect to erect pilosity; medial part of frons with thick, interrupted, dense, and longitudinal rugae with smooth interspaces; lateral sides of frons with thick and denser rugae with smooth to indistinctly rugulate interspaces, anterolateral sides with longitudinal rugae; posterolateral sides with rugae more irregular; occipital lobes and area posterolateral from eyes without smooth notches; scape, when laid back, exceeding the midlength of head by two-fifths of its length; inner hypostomal teeth distinct, large, and wide, closely spaced, triangular with apex directed upward; outer hypostomal teeth lobe-like, narrower, and approximately as high as inner teeth, apex directed upward; inner and outer hypostomal teeth closely spaced and not connected by concavity; mesosoma rugofoveolate; pronotum with reduced rugofoveolae and with additional sparse and thin transverse rugae; katepisternum with smooth notch; gaster smooth; body brown. ***Minor workers.*** Head foveolate; vertex with fading foveolae and additional transverse rugae; frons with distinct and sparse, longitudinal rugae; area posterolateral from eyes smooth; scape, when laid back, exceeding the posterior head margin by one-third of its length; promesonotum low and moderately long; promesonotal groove present; propodeal spines minute, triangular; mesosoma foveolate; promesonotal dorsum and katepisternum with smooth notches; body brown.

##### Description.

**Major workers.** Measurements (*N* = 2): HL: 1.38, 1.36; HW: 1.33, 1.35; SL: 0.91, 0.91; EL: 0.17, 0.19; WL: 1.2, 1.18; PSL: 0.19, 0.2; MTL: 0.9, 0.91; PNW: 0.51, 0.52; PTW: 0.15, 0.17; PPW: 0.33, 0.35; CI: 104.2, 100.4; SI: 68.3, 67.5; PSLI: 13.9, 14.5; PPI: 44.2, 48.3; PNI: 38.6, 38.3; MTI: 67.8, 67.1.

***Head.*** In full-face view sub-oval, not widening posteriorly, with anterior and posterior sides convex (Fig. 39B). In lateral view sub-oval; ventral and dorsal faces convex; inner hypostomal teeth visible. Sides of the head with very dense, long, suberect to erect pilosity; whole head with dense, long, decumbent to erect pilosity. Medial part of frons with thick, interrupted, dense, and longitudinal rugae with smooth interspaces; lateral sides of frons with thick and denser rugae with smooth to indistinctly rugulate interspaces, anterolateral sides with longitudinal rugae; posterolateral sides with more irregular rugae. Occipital lobes with sparser and fading irregular rugae and indistinctly rugulate to smooth interspaces. Area posterolateral from eyes with dense, moderately thick, longitudinal rugae with smooth to indistinctly rugulate interspaces, sculpture slightly fading posteriorly. Gena with relatively sparse, thick, longitudinal rugae and indistinctly rugulate interspaces. Centre of clypeus smooth with few longitudinal rugulae and shiny, lateral sides with indistinct rugulae; median notch present, moderately wide, and shallow; median longitudinal carina present; lateral longitudinal carinae absent. Scape, when laid back, exceeding the midlength of head by two-fifths of its length; pilosity subdecumbent to erect (Fig. 39B, D). Inner hypostomal teeth distinct, large, and wide, closely spaced, triangular with apex directed upward; outer hypostomal teeth lobe-like, narrower, and approximately as high as inner teeth, apex directed upward; inner and outer hypostomal teeth closely spaced and not connected by concavity (Fig. 63U). ***Mesosoma.*** In lateral view, promesonotum short, angular, and moderately low, posterior mesonotum moderately steep, mesonotal process indistinct, tubercle-like; promesonotal groove absent; metanotal groove absent; propodeal spines moderately long, narrow and with acute apex; humeral area weakly produced (Fig. 39D). Surface shiny and rugofoveolate; pronotum with reduced rugofoveolae and with additional sparse and thin transverse rugae; katepisternum with smooth notch. Pilosity moderately dense, long, and erect (Fig. 39D, F). ***Petiole.*** Shiny with foveolate; node finely smooth, triangular, with rounded and thick apex, in rear view node dorsoventrally slightly convex; pilosity moderately sparse and erect (Fig. 39D, F). ***Postpetiole.*** Shiny and foveolate; in dorsal view oval, lateral margins medially with two very small and dentate projections; pilosity long, moderately sparse, and erect (Fig. 39D, F). ***Gaster.*** Shiny and smooth; pilosity moderately dense, long, and erect (Fig. 39D, F). ***Colour.*** Brown, antenna and legs yellowish (Fig. 39D, F).

**Minor workers.** Measurements (*N* = 5): HL: 0.64–0.69 (0.67); HW: 0.51–0.54 (0.53); SL: 0.8–0.87 (0.84); EL: 0.13–0.14 (0.13); WL: 0.89–0.93 (0.91); PSL: 0.09–0.11 (0.1); MTL: 0.64–0.73 (0.7); PNW: 0.34–0.37 (0.36); PTW: 0.09–0.1 (0.09); PPW: 0.15–0.16 (0.15); CI: 123.3–127.3 (124.9); SI: 154.3–161.4 (157.1); PSLI: 13.2–16.7 (14.5); PPI: 57.8–66.9 (61.4); PNI: 64.8–68.6 (66.7); MTI: 126.0–136.5 (130.6).

***Head.*** Cephalic margin slightly convex (Fig. 39A). Pilosity relatively sparse, short, subdecumbent. Sculpture shiny and foveolate; vertex with fading foveolae and additional transverse rugae; frons with distinct and sparse, longitudinal rugae; area posterolateral from eyes smooth; antennal sockets with few indistinct, curved outward rugae and foveolate interspaces. Clypeus with median longitudinal carina absent; two lateral longitudinal carinae absent. Scape, when laid back, exceeding the posterior head margin by one-third of its length; pilosity dense, subdecumbent to erect (Fig. 39A, C). ***Mesosoma.*** In lateral view, promesonotum low and moderately long, arched; promesonotal groove indistinct; metanotal groove distinct; propodeal spines minute and triangular (Fig. 39C). Sculpture shiny and foveolate; promesonotal dorsum and katepisternum with smooth notches. Pilosity sparse, moderately long, and erect (Fig. 39C, E). ***Gaster.*** With sparse, erect pilosity (Fig. 39C, E). ***Colour.*** Brown, legs, gaster and antenna yellowish (Fig. 39C, E).

##### Etymology.

From the type locality.

##### Biology.

The species was collected at 900 m in elevation, in montane rainforest. Nest was located in leaf litter.

##### Comments.

*Pheidole mahamavo* sp. nov. is most similar to sympatric *P. veteratrix*, and to *P. trichotos* sp. nov., and *P. anomala* sp. nov., known from a remote locality in Antsiranana prefecture. Major workers of *P. mahamavo* sp. nov. can be easily separated from those three taxa based on presence of thick, interrupted, dense, and longitudinal rugae with smooth interspaces on medial frons and more longitudinal rugae on lateral sides of frons; minors can be separated from *P. veteratrix* and *P. trichotos* sp. nov. based on presence of distinct and arcing rugae on vertex and sparser sculpture on pronotal dorsum, and from *P. anomala* sp. nov. based on darker brown body colouration, sparser sculpture on propodeum, and presence of distinct and arcing rugae on vertex.

#### Pheidole mainty sp. nov.

Taxon classificationAnimalia

206193C3-EB1D-5F3A-8DA5-19D451D92280

http://zoobank.org/0F8A6886-41A2-4031-A3C8-26E286D37819

**Figure 40.**
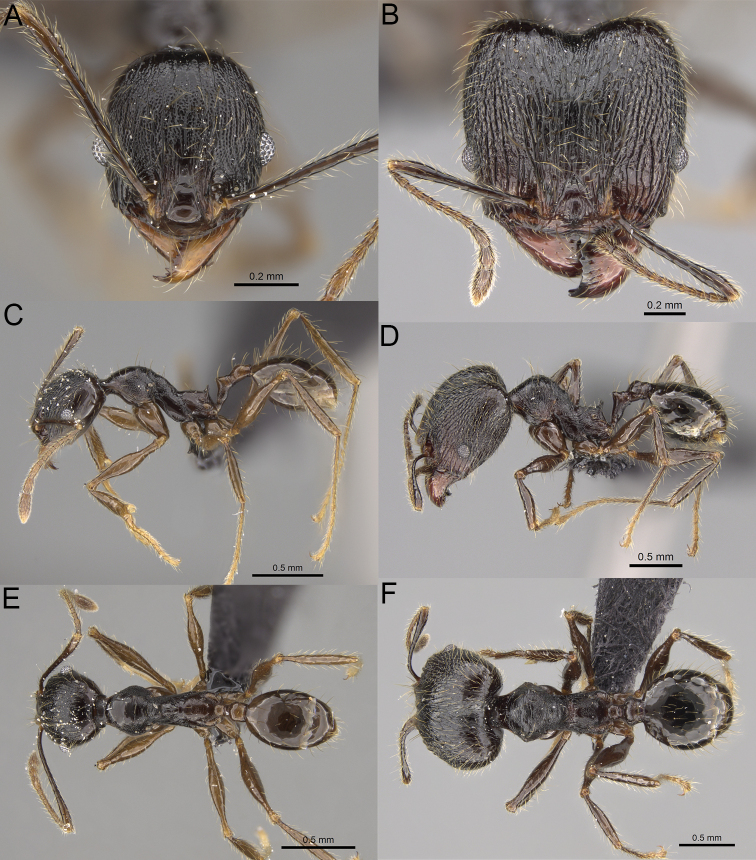
*Pheidole mainty* sp. nov., full-face view (**A**), profile (**C**), and dorsal view (**E**) of paratype minor worker (CASENT0809553) and full-face view (**B**), profile (**D**), and dorsal view (**F**) of holotype major worker (CASENT0923261).

Figs 40A–F , 63V , 66B

##### Type material.

***Holotype.*** Madagascar. • 1 major worker; Antsiranana; Sava Region: Parc National de Marojejy, near summit, 25.4 km 20.1°NE Andapa; -14.44918, 49.73243; alt. 2100 m; 10 Feb 2018; B. L. Fisher et al.; montane shrubland, under rootmat, on stone; BLF40947; CASENT0923261 (CASC). ***Paratype.*** • 1w.; same data as for holotype, CASENT0809553 (CASC).

##### Diagnosis.

Moderately large species. ***Major workers.*** Head in full-face view sub-oval and slightly widening posteriorly, with anterior and posterior sides slightly convex, in lateral view sub-oval; ventral and dorsal faces convex; sides of the head with very dense, moderately long, suberect to erect pilosity; medial part of frons with thick, interrupted, dense, and longitudinal rugae with smooth to indistinctly rugulate interspaces; occipital lobes and area posterolateral from eyes without smooth notches; scape, when laid back, exceeding the midlength of head by one-fifth of its length; inner hypostomal teeth distinct, moderately large, and narrow, closely spaced, triangular with apex directed slightly inward; outer hypostomal teeth lobe-like, wider and higher than inner teeth, apex directed outward; inner and outer hypostomal teeth closely spaced and not connected by concavity; mesosoma rugofoveolate; katepisternum with smooth notch; dorsal side of promesonotum with reduced and sparser sculpture; gaster smooth; body black. ***Minor workers.*** Head foveolate; vertex with fading foveolae; frons with distinct and sparse, longitudinal rugae; area posterolateral from eyes smooth; scape, when laid back, exceeding the posterior head margin by one-third of its length; promesonotum low and moderately long; promesonotal groove present; propodeal spines small, triangular; mesosoma foveolate; promesonotal dorsum, katepisternum, and propodeum predominantly smooth; body black.

##### Description.

**Major workers.** Measurements (*N* = 1): HL: 1.34; HW: 1.33; SL: 0.9; EL: 0.18; WL: 1.25; PSL: 0.22; MTL: 0.94; PNW: 0.53; PTW: 0.16; PPW: 0.36; CI: 100.4; SI: 67.8; PSLI: 16.5; PPI: 46.1; PNI: 39.6; MTI: 70.5.

***Head.*** In full-face view sub-oval, slightly widening posteriorly, with anterior and posterior sides convex (Fig. 40B). In lateral view sub-oval; ventral and dorsal faces convex; inner hypostomal teeth invisible. Sides of the head with very dense, moderately long, suberect to erect pilosity; whole head with dense, long, decumbent to erect pilosity. Medial part of frons with thick, interrupted, dense, and longitudinal rugae with smooth to indistinctly rugulate interspaces; lateral sides of frons with denser, thick, and longitudinal rugae, interspaces with dense, thick, and distinct rugulae. Occipital lobes with sparser and fading rugae and indistinctly rugulate to smooth interspaces. Area posterolateral from eyes with dense, thick, longitudinal rugae with distinctly rugulate interspaces, sculpture slightly fading posteriorly. Gena with relatively sparse, thick, longitudinal rugae and indistinctly rugulate interspaces. Centre of clypeus smooth with few longitudinal rugulae and shiny, lateral sides with indistinct rugulae; median notch present, moderately wide, and shallow; median longitudinal carina present; lateral longitudinal carinae absent. Scape, when laid back, exceeding the midlength of head by one-fifth of its length; pilosity subdecumbent to erect (Fig. 40B, D). Inner hypostomal teeth distinct, moderately large, and narrow, closely spaced, triangular with apex directed slightly inward; outer hypostomal teeth lobe-like, wider and higher than inner teeth, apex directed outward; inner and outer hypostomal teeth closely spaced and not connected by concavity (Fig. 63V). ***Mesosoma.*** In lateral view, promesonotum short, angular, and moderately low, posterior mesonotum moderately steep, mesonotal process indistinct, tubercle-like; promesonotal groove absent; metanotal groove absent; propodeal spines moderately long, relatively wide, and with acute apex; humeral area weakly produced (Fig. 40D). Surface shiny and rugofoveolate; katepisternum with smooth notch; dorsal side of promesonotum with reduced and sparser sculpture. Pilosity moderately dense, long, and erect (Fig. 40D, F). ***Petiole.*** Shiny with sparse foveolae; node finely smooth, triangular, with rounded and thick apex, in rear view node dorsoventrally slightly convex; pilosity moderately sparse and erect (Fig. 40D, F). ***Postpetiole.*** Shiny and foveolate; dorsum smooth; in dorsal view oval, lateral margins medially with two very small and dentate projections; pilosity long, moderately sparse, and erect (Fig. 40D, F). ***Gaster.*** Shiny and smooth; pilosity moderately dense, long, and erect (Fig. 40D, F). ***Colour.*** Black, antenna and legs brownish (Fig. 40D, F).

**Minor workers.** Measurements (*N* = 1): HL: 0.74; HW: 0.64; SL: 0.93; EL: 0.14; WL: 1.01; PSL: 0.12; MTL: 0.78; PNW: 0.45; PTW: 0.1; PPW: 0.19; CI: 114.3; SI: 144.6; PSLI: 15.9; PPI: 52.1; PNI: 69.8; MTI: 121.6.

***Head.*** Cephalic margin slightly convex (Fig. 40A). Pilosity relatively sparse, short, subdecumbent. Sculpture shiny and foveolate; vertex with fading foveolae; frons with distinct, sparse, and longitudinal rugae; area posterolateral from eyes smooth; antennal sockets with few indistinct, curved outward rugae and foveolate interspaces. Clypeus with median longitudinal carina absent; two lateral longitudinal carinae absent. Scape, when laid back, exceeding the posterior head margin by one-third of its length; pilosity dense, subdecumbent to erect (Fig. 40A, C). ***Mesosoma.*** In lateral view, promesonotum low and moderately long, arched; promesonotal groove indistinct; metanotal groove distinct; propodeal spines small and triangular (Fig. 40C). Sculpture shiny and foveolate; promesonotal dorsum, katepisternum and propodeum predominantly smooth. Pilosity sparse, moderately long, and erect (Fig. 40C, E). ***Gaster.*** With sparse, erect pilosity (Fig. 40C, E). ***Colour.*** Black, with legs, gaster, and antenna yellowish brown (Fig. 40C, E).

##### Etymology.

Malagasy for black in reference to black body colouration.

##### Biology.

The species was collected at 2100 m in elevation, in montane shrubland. Nest was located under rootmats on stones.

##### Comments.

*Pheidole mainty* sp. nov. is a member of a group of taxa characterised by dark body colouration that in major workers ranges from brownish black to black and in minor workers ranges from black (head and mesosoma) to dark brown with body predominantly foveolate (only in dark brown specimens). The group consists of three species: *P. alina* sp. nov., *P. trichotos* sp. nov., and *P. mainty* sp. nov. All members of this group are sympatric and their distribution is limited to the northernmost parts of the island, predominantly in Antsiranana prefecture. Major workers of *Pheidole mainty* sp. nov. differ from *P. trichotos* sp. nov. in medial part of frons with thick, interrupted, dense, and longitudinal rugae with smooth to indistinctly rugulate interspaces, base of first gastral tergite smooth and indistinct, bulge-like inner hypostomal teeth; from *P. alina* sp. nov. in sides of the head with very dense, moderately long, suberect to erect pilosity, distinct, triangular inner hypostomal teeth, and wide and moderately long propodeal spines. Minor workers of *P. mainty* sp. nov. differ from *P. trichotos* sp. nov. and *P. alina* sp. nov. in entirely smooth area posterolateral from eyes and predominantly smooth promesonotal dorsum, katepisternum, and propodeum.

#### Pheidole mamiratra sp. nov.

Taxon classificationAnimalia

57ED11DE-9AFA-5B6C-A9AA-6F4F767B3D0C

http://zoobank.org/B9BF8E79-29DA-40CA-9F90-672FD95E5E25

**Figure 41.**
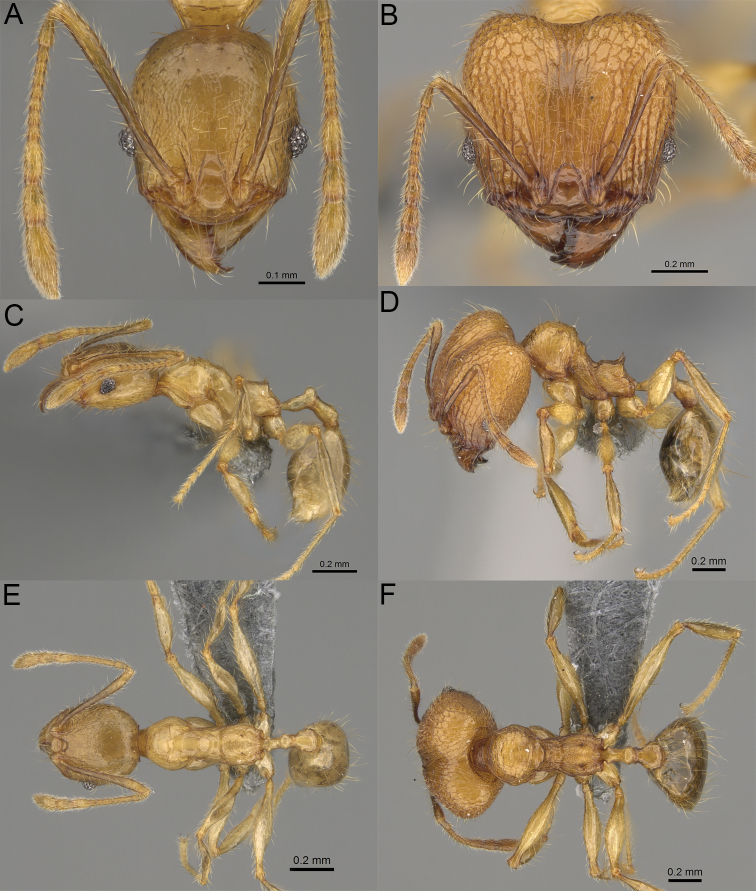
*Pheidole mamiratra* sp. nov., full-face view (**A**), profile (**C**), and dorsal view (**E**) of paratype minor worker (CASENT0923291) and full-face view (**B**), profile (**D**), and dorsal view (**F**) of holotype major worker (CASENT0303725).

Figs 41A–F , 63W , 66C

##### Type material.

***Holotype.*** Madagascar. • 1 major worker; Antananarivo; Station Forestière Angavokely; -18.92207, 47.74157; alt. 1460 m; 9 Mar 2013; B. L. Fisher et al. leg.; rainforest, ex soil; BLF31319; CASENT0303725 (CASC). ***Paratypes.*** • 2w., 1q.; same data as for holotype; CASENT0923291, CASENT0303724 (CASC).

##### Other material.

Madagascar. –**Antananarivo**: • 2w., 1s., 1m.; Station Forestière Angavokely; -18.92207, 47.74157; alt. 1460 m; 9 Mar 2013; B. L. Fisher et al. leg.; rainforest, ground nest; BLF31350 (CASC).

##### Diagnosis.

Moderately large species. ***Major workers.*** Head in full-face view sub-oval, not widening posteriorly, with anterior and posterior sides slightly convex, in lateral view sub-oval, ventral and dorsal faces convex; sides of the head with moderately dense, long, suberect to erect pilosity; medial frons with moderately sparse, thick rugae, anteriorly longitudinal and interrupted, posteromedially rugae irregular, interspaces shiny and predominantly smooth, with sparse and indistinct, irregular rugulae; lateral sides with rugae that are longitudinal anteriorly to irregular posteriorly, thick and relatively sparse rugae with distinctly rugoreticulate interspaces; occipital lobes and area posterolateral from eyes never smooth; scape, when laid back, exceeding the midlength of head by two-fifths of its length; inner hypostomal teeth distinct, small, closely spaced, triangular, with rounded apex directed upward; outer hypostomal teeth lobe-like, wider and higher than inner hypostomal teeth, apex directed outward; inner and outer hypostomal teeth closely spaced, connected by indistinct concavity; pronotum and mesonotum predominantly smooth with indistinct rugofoveolae and transverse rugae; katepisternum, anepisternum, and propodeum with sparse rugofoveolae; body yellow. ***Minor workers.*** Head foveolate; medial part of frons and vertex with strongly reduced sculpture and predominantly smooth; area posterolateral from eyes smooth; scape, when laid back, surpassing the posterior head margin by two-fifths of its length; promesonotum moderately low and short; promesonotal groove absent; propodeal spines very small and triangular; mesosoma smooth; body yellow.

##### Description.

**Major workers.** Measurements (*N* = 2): HL: 0.95, 0.99; HW: 0.97, 1.03; SL: 0.65, 0.65; EL: 0.13, 0.12; WL: 0.94, 0.95; PSL: 0.16, 0.17; MTL: 0.65, 0.61; PNW: 0.41, 0.44; PTW: 0.13, 0.14; PPW: 0.25, 0.26; CI: 97.8, 96.3; SI: 67.5, 62.6; PSLI: 17.1, 17.2; PPI: 52.6, 57.0; PNI: 41.8, 42.7; MTI: 67.6, 59.1.

***Head.*** In full-face view sub-oval, not widening posteriorly, with anterior and posterior sides slightly convex (Fig. 41B). In lateral view sub-oval; ventral and dorsal faces convex; inner hypostomal teeth visible. Sides of the head with moderately dense, long, suberect to erect pilosity; whole head with dense, long, decumbent to erect pilosity. Medial part of frons with moderately sparse, thick rugae, anteriorly longitudinal and interrupted, posteromedially rugae irregular, interspaces shiny and predominantly smooth, with sparse and indistinct irregular rugulae; lateral sides with rugae longitudinal anteriorly to irregular posteriorly, thick and relatively sparse rugae with distinctly rugoreticulate interspaces. Occipital lobes with sparse, thick, and irregular rugae; interspaces smooth. Gena with relatively dense, thick, and longitudinal rugae and smooth interspaces. Area posterolateral from eyes predominantly smooth, with sparse and indistinct rugoreticulae. Centre of clypeus smooth and shiny, lateral sides with indistinct rugulae; median notch present, moderately wide, and shallow; median longitudinal carina present; lateral longitudinal carinae absent. Scape, when laid back, exceeding the midlength of head by two-fifths of its length; pilosity subdecumbent to erect (Fig. 41B, D). Inner hypostomal teeth distinct, small, closely spaced, triangular, with rounded apex directed upward; outer hypostomal teeth lobe-like, wider and higher than inner hypostomal teeth, apex directed outward; inner and outer hypostomal teeth closely spaced, connected by indistinct concavity (Fig. 63W). ***Mesosoma.*** In lateral view, promesonotum short, angular, and moderately high, posterior mesonotum moderately steep, mesonotal process indistinct, tubercle-like; promesonotal groove absent; metanotal groove indistinct; propodeal spines moderately long, moderately wide, with acute apex; humeral area laterally weakly produced (Fig. 41D). Surface shiny; pronotum and mesonotum predominantly smooth with indistinct rugofoveolae and transverse rugae; katepisternum, anepisternum, and propodeum with sparse rugofoveolae. Pilosity relatively dense, long, and erect (Fig. 41D, F). ***Petiole.*** Shiny with fine and dense rugofoveolae; peduncle; node partially smooth, low, triangular, with rounded and thin apex, in rear view node dorsoventrally slightly convex; pilosity moderately sparse and erect (Fig. 41D, F). ***Postpetiole.*** Shiny and smooth; in dorsal view oval, lateral margins medially with two dentate projections; pilosity long, moderately sparse, and erect (Fig. 41D, F). ***Gaster.*** Shiny and smooth; pilosity moderately dense, long, and erect (Fig. 41D, F). ***Colour.*** Yellow; mandibles and gaster slightly darker (Fig. 41D, F).

**Minor workers.** Measurements (*N* = 4): HL: 0.56–0.59 (0.58); HW: 0.47–0.51 (0.49); SL: 0.59–0.61 (0.6); EL: 0.1–0.11 (0.11); WL: 0.69–0.74 (0.71); PSL: 0.08–0.09 (0.08); MTL: 0.48–0.51 (0.5); PNW: 0.31–0.35 (0.33); PTW: 0.08–0.1 (0.09); PPW: 0.14–0.16 (0.15); CI: 115.3–121.7 (117.8); SI: 116.1–127.3 (122.3); PSLI: 13.9–15.6 (14.7); PPI: 55.6–70.6 (60.6); PNI: 64.7–68.8 (66.4); MTI: 99.6–102.7 (101.2).

***Head.*** Cephalic margin indistinctly concave or straight (Fig. 41A). Pilosity relatively sparse, long, decumbent to suberect. Sculpture foveolate; medial part of frons and vertex with strongly reduced sculpture and predominantly smooth; area posterolateral from eyes smooth. Clypeus with median longitudinal carina absent; two lateral longitudinal carinae absent. Scape, when laid back, surpassing the posterior head margin by two-fifths of its length; pilosity dense, subdecumbent to erect (Fig. 41A, C). ***Mesosoma.*** In lateral view, promesonotum moderately low and short, arched; promesonotal groove absent; metanotal groove present and distinct; propodeal spines very small and triangular (Fig. 41C). Sculpture smooth. Pilosity very sparse, moderately long, and erect (Fig. 41C, E). ***Postpetiole.*** Short, low, and relatively flat; with few short, erect setae (Fig. 41C, E). ***Gaster.*** With sparse, erect pilosity (Fig. 41C, E). ***Colour.*** Yellow, vertex slightly darker (Fig. 41C, E).

##### Etymology.

Malagasy for “to shine” or “to give light” in reference to bright body colouration.

##### Biology.

The species was collected at 1460 m in elevation, in rainforest. Nests were in soil.

##### Comments.

*Pheidole mamiratra* sp. nov. is a member of a group of species characterised by body colouration that is bright and yellow to orange in majors and yellow in minors, sub-oval, not widening posteriorly head with sides not convex or convex indistinctly, and medial part of frons with longitudinal and rugae interrupted anteriorly and distinctly irregular posteriorly. The group includes three taxa: *P. vony* sp. nov., *P. befotaka* sp. nov., and *P. mamiratra* sp. nov. *Pheidole mamiratra* sp. nov. is known from Station Forestière Angavokely in Antananarivo and its distribution does not overlap with two remaining members of the group. Morphologically *P. mamiratra* sp. nov. is most similar to *P. vony* sp. nov., known only from two localities in Toamasina: Montagne d’Anjanaharibe and Montagne d’Akirindro. Major workers of *P. mamiratra* sp. nov. differ from *P. vony* sp. nov. in medial part of frons with moderately sparse and thick rugae and inner hypostomal teeth small and triangular with rounded apex directed upward and outer hypostomal teeth lobe-like, wider and higher than inner hypostomal teeth; minors differ in less distinctly foveolate head sculpture with predominantly smooth frons and entirely smooth area posterolateral from eyes. Additionally, minor and major workers of *P. mamiratra* sp. nov. can be also confused with *P. sparsa* sp. nov., known from Bemanevika in Mahajanga, and sympatric *P. hazo* sp. nov., described from the vicinity of Antananarivo. Majors of *P. mamiratra* sp. nov. differ from *P. sparsa* sp. nov. and *P. hazo* sp. nov. in median frons with distinctly irregular rugae and more irregular sculpture on later sides of frons and outer hypostomal teeth wider and higher than inner hypostomal teeth; additionally, majors of sympatric *P. hazo* sp. nov. differ in lack of smooth notches on katepisternum. Minor workers of *P. mamiratra* sp. nov. differ from *P. sparsa* sp. nov. and *P. hazo* sp. nov. in medial part of frons and vertex with strongly reduced sculpture, predominantly smooth vertex, and lack of additional, indistinct rugulae on head.

#### Pheidole manantenina sp. nov.

Taxon classificationAnimalia

0C422DCB-0CDF-53F4-8930-025E13E278F4

http://zoobank.org/AE2C43FF-B209-45A6-8941-EB9F7679F4A8

**Figure 42.**
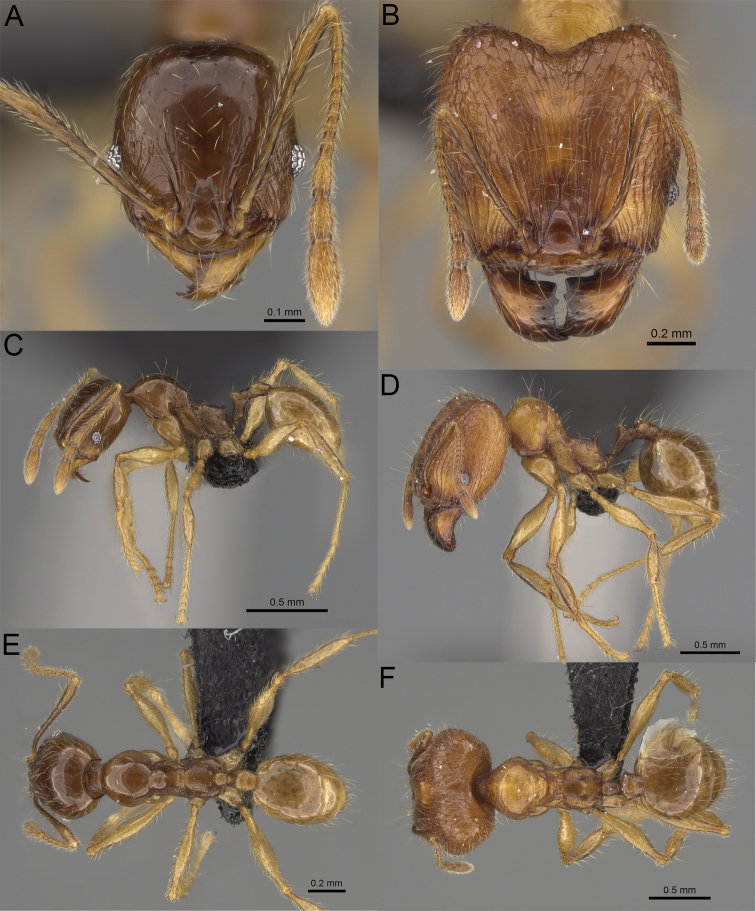
*Pheidole manantenina* sp. nov., full-face view (**A**), profile (**C**), and dorsal view (**E**) of paratype minor worker (CASENT0487100) and full-face view (**B**), profile (**D**), and dorsal view (**F**) of holotype major worker (CASENT0923299).

Figs 42A–F , 63X , 66D

##### Type material.

***Holotype.*** Madagascar. • 1 major worker; Antsiranana; Parc National de Marojejy, 25.4 km 30°NNE Andapa, 10.9 km 311°NW Manantenina; -14.445, 49.735; alt. 2000 m; 23 Nov 2003; B. L. Fisher et al. leg.; montane shrubland, under moss, on ground; BLF09343; CASENT0923299, top specimen on the pin (CASC). ***Paratypes.*** • 6w., 2s.; same data as for holotype; CASENT0487098, CASENT0487099, CASENT0487100 (CASC, MHNG, PBZT).

##### Other material.

Madagascar. –**Antsiranana**: • 3w.; Parc National de Marojejy, 25.7 km 32°NNE Andapa, 10.3 km 314°NW Manantenina; -14.445, 49.74167; alt. 1575 m; 22 Nov 2003; B. L. Fisher et al. leg.; montane rainforest, ex rotten log; BLF09267 (CASC). • 6w., 3s., 1m., 1q.; Parc National de Marojejy, 25.4 km 30°NNE Andapa, 10.9 km 311°NW Manantenina; -14.445, 49.735; alt. 2000 m; 23 Nov 2003; B. L. Fisher et al. leg.; montane shrubland, under moss, on ground; BLF09334 (CASC). • 3w., 3s., 1q.; ibid.; montane shrubland, ex root mat, ground layer; BLF09340 (CASC). • 5w., 1s.; ibid.; BLF09345 (CASC). • 3s.; ibid.; 24 Nov 2003; montane shrubland, ex rotten log; BLF09360 (CASC). 3w., 3s.; ibid.; montane shrubland, under moss, on ground; BLF09388 (CASC). • 3w.; ibid.; montane shrubland, under moss, on ground; BLF09403 (CASC). • 3w., 3s.; ibid.; montane shrubland, under moss, on ground; BLF09415 (CASC).

##### Diagnosis.

Moderately large species. ***Major workers.*** Head in full-face view sub-oval, not widening posteriorly, with anterior and posterior sides convex, slightly narrowing posteriorly, in lateral view sub-oval; ventral and dorsal faces convex; sides of the head with dense, long, erect pilosity; medial part of frons with moderately sparse, thick, interrupted, and longitudinal rugae, on posteromedial part rugae directed outward, interspaces with sparse and indistinct rugofoveolae or smooth; lateral sides with irregular to longitudinally irregular and thick rugae with distinctly rugofoveolate interspaces; occipital lobes and area posterolateral from eyes without smooth notches; scape, when laid back, exceeding the midlength of head by two-fifths of its length; inner hypostomal teeth distinct, moderate, closely spaced, triangular, with rounded apex slightly directed inward; outer hypostomal teeth lobe-like, wider than inner hypostomal teeth and approximately the same height, apex directed outward; inner and outer hypostomal teeth closely spaced but not connected; mesosoma with fine rugofoveolae, pronotal dorsum with weaker sculpture and usually its medial part with smooth notch; gaster smooth; body dark orange to brown. ***Minor workers.*** Head smooth with sparse, irregular to longitudinal, thick rugae on anterior frons and gena, interspaces smooth to indistinctly foveolate; scape, when laid back, surpassing the posterior head margin by one-fifth of its length; promesonotum moderately high and short, arched; promesonotal groove present; propodeal spines small and triangular; pronotum and mesonotum smooth and sometimes with indistinct rugofoveolae on lateral sides, anepisternum, katepisternum, and propodeum with indistinct and moderately dense rugofoveolae, katepisternum sometimes with smooth notch; body brown to dark brown.

##### Description.

**Major workers.** Measurements (*N* = 10): HL: 1.23–1.51 (1.34); HW: 1.19–1.5 (1.28); SL: 0.76–0.86 (0.79); EL: 0.11–0.15 (0.13); WL: 1.13–1.3 (1.18); PSL: 0.2–0.23 (0.22); MTL: 0.71–0.91 (0.78); PNW: 0.55–0.67 (0.59); PTW: 0.12–0.19 (0.15); PPW: 0.27–0.4 (0.31); CI: 100.8–107.9 (104.8); SI: 57.1–65.4 (61.5); PSLI: 14.4–17.5 (16.1); PPI: 45.7–50.5 (48.1); PNI: 43.3–48.9 (45.8); MTI: 55.4–64.7 (60.5).

***Head.*** In full-face view sub-oval, not widening posteriorly, with anterior and posterior sides convex, slightly narrowing posteriorly (Fig. 42B). In lateral view sub-oval; ventral and dorsal faces convex; inner hypostomal teeth visible. Sides of the head with dense, long, erect pilosity; whole head with relatively dense, long, decumbent to erect pilosity. Medial part of frons with moderately sparse, thick, interrupted longitudinal rugae, on posteromedial part rugae directed outward, interspaces with sparse and indistinct rugofoveolae or smooth; lateral sides with irregular to longitudinally irregular and thick rugae with distinctly rugofoveolate interspaces. Occipital lobes with thick, sparse, irregular rugae, interspaces smooth or with fine, indistinct rugulae. Gena with relatively dense, thick, and longitudinal rugae and indistinctly rugofoveolate interspaces. Area posterolateral from eyes with sparse and moderately thick rugae with distinctly rugofoveolate interspaces. Centre of clypeus smooth and shiny, lateral sides with indistinct rugulae; median notch present, moderately wide, and shallow; median longitudinal carina absent; lateral longitudinal carinae absent. Scape, when laid back, exceeding the midlength of head by approximately two-fifths of its length; pilosity decumbent to erect (Fig. 42B, D). Inner hypostomal teeth distinct, moderate, closely spaced, triangular, with rounded apex slightly directed inward; outer hypostomal teeth lobe-like, wider than inner hypostomal teeth and approximately the same height, apex directed outward; inner and outer hypostomal teeth closely spaced but not connected (Fig. 63X). ***Mesosoma.*** In lateral view, promesonotum short, angular, and moderately high, posterior mesonotum moderately steep, mesonotal process indistinct, tubercle-like; promesonotal groove absent; metanotal groove absent; propodeal spines moderately long, narrow, with acute apex; humeral area laterally weakly produced (Fig. 42D). Surface shiny with fine rugofoveolae, pronotal dorsum with weaker sculpture and usually its medial part with smooth notch. Pilosity relatively dense, long, and erect (Fig. 42D, F). ***Petiole.*** Shiny with fine and sparse rugofoveolae; peduncle; node smooth or partially rugofoveolate, low, triangular, with rounded and thin apex, in rear view node dorsoventrally convex; pilosity moderately sparse and erect (Fig. 42D, F). ***Postpetiole.*** Shiny, with fine and sparse rugofoveolae laterally, dorsum smooth; in dorsal view postpetiole rectangular, lateral margins medially with two dentate projections; pilosity long, moderately sparse, and erect (Fig. 42D, F). ***Gaster.*** Shiny and smooth; pilosity moderately dense, long, and erect (Fig. 42D, F). ***Colour.*** Brownish to dark orange; legs dark yellow (Fig. 42D, F).

**Minor workers.** Measurements (*N* = 10): HL: 0.66–0.7 (0.68); HW: 0.55–0.6 (0.58); SL: 0.67–0.71 (0.69); EL: 0.11–0.12 (0.11); WL: 0.81–0.91 (0.86); PSL: 0.09–0.11 (0.1); MTL: 0.55–0.59 (0.57); PNW: 0.37–0.4 (0.39); PTW: 0.08–0.1 (0.09); PPW: 0.15–0.17 (0.16); CI: 115.0–123.7 (120.5); SI: 117.2–123.7 (118.8); PSLI: 13.1–16.5 (14.5); PPI: 51.3–65.8 (59.0); PNI: 65.6–70.8 (67.4); MTI: 95.2–104.9 (98.5).

***Head.*** Cephalic margin indistinctly convex or straight (Fig. 42A). Pilosity relatively sparse, long, decumbent to subdecumbent. Head smooth with sparse, irregular to longitudinal, thick rugae on anterior frons and gena, interspaces smooth to indistinctly foveolate; antennal sockets with few thick, curved outward rugae and smooth to rugofoveolae interspaces. Clypeus with median longitudinal carina absent; two lateral longitudinal carinae absent. Scape, when laid back, surpassing the posterior head margin by one-fifth of its length; pilosity dense, subdecumbent to erect (Fig. 42A, C). ***Mesosoma.*** In lateral view, promesonotum moderately high and short, arched; promesonotal groove present but indistinct; metanotal groove present and distinct; propodeal spines very small and triangular (Fig. 42C). Pronotum and mesonotum smooth and sometimes with indistinct rugofoveolae on lateral sides, anepisternum, katepisternum, and propodeum with indistinct and moderately dense rugofoveolae, katepisternum sometimes with smooth notch. Pilosity sparse, moderately long, and erect (Fig. 42C, E). ***Petiole.*** Peduncle short and thin with ventral face relatively straight (Fig. 42C, E). ***Gaster.*** With sparse, erect pilosity (Fig. 42C, E). ***Colour.*** Brown to dark brown, legs always yellow (Fig. 42C, E).

##### Etymology.

From the type locality.

##### Biology.

The species was collected between 1575–2000 m in elevation, in montane shrublands and montane rainforest. Nests were located in rotten logs, under moss, in root mats.

##### Comments.

*Pheidole manantenina* sp. nov. is known from Parc National de Marojejy in Antsiranana and morphologically is most similar to parapatric and widespread *P. sofia* sp. nov. Majors of *P. manantenina* sp. nov. differ from *P. sofia* sp. nov. in medial part of frons with smooth to indistinctly rugofoveolate interspaces, lateral sides of frons with more irregular rugae, body brownish to dark orange, and promesonotum with reduced sculpture; minor workers can be separated based on more reduced sculpture on head, and mesosoma predominantly smooth with indistinct rugofoveolae.

#### Pheidole masoandro sp. nov.

Taxon classificationAnimalia

DC1AC8AD-6399-5591-A428-F5D29F955AF2

http://zoobank.org/9F363E57-AE92-493D-A242-169F9B30CB72

**Figure 43.**
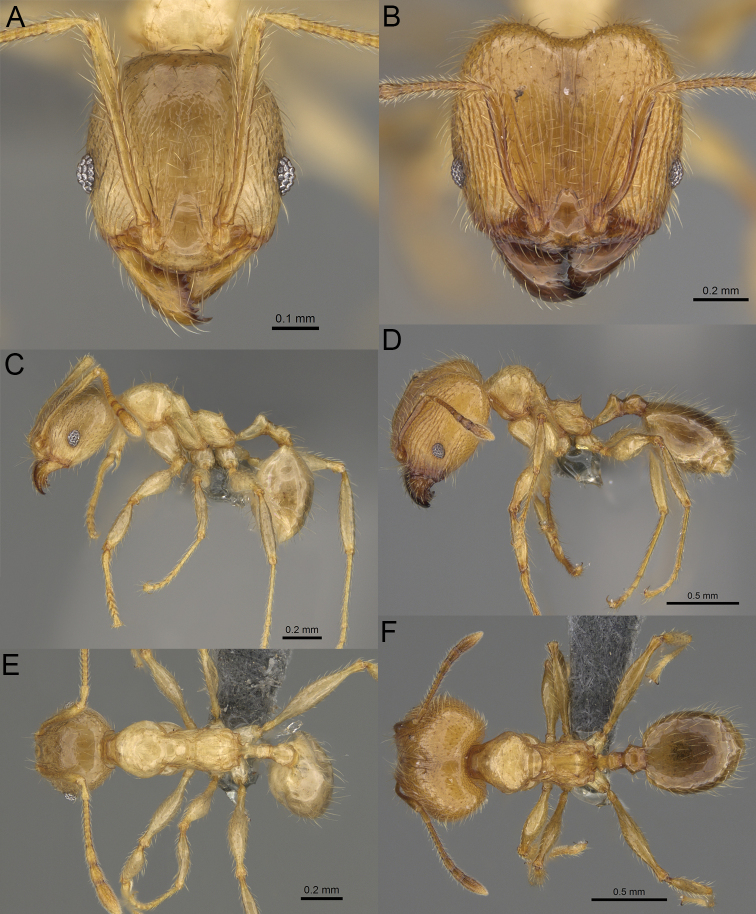
*Pheidole masoandro* sp. nov., full-face view (**A**), profile (**C**), and dorsal view (**E**) of paratype minor worker (CASENT0923293) and full-face view (**B**), profile (**D**), and dorsal view (**F**) of holotype major worker (CASENT0704282).

Figs 43A–F , 64A , 66E

##### Type material.

***Holotype.*** Madagascar. • 1 major worker; Toliara; Anosy Region, Anosyenne Mts, 31.2 km NW Manantenina; -24.13894, 47.06804; alt. 1125 m; 26 Feb 2015; B. L. Fisher et al. leg.; rainforest, ex root mat; BLF36559; CASENT0704282 (CASC). ***Paratype.*** • 1w.; same data as for holotype; CASENT0923293 (CASC).

##### Diagnosis.

Moderately large species. ***Major workers.*** Head in full-face view sub-oval and not widening posteriorly, with anterior and posterior sides slightly convex, in lateral view sub-oval; ventral and dorsal faces convex; sides of the head with very dense, long, erect pilosity; medial part of frons with moderately dense, thick, interrupted longitudinal rugae, interspaces smooth; occipital lobes smooth and shiny; area posterolateral from eyes with reduced sculpture and smooth notch; scape, when laid back, exceeding the midlength of head by approximately two-fifths of its length; inner hypostomal teeth distinct, moderately high, closely spaced, triangular, with rounded apex slightly directed outward; outer hypostomal teeth lobe-like, wider and higher than inner hypostomal teeth, apex directed outward; inner and outer hypostomal teeth closely spaced but not connected; mesosoma predominantly smooth, indistinct, thin, sparse, and irregular rugae occur on lateral sides; body yellow. ***Minor workers.*** Medial part of frons with sparse and fading foveolae and short, longitudinal rugae; lateral sides with thicker and denser foveolae and longitudinal, interrupted, and thick rugae; area posterolateral from eyes smooth; scape, when laid back, surpassing the posterior head margin by one-fifth of its length; promesonotum moderately low and short; promesonotal groove present; propodeal spines minute and triangular; mesosoma smooth with indistinct foveolae on posterior katepisternum; body yellow.

##### Description.

**Major workers.** Measurements (*N* = 1): HL: 1.05; HW: 1.05; SL: 0.59; EL: 0.15; WL: 1.0; PSL: 0.17; MTL: 0.64; PNW: 0.48; PTW: 0.16; PPW: 0.26; CI: 100.1; SI: 56.4; PSLI: 16.2; PPI: 59.8; PNI: 45.5; MTI: 61.0.

***Head.*** In full-face view sub-oval, not widening posteriorly, with anterior and posterior sides convex (Fig. 43B). In lateral view sub-oval; ventral and dorsal faces convex; inner hypostomal teeth invisible. Sides of the head with very dense, long, erect pilosity; whole head with dense, long, decumbent to erect pilosity. Medial part of frons with moderately dense, thick, interrupted longitudinal rugae, interspaces smooth; lateral sides with longitudinal and thick rugae with distinctly rugofoveolate interspaces. Occipital lobes smooth and shiny. Gena with relatively dense, thick, and longitudinal rugae and distinctly rugofoveolate interspaces. Area posterolateral from eyes with reduced sculpture and smooth notch. Centre of clypeus smooth and shiny, lateral sides with indistinct rugulae; median notch present, moderately wide, and shallow; median longitudinal carina absent; lateral longitudinal carinae absent. Scape, when laid back, exceeding the midlength of head by approximately two-fifths of its length; pilosity decumbent to erect (Fig. 43B, D). Inner hypostomal teeth distinct, moderately high, closely spaced, triangular, with rounded apex slightly directed outward; outer hypostomal teeth lobe-like, wider and higher than inner hypostomal teeth, apex directed outward; inner and outer hypostomal teeth closely spaced but not connected (Fig. 64A). ***Mesosoma.*** In lateral view, promesonotum short, angular, and moderately high, posterior mesonotum moderately steep, mesonotal process indistinct, tubercle-like; promesonotal groove absent; metanotal groove indistinct; propodeal spines moderately long, wide, with acute apex; humeral area laterally weakly produced (Fig. 43D). Surface shiny and predominantly smooth, indistinct, thin, sparse, and irregular rugae occur on lateral sides. Pilosity relatively dense, long, and erect (Fig. 43D, F). ***Petiole.*** Shiny with fine and sparse rugofoveolae; peduncle; node smooth, low, triangular, with rounded and thin apex, in rear view node dorsoventrally convex; pilosity moderately sparse and erect (Fig. 43D, F). ***Postpetiole.*** Shiny and smooth; in dorsal view oval, lateral margins medially with two dentate projections; pilosity long, moderately sparse, and erect (Fig. 43D, F). ***Gaster.*** Shiny and smooth; pilosity moderately dense, long, and erect (Fig. 43D, F). ***Colour.*** Yellow; mandibles and gaster slightly darker (Fig. 43D, F).

**Minor workers.** Measurements (*N* = 1): HL: 0.61; HW: 0.56; SL: 0.58; EL: 0.11; WL: 0.77; PSL: 0.09; MTL: 0.5; PNW: 0.35; PTW: 0.09; PPW: 0.16; CI: 109.7; SI: 103.8; PSLI: 14.1; PPI: 58.5; PNI: 62.5; MTI: 90.8.

***Head.*** Cephalic margin indistinctly convex or occipital carina absent (Fig. 43A). Pilosity relatively sparse, long, decumbent to subdecumbent. Medial part of frons with sparse and fading foveolae and short, longitudinal rugae; lateral sides with thicker and denser foveolae and longitudinal, interrupted, and thick rugae; area posterolateral from eyes smooth. Clypeus with median longitudinal carina absent; two lateral longitudinal carinae absent. Scape, when laid back, surpassing the posterior head margin by one-fifth of its length; pilosity dense, subdecumbent to erect (Fig. 43A, C). ***Mesosoma.*** In lateral view, promesonotum moderately high and short, arched; promesonotal groove present and distinct; metanotal groove present and distinct; propodeal spines minute and triangular (Fig. 43C). Sculpture smooth with indistinct foveolae on posterior katepisternum. Pilosity sparse, moderately long, and erect (Fig. 43C, E). ***Petiole.*** Peduncle short and thin with ventral face relatively straight(Fig. 43C, E). ***Postpetiole.*** Short, low, and relatively flat; with few short, erect setae (Fig. 43C, E). ***Gaster.*** With sparse, erect pilosity (Fig. 43C, E). ***Colour.*** Yellow, vertex and gaster slightly darker (Fig. 43C, E).

##### Etymology.

Malagasy for sun, in reference to bright yellow body colouration.

##### Biology.

The species was collected at 1125 m in elevation, in rainforest. Nest was located in root mat.

##### Comments.

*Pheidole masoandro* sp. nov. is a member of a group of species characterised by distinctly reduced head sculpture in major workers with occipital lobes entirely or predominantly smooth, area posterolateral from eyes partially or entirely smooth and shiny or with reduced sculpture and smooth notches. The group consists of four species: *P. litigiosa*, *P. masoandro* sp. nov., *P. gracilis* sp. nov., and *P. tampony* sp. nov. *Pheidole masoandro* sp. nov. is the only member of this group known from the Anosyenne Mts. in Toliara. Morphologically *P. masoandro* sp. nov. is most similar to *P. litigiosa* distributed in the Antananarivo prefecture. Its major workers can be separated based on medial part of frons with moderately dense, thick, interrupted, and longitudinal rugae and smooth interspaces, sides of the head with very dense, long, erect pilosity, and outer hypostomal teeth lobe-like, wider and higher than inner hypostomal teeth with apex directed outward; minor workers have distinct promesonotal groove, their propodeal spines are minute and triangular, and frons have additional short rugae. Despite strong morphological differences in major workers, minor workers of *P. masoandro* sp. nov., *P. sikorae* and *P. antranohofa* sp. nov. are indistinguishable.

#### Pheidole mavohavoana sp. nov.

Taxon classificationAnimalia

C5FECD65-DE92-5FBC-BEFF-9CCE7AAC7522

http://zoobank.org/EEA3C5B6-0DE0-4305-A179-89166B60B3DA

**Figure 44.**
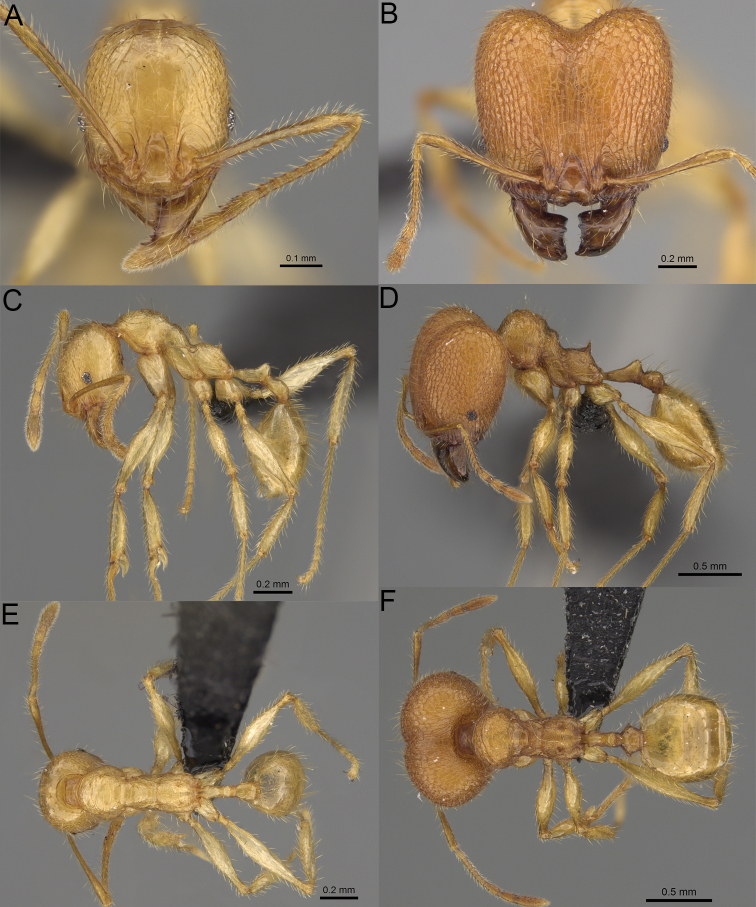
*Pheidole mavohavoana* sp. nov., full-face view (**A**), profile (**C**), and dorsal view (**E**) of paratype minor worker (CASENT0059948) and full-face view (**B**), profile (**D**), and dorsal view (**F**) of holotype major worker (CASENT0923298).

Figs 44A–F , 64B , 66F

##### Type material.

***Holotype.*** Madagascar. • 1 major worker; Fianarantsoa; 2 km W Andrambovato, along river Tatamaly; -21.51167, 47.41; alt. 1075 m; 3 Jun 2005; B. L. Fisher et al. leg.; montane rainforest, ex rotten log; BLF12291; CASENT0923298 (CASC). ***Paratype.*** • 1w.; same data as for holotype, CASENT0059948 (CASC).

##### Other material.

Madagascar. –**Toliara**: • 3w.; Parc National d’Andohahela, Col du Sedro, 3.8 km 113°ESE Mahamavo, 37.6 km 341°NNW Tolagnaro; -24.76389, 46.75167; alt. 900 m; 21 Jan 2002; B. L. Fisher et al. leg.; montane rainforest, under stone; BLF05034 (CASC).

##### Diagnosis.

Moderately large species. ***Major workers.*** Head in full-face view sub-oval, widening posteriorly, with anterior and posterior sides convex, in lateral view sub-oval; ventral and dorsal faces convex; sides of the head very dense, short, suberect to erect pilosity; medial part of frons with thick, longitudinal, and dense rugae, interspaces predominantly smooth to indistinctly rugulate, rugae more irregular and directed slightly outward on posteromedial part; lateral sides with thick, dense, and irregular rugae with sparsely rugulate interspaces; area posterolateral from eyes without smooth notches; scape, when laid back, exceeding the midlength of head by three-fifths of its length; inner hypostomal teeth distinct, large, closely spaced, triangular, with rounded apex directed upward; outer hypostomal teeth lobe-like, distinctly lower and narrower than inner teeth; inner and outer hypostomal teeth closely spaced and not connected by concavity; mesosoma rugofoveolate; dorsal promesonotum and katepisternum with sparser rugofoveolae; gaster smooth with very dense pilosity; body orange. ***Minor workers.*** Head smooth; lateral sides of frons with longitudinal, short, and thick rugae; vertex with very short, sparse, and transverse rugae; scape, when laid back, exceeding the posterior head margin by two-fifths of its length; promesonotum moderately low and moderately long; promesonotal groove present; propodeal spines very small and triangular; mesosoma smooth; dorsal promesonotum with very sparse and transverse rugae; propodeum with sparse, irregular, and indistinct rugulae; body yellow.

##### Description.

**Major workers.** Measurements (*N* = 1): HL: 1.3; HW: 1.28; SL: 0.83; EL: 0.13; WL: 1.12; PSL: 0.2; MTL: 0.79; PNW: 0.55; PTW: 0.15; PPW: 0.34; CI: 101.5; SI: 64.9; PSLI: 15.6; PPI: 43.9; PNI: 43.1; MTI: 62.1.

***Head.*** In full-face view sub-oval, distinctly widening posteriorly, with anterior and posterior sides convex (Fig. 44B). In lateral view sub-oval; ventral and dorsal faces convex; inner hypostomal teeth visible. Sides of the head with very dense, short, suberect to erect pilosity; whole head with dense, long, decumbent to erect pilosity. Medial part of frons with thick, longitudinal and dense rugae, interspaces predominantly smooth to indistinctly rugulate, rugae more irregular and directed slightly outward on posteromedial part; lateral sides with thick, dense, and irregular rugae with sparsely rugulate interspaces. Occipital lobes with dense, irregular rugae and sparsely rugulate interspaces. Area posterolateral from eyes with weaker sculpture. Gena with relatively sparse, thick, and longitudinal rugae and distinctly rugulate interspaces. Centre of clypeus smooth and shiny, lateral sides with indistinct rugulae; median notch present, moderately wide, and shallow; median longitudinal carina absent; lateral longitudinal carinae absent. Scape, when laid back, exceeding the midlength of head by three-fifths of its length; pilosity subdecumbent to erect (Fig. 44B, D). Inner hypostomal teeth distinct, large, closely spaced, triangular, with rounded apex directed upward; outer hypostomal teeth lobe-like, distinctly lower and narrower than inner teeth; inner and outer hypostomal teeth closely spaced and not connected by concavity (Fig. 64B). ***Mesosoma.*** In lateral view, promesonotum short, angular, and moderately high, posterior mesonotum steep, mesonotal process indistinct, tubercle-like; promesonotal groove absent; metanotal groove indistinct; propodeal spines moderately long, with moderately wide base and acute apex; humeral area poorly produced (Fig. 44D). Surface shiny and rugofoveolate; dorsal promesonotum and katepisternum with sparser rugofoveolae. Pilosity sparse, long, and erect (Fig. 44D, F). ***Petiole.*** Shiny with sparse foveolae; node finely foveolate, triangular, with rounded and thick apex, in rear view node dorsoventrally convex; pilosity moderately sparse and erect (Fig. 44D, F). ***Postpetiole.*** Shiny and foveolate; dorsum with reduced sculpture and smooth notch; in dorsal view oval, lateral margins medially with two dentate projections; pilosity long, moderately sparse, and erect (Fig. 44D, F). ***Gaster.*** Shiny and smooth; pilosity very dense, moderately short, and erect (Fig. 44D, F). ***Colour.*** Yellowish orange with yellow legs and gaster (Fig. 44D, F).

**Minor workers.** Measurements (*N* = 4): HL: 0.64–0.66 (0.65); HW: 0.53–0.56 (0.55); SL: 0.68–0.75 (0.7); EL: 0.08–0.1 (0.09); WL: 0.81–0.84 (0.83); PSL: 0.08–0.1 (0.09); MTL: 0.57–0.61 (0.58); PNW: 0.37–0.38 (0.37); PTW: 0.09–0.1 (0.09); PPW: 0.13–0.15 (0.14); CI: 116.8–121.8 (118.5); SI: 124.1–140.8 (128.5); PSLI: 12.3–14.9 (14.0); PPI: 58.8–64.4 (62.7); PNI: 66.5–69.0 (68.1); MTI: 103.8–113.9 (106.7).

***Head.*** Cephalic margin slightly convex (Fig. 44A). Pilosity relatively dense, moderately long, decumbent to subdecumbent. Sculpture shiny and smooth; lateral sides of frons with longitudinal, short, and thick rugae; vertex with very short, sparse, and transverse rugae; antennal sockets with few thick, curved outward rugae and smooth interspaces. Clypeus with median longitudinal carina absent; two lateral longitudinal carinae absent. Scape, when laid back, exceeding the posterior head margin by two-fifths of its length; pilosity dense, suberect to erect (Fig. 44A, C). ***Mesosoma.*** In lateral view, promesonotum moderately low and moderately long, arched; promesonotal groove present; metanotal groove distinct; propodeal spines very small and triangular (Fig. 44C). Sculpture shiny and smooth; dorsal promesonotum with very sparse and transverse rugae; propodeum with sparse, irregular, and indistinct rugulae. Pilosity sparse, moderately long, and erect (Fig. 44C, E). ***Gaster.*** With dense, erect pilosity (Fig. 44C, E). ***Colour.*** Yellow (Fig. 44C, E).

##### Etymology.

Malagasy for yellow and hill in reference to body colouration and a habitat occupied by the species.

##### Biology.

The species was collected between 900–1075 m in elevation, in montane rainforest. Nests were located in rotten logs and under stones.

##### Comments.

*Pheidole mavohavoana* sp. nov. has very distinct major and minor workers. The species is known from two localities: Andrambovato in Fianarantsoa and Parc National d’Andohahela in Toliara. Morphologically *P. mavohavoana* sp. nov. is most similar to *P. antranohofa* sp. nov., a species known from a remote locality in Antsiranana. Major workers of *P. mavohavoana* sp. nov. differ from those of *P. antranohofa* sp. nov. in very dense, short, suberect to erect pilosity on sides of the head with, thick, dense, and irregular rugae with sparsely rugulate interspaces on lateral sides of frons, very dense pilosity on gaster, and reduced outer hypostomal teeth; minor workers can be separated based on predominantly smooth head lacking foveolae, moderately low and moderately long promesonotum, and dense pilosity on gaster.

#### Pheidole midongy sp. nov.

Taxon classificationAnimalia

A1F22F1F-8FAA-512A-9845-5C9D9DD18978

http://zoobank.org/100E9394-CB7C-4A53-84F1-D208EC6EBFC1

**Figure 45.**
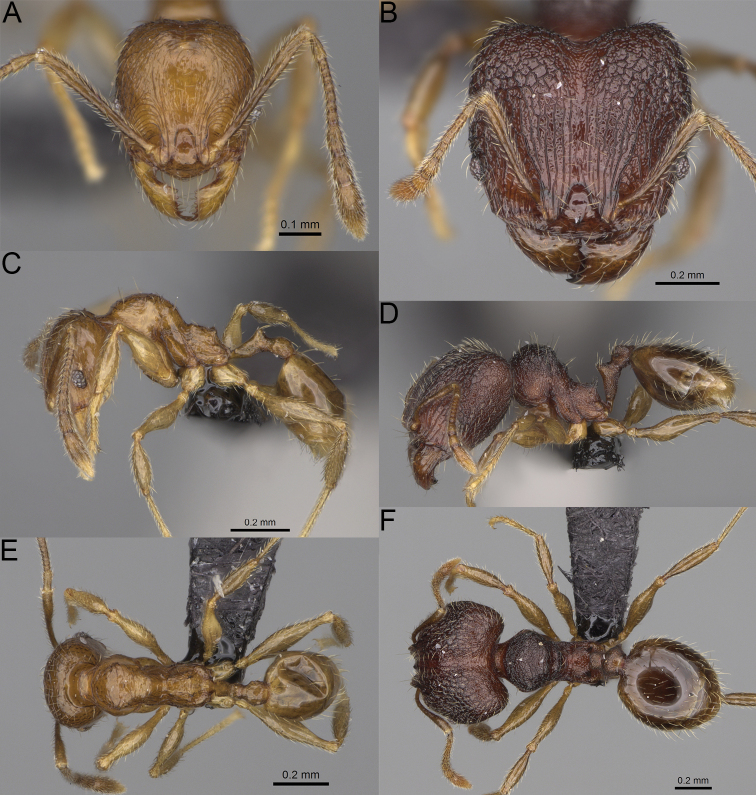
*Pheidole midongy* sp. nov., full-face view (**A**), profile (**C**), and dorsal view (**E**) of paratype minor worker (CASENT0923275) and full-face view (**B**), profile (**D**), and dorsal view (**F**) of holotype major worker (CASENT0119419).

Figs 45A–F , 64C , 66G

##### Type material.

***Holotype.*** Madagascar. • 1 major worker; Fianarantsoa; Parc National Befotaka-Midongy, Papango 27.7 km S Midongy-Sud, Mount Papango; -23.83517, 46.96367; alt. 940 m; 14 Nov 2006; B. L. Fisher et al. leg.; rainforest, ex rotten log; BLF14842; CASENT0119419 (CASC). ***Paratype.*** • 1w.; same data as for holotype, CASENT0923275 (CASC).

##### Diagnosis.

Minute species. ***Major workers.***HL < 0.9 mm and WL < 0.7 mm; head in full-face view sub-oval, slightly widening posteriorly, with anterior and posterior sides convex, in lateral view sub-oval; ventral and dorsal faces convex; body blackish brown; sides of head with moderately dense, moderately short, suberect pilosity; entire head distinctly sculptured, medial part of frons with thick, longitudinal, and dense rugae and distinctly rugulate interspaces, rugae more irregular and directed slightly outward on posteromedial part; scape, when laid back, exceeding the midlength of head by one-fifth of its length; mesosoma distinctly foveolate, promesonotum with additional sparse, thin, and irregular rugae on dorsum; katepisternum and propodeum with sparser foveolae; inner hypostomal teeth distinct, large, closely spaced, triangular, with rounded apex directed upward; outer hypostomal teeth lobe-like, wider than inner teeth and approximately the same height; inner and outer hypostomal teeth closely spaced and not connected by concavity; base of first gastral tergite smooth. ***Minor workers.***HL < 0.5 mm and WL < 0.6 mm, scape, when laid back, exceeding the posterior head margin by less than one-fifth of its length; propodeal spines reduced to small tubercles; head relatively oval; frons with sparse and moderately thick, short, and predominantly longitudinal rugulae fading medially; vertex with transverse, sparse, moderately thick, and short rugulae; area posterolateral from eyes smooth; body dark yellow; mesosoma smooth.

##### Description.

**Major workers.** Measurements (*N* = 1): HL: 0.89; HW: 0.91; SL: 0.5; EL: 0.12; WL: 0.74; PSL: 0.15; MTL: 0.46; PNW: 0.45; PTW: 0.12; PPW: 0.3; CI: 96.8; SI: 54.5; PSLI: 17.4; PPI: 40.5; PNI: 49.2; MTI: 50.0.

***Head.*** In full-face view sub-oval, slightly widening posteriorly, with anterior and posterior sides convex (Fig. 45B). In lateral view sub-oval; ventral and dorsal faces convex; inner hypostomal teeth visible. Sides of the head with moderately dense, moderately short, suberect pilosity; whole head with dense, long, decumbent to erect pilosity. Medial part of frons with thick, longitudinal, and dense rugae and distinctly rugulate interspaces, rugae more irregular and directed slightly outward on posteromedial part; lateral sides with thick, sparse, and irregular rugae with distinctly rugofoveolate interspaces. Occipital lobes with dense, irregular rugae and distinctly rugoreticulate interspaces. Area posterolateral from eyes with dense rugofoveolae and additional sparse and irregular rugae. Gena with relatively sparse and thick, longitudinal rugae and smooth to indistinctly rugoreticulate interspaces. Centre of clypeus smooth and shiny, lateral sides with indistinct rugulae; median notch present, moderately wide, and shallow; median longitudinal carina absent; lateral longitudinal carinae absent. Scape, when laid back, exceeding the midlength of head by one-fifth of its length; pilosity subdecumbent to erect (Fig. 45B, D). Inner hypostomal teeth distinct, large, closely spaced, triangular, with rounded apex directed upward; outer hypostomal teeth lobe-like, wider than inner teeth and approximately the same height; inner and outer hypostomal teeth closely spaced and not connected by concavity (Fig. 64C). ***Mesosoma.*** In lateral view, promesonotum short, angular, and moderately high, posterior mesonotum moderately steep, mesonotal process very indistinct, tubercle-like; promesonotal groove absent; metanotal groove absent; propodeal spines moderate, with wide base and acute apex; humeral area produced (Fig. 45D). Surface shiny and distinctly foveolate; promesonotum with additional sparse, thin, and irregular rugae on dorsum; katepisternum and propodeum with sparser foveolae. Pilosity moderately sparse, moderately long, and erect (Fig. 45D, F). ***Petiole.*** Shiny with dense foveolae; node finely foveolate, triangular, with rounded and thin apex, in rear view node dorsoventrally convex; pilosity moderately sparse and erect (Fig. 45D, F). ***Postpetiole.*** Shiny and foveolate; dorsum with reduced sculpture; in dorsal view oval, lateral margins medially with two dentate projections; pilosity long, moderately sparse, and erect (Fig. 45D, F). ***Gaster.*** Shiny and smooth; pilosity moderately dense, moderately short, and erect (Fig. 45D, F). ***Colour.*** Blackish brown with yellow legs and antenna (Fig. 45D, F).

**Minor workers.** Measurements (*N* = 1): HL: 0.48; HW: 0.42; SL: 0.44; EL: 0.09; WL: 0.57; PSL: 0.05; MTL: 0.34; PNW: 0.29; PTW: 0.06; PPW: 0.12; CI: 114.8; SI: 105.7; PSLI: 11.0; PPI: 50.8; PNI: 70.0; MTI: 81.4.

***Head.*** Cephalic margin slightly concave (Fig. 45A). Pilosity relatively sparse, moderately long, decumbent to subdecumbent. Sculpture shiny; frons with sparse and moderately thick, short, and predominantly longitudinal rugulae fading medially; vertex with transverse, sparse, moderately thick, and short rugulae; area posterolateral from eyes smooth; antennal sockets with few thick, curved outward rugae and smooth interspaces. Clypeus with median longitudinal carina absent; two lateral longitudinal carinae absent. Scape, when laid back, exceeding the posterior head margin by less than one-fifth of its length; pilosity dense, subdecumbent to erect (Fig. 45A, C). ***Mesosoma.*** In lateral view, promesonotum moderately high and short, arched; promesonotal groove absent; metanotal groove distinct; propodeal spines reduced to small tubercles (Fig. 45C). Sculpture smooth; promesonotal dorsum with very sparse, short, and transverse rugulae; propodeum with indistinct and irregular, sparse rugae. Pilosity sparse, moderately long, and erect (Fig. 45C, E). ***Gaster.*** With sparse, erect pilosity (Fig. 45C, E). ***Colour.*** Dark yellow (Fig. 45C, E).

##### Etymology.

From the type locality.

##### Biology.

The species was collected at 940 m in elevation, in rainforest. Nest was located in a rotten log.

##### Comments.

*Pheidole midongy* sp. nov. belongs to the group of species characterised by small body size (major workers: HL < 1.05 mm, WL < 0.9 mm and minor workers HL < 0.5 mm, WL < 0.6 mm), sub-oval, slightly widening posteriorly head with anterior and posterior sides convex in major workers, and minor workers with yellow to brown body colouration and head foveolate or predominantly smooth and relatively oval. The group includes six species: *P. havoana* sp. nov., *P. kely* sp. nov., *P. parvula* sp. nov., *P. parvulogibba* sp. nov., *P. volontany* sp. nov., and *P. midongy* sp. nov. Because of dark body colouration *P. midongy* sp. nov., described form Parc National Befotaka-Midongy in Fianarantsoa, is most similar to *P. volontany* sp. nov., known from Forêt Classée d’Analavelona in Toliara. Major workers of *P. midongy* sp. nov. differ from *P. volontany* sp. nov. in frons with thick, longitudinal, and dense rugae and distinctly rugulate interspaces, and sides of the head with moderately dense, moderately short, and suberect pilosity; minor workers differ in frons with sparse and moderately thick, short, and predominantly longitudinal rugulae, vertex with transverse, sparse, and moderately thick short rugulae, oval head shape, and brighter body colouration.

#### Pheidole mikros sp. nov.

Taxon classificationAnimalia

E90F2730-7B79-52DA-A835-01D9311E645B

http://zoobank.org/6378BC54-2DA8-44F8-9768-7210E473D2CF

**Figure 46.**
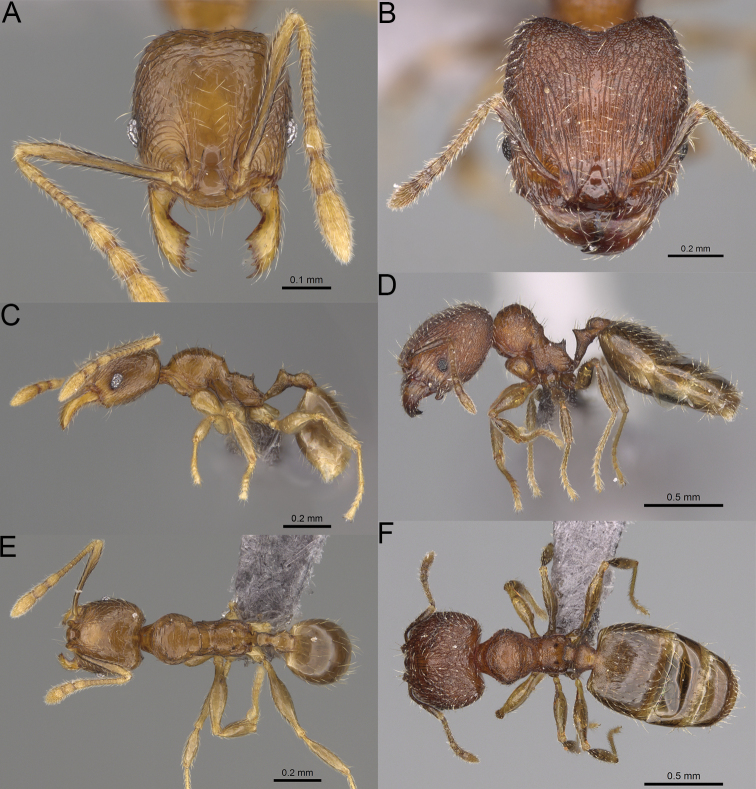
*Pheidole mikros* sp. nov., full-face view (**A**), profile (**C**), and dorsal view (**E**) of paratype minor worker (CASENT0261101) and full-face view (**B**), profile (**D**), and dorsal view (**F**) of holotype major worker (CASENT0923271).

Figs 46A–F , 64D , 66H

##### Type material.

***Holotype.*** Madagascar. • 1 major worker; Antsiranana; Sakaramy; -12.44131, 49.22723; alt. 365 m; 13 May 2011; B. L. Fisher et al. leg.; tropical dry forest, under tree bark, live tree; BLF27321; CASENT0923271 (CASC). ***Paratype.*** • 1w.; same data as for holotype, CASENT0261101 (CASC).

##### Other material.

Madagascar. –**Antsiranana**: •1w.; Parc National de Marojejy, Manantenina River, 28.0 km 38°NE Andapa, 8.2 km 333°NNW Manantenina; -14.43667, 49.775; alt. 450 m; 23 Nov 2004; B. L. Fisher et al. leg.; rainforest, ex rotten log; BLF10982 (CASC). •1w., 1s.; Réserve Spéciale de l’Ankarana, 22.9 km 224°SW Anivorano Nord; -12.90889, 49.10983; alt. 80 m; 10 Feb 2001; B. L. Fisher et al. leg.; tropical dry forest, ex rotten log; BLF03008 (CASC).

##### Diagnosis.

Minute species. ***Major workers.***HL < 0.9 mm and WL < 0.75 mm; head in full-face view elongate, not widening posteriorly with posterior sides slightly convex, in lateral view sub-oval and short with convex dorsal and ventral sides; body brownish orange; sides of head with moderately sparse, moderately long, subdecumbent to suberect pilosity; entire head distinctly sculptured, medial part of frons with thick and longitudinal rugae and indistinctly rugulate and never smooth interspaces, occipital lobes with dense and irregular rugae with distinctly rugoreticulate interspaces; scape, when laid back, slightly exceeding the midlength of head; lateral sides of promesonotum distinctly foveolate with additional sparse and indistinct rugulae; inner hypostomal teeth large, closely spaced, triangular, with rounded apex directed upward; outer hypostomal teeth lobe-like, slightly lower and narrower than inner teeth; inner and outer hypostomal teeth moderately closely spaced and not connected by concavity; base of first gastral tergite shagreened. ***Minor workers.***HL < 0.5 mm and WL < 0.6 mm, scape, when laid back, surpassing the posterior head margin by one-fifth of its length; propodeal spines small, triangular; head relatively rectangular; body yellowish brown; lateral sides of frons with fine, sparse, and irregular rugulae with smooth interspaces; vertex with distinct, sparse, and transverse rugulae; mesosoma with sparse and moderately thick network of irregular rugulae; mesonotum and katepisternum smooth.

##### Description.

**Major workers.** Measurements (*N* = 2): HL: 0.86, 0.88; HW: 0.8, 0.83; SL: 0.49, 0.47; EL: 0.12, 0.11; WL: 0.74, 0.77; PSL: 0.12, 0.13; MTL: 0.44, 0.41; PNW: 0.46, 0.46; PTW: 0.13, 0.14; PPW: 0.35, 0.37; CI: 107.6, 105.2; SI: 61.7, 56.5; PSLI: 13.9, 15.0; PPI: 37.8, 38.7; PNI: 57.3, 55.6; MTI: 54.8, 49.7.

***Head.*** In full-face view elongate, not widening posteriorly, with anterior and posterior sides slightly convex (Fig. 46B). In lateral view sub-oval; ventral and dorsal faces convex; inner hypostomal teeth visible. Sides of the head with moderately sparse, moderately long, subdecumbent to suberect pilosity; whole head with dense, long, decumbent to erect pilosity. Medial part of frons with thick, longitudinal, and dense rugae and indistinctly rugulate interspaces, rugae more irregular and directed slightly outward on posteromedial part; lateral sides with thick, sparse, and predominantly longitudinal rugae, interspaces shiny and distinctly foveolate. Occipital lobes with denser, irregular rugae and distinctly foveolate interspaces. Area posterolateral from eyes foveolate with sparse and moderately thick rugae. Gena with relatively sparse, thick, and longitudinal rugae and indistinctly foveolate interspaces. Centre of clypeus smooth and shiny, lateral sides with indistinct rugulae; median notch present, moderately wide, and shallow; median longitudinal carina absent; lateral longitudinal carinae absent. Scape, when laid back, slightly exceeding the midlength of head; pilosity subdecumbent to erect (Fig. 46B, D). Inner hypostomal teeth distinct, large, closely spaced, triangular, with rounded apex directed upward; outer hypostomal teeth lobe-like, slightly lower and narrower than inner teeth; inner and outer hypostomal teeth moderately closely spaced and not connected by concavity (Fig. 64D). ***Mesosoma.*** In lateral view, promesonotum short, angular, and moderately low, posterior mesonotum moderately steep, mesonotal process very indistinct, tubercle-like; promesonotal groove absent; metanotal groove absent; propodeal spines moderately long, with wide base and acute apex; humeral area laterally weakly produced (Fig. 46D). Surface shiny and distinctly foveolate with additional sparse and indistinct rugulae on promesonotum. Pilosity moderately sparse, moderately long, and erect (Fig. 46D, F). ***Petiole.*** Shiny with dense foveolae; node smooth to finely foveolate, triangular, with rounded and thick apex, in rear view node dorsoventrally concave; pilosity moderately sparse and erect (Fig. 46D, F). ***Postpetiole.*** Shiny and foveolate; dorsum with slightly reduced sculpture; in dorsal view trapezoid, lateral margins medially with two large dentate lobes; pilosity long, moderately sparse, and erect (Fig. 46D, F). ***Gaster.*** Shiny, its base shagreened; pilosity dense, moderately short, and erect (Fig. 46D, F). ***Colour.*** Brownish orange; mandibles and gaster brown (Fig. 46D, F).

**Minor workers.** Measurements (*N* = 3): HL: 0.47–0.48 (0.47); HW: 0.4–0.42 (0.41); SL: 0.42–0.43 (0.43); EL: 0.08–0.08 (0.08); WL: 0.54–0.56 (0.55); PSL: 0.07–0.07 (0.07); MTL: 0.3–0.31 (0.31); PNW: 0.29–0.3 (0.29); PTW: 0.07–0.07 (0.07); PPW: 0.12–0.13 (0.12); CI: 110.8–117.7 (115.1); SI: 101.9–105.4 (104.2); PSLI: 14.4–15.5 (15.0); PPI: 54.7–57.5 (56.4); PNI: 67.7–73.7 (71.6); MTI: 73.4–76.0 (74.4).

***Head.*** Cephalic margin indistinctly concave to straight (Fig. 46A). Pilosity relatively dense, moderately long, decumbent to suberect. Sculpture shiny; medial frons smooth; lateral sides of frons with fine, sparse, and irregular rugulae with smooth interspaces; vertex with distinct, sparse, and transverse rugulae; area posterolateral from eyes with fine, sparse, and irregular rugulae with smooth interspaces; antennal sockets with few thick, curved outward rugae and indistinctly foveolate interspaces. Clypeus with median longitudinal carina absent; two lateral longitudinal carinae absent. Scape, when laid back, surpassing the posterior head margin by one-fifth of its length; pilosity dense, subdecumbent to erect (Fig. 46A, C). ***Mesosoma.*** In lateral view, promesonotum moderately high and short, arched; promesonotal groove absent; metanotal groove distinct; propodeal spines small and triangular (Fig. 46C). Sculpture shiny with sparse and moderately thick network of irregular rugulae; mesonotum and katepisternum smooth. Pilosity moderately sparse, moderately long, and erect (Fig. 46C, E). ***Gaster.*** With sparse, erect pilosity (Fig. 46C, E). ***Colour.*** Yellowish brown, legs and antenna yellow (Fig. 46C, E).

##### Etymology.

Greek for small in reference to the body size.

##### Biology.

The species was collected between 80–450 m in elevation, in rainforest and tropical dry forest. Nests were located in rotten logs and under tree bark.

##### Comments.

*Pheidole mikros* sp. nov. belongs to the group of species characterised by small body size (major workers: HL < 1.05 mm, WL < 0.9 mm and minor workers HL < 0.5 mm, WL < 0.6 mm), head elongate and not widening posteriorly in major workers, and minor workers with head predominantly smooth and relatively rectangular, and body colouration yellow to brown. The group includes four species: *P. flavominuta* sp. nov., *P. nitidobruna* sp. nov., *P. mikros* sp. nov., and *P. beanka* sp. nov. *Pheidole mikros* sp. nov. is known from the area between Andapa and Antisiranana and is parapatric with *Pheidole nitidobruna* sp. nov. described from Makirovana forest in Antsiranana. Major workers of *P. mikros* sp. nov. can be separated from *P. nitidobruna* sp. nov. by predominantly indistinctly rugulate interspaces on frons, occipital lobes with distinctly foveolate or rugoreticulae interspaces, and absence of smooth notches on lateral sides of promesonotum. Minor workers of *P. mikros* sp. nov. can be separated from *P. nitidobruna* sp. nov. based on presence of sculpture on vertex and frons and presence of small, triangular propodeal spines. However, the morphology of *P. mikros* sp. nov. is most similar to *P. beanka* sp. nov., which is known from Réserve forestière Beanka in Mahajanga. *Pheidole mikros* sp. nov. differs from *P. beanka* sp. nov. by the combination of the following characters: major workers have medial part of frons with indistinctly rugulate interspaces and their propodeal spines are moderately long; minor workers have lateral sides of frons with fine, sparse, and irregular rugulae, mesosoma covered by a sparse and moderately thick network of irregular rugulae, and body is yellowish brown.

#### Pheidole mivory sp. nov.

Taxon classificationAnimalia

F0758B87-C1BB-5492-A3D7-61F0C27723BB

http://zoobank.org/073177E4-C6C2-489D-A764-01F0B3EB918D

**Figure 47.**
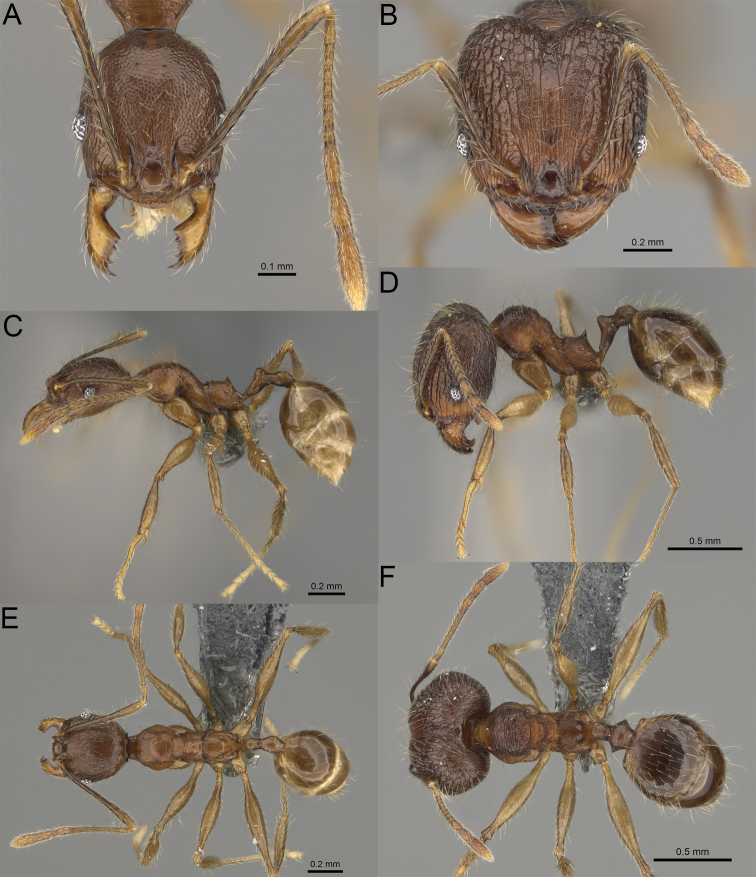
*Pheidole mivory* sp. nov., full-face view (**A**), profile (**C**), and dorsal view (**E**) of paratype minor worker (CASENT0923288) and full-face view (**B**), profile (**D**), and dorsal view (**F**) of holotype major worker (CASENT0825256).

Figs 47A–F , 64E , 66I

##### Type material.

***Holotype.*** Madagascar. • 1 major worker; Antsiranana; Sava Region: Parc National de Marojejy, near Manantenina tributary, 28.3 km 28.5°NE Andapa, forest along trail below Camp 1; -14.43934, 49.77689; alt. 450 m; 8 Feb 2018; B. L. Fisher et al. leg.; rainforest, ex rotten log; BLF40787; CASENT0825256 (CASC). ***Paratypes.*** • 2w., 1m.; same data as for holotype, CASENT0825255, CASENT0923288 (CASC, MHNG).

##### Diagnosis.

Moderately large species. ***Major workers.*** Head in full-face view sub-oval and slightly widening posteriorly, with anterior and posterior sides slightly convex, in lateral view sub-oval; ventral and dorsal faces convex; sides of the head with moderately sparse, long, suberect to erect pilosity; with thick, interrupted, dense, and longitudinal rugae, posteromedial frons with rugae irregular, interspaces with sparse rugofoveolate; lateral sides of frons with dense, thick, predominantly irregular rugae with few distinct, longitudinal rugae, interspaces with sparse rugulae; occipital lobes, area posterolateral from eyes without smooth notches; scape, when laid back, exceeding the midlength of head by two-fifths of its length; inner hypostomal teeth distinct, large, closely spaced, triangular, with rounded apex directed upward; outer hypostomal teeth lobe-like, wider than and approximately as high as inner teeth, apex directed upward; inner and outer hypostomal teeth closely spaced and not connected by concavity; mesosoma rugofoveolate; pronotum with additional thin, moderately dense, and transverse rugae; gaster smooth with slightly shagreened base of first tergite; body brown. ***Minor workers.*** Head foveolate; median frons with short and indistinct longitudinal rugulae; area posterolateral from eyes with weaker sculpture; scape, when laid back, exceeding the posterior head margin by one-third of its length; promesonotum low and moderately long, arched; promesonotal groove absent; propodeal spines small and triangular; mesosoma with sparse foveolae; dorsal promesonotum and medial parts of lateral sides of pronotum, propodeum, and katepisternum with smooth notches; body brown.

##### Description.

**Major workers.** Measurements (*N* = 1): HL: 1.03; HW: 1.0; SL: 0.74; EL: 0.15; WL: 0.96; PSL: 0.14; MTL: 0.65; PNW: 0.41; PTW: 0.13; PPW: 0.3; CI: 102.5; SI: 73.8; PSLI: 13.5; PPI: 42.8; PNI: 40.9; MTI: 65.3.

***Head.*** In full-face sub-oval, slightly widening posteriorly, with anterior and posterior sides slightly convex (Fig. 47B). In lateral view sub-oval; ventral and dorsal faces convex; inner hypostomal teeth visible. Sides of the head with moderately sparse, long, suberect to erect pilosity; whole head with dense, long, decumbent to erect pilosity. Anteromedial part of frons with thick, interrupted, dense, and longitudinal rugae, posteromedial frons with irregular rugae, interspaces with sparse rugofoveolate; lateral sides of frons with dense, thick, and predominantly irregular rugae with few distinct longitudinal rugae, interspaces with sparse rugulae. Occipital lobes with irregular and thinner rugae and indistinctly rugulate interspaces. Area posterolateral from eyes with sparse, thick, longitudinal rugae and distinctly rugofoveolate interspaces. Gena with relatively sparse, thick, longitudinal rugae indistinctly rugulate interspaces. Centre of clypeus smooth and shiny, lateral sides with indistinct rugulae; median notch present, moderately wide, and shallow; median longitudinal carina present; lateral longitudinal carinae absent. Scape, when laid back, exceeding the midlength of head by two-fifths of its length; pilosity subdecumbent to erect (Fig. 47B, D). Inner hypostomal teeth distinct, large, closely spaced, triangular, with rounded apex directed upward; outer hypostomal teeth lobe-like, wider than and approximately as high as inner teeth, apex directed upward; inner and outer hypostomal teeth closely spaced and not connected by concavity (Fig. 64E). ***Mesosoma.*** In lateral view, promesonotum short, angular, and moderately low, posterior mesonotum moderately steep, mesonotal process indistinct, tubercle-like; promesonotal groove absent; metanotal groove indistinct; propodeal spines moderate, with wide base and acute apex; humeral area weakly produced (Fig. 47D). Surface shiny and rugofoveolate; pronotum with additional thin, moderately dense, and transverse rugae. Pilosity moderately dense, long, and erect (Fig. 47D, F). ***Petiole.*** Shiny with dense foveolae; node finely foveolate, triangular, with rounded and thick apex, in rear view node dorsoventrally slightly convex; pilosity moderately sparse and erect (Fig. 47D, F). ***Postpetiole.*** Shiny and foveolate; dorsum with reduced sculpture and smooth notch; in dorsal view oval, lateral margins medially with two dentate projections; pilosity long, moderately sparse, and erect (Fig. 47D, F). ***Gaster.*** Shiny and smooth with slightly shagreened base of first tergite; pilosity moderately dense, long, and erect (Fig. 47D, F). ***Colour.*** Brown, gaster slightly darker, legs yellowish (Fig. 47D, F).

**Minor workers.** Measurements (*N* = 2): HL: 0.56, 0.61; HW: 0.47, 0.48; SL: 0.74, 0.75; EL: 0.11, 0.12; WL: 0.75, 0.75; PSL: 0.09, 0.09; MTL: 0.54, 0.57; PNW: 0.32, 0.33; PTW: 0.08, 0.08; PPW: 0.13, 0.12; CI: 119.1, 126.6; SI: 156.8, 155.4; PSLI: 15.2, 14.5; PPI: 66.7, 66.1; PNI: 68.5, 69.5; MTI: 115.1, 119.7.

***Head.*** Cephalic margin slightly convex (Fig. 47A). Pilosity relatively sparse, moderately long, subdecumbent to erect. Sculpture shiny and foveolate; median frons with short and indistinct longitudinal rugulae; area posterolateral from eyes with weaker sculpture; antennal sockets with few indistinct, curved outward rugae and foveolate interspaces. Clypeus with median longitudinal carina absent; two lateral longitudinal carinae absent. Scape, when laid back, exceeding the posterior head margin by one-third of its length; pilosity dense, subdecumbent to erect (Fig. 47A, C). ***Mesosoma.*** In lateral view, promesonotum low and moderately long, arched; promesonotal groove absent; metanotal groove distinct; propodeal spines small and triangular (Fig. 47C). Sculpture shiny with sparse foveolae; dorsal promesonotum and medial parts of lateral sides of pronotum, propodeum, and katepisternum with smooth notches. Pilosity very sparse, moderately long, and erect (Fig. 47C, E). ***Gaster.*** With sparse, erect pilosity (Fig. 47C, E). ***Colour.*** Brown, legs yellowish (Fig. 47C, E).

##### Etymology.

Malagasy for watercourse in reference to the river located close to the sampling site of the species.

##### Biology.

The species was collected at 450 m in elevation, in rainforest. Nest was located in a rotten log.

##### Comments.

*Pheidole mivory* sp. nov., described from Parc National de Marojejy in Antsiranana, has major workers with dense and thick rugae that are anteromedially longitudinal and posteromedially irregular, with distinctly rugofoveolate interspaces, and brown body colouration. Morphologically it is most similar to the parapatric *P. joffreville* sp. nov., known from Parc National Montagne d’Ambre in Antsiranana. Majors of both taxa are extremely similar and species separation should be supported by or based exclusively on minors. Majors of *P. mivory* sp. nov. differ from *P. joffreville* sp. nov. in indistinctly rugofoveolate interspaces on frons, and presence of longitudinal rugae on lateral sides of frons, and indistinctly shagreened sculpture of first gastral tergite; minor workers differ in mesosoma with sparse foveolae with dorsal promesonotum and medial parts of lateral sides of pronotum, propodeum, and katepisternum with smooth notches.

#### Pheidole nitidobruna sp. nov.

Taxon classificationAnimalia

71E81728-B37D-5C03-97B5-B5BEFD3A3F0D

http://zoobank.org/4676573B-D010-4733-BB04-D4F3AB90022E

**Figure 48.**
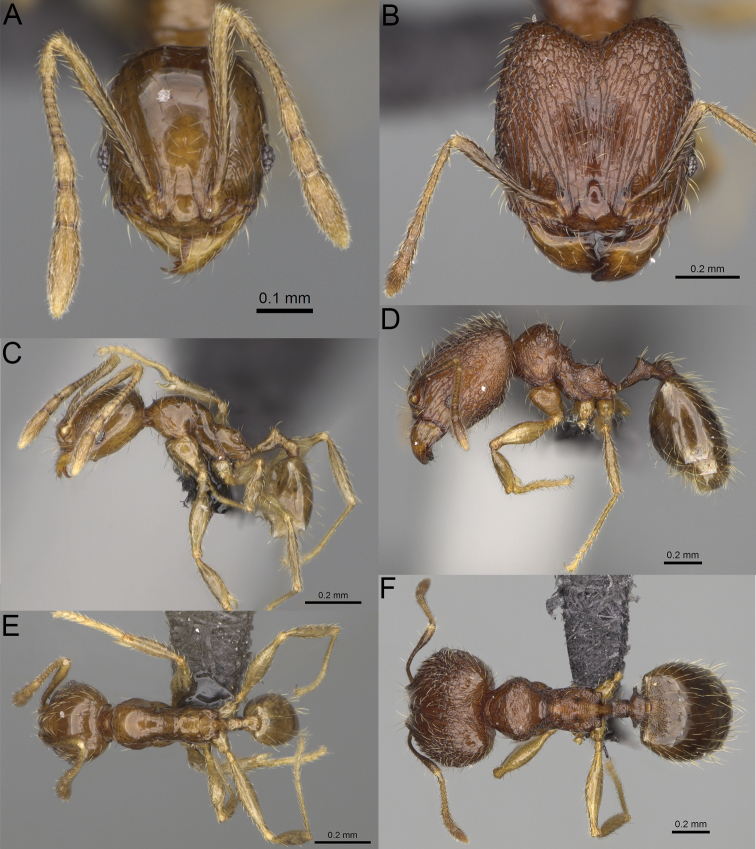
*Pheidole nitidobruna* sp. nov., full-face view (**A**), profile (**C**), and dorsal view (**E**) of paratype minor worker (CASENT0245024) and full-face view (**B**), profile (**D**), and dorsal view (**F**) of holotype major worker (CASENT0923273).

Figs 48A–F , 64F , 66J

##### Type material.

***Holotype.*** Madagascar. • 1 major worker; Antsiranana; Makirovana forest; -14.16044, 49.95216; alt. 550 m; 1 May 2011; B. L. Fisher et al. leg.; rainforest, ex rotten log; BLF26855; CASENT0923273 (CASC). ***Paratype.*** • 1w.; same data as for holotype, CASENT0245024 (CASC).

##### Diagnosis.

Minute species. ***Major workers.***HL < 0.9 mm and WL < 0.8 mm; head in full-face view elongate, not widening posteriorly with posterior sides slightly convex, in lateral view sub-oval and short with convex dorsal and ventral sides; body brown; sides of head with moderately sparse, moderately long, suberect to erect pilosity; entire head distinctly sculptured, medial part of frons with thick and longitudinal rugae and smooth to indistinctly rugulate interspaces, occipital lobes with dense and irregular rugae with indistinctly rugoreticulate interspaces; scape, when laid back, exceeding the midlength of head by one-fifth of its length; lateral sides of promesonotum with smooth notches; inner hypostomal teeth distinct, moderate, closely spaced, triangular, with rounded apex directed upward; outer hypostomal teeth lobe-like, lower and wider than inner teeth; inner and outer hypostomal teeth closely spaced and not connected by concavity; base of first gastral tergite shagreened. ***Minor workers.***HL < 0.45 mm and WL < 0.5 mm, scape, when laid back, exceeding the posterior head margin by one-fifth of its length; propodeal spines reduced to small tubercles; head relatively rectangular; body yellowish brown; head with frons and vertex smooth; mesosoma smooth.

##### Description.

**Major workers.** Measurements (*N* = 1): HL: 0.9; HW: 0.79; SL: 0.53; EL: 0.1; WL: 0.79; PSL: 0.11; MTL: 0.46; PNW: 0.44; PTW: 0.11; PPW: 0.28; CI: 113.1; SI: 66.7; PSLI: 12.7; PPI: 37.9; PNI: 55.8; MTI: 57.4.

***Head.*** In full-face view elongate, not widening posteriorly, with anterior and posterior sides slightly convex (Fig. 48B). In lateral view sub-oval; ventral and dorsal faces convex; inner hypostomal teeth visible. Sides of the head with moderately sparse, moderately long, suberect to erect pilosity; whole head with dense, long, decumbent to erect pilosity. Medial part of frons with thick, longitudinal, and moderately dense rugae and smooth to indistinctly rugulate interspaces, rugae directed slightly outward on posteromedial part; anterolateral sides with thick, sparse, and longitudinal rugae with smooth to indistinctly rugulate interspaces, posterolateral sides with dense, thick, and irregular rugae with distinctly rugulae interspaces. Occipital lobes with dense, irregular rugae and indistinctly rugoreticulate interspaces. Area posterolateral from eyes with weaker sculpture, rugoreticulate. Gena with relatively sparse, thick, and longitudinal rugae and smooth interspaces. Centre of clypeus smooth and shiny, lateral sides with indistinct rugulae; median notch present, moderately wide, and shallow; median longitudinal carina absent; lateral longitudinal carinae absent. Scape, when laid back, exceeding the midlength of head by one-fifth of its length; pilosity subdecumbent to erect (Fig. 48B, D). Inner hypostomal teeth distinct, moderate, closely spaced, triangular, with rounded apex directed upward; outer hypostomal teeth lobe-like, lower and wider than inner teeth; inner and outer hypostomal teeth closely spaced and not connected by concavity (Fig. 64F). ***Mesosoma.*** In lateral view, promesonotum short, angular, and moderately high, posterior mesonotum moderately steep, mesonotal process very indistinct, tubercle-like; promesonotal groove absent; metanotal groove absent; propodeal spines short, with wide base and acute apex; humeral area produced (Fig. 48D). Surface shiny; promesonotum with sparse, thick to moderately thick, irregular rugae and foveolate interspaces, lateral sides with smooth notches; anepisternum, katepisternum, and propodeum with denser rugofoveolae. Pilosity moderately sparse, moderately long, and erect (Fig. 48D, F). ***Petiole.*** Shiny with dense foveolae; node finely foveolate, triangular, with rounded and thin apex, in rear view node dorsoventrally concave; pilosity moderately sparse and erect (Fig. 48D, F). ***Postpetiole.*** Shiny and foveolate; dorsum with reduced sculpture and smooth notch; in dorsal view trapezoid, lateral margins medially with two distinct dentate projections; pilosity long, moderately sparse, and erect (Fig. 48D, F). ***Gaster.*** Shiny and smooth with shagreened base; pilosity moderately dense, moderately short, and erect (Fig. 48D, F). ***Colour.*** Brown with yellow legs (Fig. 48D, F).

**Minor workers.** Measurements (*N* = 1): HL: 0.44; HW: 0.38; SL: 0.42; EL: 0.08; WL: 0.5; PSL: 0.03; MTL: 0.31; PNW: 0.25; PTW: 0.06; PPW: 0.08; CI: 117.2; SI: 110.3; PSLI: 7.4; PPI: 75.4; PNI: 67.0; MTI: 82.3.

***Head.*** Cephalic margin indistinctly relatively straight (Fig. 48A). Pilosity relatively sparse, moderately long, decumbent to suberect. Sculpture shiny and smooth; antennal sockets with few thick, curved outward rugae and smooth interspaces. Clypeus with median longitudinal carina absent; two lateral longitudinal carinae absent. Scape, when laid back, exceeding the posterior head margin by one-fifth of its length; pilosity dense, subdecumbent to erect (Fig. 48A, C). ***Mesosoma.*** In lateral view, promesonotum moderately high and short, arched; promesonotal groove absent; metanotal groove indistinct; propodeal spines reduced to small tubercles (Fig. 48C). Sculpture shiny and smooth. Pilosity moderately sparse, moderately long, and erect (Fig. 48C, E). ***Gaster.*** With sparse, erect pilosity (Fig. 48C, E). ***Colour.*** Yellowish brown, legs and antenna yellow (Fig. 48C, E).

##### Etymology.

Latin for smooth and brown in reference to smooth sculpture and dark body colouration.

##### Biology.

The species was collected at 550 m in elevation, in rainforest. Nest was located in a rotten log.

##### Comments.

*Pheidole nitidobruna* sp. nov. belongs to the group of species characterised by small body size (major workers: HL < 1.05 mm, WL < 0.9 mm and minor workers HL < 0.5 mm, WL < 0.6 mm), major workers with head elongate and not widening posteriorly, and minor workers with predominantly smooth and relatively rectangular head and yellow to brown body colouration. The group includes four species: *P. flavominuta* sp. nov., *P. nitidobruna* sp. nov., *P. mikros* sp. nov., and *P. beanka* sp. nov. *Pheidole nitidobruna* sp. nov. is known only from Makirovana forest in Antsiranana and is parapatric with *P. mikros* sp. nov., which is recorded from the area between Andapa and Antisiranana. Major workers of *P. nitidobruna* sp. nov. can be separated from *P. mikros* sp. nov. by smooth to indistinctly rugulate interspaces on frons and occipital lobes and presence of smooth notches on lateral sides of promesonotum. Minor workers of *P. nitidobruna* sp. nov. can be separated from *P. mikros* sp. nov. based on entirely smooth vertex and frons and presence of propodeal spines reduced to small tubercles.

#### Pheidole parvula sp. nov.

Taxon classificationAnimalia

643CAC9C-316D-5003-882B-FEEF10CDF6E7

http://zoobank.org/96E7640F-935D-4C1E-B984-A94180145035

**Figure 49.**
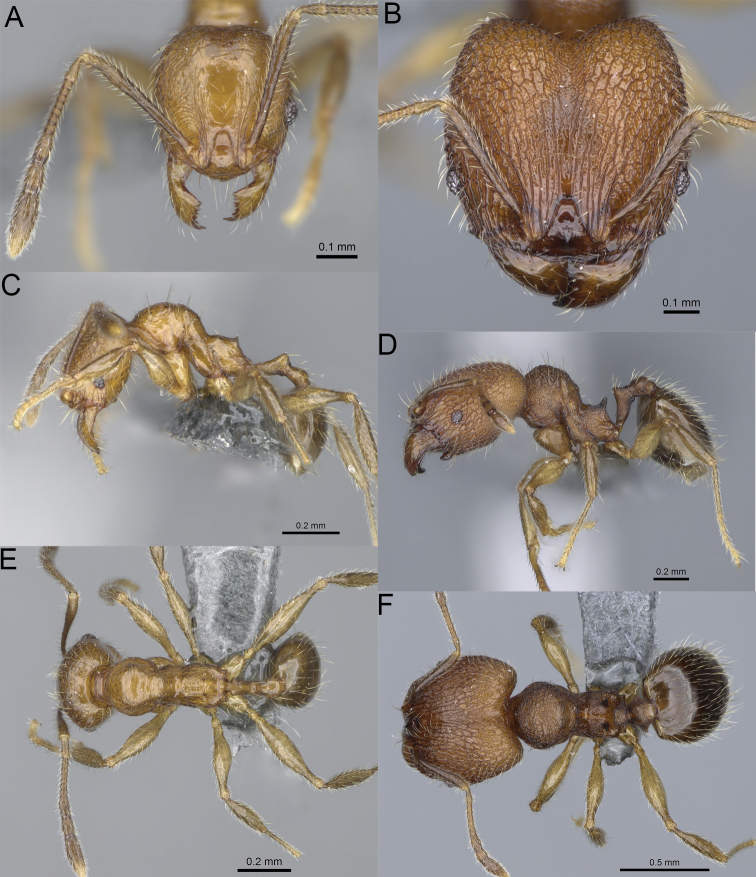
*Pheidole parvula* sp. nov., full-face view (**A**), profile (**C**), and dorsal view (**E**) of paratype minor worker (CASENT0923270) and full-face view (**B**), profile (**D**), and dorsal view (**F**) of holotype major worker (CASENT0274865).

Figs 49A–F , 64G , 66K

##### Type material.

***Holotype.*** Madagascar. • 1 major worker; Toamasina; Ankerana; -18.40062, 48.81311; alt. 865 m; 17 Jan 2012; B. L. Fisher et al. leg.; rainforest, ex rotten log; BLF27754; CASENT0274865 (CASC). ***Paratype.*** • 1w.; same data as for holotype, CASENT0923270 (CASC).

##### Other material.

Madagascar. –**Antananarivo**: •1w., 1s.; Réserve Speciale d’Ambohitantely; -18.22444, 47.2774; alt. 1490 m; 9 Mar 2012; B. L. Fisher et al. leg.; montane forest, ex litter; BLF28300 (CASC). –**Toamasina**: •1w., 1q.; Ankerana; -18.40829, 48.82107; alt. 750 m; 21 Jan 2012; B. L. Fisher et al. leg.; rainforest, ex rotten log; BLF27962 (CASC).

##### Diagnosis.

Minute species. ***Major workers.***HL < 0.9 mm and WL < 0.8 mm; head in full-face view sub-oval, slightly widening posteriorly, with anterior and posterior sides convex, in lateral view sub-oval; ventral and dorsal faces convex; body dark orange; sides of head with moderately dense, moderately long, suberect pilosity; entire head distinctly sculptured, medial part of frons with thick and dense rugae and distinctly rugulate interspaces, rugae predominantly irregular and only centre and basal parts with few longitudinal rugulae; scape, when laid back, exceeding the midlength of head by one-fifth of its length; pronotum distinctly foveolate with additional irregular rugae on dorsum; anepisternum, katepisternum, and propodeum with fine rugulae and smooth interspaces; inner hypostomal teeth distinct, large, closely spaced, triangular, with rounded apex directed upward; outer hypostomal teeth lobe-like, slightly lower and wider than inner teeth; inner and outer hypostomal teeth moderately closely spaced and not connected by concavity; base of first gastral tergite smooth. ***Minor workers.***HL < 0.5 mm and WL < 0.6 mm, scape, when laid back, surpassing the posterior head margin by one-fifth of its length; propodeal spines small and triangular; head relatively oval; body dark yellow; medial frons smooth; lateral sides of frons and vertex with fine, sparse, and irregular rugulae with smooth interspaces; area posterolateral from eyes smooth; mesosoma predominantly smooth with very sparse, fine, and irregular rugulae.

##### Description.

**Major workers.** Measurements (*N* = 2): HL: 0.86, 0.91; HW: 0.88, 0.9; SL: 0.49, 0.51; EL: 0.11, 0.12; WL: 0.79, 0.83; PSL: 0.15, 0.16; MTL: 0.48, 0.46; PNW: 0.43, 0.43; PTW: 0.09, 0.15; PPW: 0.28, 0.32; CI: 97.6, 101.7; SI: 56.1, 57.1; PSLI: 17.8, 17.8; PPI: 31.8, 45.3; PNI: 48.5, 48.2; MTI: 54.3, 51.7.

***Head.*** In full-face view sub-oval, slightly widening posteriorly, with anterior and posterior sides convex (Fig. 49B). In lateral view sub-oval; ventral and dorsal faces convex; inner hypostomal teeth visible. Sides of the head with moderately dense, moderately long, suberect pilosity; whole head with dense, long, decumbent to erect pilosity. Medial part of frons with thick and dense rugae and distinctly rugulate interspaces, rugae predominantly irregular and only centre and basal parts with few longitudinal rugulae; lateral sides with thick, dense, and irregular rugae, interspaces shiny and distinctly rugulate. Occipital lobes with denser, irregular rugae and distinctly rugofoveolate interspaces. Area posterolateral from eyes with thick, dense, and irregular rugae, interspaces shiny and distinctly rugulate, posteriormost part distinctly foveolate with few thick, irregular rugae. Gena with relatively sparse, thick, and longitudinal rugae and indistinctly rugoreticulate interspaces. Centre of clypeus smooth and shiny, lateral sides with indistinct rugulae; median notch present, moderately wide, and shallow; median longitudinal carina present; lateral longitudinal carinae absent. Scape, when laid back, exceeding the midlength of head by one-fifth of its length; pilosity subdecumbent to erect (Fig. 49B, D). Inner hypostomal teeth distinct, large, closely spaced, triangular, with rounded apex directed upward; outer hypostomal teeth lobe-like, slightly lower and wider than inner teeth; inner and outer hypostomal teeth moderately closely spaced and not connected by concavity (Fig. 64G). ***Mesosoma.*** In lateral view, promesonotum short, angular, and moderately low, posterior mesonotum moderately steep, mesonotal process very indistinct, tubercle-like, or absent; promesonotal groove absent; metanotal groove absent; propodeal spines long, with moderately narrow base and acute apex; humeral area laterally weakly produced (Fig. 49D). Surface shiny; pronotum distinctly foveolate with additional irregular rugae on dorsum; anepisternum, katepisternum, and propodeum with fine rugulae and smooth interspaces. Pilosity moderately dense, long, and erect (Fig. 49D, F). ***Petiole.*** Shiny with fine foveolae; node smooth to finely foveolate, triangular, with rounded and thick apex, in rear view node dorsoventrally straight to slightly convex; pilosity moderately sparse and erect (Fig. 49D, F). ***Postpetiole.*** Shiny and foveolate; dorsum with reduced sculpture; in dorsal view oval, lateral margins medially with two dentate projections; pilosity long, moderately sparse, and erect (Fig. 49D, F). ***Gaster.*** Shiny and smooth; pilosity dense, moderately long, and erect (Fig. 49D, F). ***Colour.*** Dark orange; mandibles and gaster brown (Fig. 49D, F).

**Minor workers.** Measurements (*N* = 3): HL: 0.45–0.49 (0.47); HW: 0.4–0.43 (0.42); SL: 0.43–0.45 (0.44); EL: 0.09–0.1 (0.09); WL: 0.55–0.59 (0.57); PSL: 0.05–0.05 (0.05); MTL: 0.34–0.36 (0.35); PNW: 0.27–0.29 (0.28); PTW: 0.06–0.07 (0.06); PPW: 0.09–0.1 (0.1); CI: 111.5–116.3 (113.5); SI: 103.0–108.5 (106.3); PSLI: 9.3–11.2 (10.1); PPI: 64.6–71.0 (67.3); PNI: 65.8–68.1 (66.9); MTI: 82.9–85.8 (84.3).

***Head.*** Cephalic margin indistinctly concave to straight (Fig. 49A). Pilosity relatively dense, moderately long, decumbent to suberect. Sculpture shiny; medial frons smooth; lateral sides of frons and vertex with fine, sparse, and irregular rugulae with smooth interspaces; area posterolateral from eyes smooth; antennal sockets with few thick, curved outward rugae and indistinctly foveolate interspaces. Clypeus with median longitudinal carina absent; two lateral longitudinal carinae absent. Scape, when laid back, surpassing the posterior head margin by one-fifth of its length; pilosity dense, subdecumbent to erect (Fig. 49A, C). ***Mesosoma.*** In lateral view, promesonotum moderately high and short, arched; promesonotal groove absent; metanotal groove distinct; propodeal spines small and triangular (Fig. 49C). Sculpture shiny and predominantly smooth with very sparse, fine, and irregular rugulae. Pilosity moderately sparse, moderately long, and erect (Fig. 49C, E). ***Gaster.*** With sparse, erect pilosity (Fig. 49C, E). ***Colour.*** Dark yellow, gaster and vertex brownish (Fig. 49C, E).

##### Etymology.

Latin for small in reference to the body size.

##### Biology.

The species was collected between 750–1490 m in elevation, in montane forest and rainforest. Nests were located in rotten logs and litter.

##### Comments.

*Pheidole parvula* sp. nov. belongs to the group of species characterised by small body size (major workers: HL < 1.05 mm, WL < 0.9 mm and minor workers HL < 0.5 mm, WL < 0.6 mm), head sub-oval and slightly widening posteriorly with anterior and posterior sides convex in major workers, and minor workers with yellow to brown body colouration and head foveolate or predominantly smooth and relatively oval. The group includes six species: *P. havoana* sp. nov., *P. kely* sp. nov., *P. parvula* sp. nov., *P. parvulogibba* sp. nov., *P. volontany* sp. nov., and *P. midongy* sp. nov. Within this group, *P. parvula* sp. nov., described form two localities, Réserve Speciale d’Ambohitantely in Antananarivo and Ankerana in Toamasina, is most similar to parapatric *P. kely* sp. nov. *P. parvula* sp. nov. may also be confused with two taxa known only from the Anosyenne Mts. in Toliara, *P. parvulogibba* sp. nov. and *P. havoana* sp. nov. Minor workers of *P. parvula* sp. nov. distinctly differ from *P. kely* sp. nov. and *P. havoana* sp. nov. in never foveolate head; and from *P. parvulogibba* sp. nov. in lateral sides of frons and vertex with fine, sparse and irregular rugulae with smooth interspaces. Major workers of *P. parvula* sp. nov. differ from *P. kely* sp. nov. and *P. parvulogibba* sp. nov. by medial part of frons with thick and dense rugae and distinctly rugulate interspaces; and differ from *P. havoana* sp. nov. in promesonotum foveolate with sparse, thick to moderately thick, transverse to irregular rugae on dorsum and lack of smooth notches.

#### Pheidole parvulogibba sp. nov.

Taxon classificationAnimalia

6CCF6B98-8973-5292-9020-09D5A75BD9C4

http://zoobank.org/0F2B084A-3C82-4BC0-9B89-8C098D5F8E9E

**Figure 50.**
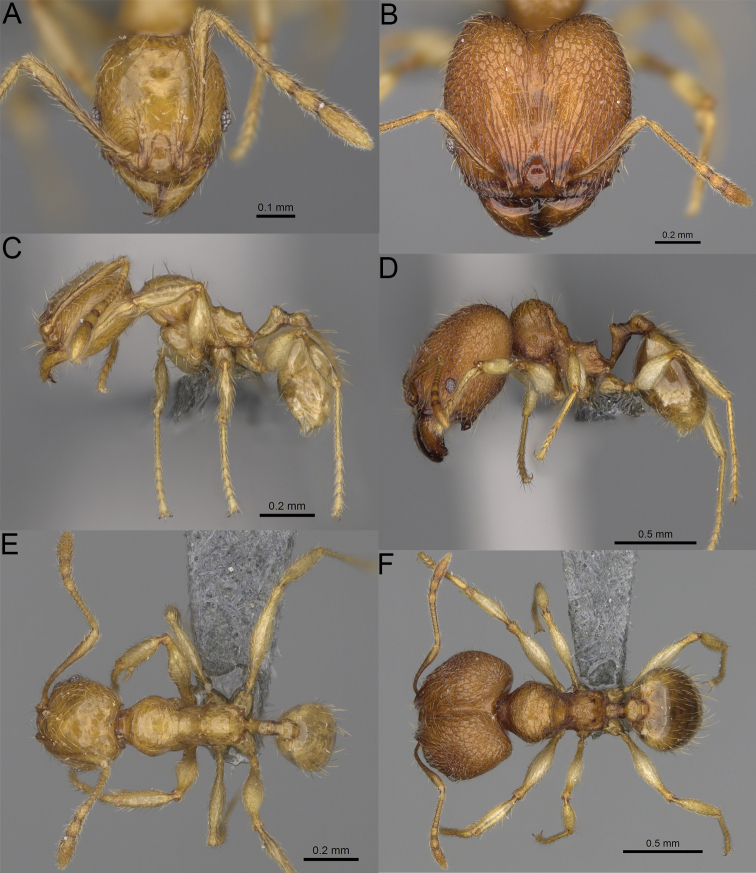
*Pheidole parvulogibba* sp. nov., full-face view (**A**), profile (**C**), and dorsal view (**E**) of paratype minor worker (CASENT0923274) and full-face view (**B**), profile (**D**), and dorsal view (**F**) of holotype major worker (CASENT0704453).

Figs 50A–F , 64H , 66L

##### Type material.

***Holotype.*** Madagascar. • 1 major worker; Toliara; Anosy Region, Anosyenne Mts, 29.9 km NW Manantenina; -24.13894, 47.06804; alt. 750 m; 22 Feb 2015; B. L. Fisher et al. leg.; montane rainforest, under rootmat, on rock; BLF36257; CASENT0704453 (CASC). ***Paratype.*** • 1w.; same data as for holotype, CASENT0923274 (CASC).

##### Diagnosis.

Minute species. ***Major workers.***HL < 1.1 mm and WL < 0.9 mm; head in full-face view sub-oval, slightly widening posteriorly, with anterior and posterior sides convex, in lateral view sub-oval; ventral and dorsal faces convex; body yellowish orange; sides of head with moderately dense, moderately long, decumbent to subdecumbent pilosity; entire head distinctly sculptured, medial part of frons with thick, longitudinal, and dense rugae and smooth to indistinctly rugulate interspaces, rugae more irregular and directed slightly outward on posteromedial part; scape, when laid back, reaching the midlength of head; promesonotum foveolate with sparse, thick to moderately thick, transverse to irregular rugae on dorsum; anepisternum, katepisternum, and propodeum with sparser foveolae; inner hypostomal teeth distinct, large, closely spaced, triangular, with rounded apex directed upward; outer hypostomal teeth lobe-like, wider than inner teeth and approximately the same height; inner and outer hypostomal teeth closely spaced and not connected by concavity; base of first gastral tergite smooth. ***Minor workers.***HL < 0.5 mm and WL < 0.6 mm, scape, when laid back, exceeding the posterior head margin by one-fifth of its length; propodeal spines reduced to small tubercles; head relatively oval and entirely smooth; body yellow; mesosoma smooth.

##### Description.

**Major workers.** Measurements (*N* = 1): HL: 1.06; HW: 1.05; SL: 0.56; EL: 0.12; WL: 0.9; PSL: 0.13; MTL: 0.5; PNW: 0.5; PTW: 0.12; PPW: 0.32; CI: 101.2; SI: 53.7; PSLI: 12.4; PPI: 36.7; PNI: 47.4; MTI: 48.1.

***Head.*** In full-face view sub-oval, slightly widening posteriorly, with anterior and posterior sides convex (Fig. 50B). In lateral view sub-oval; ventral and dorsal faces convex; inner hypostomal teeth visible. Sides of the head with moderately dense, moderately long, decumbent to subdecumbent pilosity; whole head with dense, long, decumbent to erect pilosity. Medial part of frons with thick, longitudinal, and dense rugae and smooth to indistinctly rugulate interspaces, rugae more irregular and directed slightly outward on posteromedial part; lateral sides with thick, sparse, and irregular rugae with distinctly rugulate interspaces. Occipital lobes with dense, irregular rugae and distinctly rugoreticulate interspaces. Area posterolateral from eyes with weaker sculpture, rugoreticulate. Gena with relatively dense, thick, and longitudinal rugae and smooth to rugoreticulate interspaces. Clypeus with smooth centre and shiny, lateral sides with indistinct rugulae; median notch present, moderately wide, and shallow; median longitudinal carina absent; lateral longitudinal carinae absent. Scape, when laid back, reaching the midlength of head; pilosity subdecumbent to erect (Fig. 50B, D). Inner hypostomal teeth distinct, large, closely spaced, triangular, with rounded apex directed upward; outer hypostomal teeth lobe-like, wider than inner teeth and approximately the same height; inner and outer hypostomal teeth closely spaced and not connected by concavity (Fig. 64H). ***Mesosoma.*** In lateral view, promesonotum short, angular, and moderately high, posterior mesonotum moderately steep, mesonotal process very indistinct, bulge-like; promesonotal groove absent; metanotal groove absent; propodeal spines very short, with wide base and acute apex; humeral area produced (Fig. 50D). Surface shiny; promesonotum foveolate with sparse, thick to moderately thick, transverse to irregular rugae on dorsum; anepisternum, katepisternum, and propodeum with sparser foveolae. Pilosity moderately sparse, moderately long, and erect (Fig. 50D, F). ***Petiole.*** Shiny with dense foveolae; node finely foveolate, triangular, with rounded and thin apex, in rear view node dorsoventrally convex; pilosity moderately sparse and erect (Fig. 50D, F). ***Postpetiole.*** Shiny and foveolate; dorsum with reduced sculpture and smooth notch; in dorsal view oval, lateral margins medially with two dentate projections; pilosity long, moderately sparse, and erect (Fig. 50D, F). ***Gaster.*** Shiny and smooth; pilosity moderately dense, moderately short, and erect (Fig. 50D, F). ***Colour.*** Yellowish orange with yellow legs (Fig. 50D, F).

**Minor workers.** Measurements (*N* = 1): HL: 0.49; HW: 0.44; SL: 0.47; EL: 0.09; WL: 0.58; PSL: 0.06; MTL: 0.36; PNW: 0.29; PTW: 0.06; PPW: 0.11; CI: 113.0; SI: 107.8; PSLI: 12.8; PPI: 60.0; PNI: 67.0; MTI: 82.6.

***Head.*** Cephalic margin indistinct and relatively straight (Fig. 50A). Pilosity relatively sparse, moderately long, decumbent to subdecumbent. Sculpture shiny and smooth; antennal sockets with few thick, curved outward rugae and smooth interspaces. Clypeus with median longitudinal carina absent; two lateral longitudinal carinae absent. Scape, when laid back, exceeding the posterior head margin by one-fifth of its length; pilosity dense, subdecumbent to erect (Fig. 50A, C). ***Mesosoma.*** In lateral view, promesonotum moderately high and short, arched; promesonotal groove absent; metanotal groove distinct; propodeal spines reduced to small tubercles (Fig. 50C). Sculpture shiny and smooth. Pilosity moderately sparse, moderately long, and erect (Fig. 50C, E). ***Gaster.*** With sparse, erect pilosity (Fig. 50C, E). ***Colour.*** Yellow (Fig. 50C, E).

##### Etymology.

Latin for both small and gibbus, in reference to small body size and shape of promesonotum of major workers.

##### Biology.

The species was collected at 750 m in elevation, in montane rainforest. Nest was located under rootmats, on rock.

##### Comments.

*Pheidole parvulogibba* sp. nov. belongs to the group of species characterised by small body size (major workers: HL < 1.05 mm, WL < 0.9 mm and minor workers HL < 0.5 mm, WL < 0.6 mm), head sub-oval, slightly widening posteriorly with anterior and posterior sides convex in major workers, and minor workers with yellow to brown body colouration and head foveolate or predominantly smooth and relatively oval. The group includes six species: *P. havoana* sp. nov., *P. kely* sp. nov., *P. parvula* sp. nov., *P. parvulogibba* sp. nov., *P. volontany* sp. nov., and P. *midongy* sp. nov. Within this group, *P. parvulogibba* sp. nov., described from the Anosyenne Mts. in Toliara, is most similar to *P. kely* sp. nov., known from northern Madagascar, *P. parvula* sp. nov., known from the vicinity of Antananarivo, and sympatric *P. havoana* sp. nov. Minor workers of *P. parvulogibba* sp. nov. distinctly differ from *P. kely* sp. nov. and *P. havoana* sp. nov. in never foveolate head; and from *P. parvula* sp. nov. in entirely smooth frons and vertex. Major workers of *P. parvulogibba* sp. nov. differ from *P. havoana* sp. nov. and *P. parvula* sp. nov. in medial part of frons with thick, dense rugae and smooth to indistinctly rugulate interspaces; from *P. kely* sp. nov. in promesonotum foveolate with sparse, thick to moderately thick, transverse to irregular rugae on dorsum; anepisternum, katepisternum, and propodeum with sparser foveolae but never smooth notches.

#### Pheidole renirano sp. nov.

Taxon classificationAnimalia

EDBFE9BB-5B35-5AF2-9322-0A90CD1E0ADB

http://zoobank.org/E2EAF02B-CBFA-4AA5-BD9B-DD1115D47A01

**Figure 51.**
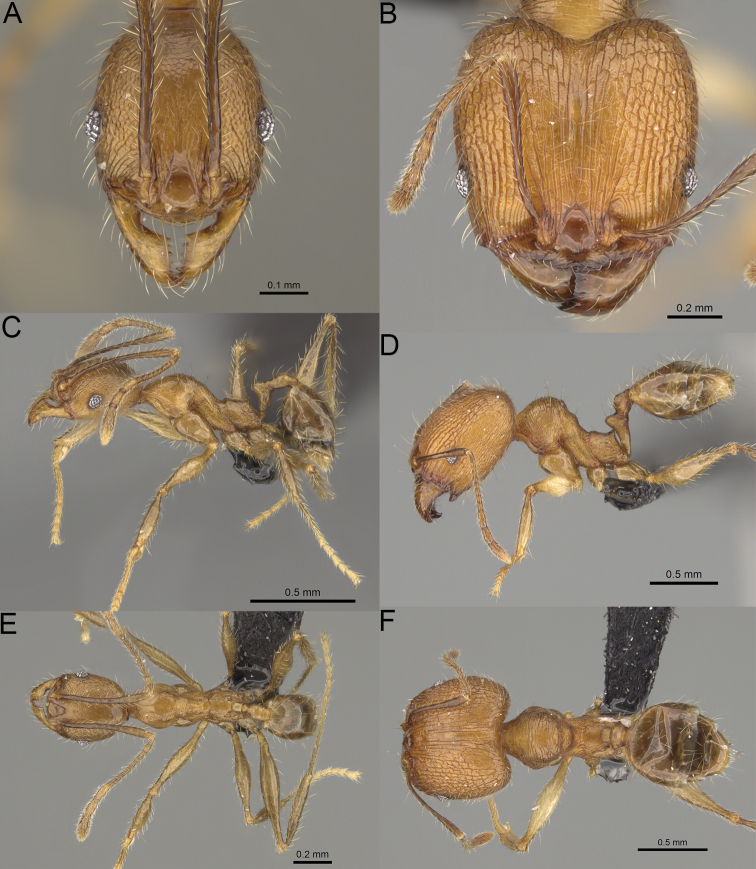
*Pheidole renirano* sp. nov., full-face view (**A**), profile (**C**), and dorsal view (**E**) of paratype minor worker (CASENT0923324) and full-face view (**B**), profile (**D**), and dorsal view (**F**) of holotype major worker (CASENT0161924).

Figs 51A–F , 64I , 67A

##### Type material.

***Holotype.*** Madagascar. • 1 major worker; Toamasina; Réserve Spéciale Ambatovaky, Sandrangato river; -16.7674, 49.26813; alt. 500 m; 23 Feb 2010; B. L. Fisher et al. leg.; rainforest, ex rotten log; BLF24675; CASENT0161924 (CASC). ***Paratype.*** • 1w.; same data as for holotype, CASENT0923324 (CASC).

##### Other material.

Madagascar. –**Toamasina**: • 1w.; Ankerana; -18.4061, 48.82029; alt. 725 m; 16 Jan 2012; B. L. Fisher et al. leg.; rainforest, ex rotten log; BLF27684 (CASC). •1w., 1s.; Parc National Mananara-Nord, 7.1 km 261°Antanambe; -16.455, 49.7875; alt. 225 m; 15 Nov 2005; B. L. Fisher et al. leg.; rainforest, ex rotten stick on ground; BLF12611 (CASC). • 1w., 1s.; Res. Ambodiriana, 4.8 km 306°Manompana, along Manompana river; -16.67233, 49.70117; alt. 125 m; 19 Nov 2005; B. L. Fisher et al. leg.; rainforest, ex rotten log; BLF12809 (CASC). • 1w., 1s.; Réserve Spéciale Ambatovaky, Sandrangato river; -16.81745, 49.2925; alt. 400 m; 26 Feb 2010; B. L. Fisher et al. leg.; rainforest, ex rotten log; BLF24866 (CASC).

##### Diagnosis.

Moderately large species. ***Major workers.*** Head in full-face view sub-oval, not widening posteriorly, with anterior and posterior sides slightly convex, in lateral view sub-oval; ventral and dorsal faces convex; sides of the head with moderately dense, moderately long, suberect pilosity; medial part of frons with thick, longitudinal, interrupted and dense rugae, interspaces smooth to indistinctly rugulate; lateral sides with thicker, dense and longitudinal to posteriorly slightly irregular rugae with predominantly smooth but sometimes indistinctly rugulate interspaces; occipital lobes and area posterolateral from eyes without smooth notches; scape, when laid back, exceeding the midlength of head by two-fifths of its length; inner hypostomal teeth distinct, large, closely spaced, triangular, with rounded apex directed upward; outer hypostomal teeth lobe-like, wider than and approximately as high as inner teeth; inner and outer hypostomal teeth closely spaced and not connected by concavity; mesosoma with sparse foveolae; promesonotum with additional sparse, thin, and transverse rugulae and smooth to indistinctly foveolate interspaces on dorsum; gaster smooth with base indistinctly shagreened; body yellow. ***Minor workers.*** Head foveolate, foveolae sparse; scape, when laid back, exceeding the posterior head margin by one-third of its length; promesonotum low and moderately long; promesonotal groove absent; propodeal spines minute, triangular; mesosoma foveolate; katepisternum with large, smooth notch; dorsal side of promesonotum and lateral sides of pronotum with reduced sculpture and smooth notches on medial parts; body yellowish brown.

##### Description.

**Major workers.** Measurements (*N* = 4): HL: 1.05–1.21 (1.16); HW: 1.03–1.16 (1.12); SL: 0.76–0.88 (0.84); EL: 0.12–0.17 (0.15); WL: 1.03–1.14 (1.1); PSL: 0.14–0.18 (0.16); MTL: 0.69–0.82 (0.77); PNW: 0.45–0.54 (0.5); PTW: 0.13–0.15 (0.14); PPW: 0.26–0.31 (0.29); CI: 101.5–105.3 (103.6); SI: 73.2–76.3 (75.1); PSLI: 11.9–15.0 (13.4); PPI: 46.2–49.6 (47.8); PNI: 39.5–51.8 (44.4); MTI: 66.5–70.5 (68.8).

***Head.*** In full-face sub-oval, not widening posteriorly, with anterior and posterior sides slightly convex (Fig. 51B). In lateral view sub-oval; ventral and dorsal faces convex; inner hypostomal teeth visible. Sides of the head with moderately dense, moderately long, suberect pilosity; whole head with dense, long, decumbent to erect pilosity. Medial part of frons with thick, longitudinal, interrupted and dense rugae, interspaces smooth to indistinctly rugulate; lateral sides with thicker, dense, and longitudinal to posteriorly slightly irregular rugae with predominantly smooth but sometimes indistinctly rugulate interspaces. Occipital lobes with thick and dense irregular rugae and predominantly smooth interspaces. Area posterolateral from eyes with longitudinal, moderately thick, very dense rugae with smooth to rugulate interspaces. Gena with relatively sparse, thick, and longitudinal rugae and smooth to indistinctly rugulate interspaces. Centre of clypeus smooth and shiny, lateral sides with indistinct rugulae; median notch present, moderately wide, and shallow; median longitudinal carina present; lateral longitudinal carinae absent. Scape, when laid back, exceeding the midlength of head by two-fifths of its length; pilosity subdecumbent to erect (Fig. 51B, D). Inner hypostomal teeth distinct, large, closely spaced, triangular, with rounded apex directed upward; outer hypostomal teeth lobe-like, wider than and approximately as high as inner teeth; inner and outer hypostomal teeth closely spaced and not connected by concavity (Fig. 64I). ***Mesosoma.*** In lateral view, promesonotum short, angular and moderately low, posterior mesonotum moderately steep, mesonotal process indistinct, tubercle-like; promesonotal groove absent; metanotal groove absent; propodeal spines moderate, with wide base and acute apex; humeral area produced (Fig. 51D). Surface shiny and sparse foveolae; promesonotum with additional sparse, thin, and transverse rugulae and smooth to indistinctly foveolate interspaces on dorsum. Pilosity moderately sparse, long, and erect (Fig. 51D, F). ***Petiole.*** Shiny with dense foveolae; node finely foveolate, triangular, with rounded and thick apex, in rear view node dorsoventrally slightly convex; pilosity moderately sparse and erect (Fig. 51D, F). ***Postpetiole.*** Shiny and foveolate; dorsum with reduced sculpture and smooth notch; in dorsal view oval, lateral margins medially with two dentate projections; pilosity long, moderately sparse, and erect (Fig. 51D, F). ***Gaster.*** Shiny and smooth with base indistinctly shagreened; pilosity moderately sparse, long, and erect (Fig. 51D, F). ***Colour.*** Yellow with yellowish legs (Fig. 51D, F).

**Minor workers.** Measurements (*N* = 4): HL: 0.53–0.59 (0.55); HW: 0.42–0.46 (0.44); SL: 0.66–0.74 (0.72); EL: 0.11–0.11 (0.11); WL: 0.69–0.78 (0.74); PSL: 0.06–0.08 (0.07); MTL: 0.51–0.58 (0.55); PNW: 0.3–0.33 (0.31); PTW: 0.06–0.08 (0.07); PPW: 0.11–0.14 (0.12); CI: 119.8–130.5 (126.5); SI: 157.8–170.9 (163.5); PSLI: 11.4–15.6 (13.3); PPI: 55.1–63.9 (59.3); PNI: 68.5–73.9 (70.8); MTI: 121.1–134.2 (126.2).

***Head.*** Cephalic margin slightly convex (Fig. 51A). Pilosity relatively sparse, moderately long, subdecumbent to erect. Sculpture shiny and foveolate, foveolae sparse; antennal sockets with few thick, curved outward rugae and foveolate interspaces. Clypeus with median longitudinal carina absent; two lateral longitudinal carinae absent. Scape, when laid back, exceeding the posterior head margin by one-third of its length; pilosity dense, suberect to erect (Fig. 51A, C). ***Mesosoma.*** In lateral view, promesonotum low and moderately long, arched; promesonotal groove absent; metanotal groove distinct; propodeal spines minute, triangular (Fig. 51C). Sculpture shiny and foveolate; katepisternum with big smooth notch; dorsal side of promesonotum and lateral sides of pronotum with reduced sculpture and smooth notches on medial parts. Pilosity very sparse, moderately long, and erect (Fig. 51C, E). ***Gaster.*** With sparse, erect pilosity (Fig. 51C, E). ***Colour.*** Yellowish brown, vertex and gaster darker (Fig. 51C, E).

##### Etymology.

Malagasy for river in reference to the Sandrangato River located in the type locality.

##### Biology.

The species was collected between 125–725 m in elevation, in rainforest. Nests were located in rotten logs and sticks on the ground.

##### Comments.

*Pheidole renirano* sp. nov. is known from several localities in the Toamasina prefecture. Its range covers the area from Réserve Spéciale Ambatovaky north to Antanambe. Morphologically it is most similar to parapatric *P. tsaravoniana* sp. nov., known from the vicinity of Tsaravoniana in Toamasina. Major workers of *P. renirano* sp. nov. can be distinguished from majors of *P. tsaravoniana* sp. nov. based on bright yellow body colouration and promesonotal dorsum with short, transverse rugae with smooth to indistinctly foveolae interspaces; minors can be separated based on yellowish brown body, and reduced sculpture and smooth notches on medial parts of dorsal side of promesonotum and lateral sides of pronotum.

#### Pheidole sava sp. nov.

Taxon classificationAnimalia

B1BA5EB3-7B3D-5113-BF9C-991F6618A2D2

http://zoobank.org/B7516043-C09C-4573-9A1B-9B649C329C67

**Figure 52.**
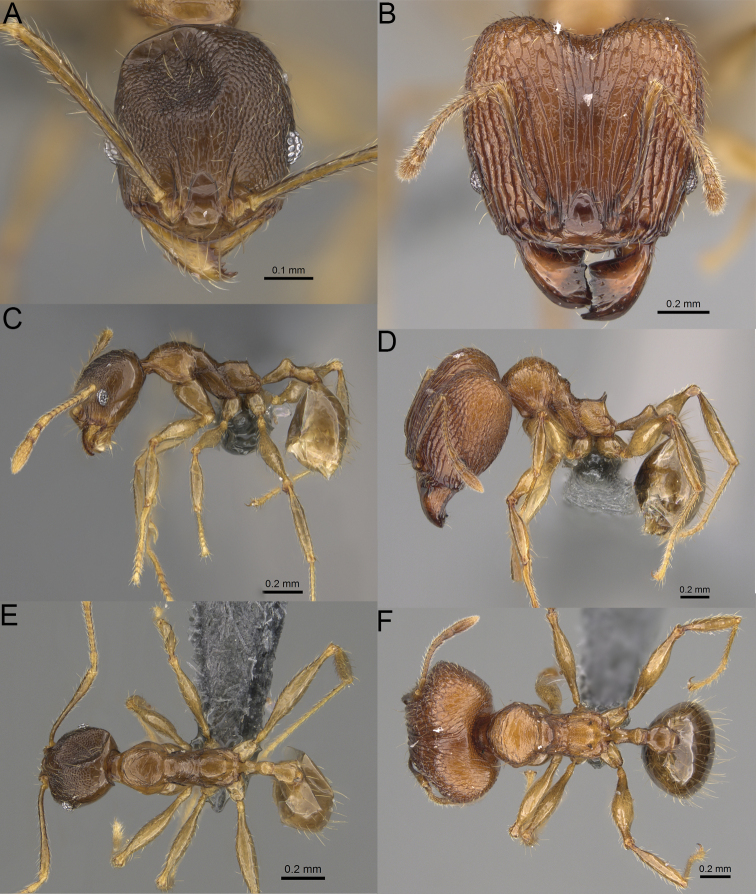
*Pheidole sava* sp. nov., full-face view (**A**), profile (**C**), and dorsal view (**E**) of paratype minor worker (CASENT0923296) and full-face view (**B**), profile (**D**), and dorsal view (**F**) of holotype major worker (CASENT0808425).

Figs 52A–F , 64J , 67B

##### Type material.

***Holotype.*** Madagascar. • 1 major worker; Antsiranana; Sava Region: Parc National de Marojejy, Manantenina River, 21.3 km 27.0°NE Andapa; -14.43686, 49.74291; alt. 1350 m; 12 Feb 2018; B. L. Fisher et al. leg.; montane rainforest, ex rotten log; BLF41084; CASENT0808425 (CASC). ***Paratype.*** • 1w.; same data as for holotype; CASENT0923296 (CASC).

##### Other material.

Madagascar. –**Antsiranana**: • 1w., 1s.; Sava Region: Parc National de Marojejy, Manantenina River, 21.3 km 27.0°NE Andapa; -14.43686, 49.74291; alt. 1350 m; 12 Feb 2018; B. L. Fisher et al. leg.; montane rainforest, ex root mat; BLF41089 (CASC). • 1w., 1s.; Sava Region: Parc National de Marojejy, Manantenina River, 21.3 km 27.0°NE Andapa; -14.43686, 49.74291; alt. 1350 m; 12 Feb 2018; B. L. Fisher et al. leg.; montane rainforest, ex rotten log; BLF41097 (CASC).

##### Diagnosis.

Moderately large species. ***Major workers.*** Head in full-face view sub-oval, widening posteriorly, with anterior and posterior sides convex, in lateral view sub-oval; ventral and dorsal faces convex; sides of the head with dense, short, suberect pilosity; anterior and medial parts of frons with moderately dense, thick, and longitudinal rugae sometimes interrupted in the posteromedial part, interspaces with sparse and distinct rugulae; posterolateral sides with longitudinal to irregular and thick rugae with distinctly rugulate interspaces; area posterolateral from eyes without smooth notches; scape, when laid back, exceeding the midlength of head by approximately one-fifth of its length; inner hypostomal teeth distinct, moderate, closely spaced, triangular, with rounded apex and wide base; outer hypostomal teeth lobe-like, wider than inner hypostomal teeth and approximately the same height, apex directed outward; inner and outer hypostomal teeth closely spaced and connected by indistinct concavity; mesosoma with fine rugofoveolae, dorsal and lateral sides of pronotum with additional transverse and thin rugae and smooth notches; gaster smooth; body brownish orange. ***Minor workers.*** Head foveolate with additional indistinct longitudinal rugulae on medial frons, area posterolateral from eyes smooth; scape, when laid back, surpassing the posterior head margin by one-fifth of its length; promesonotum moderately high and long, arched; promesonotal groove present; propodeal spines indistinct, triangular; mesosoma smooth and shiny, only promesonotal dorsum with arched rugae and indistinct foveolae, mesonotum and lateral sides of propodeum with indistinct and sparse foveolae; body yellowish brown.

##### Description.

**Major workers.** Measurements (*N* = 3): HL: 1.14–1.24 (1.2); HW: 1.16–1.23 (1.2); SL: 0.66–0.69 (0.68); EL: 0.13–0.15 (0.14); WL: 1.07–1.1 (1.09); PSL: 0.19–0.21 (0.2); MTL: 0.68–0.73 (0.7); PNW: 0.52–0.53 (0.52); PTW: 0.14–0.17 (0.16); PPW: 0.28–0.34 (0.31); CI: 98.2–101.0 (99.7); SI: 55.5–57.1 (56.4); PSLI: 16.2–16.7 (16.5); PPI: 44.8–61.3 (51.3); PNI: 42.7–45.3 (43.7); MTI: 57.5–59.1 (58.4).

***Head.*** In full-face view sub-oval, widening posteriorly, with anterior and posterior sides convex (Fig. 52B). In lateral view sub-oval; ventral and dorsal faces convex; inner hypostomal teeth invisible. Sides of the head with dense, short, suberect pilosity; whole head with relatively dense, short, decumbent to erect pilosity. Anterior and medial parts of frons with moderately dense and thick, longitudinal, and sometimes interrupted rugae in posteromedial part, interspaces with sparse and distinct rugulae; posterolateral sides with longitudinal to irregular and thick rugae with distinctly rugulate interspaces. Occipital lobes with thick, sparse, irregular rugae, interspaces smooth or with fine, indistinct rugulae. Gena with sparse, thick, and longitudinal rugae and smooth interspaces. Area posterolateral from eyes with dense longitudinal to irregular rugofoveolae. Centre of clypeus smooth and shiny, lateral sides with indistinct rugulae; median notch present, wide, and shallow; median longitudinal carina absent; lateral longitudinal carinae absent. Scape, when laid back, exceeding the midlength of head by approximately one-fifth of its length; pilosity decumbent to suberect (Fig. 52B, D). Inner hypostomal teeth distinct, moderate, closely spaced, triangular, with rounded apex and wide base; outer hypostomal teeth lobe-like, wider than inner hypostomal teeth and approximately the same height, apex directed outward; inner and outer hypostomal teeth closely spaced and connected by indistinct concavity (Fig. 64J). ***Mesosoma.*** In lateral view, promesonotum short, angular, and moderately high, posterior mesonotum moderately steep, mesonotal process indistinct, tubercle-like; promesonotal groove absent; metanotal groove absent; propodeal spines moderate, thin, with acute apex; humeral area laterally weakly produced (Fig. 52D). Surface shiny with fine rugofoveolae, dorsal and lateral sides of pronotum with additional transverse and thin rugae and smooth notches. Pilosity sparse, moderately long, and erect (Fig. 52D, F). ***Petiole.*** Shiny with fine and sparse rugofoveolae; peduncle; node smooth to indistinctly rugulose, low, triangular, with rounded apex, in rear view node dorsoventrally convex; pilosity moderately sparse and erect (Fig. 52D, F). ***Postpetiole.*** Shiny, with fine and sparse rugulae; in dorsal view postpetiole oval, lateral margins medially with two indistinct dentate projections; pilosity long, moderately sparse, and erect (Fig. 52D, F). ***Gaster.*** Shiny and smooth; pilosity moderately dense, long, and erect (Fig. 52D, F). ***Colour.*** Brownish orange; legs dark yellow, gaster bright brown (Fig. 52D, F).

**Minor workers.** Measurements (*N* = 3): HL: 0.58–0.61 (0.59); HW: 0.48–0.49 (0.49); SL: 0.6–0.63 (0.61); EL: 0.11–0.12 (0.11); WL: 0.73–0.74 (0.73); PSL: 0.07–0.09 (0.08); MTL: 0.5–0.52 (0.51); PNW: 0.3–0.34 (0.32); PTW: 0.09–0.1 (0.09); PPW: 0.13–0.16 (0.15); CI: 117.1–127.1 (121.6); SI: 122.0–129.4 (126.2); PSLI: 11.3–14.2 (12.8); PPI: 60.4–65.7 (63.0); PNI: 62.7–70.4 (66.4); MTI: 103.3–106.8 (104.7).

***Head.*** Cephalic margin indistinctly convex (Fig. 52A). Pilosity relatively sparse, long, decumbent to subdecumbent. Head foveolate with additional indistinct longitudinal rugulae on medial frons, area posterolateral from eyes smooth; antennal sockets with few thick, curved outward rugae and foveolae interspaces. Clypeus with median longitudinal carina absent; two lateral longitudinal carinae absent. Scape, when laid back, surpassing the posterior head margin by one-fifth of its length; pilosity dense, subdecumbent to erect (Fig. 52A, C). ***Mesosoma.*** In lateral view, promesonotum moderately high and long, arched; promesonotal groove present but indistinct; metanotal groove present and distinct; propodeal spines indistinct, lobe-like (Fig. 52C). Sculpture smooth and shiny, only promesonotal dorsum with arched rugae and indistinct foveolae, mesonotum and lateral sides of propodeum with indistinct and sparse foveolae. Pilosity sparse, moderately long, and erect (Fig. 52C, E). ***Petiole.*** Peduncle short and thin with ventral face relatively straight (Fig. 52C, E). ***Gaster.*** With sparse, erect pilosity (Fig. 52C, E). ***Colour.*** Brown; legs and lateral sides of pronotum yellowish brown (Fig. 52C, E).

##### Etymology.

From the type locality.

##### Biology.

The species was collected at 1350 m in elevation, in montane rainforest. Nests were located in rotten logs and root mat.

##### Comments.

*Pheidole sava* sp. nov., known from Parc National de Marojejy in Antsiranana, is most similar to *P. itremo* sp. nov., known from Forêt d’Atsirakambiaty in Fianarantsoa and Forêt d Ambohitantely in Antananarivo. Major workers of *P. sava* sp. nov. can be separated based on brighter body colouration, presence of dense, short, suberect pilosity sides of the head, medial frons with sparse and distinctly rugulate interspaces, finely rugofoveolae mesosoma with dorsal and lateral sides of pronotum with additional transverse and thin rugae and smooth notches; minor workers can be separated based on foveolate head with additional indistinct longitudinal rugulae on medial frons, smooth area posterolateral from eyes, predominantly smooth mesosoma with promesonotal dorsum with arched rugae and indistinct foveolae, and indistinct and sparse foveolae on mesonotum and lateral sides of propodeum.

#### Pheidole sikorae

Taxon classificationAnimalia

Forel, 1891

554932BE-F634-5443-A6E9-4C2C115F813E

**Figure 53.**
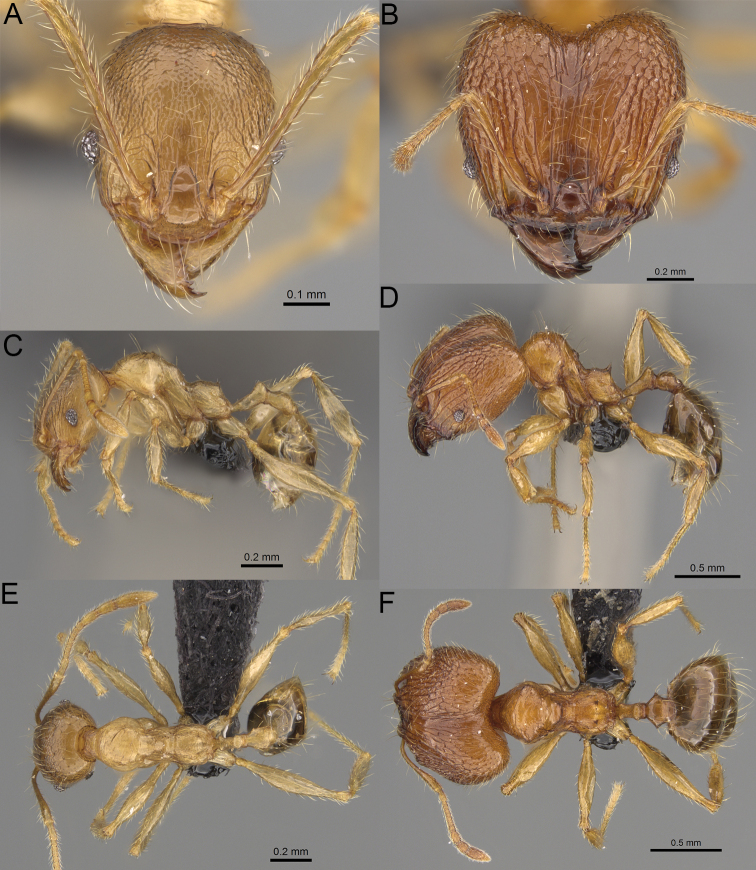
*Pheidole sikorae* Forel, full-face view (**A**), profile (**C**), and dorsal view (**E**) of minor worker (CASENT0923322) and full-face view (**B**), profile (**D**), and dorsal view (**F**) of major worker (CASENT0149951).

Figs 53A–F , 64K , 67C

##### Type material.

*Pheidole sikorae* Forel, 1891: 223 (s.w.). Lectotype [designated here]: major worker (top specimen, CASENT0101692): Madagascar, Antananarivo, Andrangoloaka, coll. Sikora (MHNG) [examined]. Paralectotypes: 1 major worker (CASENT0876548, bottom specimen, the same pin as lectotype) (MHNG) [examined], 3 minor workers (CASENT0101668) (MHNG) [examined].

##### Other material.

Madagascar. –**Fianarantsoa**: • 1w., 1s.; 2 km W Andrambovato, along river Tatamaly; -21.51167, 47.41; alt. 1075 m; 3 Jun 2005; B. L. Fisher et al. leg.; montane rainforest, ex rotten pocket above ground; BLF12209 (CASC). • 6w., 3s.; Parc National de Ranomafana, Vatoharanana River, 4.1 km 231°SW Ranomafana; -21.29, 47.43333; alt. 1100 m; 27 Mar 2003; B. L. Fisher et al. leg.; montane rainforest, ex rotten log; BLF08414 (CASC).–**Toamasina**: • 1w., 1s.; Ile Sainte Marie, Forêt Ambohidena, 22.8 km 44° Ambodifotatra; -16.82433, 49.96417; alt. 20 m; 22 Nov 2005; B. L. Fisher et al. leg.; littoral rainforest, ex dead twig above ground; BLF12907 (CASC).–**Toliara**: • 1w., 1s.; Réserve Spéciale Kalambatritra, Ambinanitelo; -23.4502, 46.45658; alt. 1325 m; 11 Feb 2009; B. L. Fisher et al. leg.; montane rainforest, ex rotten log; BLF21764 (CASC). • 2w., 1s.; ibid.; BLF21779 (CASC). • 1w., 1m.; ibid.; BLF21794 (CASC). • 1w., 1s.; ibid.; BLF21805 (CASC). • 1w.; ibid.; BLF21826 (CASC). •1 w., 1m.; ibid.; BLF21829 (CASC). • 1w., 1s.; ibid.; BLF21843 (CASC). • 1w., 1s.; ibid.; BLF21849 (CASC). • 1w.; Réserve Spéciale Kalambatritra, Ampanihy; -23.4635, 46.4631; alt. 1270 m; 9 Feb 2009; B. L. Fisher et al. leg.; montane rainforest; BLF21567 (CASC). • 1w., 1s.; ibid.; BLF21609 (CASC). • 1w., 1s.; ibid.; under moss on rotten log; BLF21647 (CASC). • 1w., 1s.; Réserve Spéciale Kalambatritra, Ampanihy; -23.463, 46.47057; alt. 1269 m; 10 Feb 2009; B. L. Fisher et al. leg.; montane rainforest, ex rotten log; BLF21655 (CASC). • 1w., 1s.; ibid.; BLF21656 (CASC). • 2w., 1s., 1q.; ibid.; BLF21712 (CASC). • 1w., 1s.; ibid.; BLF21716 (CASC). • 1w., 1s.; Réserve Spéciale Kalambatritra, Ampanihy; -23.4635, 46.4631; alt. 1270 m; 10 Feb 2009; B. L. Fisher et al. leg.; montane rainforest, ex rotten log; BLF21745 (CASC). • 1w.; Réserve Spéciale Kalambatritra, Befarara; -23.4178, 46.4478; alt. 1390 m; 7 Feb 2009; B. L. Fisher et al. leg.; montane rainforest; BLF21329 (CASC). • 2w., 1s., 1m.; ibid.; ex rotten log; BLF21358 (CASC). • 1w., 1s.; ibid.; BLF21360 (CASC). • 1m.; ibid.; BLF21361 (CASC). • 1w., 1s.; ibid.; BLF21362 (CASC). • 2w., 1s., 1q.; ibid.; BLF21369 (CASC). • 1w., 1s.; ibid.; BLF21392 (CASC). • 1w., 1s.; ibid.; BLF21400 (CASC). • 1w., 1s.; Réserve Spéciale Kalambatritra, Betanana; -23.4144, 46.459; alt. 1360 m; 8 Feb 2009; B. L. Fisher et al. leg.; montane rainforest, ex rotten log; BLF21426 (CASC). • 1w., 1s.; ibid.; BLF21437 (CASC). • 1w., 1s.; ibid; under moss on rotten log; BLF21450 (CASC). • 1m.; ibid; ex rotten log; BLF21482 (CASC).

##### Diagnosis.

Moderately large species. ***Major workers.*** Head in full-face view sub-oval, widening posteriorly, with anterior and posterior sides convex, in lateral view sub-oval; ventral and dorsal faces convex; impressed and smooth concavity placed lateral to antennal socket and tentorial pit present; sides of the head with dense, long, suberect to erect pilosity; anterior and medial parts of frons with sparse, thick, longitudinal, and sometimes interrupted rugae and smooth to indistinctly rugulae interspaces; posterolateral sides with more irregular rugae and smooth to indistinctly rugulate interspaces, area posterolateral from eyes without smooth notches; scape, when laid back, exceeding the midlength of head by approximately one-third of its length; inner hypostomal teeth distinct, moderately high, closely spaced, triangular, with rounded apex and wide base; outer hypostomal teeth lobe-like, wider and higher than inner hypostomal teeth, apex directed outward; inner and outer hypostomal teeth closely spaced and connected by concavity; promesonotum predominantly smooth, with sparse, thin to thick, transverse rugae on pronotum and sometimes with fine, irregular rugulae on anepisternum and katepisternum; propodeum with fine, sparse, and indistinct rugulae; gaster smooth; body yellow to orange. ***Minor workers.*** Head sculpture variable, from smooth with foveolae restricted to anterior or medial frons to foveolate with smooth patches on vertex and area posterolateral from eyes; foveolae always sparse, frons sometimes with additional, sparse, and irregular rugae; scape, when laid back, surpassing the posterior head margin by one-fifth of its length; promesonotum moderately high and short; promesonotal groove present; propodeal spines very small and triangular; mesosoma smooth and shiny, only dorsum with few transverse, thick rugulae and propodeum with indistinct and sparse foveolae; body yellow to orange.

##### Description.

**Major workers.** Measurements (*N* = 10): HL: 0.94–1.18 (1.11); HW: 0.93–1.19 (1.14); SL: 0.65–0.7 (0.67); EL: 0.12–0.15 (0.14); WL: 0.9–1.07 (1.01); PSL: 0.13–0.19 (0.16); MTL: 0.59–0.69 (0.66); PNW: 0.39–0.5 (0.47); PTW: 0.13–0.15 (0.14); PPW: 0.23–0.3 (0.27); CI: 94.2–101.4 (97.3); SI: 55.6–69.7 (59.3); PSLI: 12.1–15.7 (14.5); PPI: 42.5–55.4 (50.1); PNI: 40.0–42.2 (41.5); MTI: 54.4–63.7 (57.7).

***Head.*** In full-face view sub-oval, widening posteriorly, with anterior and posterior sides convex (Fig. 53B). In lateral view sub-oval; ventral and dorsal faces convex; inner hypostomal teeth invisible. Sides of the head with dense, long, suberect to erect pilosity; whole head with relatively dense, long, decumbent to erect pilosity. Anterior and medial parts of frons with sparse and thick, longitudinal, and sometimes interrupted rugae and smooth to indistinctly rugulate interspaces; posterolateral sides with more irregular rugae and smooth to indistinctly rugulate interspaces. Impressed and smooth concavity placed lateral to antennal socket and tentorial pit present. Occipital lobes with thick, sparse, irregular rugae, sometimes fading medially, interspaces smooth or with fine, indistinct rugulae. Gena with sparse, thick, and longitudinal rugae and smooth interspaces. Area posterolateral from eyes with weaker and sparser rugoreticulae. Centre of clypeus smooth and shiny, lateral sides with indistinct rugulae; median notch present, wide, and shallow; median longitudinal carina absent; lateral longitudinal carinae absent. Scape, when laid back, exceeding the midlength of head by approximately one-third of its length; pilosity decumbent to suberect (Fig. 53B, D). Inner hypostomal teeth distinct, moderately high, closely spaced, triangular, with rounded apex and wide base; outer hypostomal teeth lobe-like, wider and higher than inner hypostomal teeth, apex directed outward; inner and outer hypostomal teeth closely spaced and connected by concavity (Fig. 64K). ***Mesosoma.*** In lateral view, promesonotum short, angular, and moderately high, posterior mesonotum moderately steep, mesonotal process indistinct, tubercle-like; promesonotal groove absent; metanotal groove present; propodeal spines moderate, triangular, thin, with acute apex; humeral area laterally weakly produced (Fig. 53D). Surface shiny, promesonotum predominantly smooth, with sparse, thin to thick, transverse rugae on pronotum and sometimes with fine, irregular rugulae on anepisternum and katepisternum; propodeum with fine, sparse, and indistinct rugulae. Pilosity sparse, long, and erect (Fig. 53D, F). ***Petiole.*** Shiny with fine and sparse rugulae; peduncle; node smooth to indistinctly rugulose, low, triangular, with rounded apex, in rear view node dorsoventrally convex; pilosity moderately sparse and erect (Fig. 53D, F). ***Postpetiole.*** Shiny, with fine and sparse rugulae; in dorsal view postpetiole oval, lateral margins medially with two dentate projections; pilosity long, moderately sparse, and erect (Fig. 53D, F). ***Gaster.*** Shiny and smooth; pilosity sparse, long, and erect (Fig. 53D, F). ***Colour.*** Yellow to orange; gaster usually slightly darker (Fig. 53D, F).

**Minor workers.** Measurements (*N* = 10): HL: 0.55–0.61 (0.58); HW: 0.46–0.51 (0.49); SL: 0.58–0.63 (0.61); EL: 0.1–0.12 (0.11); WL: 0.67–0.76 (0.72); PSL: 0.06–0.08 (0.07); MTL: 0.45–0.53 (0.48); PNW: 0.3–0.35 (0.33); PTW: 0.07–0.09 (0.08); PPW: 0.13–0.16 (0.14); CI: 114.4–120.7 (118.4); SI: 121.9–127.5 (124.6); PSLI: 10.3–14.0 (11.8); PPI: 49.3–69.0 (60.6); PNI: 64.9–68.8 (67.5); MTI: 94.4–103.9 (98.9).

***Head.*** Cephalic margin straight or indistinctly convex (Fig. 53A). Pilosity moderately dense, long, and erect. Head sculpture variable, from smooth with foveolae restricted to anterior or medial frons to foveolate with smooth patches on vertex and area posterolateral from eyes; foveolae always sparse, frons sometimes with additional, sparse, and irregular rugae; antennal sockets with few thick, curved outward rugae and smooth to foveolae interspaces. Clypeus with median longitudinal carina absent; two lateral longitudinal carinae absent. Scape, when laid back, surpassing the posterior head margin by one-fifth of its length; pilosity dense, suberect to erect (Fig. 53A, C). ***Mesosoma.*** In lateral view, promesonotum moderately high, short, arched; promesonotal groove present, sometimes indistinct; metanotal groove present and distinct; propodeal spines very small and triangular, apex acute (Fig. 53C). Sculpture smooth and shiny, only dorsum with few transverse, thick rugulae and propodeum with indistinct and sparse foveolae. Pilosity sparse, moderately long, and erect (Fig. 53C, E). ***Petiole.*** Peduncle short and thin with ventral face slightly convex(Fig. 53C, E). ***Postpetiole.*** Short, low, and convex; with few short, erect setae (Fig. 53C, E). ***Gaster.*** With sparse, erect pilosity (Fig. 53C, E). ***Colour.*** Orange to yellow (Fig. 53C, E).

##### Biology.

The species was collected between 20–1580 m in elevation, in montane and littoral rainforest. Nests were located in rotten logs, dead twigs above ground, under moss, and rot pockets above ground.

##### Comments.

Major workers of *Pheidole sikorae* can be easily separated from all other Malagasy *Pheidole* based on strongly reduced sculpture of mesosoma and presence of impressed and smooth concavity placed lateral to antennal socket and tentorial pit. However, minor workers of *P. sikorae* have highly variable head sculpture. In most cases the head is predominantly smooth with sparse foveolae restricted to anterior and medial frons, but sometimes sparse foveolae extend to the whole frons and smooth patches are present only on vertex and area posterolateral from eyes. Minors of *P. sikorae* with strongly developed head sculpture are indistinguishable from minor workers of *P. antranohofa* sp. nov., a species known from Parc National de Marojejy in Antsiranana. Distribution ranges of these taxa do not overlap.

#### Pheidole sofia sp. nov.

Taxon classificationAnimalia

4A422666-4CEB-5246-9883-31C1E9F5CD4D

http://zoobank.org/058F9EDA-A9D8-4C24-9FDE-6B3D96C16D23

**Figure 54.**
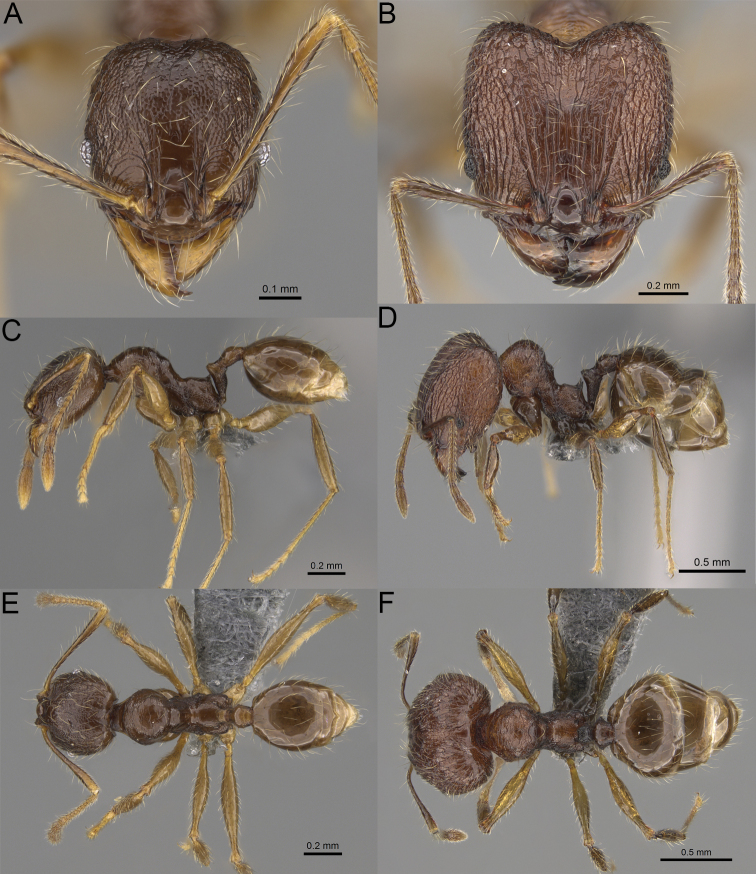
*Pheidole sofia* sp. nov., full-face view (**A**), profile (**C**), and dorsal view (**E**) of paratype minor worker (CASENT0923280) and full-face view (**B**), profile (**D**), and dorsal view (**F**) of holotype major worker (CASENT0705626).

Figs 54A–F , 64L , 67D

##### Type material.

***Holotype.*** Madagascar. • 1 major worker; Mahajanga; Region Sofia, Bemanevika; -14.32826, 48.58406; alt. 1657 m; 17 Jan 2015; B. L. Fisher et al. leg.; montane rainforest, ex rotten log; BLF35790; CASENT0705626 (CASC). ***Paratype.*** • 1w.; same data as for holotype, CASENT0923280 (CASC).

##### Other material.

Madagascar. –**Antsiranana**: • 2w., 1s.; Parc National Montagne d’Ambre, Crête; -12.58132, 49.13368; alt. 1110 m; 13 Nov 2007; B. L. Fisher et al. leg.; montane rainforest, ex rotten log; BLF18090 (CASC). • 2w., 2s.; Parc National Montagne d’Ambre, Mahasarika; -12.53176, 49.17662; alt. 1135 m; 17 Nov 2007; B. L. Fisher et al. leg.; montane rainforest, ex rotten log; BLF18458 (CASC). • 1w., 1s.; Parc National Montagne d’Ambre, Roussettes; -12.52574, 49.17238; alt. 1025 m; 15 Nov 2007; B. L. Fisher et al. leg.; montane rainforest, ex rotten log; BLF18310 (CASC). • 1w., 1s.; Parc National Montagne d’Ambre, Roussettes; -12.52574, 49.17238; alt. 1025 m; 15 Nov 2007; B. L. Fisher et al. leg.; montane rainforest, ex rotten log; BLF18336 (CASC).–**Fianarantsoa**: • 2w., 1s.; Ambositra; -20.53883, 47.24433; alt. 1330 m; 13 Dec 2006; B. L. Fisher et al. leg.; urban/garden, ground nest; BLF16210 (CASC).–**Mahajanga**: • 1w.; Region Sofia, Ampotsidia; -14.42935, 48.69863; alt. 1193 m; 7 Jan 2017; B. L. Fisher et al. leg.; montane ravine forest, ex rotten log; BLF39608 (CASC). • 1w.; Region Sofia, Ampotsidia; -14.42935, 48.69863; alt. 1193 m; 7 Jan 2017; B. L. Fisher et al. leg.; montane ravine forest, ex rotten log; BLF39652 (CASC). • 1w., 1s.; Region Sofia, Bemanevika; -14.33886, 48.58729; alt. 1567 m; 13 Jan 2015; B. L. Fisher et al. leg.; montane rainforest, ex rotten log; BLF35588 (CASC). • 2w., 1s., 1q.; Region Sofia, Bemanevika; -14.337, 48.58874; alt. 1606 m; 15 Jan 2015; B. L. Fisher et al. leg.; montane rainforest, ex rotten log; BLF35718 (CASC). • 1w., 1s.; Region Sofia, Bemanevika; -14.34517, 48.58948; alt. 1547 m; 16 Jan 2015; B. L. Fisher et al. leg.; montane rainforest, ex rotten log; BLF35772 (CASC). • 1w., 1s.; Region Sofia, Bemanevika; -14.32826, 48.58406; alt. 1657 m; 19 Jan 2015; B. L. Fisher et al. leg.; montane rainforest, ex rotten log; BLF35808 (CASC). –**Toamasina**: • 1w., 1s.; Reserve Betampona, Camp Rendrirendry 34.1 km 332° Toamasina; -17.924, 49.19967; alt. 390 m;29 Nov 2005; B. L. Fisher et al. leg.; rainforest, ex rotten log; BLF13135 (CASC). • 1w., 1s.; Réserve Nationale Intégrale Betampona, Betampona 35.1 km NW Toamasina; -17.91801, 49.20074; alt. 500 m; 16 Dec 2007; B. L. Fisher et al. leg.; rainforest, ex rotten stick on ground; BLF19608 (CASC).

##### Diagnosis.

Moderately large species. ***Major workers.*** Head in full-face view sub-oval, not widening posteriorly, with anterior and posterior sides convex, slightly narrowing posteriorly, in lateral view sub-oval; ventral and dorsal faces convex; sides of the head with dense, moderately long, suberect to erect pilosity; medial part of frons with thick, moderately sparse, longitudinal and interrupted rugae, posteromedial part with rugae more irregular and slightly directed outward, interspaces smooth to finely foveolate; anterolateral sides with thick, moderately dense, and longitudinally irregular rugae, posterolateral sides with rugae more irregular, interspaces with dense and distinct foveolae; occipital lobes and area posterolateral from eyes without smooth notches; scape, when laid back, exceeding the midlength of head by two-fifths of its length; inner hypostomal teeth distinct, large, closely spaced, triangular, with rounded apex directed slightly inward; outer hypostomal teeth lobe-like, slightly wider than and approximately the same height as inner hypostomal teeth, apex directed slightly outward; inner and outer hypostomal teeth closely spaced and not connected by concavity; mesosoma with fine rugofoveolae; pronotal dorsum with reduced sculpture and additional thin, interrupted, and transverse rugae; lateral sides of pronotum and katepisternum with smooth notches; gaster smooth; body ferruginous. ***Minor workers.*** Head sculpture variable; predominantly smooth or foveolae sparse and fading on medial frons and area posterolateral from eyes; vertex with sparse and short rugulae; frons with very sparse, short, and irregular rugae; area posterolateral from eyes with reduced sculpture or predominantly smooth; scape, when laid back, surpassing the posterior head margin by one-fifth of its length; promesonotum moderately high and short, arched; promesonotal groove present; propodeal spines small and triangular; mesosoma with sparse rugofoveolae; promesonotal dorsum and katepisternum with smooth notches or smooth; pronotum with additional sparse, short, and transverse rugulae; body brown.

##### Description.

**Major workers.** Measurements (*N* = 10): HL: 0.96–1.23 (1.1); HW: 0.97–1.16 (1.06); SL: 0.55–0.76 (0.66); EL: 0.11–0.12 (0.11); WL: 0.84–1.04 (0.95); PSL: 0.15–0.19 (0.17); MTL: 0.5–0.69 (0.6); PNW: 0.42–0.57 (0.48); PTW: 0.12–0.17 (0.15); PPW: 0.28–0.37 (0.31); CI: 99.3–106.0 (103.7); SI: 54.4–77.2 (63.0); PSLI: 14.3–17.3 (15.4); PPI: 43.5–53.7 (48.5); PNI: 42.9–49.2 (45.2); MTI: 50.7–67.1 (57.0).

***Head.*** In full-face view sub-oval, not widening posteriorly, with anterior and posterior sides convex, slightly narrowing posteriorly (Fig. 54B). In lateral view sub-oval; ventral and dorsal faces convex; inner hypostomal teeth visible. Sides of the head with dense, moderately long, suberect to erect pilosity; whole head with dense, long, decumbent to erect pilosity. Medial part of frons with thick, moderately sparse, longitudinal, and interrupted rugae, posteromedial part with rugae more irregular and slightly directed outward, interspaces smooth to finely foveolate; anterolateral sides with thick moderately dense and longitudinally irregular rugae, posterolateral sides with rugae more irregular, interspaces with dense and distinct foveolae. Occipital lobes with thick, irregular rugae and rugofoveolate interspaces. Area posterolateral from eyes rugoreticulate to rugofoveolate, sometimes with additional longitudinal to irregular, thick rugae. Gena with relatively dense and thick, longitudinal rugae and rugoreticulate interspaces. Centre of clypeus smooth and shiny, lateral sides with indistinct rugulae; median notch present, moderately wide, and shallow; median longitudinal carina absent; lateral longitudinal carinae absent. Scape, when laid back, exceeding the midlength of head by two-fifths of its length; pilosity subdecumbent to erect (Fig. 54B, D). Inner hypostomal teeth distinct, large, closely spaced, triangular, with rounded apex directed slightly inward; outer hypostomal teeth lobe-like, slightly wider than and approximately the same height as inner hypostomal teeth, apex directed slightly outward; inner and outer hypostomal teeth closely spaced and not connected by concavity (Fig. 64L). ***Mesosoma.*** In lateral view, promesonotum short, angular, and moderately low, posterior mesonotum moderately steep, mesonotal process indistinct, tubercle-like; promesonotal groove absent; metanotal groove absent; propodeal spines moderately short, with moderately wide base and acute apex; humeral area laterally weakly produced (Fig. 54D). Surface shiny with fine rugofoveolae; pronotal dorsum with reduced sculpture and additional thin, interrupted, and transverse rugae; lateral sides of pronotum and katepisternum with smooth notches. Pilosity moderately dense, long, and erect (Fig. 54D, F). ***Petiole.*** Shiny with fine foveolae; node smooth to finely foveolate, low, triangular, with rounded and thin apex, in rear view node dorsoventrally straight to slightly convex; pilosity moderately sparse and erect (Fig. 54D, F). ***Postpetiole.*** Shiny and foveolate; dorsum with reduced sculpture and smooth notch; in dorsal view oval, lateral margins medially with two dentate projections; pilosity long, moderately sparse and erect (Fig. 54D, F). ***Gaster.*** Shiny and smooth; pilosity dense, long, and erect (Fig. 54D, F). ***Colour.*** Ferruginous; legs, gaster, and antennae bright brown (Fig. 54D, F).

**Minor workers.** Measurements (*N* = 10): HL: 0.46–0.62 (0.56); HW: 0.42–0.57 (0.49); SL: 0.51–0.67 (0.61); EL: 0.08–0.1 (0.09); WL: 0.58–0.78 (0.69); PSL: 0.06–0.11 (0.09); MTL: 0.36–0.53 (0.46); PNW: 0.28–0.39 (0.33); PTW: 0.08–0.1 (0.09); PPW: 0.11–0.17 (0.14); CI: 106.0–118.7 (123.4); SI: 112.1–139.2 (123.4); PSLI: 11.3–18.8 (16.2); PPI: 50.6–74.2 (60.6); PNI: 62.8–70.3 (67.6); MTI: 77.0–101.0 (92.3).

***Head.*** Cephalic margin indistinctly concave or straight (Fig. 54A). Pilosity relatively dense, long, decumbent to suberect. Sculpture shiny and variable; predominantly smooth or foveolate with foveolae sparse and fading on medial frons and area posterolateral from eyes; vertex with sparse and short rugulae; frons with very sparse, short, and irregular rugae; area posterolateral from eyes with reduced sculpture or predominantly smooth; antennal sockets with few thick, curved outward rugae and smooth to indistinctly foveolate interspaces. Clypeus with median longitudinal carina absent; two lateral longitudinal carinae absent. Scape, when laid back, surpassing the posterior head margin by one-fifth of its length; pilosity dense, subdecumbent to erect (Fig. 54A, C). ***Mesosoma.*** In lateral view, promesonotum moderately high and short, arched; promesonotal groove indistinct; metanotal groove distinct; propodeal spines small and triangular (Fig. 54C). Sculpture shiny with sparse rugofoveolae; promesonotal dorsum and katepisternum with smooth notches or smooth; pronotum with additional sparse, short, and transverse rugulae. Pilosity moderately sparse, long, and erect (Fig. 54C, E). ***Gaster.*** With sparse, erect pilosity (Fig. 54C, E). ***Colour.*** Brown, legs and antenna yellowish brown (Fig. 54C, E).

##### Etymology.

From the type locality.

##### Biology.

The species was collected between 390–1657 m in elevation, in rainforest, montane rainforest, montane ravine forest, and in urban areas. Nests were located in rotten logs, rotten sticks on the ground, and in soil.

##### Comments.

*Pheidole sofia* sp. nov. is a widespread species known from Ambositra in Fianarantsoa north to Joffreville in Antsiranana. The species is most similar to parapatric *P. manantenina* sp. nov., known from Parc National de Marojejy in Antsiranana. Major workers of *P. sofia* sp. nov. differ from *P. manantenina* sp. nov. in medial part of frons with interspaces finely foveolate, lateral sides of frons with more longitudinally irregular rugae, body ferruginous, and promesonotum never with reduced sculpture; minor workers can be separated based on more distinct sculpture on head, and mesosoma with sparse rugofoveolae, promesonotal dorsum and katepisternum with smooth notches or smooth, and presence of additional sparse, short, and transverse rugulae on pronotum.

#### Pheidole sparsa sp. nov.

Taxon classificationAnimalia

38378491-5C09-5E3D-B4EB-DFEBAE9B3954

http://zoobank.org/C1B77D52-778D-4B0D-9DAA-308706043971

**Figure 55.**
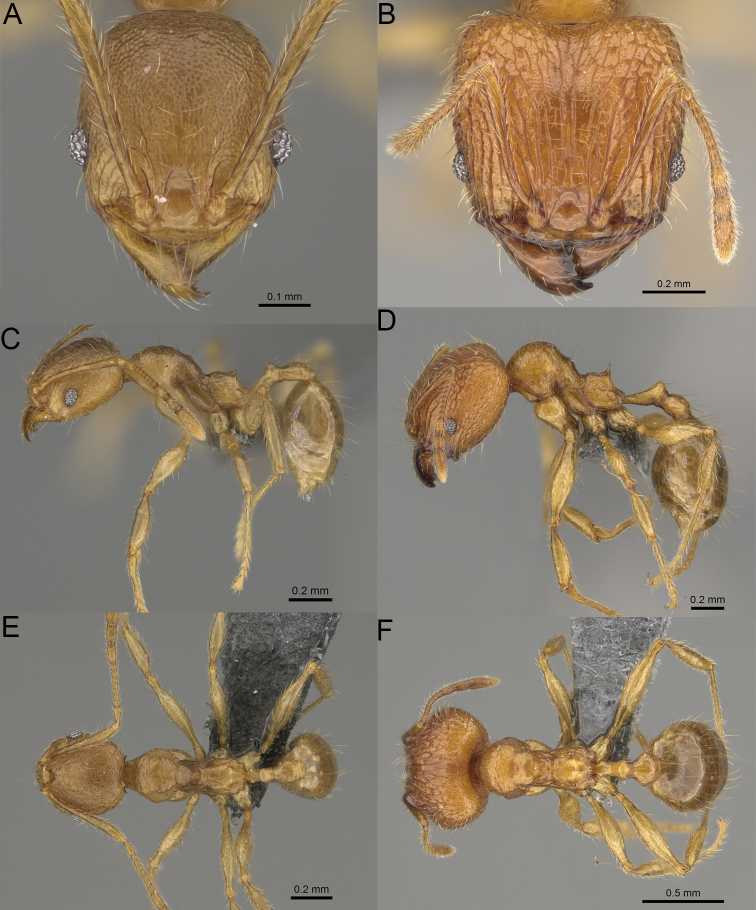
*Pheidole sparsa* sp. nov., full-face view (**A**), profile (**C**), and dorsal view (**E**) of paratype minor worker (CASENT0923289) and full-face view (**B**), profile (**D**), and dorsal view (**F**) of holotype major worker (CASENT0705586).

Figs 55A–F , 64M , 67E

##### Type material.

***Holotype.*** Madagascar. • 1 major worker; Mahajanga; Region Sofia, Bemanevika; -14.337, 48.58874; alt. 1606 m; 14 Jan 2015; B. L. Fisher et al. leg.; montane rainforest, ex rotten log; BLF35640; CASENT0705586 (CASC). ***Paratype.*** • 1w.; same data as for holotype; CASENT0923289 (CASC).

##### Other material.

Madagascar. –**Mahajanga**: •1w., 1s.; Region Sofia, Bemanevika; -14.337, 48.58874; alt. 1606 m; 14 Jan 2015; B. L. Fisher et al. leg.; montane rainforest, ex rotten log; BLF35624 (CASC). • 1w., 1s.; Region Sofia, Bemanevika; -14.337, 48.58874; alt. 1606 m; 14 Jan 2015; B. L. Fisher et al. leg.; montane rainforest, ex rotten log; BLF35664 (CASC). • 1w., 1s.; Region Sofia, Bemanevika; -14.337, 48.58874; alt. 1606 m; 15 Jan 2015; B. L. Fisher et al. leg.; montane rainforest, ex rotten log; BLF35713 (CASC).

##### Diagnosis.

Moderately large species. ***Major workers.*** Head in full-face view sub-oval, not widening posteriorly, with anterior and posterior sides relatively straight, in lateral view sub-oval; ventral and dorsal faces convex; sides of the head with dense, moderately long, suberect to erect pilosity; medial part of frons with moderately sparse, thick, longitudinal, and interrupted rugae, rugae in posteromedial slightly directed outward, interspaces shiny with sparse and indistinct rugofoveolae; anterolateral sides with longitudinal, thick, and relatively sparse rugae; posterolateral sides with irregular, thick, and relatively sparse rugae; interspaces shiny with sparse and indistinct rugofoveolae; occipital lobes and area posterolateral from eyes without smooth notches; scape, when laid back, exceeding the midlength of head by two-fifths of its length; inner hypostomal teeth distinct, large, closely spaced, triangular, with rounded apex directed upward; outer hypostomal teeth lobe-like, approximately the same height and width as inner hypostomal teeth, apex directed outward; inner and outer hypostomal teeth closely spaced and not connected by concavity; promesonotum predominantly smooth, with indistinct rugofoveolae on lateral sides; katepisternum rugofoveolate with smooth notch; propodeum rugofoveolate; gaster smooth; body dark orange. ***Minor workers.*** Head foveolate; medial part of frons with smooth notch and indistinct, short rugulae; area posterolateral from eyes predominantly smooth; scape, when laid back, surpassing the posterior head margin by one-fifth of its length; promesonotum moderately low and moderately long, arched; promesonotal groove absent; propodeal spines small and triangular; pronotum with very indistinct and sparse foveolae; mesonotum, anepisternum, katepisternum, and propodeum smooth; body dark yellow.

##### Description.

**Major workers.** Measurements (*N* = 4): HL: 0.94–1.13 (1.06); HW: 0.94–1.1 (1.04); SL: 0.61–0.72 (0.67); EL: 0.12–0.14 (0.13); WL: 0.93–1.0 (0.98); PSL: 0.16–0.2 (0.18); MTL: 0.6–0.65 (0.62); PNW: 0.41–0.5 (0.45); PTW: 0.14–0.18 (0.16); PPW: 0.27–0.32 (0.3); CI: 99.0–103.0 (101.7); SI: 62.7–65.5 (64.7); PSLI: 15.9–17.5 (16.7); PPI: 45.2–61.7 (53.9); PNI: 42.6–45.4 (43.6); MTI: 55.9–64.7 (59.4).

***Head.*** In full-face view sub-oval, not widening posteriorly, with anterior and posterior sides relatively straight (Fig. 55B). In lateral view sub-oval; ventral and dorsal faces convex; inner hypostomal teeth visible. Sides of the head with dense, moderately long, erect pilosity; whole head with dense, long, decumbent to erect pilosity. Medial part of frons with moderately sparse, thick, longitudinal, and interrupted rugae, posteromedial rugae slightly directed outward, interspaces shiny with sparse and indistinct rugofoveolae; anterolateral sides with longitudinal, thick, and relatively sparse rugae; posterolateral sides with irregular, thick, and relatively sparse rugae; interspaces shiny with sparse and indistinct rugofoveolae. Occipital lobes with sparse, thick, and irregular rugae; interspaces smooth. Gena with relatively sparse, thick, and longitudinal rugae and smooth interspaces. Area posterolateral from eyes with thin and dense rugofoveolae and smooth interspaces. Centre of clypeus smooth and shiny, lateral sides with indistinct rugulae; median notch present, moderately wide, and shallow; median longitudinal carina absent; lateral longitudinal carinae absent. Scape, when laid back, exceeding the midlength of head by two-fifths of its length; pilosity subdecumbent to erect (Fig. 55B, D). Inner hypostomal teeth distinct, large, closely spaced, triangular, with rounded apex directed upward; outer hypostomal teeth lobe-like, approximately the same height and width as inner hypostomal teeth, apex directed outward; inner and outer hypostomal teeth closely spaced and not connected by concavity (Fig. 64M). ***Mesosoma.*** In lateral view, promesonotum short, angular, and moderately low, posterior mesonotum moderately steep, mesonotal process indistinct, tubercle-like; promesonotal groove absent; metanotal groove indistinct; propodeal spines moderately long, moderately narrow, with acute apex; humeral area laterally weakly produced (Fig. 55D). Surface shiny; promesonotum predominantly smooth, with indistinct rugofoveolae on lateral sides; katepisternum rugofoveolae with smooth notch; propodeum rugofoveolate. Pilosity relatively dense, long, and erect (Fig. 55D, F). ***Petiole.*** Shiny with fine and sparse rugofoveolae; node smooth, low, triangular, with rounded and thin apex, in rear view node dorsoventrally slightly convex; pilosity moderately sparse and erect (Fig. 55D, F). ***Postpetiole.*** Shiny and smooth; in dorsal view oval, lateral margins medially with two dentate projections; pilosity long, moderately sparse, and erect (Fig. 55D, F). ***Gaster.*** Shiny and smooth; pilosity moderately dense, long, and erect (Fig. 55D, F). ***Colour.*** Dark orange; mandibles and gaster slightly darker; legs yellowish (Fig. 55D, F).

**Minor workers.** Measurements (*N* = 4): HL: 0.59–0.6 (0.59); HW: 0.49–0.52 (0.51); SL: 0.58–0.66 (0.62); EL: 0.09–0.11 (0.1); WL: 0.7–0.73 (0.72); PSL: 0.08–0.1 (0.09); MTL: 0.47–0.52 (0.49); PNW: 0.34–0.35 (0.34); PTW: 0.08–0.1 (0.09); PPW: 0.14–0.15 (0.14); CI: 116.0–119.6 (117.1); SI: 115.0–133.4 (123.3); PSLI: 14.0–16.2 (15.4); PPI: 53.1–69.9 (63.1); PNI: 65.3–69.6 (67.6); MTI: 92.9–102.4 (97.9).

***Head.*** Cephalic margin indistinctly convex or straight (Fig. 55A). Pilosity relatively sparse, long, decumbent to suberect. Sculpture foveolate; medial part of frons with smooth notch and indistinct, short rugulae; area posterolateral from eyes predominantly smooth. Clypeus with median longitudinal carina absent; two lateral longitudinal carinae absent. Scape, when laid back, surpassing the posterior head margin by one-fifth of its length; pilosity dense, subdecumbent to erect (Fig. 55A, C). ***Mesosoma.*** In lateral view, promesonotum moderately low and moderately long, arched; promesonotal groove absent; metanotal groove distinct; propodeal spines small and triangular (Fig. 55C). Pronotum with very indistinct and sparse foveolae; mesonotum, anepisternum, katepisternum, and propodeum smooth. Pilosity very sparse, moderately long, and erect (Fig. 55C, E). ***Postpetiole.*** Short, low, and relatively flat; with few short, erect setae (Fig. 55C, E). ***Gaster.*** With sparse, erect pilosity (Fig. 55C, E). ***Colour.*** Dark yellow, vertex and gaster slightly darker (Fig. 55C, E).

##### Etymology.

Latin for sparse in reference to sparse rugae on the frons.

##### Biology.

The species was collected at 1606 m in elevation, in montane rainforest. Nests were located in rotten logs.

##### Comments.

*Pheidole sparsa* sp. nov., described from Bemanevika in Mahajanga, is most similar to *P. dasos* sp. nov., known from Makirovana forest in Antsiranana, and *P. hazo* sp. nov., recorded so far only from the vicinity of Andranoma in Antananarivo. Majors of *P. sparsa* sp. nov. can be distinguished from both taxa mentioned above by medial part of frons with moderately sparse rugae, interspaces with sparse and indistinct rugofoveolae, and predominantly smooth promesonotum with indistinct rugofoveolae on lateral sides; minors of *P. sparsa* sp. nov. can be distinguished from *P. hazo* sp. nov. by dark yellow body colouration and propodeum with indistinct and sparse foveolae, and from *P. dasos* sp. nov. by dark yellow body colouration, shorter scape surpassing the posterior head margin by one-fifth of its length, and pronotum with very indistinct and sparse foveolae.

#### Pheidole tampony sp. nov.

Taxon classificationAnimalia

2FE0B42C-907B-5574-94E8-4C289EFD1EE0

http://zoobank.org/05966990-3AEA-4921-888E-941D151DFCC1

**Figure 56.**
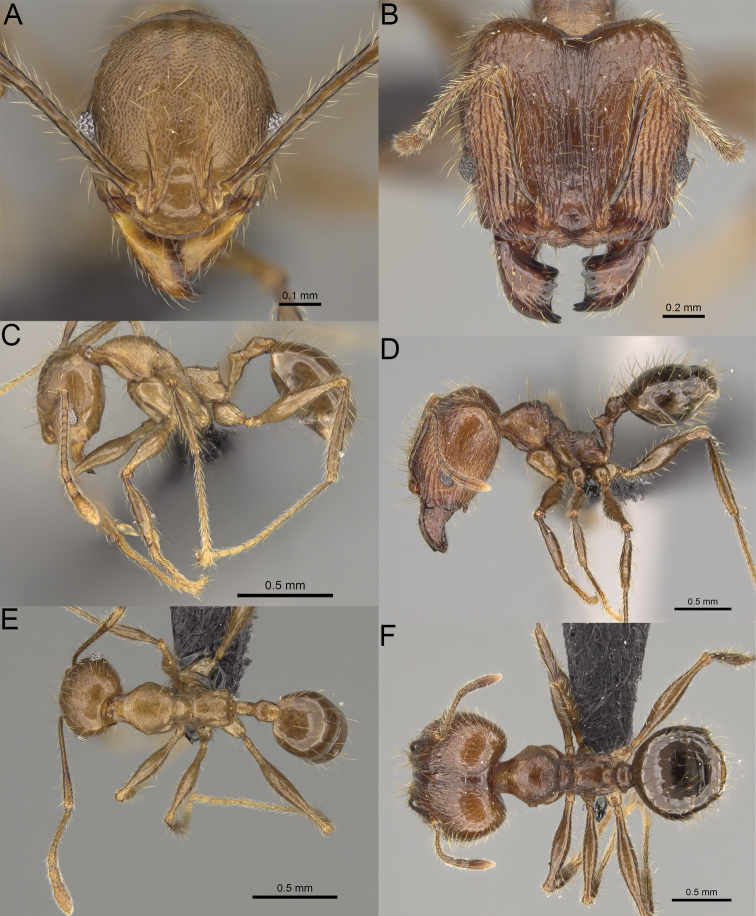
*Pheidole tampony* sp. nov., full-face view (**A**), profile (**C**), and dorsal view (**E**) of paratype minor worker (CASENT0923285) and full-face view (**B**), profile (**D**), and dorsal view (**F**) of holotype major worker (CASENT0809550).

Figs 56A–F , 64N , 67F

##### Type material.

***Holotype.*** Madagascar. • 1 major worker; Antsiranana; Sava Region: Parc National de Marojejy, near summit, 25.4 km 20.1°NE Andapa; -14.44918, 49.73243; alt. 2100 m; 10 Feb 2018; B. L. Fisher et al. leg.; montane shrubland, under rootmat, on stone; BLF40950; CASENT0809550 (CASC). ***Paratype.*** • 1w.; same data as for holotype, CASENT0923285 (CASC).

##### Diagnosis.

Moderately large species. ***Major workers.*** Head in full-face view sub-oval and not widening posteriorly, with anterior and posterior sides slightly convex, in lateral view sub-oval; ventral and dorsal faces convex; sides of the head with thick, interrupted, dense, and longitudinal rugae with smooth to indistinctly rugulate interspaces; occipital lobes predominantly smooth, only anterior part with indistinct longitudinal rugae; denser and thinner longitudinal rugae with rugulate interspaces; area posterolateral from eyes with reduced sculpture and predominantly smooth; scape, when laid back, exceeding the midlength of head by approximately two-fifths of its length; inner hypostomal teeth distinct, distinct, moderately large, closely spaced, triangular, with rounded apex directed upward; outer hypostomal teeth lobe-like, narrower than and approximately as high as inner teeth, apex directed outward; inner and outer hypostomal teeth closely spaced and not connected by concavity; mesosoma rugofoveolate; pronotum with sparse foveolae, its dorsum with smooth notch; katepisternum with large, smooth notch; body brown. ***Minor workers.*** Head foveolate; median frons with short and indistinct longitudinal rugulae; vertex with fading sculpture; area posterolateral from eyes smooth; scape, when laid back, exceeding the posterior head margin by one-third of its length; promesonotum low and moderately long; promesonotal groove present; propodeal spines small and triangular; mesosoma foveolate; body bright brown.

##### Description.

**Major workers.** Measurements (*N* = 1): HL: 1.36; HW: 1.36; SL: 0.87; EL: 0.19; WL: 1.34; PSL: 0.23; MTL: 0.89; PNW: 0.5; PTW: 0.18; PPW: 0.34; CI: 100.1; SI: 64.0; PSLI: 17.0; PPI: 52.1; PNI: 36.6; MTI: 65.4.

***Head.*** In full-face view sub-oval, not widening posteriorly, with anterior and posterior sides slightly convex (Fig. 56B). In lateral view sub-oval; ventral and dorsal faces convex; inner hypostomal teeth visible. Sides of the head with dense, long, erect pilosity; whole head with dense, long, decumbent to erect pilosity. Medial part of frons with thick, interrupted, dense, and longitudinal rugae with smooth to indistinctly rugulate interspaces; lateral sides of frons with dense, thick, and longitudinal rugae, interspaces with dense rugulae. Occipital lobes predominantly smooth, only anterior part with indistinct longitudinal rugae. Area posterolateral from eyes with denser and thinner longitudinal rugae with rugulate interspaces, posteriormost parts with reduced sculpture and predominantly smooth. Gena with relatively sparse, thick, and longitudinal rugae with distinctly rugulate interspaces. Centre of clypeus indistinctly foveolate and having shiny, lateral sides with indistinct rugulae; median notch present, moderately wide, and shallow; median longitudinal carina present; lateral longitudinal carinae absent. Scape, when laid back, exceeding the midlength of head by two-fifths of its length; pilosity subdecumbent to erect (Fig. 56B, D). Inner hypostomal teeth distinct, moderately large, closely spaced, triangular, with rounded apex directed upward; outer hypostomal teeth lobe-like, narrower than and approximately as high as inner teeth, apex directed outward; inner and outer hypostomal teeth closely spaced and not connected by concavity (Fig. 64N). ***Mesosoma.*** In lateral view, promesonotum short, angular, and moderately low, posterior mesonotum moderately steep, mesonotal process indistinct, tubercle-like; promesonotal groove absent; metanotal groove absent; propodeal spines moderately long, with narrow base and acute apex; humeral area produced (Fig. 56D). Surface shiny and rugofoveolate; pronotum with sparse foveolae, its dorsum with smooth notch; katepisternum with large, smooth notch. Pilosity moderately dense, long, and erect (Fig. 56D, F). ***Petiole.*** Shiny with dense foveolae; node finely foveolate, triangular, with rounded and thick apex, in rear view node dorsoventrally slightly convex; pilosity moderately sparse and erect (Fig. 56D, F). ***Postpetiole.*** Shiny and foveolate; dorsum with reduced sculpture and smooth notch; in dorsal view oval, lateral margins medially with two dentate projections; pilosity long, moderately sparse, and erect (Fig. 56D, F). ***Gaster.*** Shiny and smooth; pilosity moderately dense, long, and erect (Fig. 56D, F). ***Colour.*** Brown, gaster slightly darker (Fig. 56D, F).

**Minor workers.** Measurements (*N* = 1): HL: 0.68; HW: 0.57; SL: 0.81; EL: 0.13; WL: 0.93; PSL: 0.12; MTL: 0.65; PNW: 0.4; PTW: 0.09; PPW: 0.16; CI: 119.5; SI: 141.6; PSLI: 17.0; PPI: 54.7; PNI: 69.6; MTI: 114.2.

***Head.*** Cephalic margin slightly convex (Fig. 56A). Pilosity relatively sparse, moderately long, subdecumbent to erect. Sculpture shiny and foveolate; median frons with short and indistinct longitudinal rugulae; vertex with fading sculpture; area posterolateral from eyes smooth; antennal sockets with few indistinct, curved outward rugae and foveolate interspaces. Clypeus with median longitudinal carina absent; two lateral longitudinal carinae absent. Scape, when laid back, exceeding the posterior head margin by one-third of its length; pilosity dense, subdecumbent to erect (Fig. 56A, C). ***Mesosoma.*** In lateral view, promesonotum low and moderately long, arched; promesonotal groove indistinct; metanotal groove distinct; propodeal spines small and triangular (Fig. 56C). Sculpture shiny and foveolate. Pilosity very sparse, moderately long, and erect (Fig. 56C, E). ***Gaster.*** With sparse, erect pilosity (Fig. 56C, E). ***Colour.*** Bright brown (Fig. 56C, E).

##### Etymology.

Malagasy for summit in reference to the type locality located close to the mountain peak.

##### Biology.

The species was collected at 2100 m in elevation, in montane shrubland. Nest was located under rootmat on stone.

##### Comments.

*Pheidole tampony* sp. nov. is a member of a group of species characterised by distinctly reduced head sculpture in major workers with occipital lobes entirely or predominantly smooth, area posterolateral from eyes partially or entirely smooth and shiny or with reduced sculpture and smooth notches. The group consists of four species: *P. litigiosa*, *P. masoandro* sp. nov., *P. gracilis* sp. nov., and *P. tampony* sp. nov. *Pheidole tampony* sp. nov. is known only from Parc National de Marojejy in Antsiranana and its distribution does not overlap with other members of this group. Both minor and major workers of this species can be easily separated from other members of this group based on darker, bright brown to brown body colouration. However, major workers of *P. tampony* sp. nov. can be confused with majors of sympatric *P. manantenina* sp. nov. *Pheidole tampony* sp. nov. can be separated based on presence of distinct smooth notch on posteriormost part of area posterolateral from eyes and partially smooth occipital lobes (*P. manantenina* sp. nov. has posteriormost part of area posterolateral from eyes and occipital lobes with sparse and reduced sculpture that are never smooth), lower promesonotum with more distinctly developed mesonotal process (*P. manantenina* sp. nov. has promesonotum higher and lacking mesonotal process), and smooth notch on katepisternum (*P. manantenina* sp. nov. has katepisternum entirely sculptured). Both taxa distinctly differ in minor workers: *P. tampony* sp. nov. has minors with predominantly foveolate head and mesosoma and long and low promesonotum, while minors of *P. manantenina* sp. nov. have head and mesosoma predominantly smooth and shorter and higher promesonotum.

#### Pheidole trichotos sp. nov.

Taxon classificationAnimalia

200DB3C8-572B-5FEE-A1A5-63B4524816B0

http://zoobank.org/E6826B9F-4FA4-406E-8B6A-430F5C28C355

**Figure 57.**
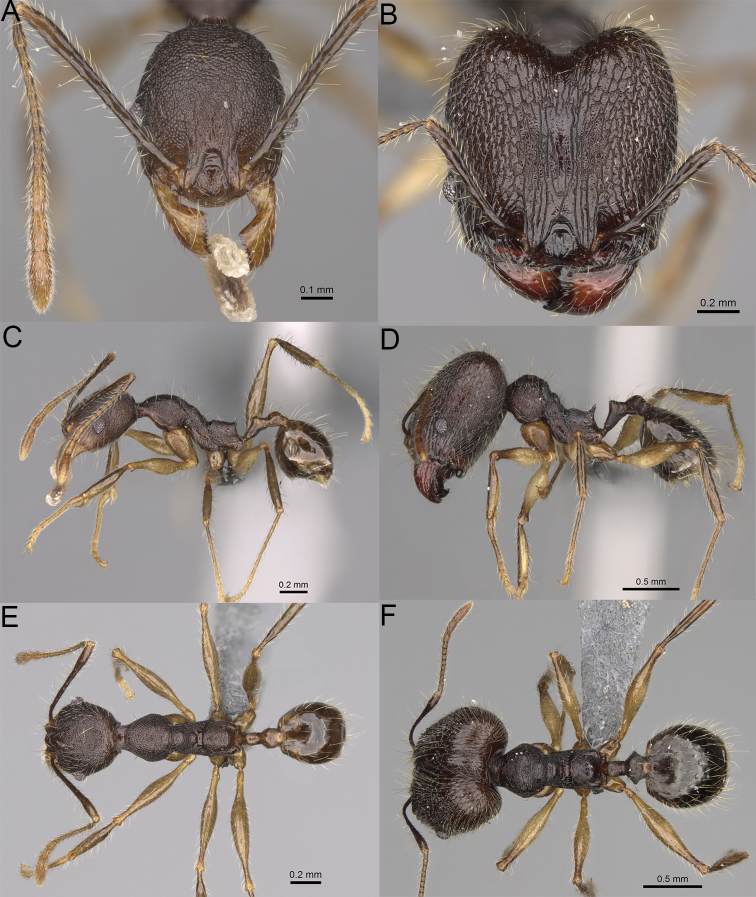
*Pheidole trichotos* sp. nov., full-face view (**A**), profile (**C**), and dorsal view (**E**) of paratype minor worker (CASENT0923258) and full-face view (**B**), profile (**D**), and dorsal view (**F**) of holotype major worker (CASENT0788058).

Figs 57A–F , 64O , 67G

##### Type material.

***Holotype.*** Madagascar. • 1 major worker; Mahajanga; Region Sofia, Ampotsidia; -14.41592, 48.71118; alt. 1364 m; 11 Jan 2017; B. L. Fisher et al. leg.; montane rainforest, ex rotten log; BLF39812; CASENT0788058 (CASC). ***Paratype.*** • 1w.; same data as for holotype, CASENT0923258 (CASC).

##### Other material.

Madagascar. –**Antsiranana**: • 1w.; 11.0 km WSW Befingotra, Rés. Anjanaharibe-Sud; -14.75, 49.45; alt. 1565 m; 16 Nov 1994; B. L. Fisher et al. leg.; montane rainforest; sifted litter (leaf mold, rotten wood); BLF01232 (CASC). • 1w.; Antongombato, 2.2 km SW Antsiranana; -12.37277, 49.22888; alt. 74 m; 14 Aug 2015; B. L. Fisher et al. leg.; urban/garden, ex rotten log; BLF37252 (CASC). • 1w.; Antongombato, 2.2 km SW Antsiranana; -12.37277, 49.22888; alt. 74 m; 14 Aug 2015; B. L. Fisher et al. leg.; urban/garden, ground forager(s); BLF37271 (CASC). • 1w., 1s.; Antongombato, 2.2 km SW Antsiranana; -12.37277, 49.22888; alt. 74 m; 14 Aug 2015; B. L. Fisher et al. leg.; urban/garden, ex rotten log; BLF37273 (CASC). • 1s.; Parc National de Marojejy, Antranohofa, 26.6 km 31°NNE Andapa, 10.7 km 318°NW Manantenina; -14.44333, 49.74333; alt. 1325 m; 18 Nov 2003; B. L. Fisher et al. leg.; montane rainforest, sifted litter (leaf mold, rotten wood); BLF09080 (CASC). • 3w., 1s.; Parc National Montagne d’Ambre, 12.2 km 211°SSW Joffreville; -12.59639, 49.1595; alt. 1300 m; 2 Feb 2001; B. L. Fisher et al. leg.; montane rainforest; BLF02782 (CASC). • 2w.; Parc National Montagne d’Ambre, 12.2 km 211°SSW Joffreville; -12.59639, 49.1595; alt. 1300 m; 2 Feb 2001; B. L. Fisher et al. leg.; montane rainforest, canopy moss and leaf litter; BLF02784 (CASC). • 3w., 2s.; Parc National Montagne d’Ambre, 12.2 km 211°SSW Joffreville; -12.59639, 49.1595; alt. 1300 m; 2 Feb 2001; B. L. Fisher et al. leg.; montane rainforest, ex rotten log; BLF02787 (CASC). • 1w., 1s.; Parc National Montagne d’Ambre, 12.2 km 211°SSW Joffreville; -12.59639, 49.1595; alt. 1300 m; 2 Feb 2001; B. L. Fisher et al. leg.; montane rainforest, ex rotten log; BLF02788 (CASC). • 1w.; Parc National Montagne d’Ambre, 12.2 km 211°SSW Joffreville; -12.59639, 49.1595; alt. 1300 m; 2 Feb 2001; B. L. Fisher et al. leg.; montane rainforest, ex rotten log; BLF02790 (CASC). • 1w., 2s.; Parc National Montagne d’Ambre, 12.2 km 211°SSW Joffreville; -12.59639, 49.1595; alt. 1300 m; 2 Feb 2001; B. L. Fisher et al. leg.; montane rainforest, ex rotten log; BLF02791 (CASC). • 1w.; Parc National Montagne d’Ambre, 12.2 km 211°SSW Joffreville; -12.59639, 49.1595; alt. 1300 m; 2 Feb 2001; B. L. Fisher et al. leg.; montane rainforest, ex rotten log; BLF02795 (CASC). • 1w.; Parc National Montagne d’Ambre, 12.2 km 211°SSW Joffreville; -12.59639, 49.1595; alt. 1300 m; 2 Feb 2001; B. L. Fisher et al. leg.; montane rainforest, ex rotten log; BLF02796 (CASC). • 1w.; Parc National Montagne d’Ambre, 12.2 km 211°SSW Joffreville; -12.59639, 49.1595; alt. 1300 m; 2 Feb 2001; B. L. Fisher et al. leg.; montane rainforest, ex dead twig above ground; BLF02815 (CASC). • 2w.; Parc National Montagne d’Ambre, 12.2 km 211°SSW Joffreville; -12.59639, 49.1595; alt. 1300 m; 2 Feb 2001; B. L. Fisher et al. leg.; montane rainforest, ex dead twig above ground; BLF02821 (CASC). • 3w., 2s.; Parc National Montagne d’Ambre, 12.2 km 211°SSW Joffreville; -12.59639, 49.1595; alt. 1300 m; 2 Feb 2001; B. L. Fisher et al. leg.; montane rainforest, ex rotten tree stump; BLF02836 (CASC). • 2w., 1s.; Parc National Montagne d’Ambre, 12.2 km 211°SSW Joffreville; -12.59639, 49.1595; alt. 1300 m; 2 Feb 2001; B. L. Fisher et al. leg.; montane rainforest, ex rotten tree stump; BLF02838 (CASC). • 1w.; Parc National Montagne d’Ambre, 12.2 km 211°SSW Joffreville; -12.59639, 49.1595; alt. 1300 m; 2 Feb 2001; B. L. Fisher et al. leg.; montane rainforest; BLF02853 (CASC). • 1w.; Parc National Montagne d’Ambre, 3.6 km 235°SW Joffreville; -12.53444, 49.1795; alt. 925 m; 20 Jan 2001; B. L. Fisher et al. leg.; montane rainforest, sifted litter (leaf mold, rotten wood); BLF02564 (CASC). • 1w., 1s.; Parc National Montagne d’Ambre, Ambre grand lac; -12.59656, 49.15932; alt. 1350 m; 13 Nov 2007; B. L. Fisher et al. leg.; montane rainforest, ex rotten log; BLF18050 (CASC). • 1w., 1s.; Parc National Montagne d’Ambre, Ambre grand lac; -12.59656, 49.15932; alt. 1350 m; 13 Nov 2007; B. L. Fisher et al. leg.; montane rainforest, ex rotten log; BLF18054 (CASC). • 1w., 1s.; Parc National Montagne d’Ambre, Ambre grand lac; -12.59656, 49.15932; alt. 1350 m; 13 Nov 2007; B. L. Fisher et al. leg.; montane rainforest, ex rotten log; BLF18060 (CASC). • 1w., 1s.; Parc National Montagne d’Ambre, Ambre grand lac; -12.59656, 49.15932; alt. 1350 m; 13 Nov 2007; B. L. Fisher et al. leg.; montane rainforest, ex rotten log; BLF18062 (CASC). –**Mahajanga**: • 1w., 1s.; Region Sofia, Ampotsidia; -14.40775, 48.70201; alt. 1625 m; 10 Jan 2017; B. L. Fisher et al. leg.; montane rainforest, ex rotten log; BLF39784 (CASC). • 10w., 10s.; Region Sofia, Bemanevika; -14.33886, 48.58729; alt. 1567 m; 13 Jan 2015; B. L. Fisher et al. leg.; montane rainforest, sifted litter; BLF35555 (CASC). • 1w., 1s.; Region Sofia, Bemanevika; -14.33886, 48.58729; alt. 1567 m; 13 Jan 2015; B. L. Fisher et al. leg.; montane rainforest, ex rotten log; BLF35567 (CASC). • 2w., 1s., 1q.; Region Sofia, Bemanevika; -14.33886, 48.58729; alt. 1567 m; 13 Jan 2015; B. L. Fisher et al. leg.; montane rainforest, ex rotten log; BLF35569 (CASC). • 1w., 1s.; Region Sofia, Bemanevika; -14.33886, 48.58729; alt. 1567 m; 13 Jan 2015; B. L. Fisher et al. leg.; montane rainforest, ex rotten log; BLF35575 (CASC). • 1w., 1s.; Region Sofia, Bemanevika; -14.33886, 48.58729; alt. 1567 m; 13 Jan 2015; B. L. Fisher et al. leg.; montane rainforest, ex rotten log; BLF35582 (CASC). • 1w., 1s.; Region Sofia, Bemanevika; -14.33886, 48.58729; alt. 1567 m; 13 Jan 2015; B. L. Fisher et al. leg.; montane rainforest, ex rotten log; BLF35592 (CASC). • 1w., 1s.; Region Sofia, Bemanevika; -14.33886, 48.58729; alt. 1567 m; 13 Jan 2015; B. L. Fisher et al. leg.; montane rainforest, under rotten log; BLF35593 (CASC). • 1w., 1s.; Region Sofia, Bemanevika; -14.33886, 48.58729; alt. 1567 m; 13 Jan 2015; B. L. Fisher et al. leg.; montane rainforest, ex rotten log; BLF35601 (CASC). • 1w., 1s.; Region Sofia, Bemanevika; -14.33886, 48.58729; alt. 1567 m; 13 Jan 2015; B. L. Fisher et al. leg.; montane rainforest, ex rotten log; BLF35601 (CASC). • 1w., 1s.; Region Sofia, Bemanevika; -14.33886, 48.58729; alt. 1567 m; 13 Jan 2015; B. L. Fisher et al. leg.; montane rainforest, ex rotten log; BLF35605 (CASC). • 1w., 1s.; Region Sofia, Bemanevika; -14.33886, 48.58729; alt. 1567 m; 13 Jan 2015; B. L. Fisher et al. leg.; montane rainforest, ex rotten log; BLF35606 (CASC). • 1w., 1s.; Region Sofia, Bemanevika; -14.337, 48.58874; alt. 1606 m; 14 Jan 2015; B. L. Fisher et al. leg.; montane rainforest, ex rotten log; BLF35630 (CASC). • 1w., 1s.; Region Sofia, Bemanevika; -14.337, 48.58874; alt. 1606 m; 14 Jan 2015; B. L. Fisher et al. leg.; montane rainforest, ex rotten log; BLF35645 (CASC). • 1w., 1s.; Region Sofia, Bemanevika; -14.337, 48.58874; alt. 1606 m; 14 Jan 2015; B. L. Fisher et al. leg.; montane rainforest, ex rotten log; BLF35653 (CASC). • 1w., 1s.; Region Sofia, Bemanevika; -14.337, 48.58874; alt. 1606 m; 15 Jan 2015; B. L. Fisher et al. leg.; montane rainforest, ex rotten log; BLF35671 (CASC). • 2w., 1s., 1q; Region Sofia, Bemanevika; -14.337, 48.58874; alt. 1606 m; 15 Jan 2015; B. L. Fisher et al. leg.; montane rainforest, ex rotten log; BLF35674 (CASC). • 2w., 1s., 1q.; Region Sofia, Bemanevika; -14.337, 48.58874; alt. 1606 m; 15 Jan 2015; B. L. Fisher et al. leg.; montane rainforest, ex rotten log; BLF35689 (CASC). • 1w., 1s.; Region Sofia, Bemanevika; -14.337, 48.58874; alt. 1606 m; 15 Jan 2015; B. L. Fisher et al. leg.; montane rainforest, ex rotten log; BLF35691 (CASC). • 1w., 1s.; Region Sofia, Bemanevika; -14.337, 48.58874; alt. 1606 m; 15 Jan 2015; B. L. Fisher et al. leg.; montane rainforest, ex rotten log; BLF35720 (CASC). • 1w., 1s.; Region Sofia, Bemanevika; -14.337, 48.58874; alt. 1606 m; 15 Jan 2015; B. L. Fisher et al. leg.; montane rainforest, ex rotten log; BLF35720 (CASC). • 1w., 1s.; Region Sofia, Bemanevika; -14.337, 48.58874; alt. 1606 m; 15 Jan 2015; B. L. Fisher et al. leg.; montane rainforest, ex dead tree stump; BLF35722 (CASC). • 1w., 1s.; Region Sofia, Bemanevika; -14.337, 48.58874; alt. 1606 m; 15 Jan 2015; B. L. Fisher et al. leg.; montane rainforest, ex rotten log; BLF35730 (CASC). • 1w.; Region Sofia, Bemanevika; -14.337, 48.58874; alt. 1606 m; 15 Jan 2015; B. L. Fisher et al. leg.; montane rainforest, on low vegetation; BLF35733 (CASC). • 1w., 1m.; Region Sofia, Bemanevika; -14.33886, 48.58729; alt. 1567 m; 16 Jan 2015; B. L. Fisher et al. leg.; montane rainforest, ex rotten log; BLF35750 (CASC). • 2w., 1s., 1q.; Region Sofia, Bemanevika; -14.33886, 48.58729; alt. 1567 m; 16 Jan 2015; B. L. Fisher et al. leg.; montane rainforest, ex rotten log; BLF35750 (CASC). • 2w., 1s., 1m.; Region Sofia, Bemanevika; -14.34517, 48.58948; alt. 1547 m; 16 Jan 2015; B. L. Fisher et al. leg.; montane rainforest, ex rotten log; BLF35768 (CASC). • 2w., 1s., 1m.; Region Sofia, Bemanevika; -14.34517, 48.58948; alt. 1547 m; 16 Jan 2015; B. L. Fisher et al. leg.; montane rainforest, ex rotten log; BLF35769 (CASC). • 1w.; Region Sofia, Bemanevika; -14.34517, 48.58948; alt. 1547 m; 16 Jan 2015; B. L. Fisher et al. leg.; montane rainforest, ex rotten log; BLF35779 (CASC). • 15w., 5s.; Region Sofia, Bemanevika; -14.32826, 48.58406; alt. 1657 m; 17 Jan 2015; B. L. Fisher et al. leg.; montane rainforest; sifted litter; BLF35784 (CASC). • 1w., 1q.; Region Sofia, Bemanevika; -14.32826, 48.58406; alt. 1657 m; 17 Jan 2015; B. L. Fisher et al. leg.; montane rainforest, ex rotten log; BLF35789 (CASC). • 1w., 1s.; Region Sofia, Bemanevika; -14.32826, 48.58406; alt. 1657 m; 17 Jan 2015; B. L. Fisher et al. leg.; montane rainforest, ex rotten log; BLF35794 (CASC). • 1w.; Region Sofia, Bemanevika; -14.32826, 48.58406; alt. 1657 m; 17 Jan 2015; B. L. Fisher et al. leg.; montane rainforest, ex rotten log; BLF35796 (CASC). • 1w., 1s.; Region Sofia, Bemanevika; -14.32826, 48.58406; alt. 1657 m; 17 Jan 2015; B. L. Fisher et al. leg.; montane rainforest, ex rotten log; BLF35797 (CASC). • 1w., 1s.; Region Sofia, Bemanevika; -14.32826, 48.58406; alt. 1657 m; 19 Jan 2015; B. L. Fisher et al. leg.; montane rainforest, ex rotten log; BLF35798 (CASC). • 1w., 1s.; Region Sofia, Bemanevika; -14.32826, 48.58406; alt. 1657 m; 19 Jan 2015; B. L. Fisher et al. leg.; montane rainforest, ex rotten log; BLF35798 (CASC). • 1w., 1s.; Region Sofia, Bemanevika; -14.32826, 48.58406; alt. 1657 m; 19 Jan 2015; B. L. Fisher et al. leg.; montane rainforest, ex rotten log; BLF35800 (CASC). • 1w., 1s.; Region Sofia, Bemanevika; -14.32826, 48.58406; alt. 1657 m; 19 Jan 2015; B. L. Fisher et al. leg.; montane rainforest, ex rotten log; BLF35805 (CASC). • 1w., 1s.; Region Sofia, Bemanevika; -14.32826, 48.58406; alt. 1657 m; 19 Jan 2015; B. L. Fisher et al. leg.; montane rainforest, ex rotten log; BLF35806 (CASC). • 1w., 1s.; Region Sofia, Bemanevika; -14.32826, 48.58406; alt. 1657 m; 19 Jan 2015; B. L. Fisher et al. leg.; montane rainforest, ex rotten stick on ground; BLF35811 (CASC). • 2w., 1s., 1q.; Region Sofia, Bemanevika; -14.32826, 48.58406; alt. 1657 m; 19 Jan 2015; B. L. Fisher et al. leg.; montane rainforest, ex rotten log; BLF35818 (CASC). • 1w., 1s.; Region Sofia, Bemanevika; -14.32826, 48.58406; alt. 1657 m; 19 Jan 2015; B. L. Fisher et al. leg.; montane rainforest, ex rotten log; BLF35824 (CASC). • 1w., 1s.; Region Sofia, Bemanevika; -14.32826, 48.58406; alt. 1657 m; 19 Jan 2015; B. L. Fisher et al. leg.; montane rainforest, ex rotten log; BLF35830 (CASC). • 2w., 1s., 1m.; Region Sofia, Bemanevika; -14.32826, 48.58406; alt. 1657 m; 19 Jan 2015; B. L. Fisher et al. leg.; montane rainforest, ex rotten log; BLF35830 (CASC). • 1w., 1s.; Region Sofia, Bemanevika; -14.32826, 48.58406; alt. 1657 m; 19 Jan 2015; B. L. Fisher et al. leg.; montane rainforest, ex rotten log; BLF35831 (CASC). • 1w., 1s.; Region Sofia, Bemanevika; -14.32826, 48.58406; alt. 1657 m; 19 Jan 2015; B. L. Fisher et al. leg.; montane rainforest, ex rotten log; BLF35833 (CASC). • 1w., 1s., 1m.; Region Sofia, Bemanevika; -14.32826, 48.58406; alt. 1657 m; 19 Jan 2015; B. L. Fisher et al. leg.; montane rainforest, ex rotten log; BLF35834 (CASC). • 2w., 1s., 1m.; Region Sofia, Bemanevika; -14.32826, 48.58406; alt. 1657 m; 19 Jan 2015; B. L. Fisher et al. leg.; montane rainforest, ex rotten log; BLF35835 (CASC). • 2w., 1s., 1m.; Region Sofia, Bemanevika; -14.32826, 48.58406; alt. 1657 m; 19 Jan 2015; B. L. Fisher et al. leg.; montane rainforest, ex rotten log; BLF35836 (CASC). • 2w., 1s., 1q.; Region Sofia, Bemanevika; -14.32826, 48.58406; alt. 1657 m; 19 Jan 2015; B. L. Fisher et al. leg.; montane rainforest, ex rotten log; BLF35837 (CASC). • 2w., 1s., 1q.; Region Sofia, Bemanevika; -14.32826, 48.58406; alt. 1657 m; 19 Jan 2015; B. L. Fisher et al. leg.; montane rainforest, ex rotten log; BLF35838 (CASC). • 2w., 1s., 1m.; Region Sofia, Bemanevika; -14.32826, 48.58406; alt. 1657 m; 19 Jan 2015; B. L. Fisher et al. leg.; montane rainforest, ex rotten log; BLF35839 (CASC). • 2w., 1s., 1m.; Region Sofia, Bemanevika; -14.32826, 48.58406; alt. 1657 m; 19 Jan 2015; B. L. Fisher et al. leg.; montane rainforest, ex rotten log; BLF35842 (CASC).

##### Description.

**Major workers.** Measurements (*N* = 10): HL: 1.34–1.5 (1.39); HW: 1.26–1.45 (1.36); SL: 0.86–0.95 (0.89); EL: 0.18–0.21 (0.19); WL: 1.14–1.3 (1.21); PSL: 0.17–0.22 (0.21); MTL: 0.82–0.91 (0.86); PNW: 0.5–0.56 (0.52); PTW: 0.15–0.19 (0.17); PPW: 0.3–0.41 (0.35); CI: 99.9–107.5 (103.0); SI: 61.7–68.2 (65.6); PSLI: 12.8–16.4 (14.8); PPI: 42.2–57.2 (48.6); PNI: 35.2–41.8 (38.6); MTI: 61.4–67.4 (63.8).

***Head.*** In full-face view sub-oval, slightly widening posteriorly, with anterior and posterior sides convex (Fig. 57B). In lateral view sub-oval; ventral and dorsal faces convex; inner hypostomal teeth visible. Sides of the head with very dense, long, suberect to erect pilosity; whole head with dense, long, decumbent to erect pilosity. Medial part of frons with thick, interrupted, dense, and longitudinally irregular rugae with indistinctly to distinctly rugulate interspaces; lateral sides of frons with denser, thick, and irregular rugae, interspaces with dense and distinct rugulae. Occipital lobes with irregular and sparser rugae and indistinctly rugulate to smooth interspaces. Area posterolateral from eyes with dense, thick, longitudinal rugae with distinctly rugulate interspaces, sculpture fading posteriorly. Gena with relatively sparse, thick, and longitudinal rugae and indistinctly rugulate interspaces. Centre of clypeus smooth and shiny, lateral sides with indistinct rugulae; median notch present, moderately wide, and shallow; median longitudinal carina present; lateral longitudinal carinae absent. Scape, when laid back, exceeding the midlength of head by one-fifth of its length; pilosity subdecumbent to erect (Fig. 57B, D). Inner hypostomal teeth distinct, large, and wide, closely spaced, triangular with apex directed slightly inward; outer hypostomal teeth lobe-like, narrower than and approximately as high as inner teeth, apex directed upward; inner and outer hypostomal teeth closely spaced and not connected by concavity (Fig. 64O). ***Mesosoma.*** In lateral view, promesonotum short, angular, and moderately low, posterior mesonotum moderately steep, mesonotal process indistinct, tubercle-like; promesonotal groove absent; metanotal groove present; propodeal spines moderately long, relatively wide, and with acute apex; humeral area weakly produced (Fig. 57D). Surface shiny and rugofoveolate; katepisternum with reduced sculpture and sometimes with smooth notch. Pilosity moderately dense, long, and erect (Fig. 57D, F). ***Petiole.*** Shiny with sparse foveolae; node finely foveolate to smooth, triangular, with rounded and thick apex, in rear view node dorsoventrally slightly convex; pilosity moderately sparse and erect (Fig. 57D, F). ***Postpetiole.*** Shiny and foveolate; dorsum with reduced sculpture; in dorsal view oval, lateral margins medially with two small and dentate projections; pilosity long, moderately sparse, and erect (Fig. 57D, F). ***Gaster.*** Shiny with slightly shagreened base; pilosity moderately dense, long, and erect (Fig. 57D, F). ***Colour.*** Yellow to blackish brown, antenna and legs yellowish (Fig. 57D, F).

**Minor workers.** Measurements (*N* = 10): HL: 0.67–0.76 (0.72); HW: 0.55–0.61 (0.57); SL: 0.83–0.92 (0.88); EL: 0.13–0.16 (0.14); WL: 0.89–1.01 (0.95); PSL: 0.1–0.14 (0.12); MTL: 0.67–0.77 (0.72); PNW: 0.38–0.42 (0.4); PTW: 0.06–0.11 (0.09); PPW: 0.17–0.18 (0.17); CI: 121.8–133.6 (125.9); SI: 144.0–158.5 (152.9); PSLI: 14.2–18.9 (16.7); PPI: 35.5–60.3 (53.5); PNI: 67.7–72.5 (70.1); MTI: 117.7–134.7 (125.0).

***Head.*** Cephalic margin slightly convex (Fig. 57A). Pilosity relatively sparse, short, subdecumbent. Sculpture shiny and foveolate; median frons with short, indistinct and irregular rugulae; antennal sockets with few indistinct, curved outward rugae and foveolate interspaces. Clypeus foveolate; with median longitudinal carina absent; two lateral longitudinal carinae absent. Scape, when laid back, exceeding the posterior head margin by two-fifths of its length; pilosity dense, subdecumbent to erect (Fig. 57A, C). ***Mesosoma.*** In lateral view, promesonotum low and moderately long, arched; promesonotal groove indistinct; metanotal groove distinct; propodeal spines minute and triangular (Fig. 57C). Sculpture shiny and foveolate; katepisternum with sparser foveolae. Pilosity sparse, moderately long, and erect (Fig. 57C, E). ***Gaster.*** With sparse, erect pilosity (Fig. 57C, E). ***Colour.*** Yellow to black, legs and antenna yellowish (Fig. 57C, E).

##### Etymology.

Greek for hairy in reference to dense setosity on lateral sides of head in major workers.

##### Biology.

The species was collected between 74–1657 m in elevation, in montane rainforest and in gardens. Nests were located in rotten logs, rotten tree stumps, and dead twigs above ground.

##### Comments.

*Pheidole trichotos* sp. nov. can be grouped with species characterised by dark body colouration, ranging in major workers from brownish black to black and in minor workers from black to dark brown, with body entirely foveolate. The group consists of three sympatric species: *P. alina* sp. nov., *P. trichotos* sp. nov., and *P. mainty* sp. nov. *Pheidole alina* sp. nov. and *P. mainty* sp. nov. are known from the northernmost part of the island. Major workers of *P. trichotos* sp. nov. differ from *P. alina* sp. nov. and *P. mainty* sp. nov. in medial part of frons with thick, interrupted, dense, and longitudinally irregular rugae with indistinctly to distinctly rugulate interspaces and shagreened base of first gastral tergite. Minor workers of *P. trichotos* sp. nov. differ from *P. alina* sp. nov. in smaller body size and brighter body colouration; from *P. mainty* sp. nov. in entirely foveolate head and mesosoma. Majors of *P. trichotos* sp. nov. can be also confused with major workers of parapatric *P. veteratrix* and sympatric *P. anomala* sp. nov. *P. trichotos* sp. nov. can be separated from both of those taxa based on shagreened base of first gastral tergite and darker body colouration.

#### Pheidole tsaravoniana sp. nov.

Taxon classificationAnimalia

6504D13F-EC3C-56C5-9184-6D9C2A22C3E1

http://zoobank.org/0D3B9BA6-A8F9-4E95-AB88-02FC7F2AC187

**Figure 58.**
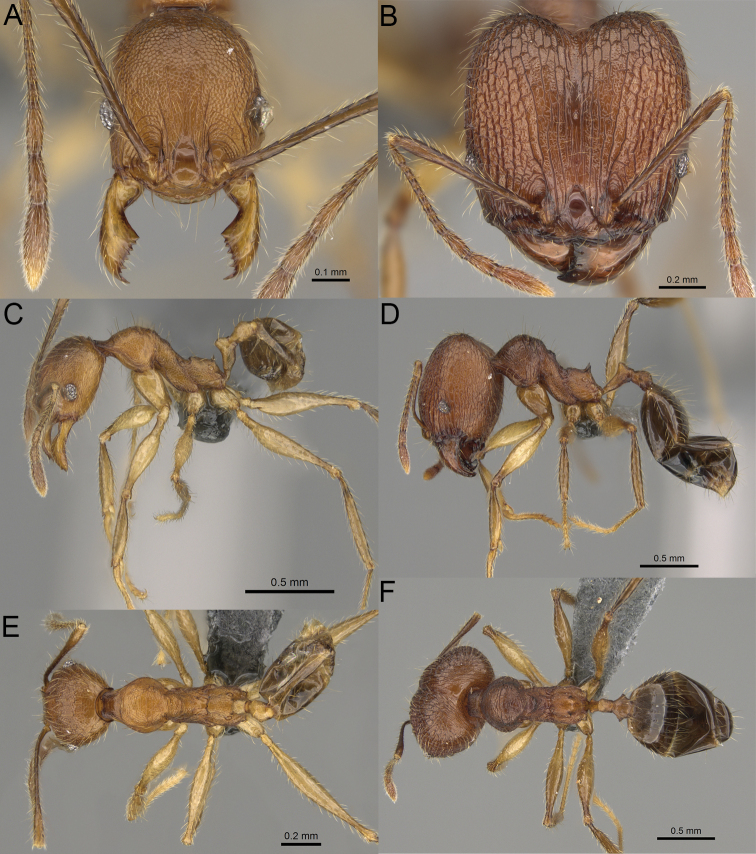
*Pheidole tsaravoniana* sp. nov., full-face view (**A**), profile (**C**), and dorsal view (**E**) of paratype minor worker (CASENT0923286) and full-face view (**B**), profile (**D**), and dorsal view (**F**) of holotype major worker (CASENT0300007).

Figs 58A–F , 64P , 67H

##### Type material.

***Holotype.*** Madagascar. • 1 major worker; Toamasina; Corridor Forestier Analamay-Mantadia, Tsaravoniana; -18.75641, 48.42195; alt. 1036 m; 7 Dec 2012; B. L. Fisher et al. leg.; rainforest, ex rotten log; BLF30162; CASENT0300007 (CASC). ***Paratype.*** • 1w.; same data as for holotype, CASENT0923286 (CASC).

##### Diagnosis.

Moderately large species. ***Major workers.*** Head in full-face view sub-oval, not widening posteriorly, with anterior and posterior sides slightly convex, in lateral view sub-oval; ventral and dorsal faces convex; sides of the head with moderately dense, moderately long, suberect pilosity; medial part of frons with thick, interrupted, dense, and longitudinal rugae with distinctly rugulate interspaces; lateral sides of frons with dense, thick, and longitudinally irregular rugae, interspaces with dense rugulae; occipital lobes and area posterolateral from eyes without smooth notches; scape, when laid back, exceeding the midlength of head by two-fifths of its length; inner hypostomal teeth distinct, moderately large, closely spaced, triangular, with rounded apex directed inward; outer hypostomal teeth lobe-like, wider than and approximately as high as inner teeth, apex directed upward; inner and outer hypostomal teeth closely spaced and connected by indistinct concavity; mesosoma rugofoveolate; pronotum with additional thin, moderately dense, and transverse rugae; gaster smooth with base indistinctly shagreened; body ferruginous. ***Minor workers.*** Head foveolate, foveolae sparse; scape, when laid back, exceeding the posterior head margin by one-third of its length; promesonotum low and moderately long; promesonotal groove absent; propodeal spines minute and triangular; mesosoma foveolate; katepisternum with large smooth notch; dorsal side of promesonotum and lateral sides of pronotum with reduced sculpture and smooth notches on medial parts; body yellowish brown.

##### Description.

**Major workers.** Measurements (*N* = 1): HL: 1.27; HW: 1.0; SL: 0.9; EL: 0.17; WL: 1.21; PSL: 0.18; MTL: 0.86; PNW: 0.5; PTW: 0.14; PPW: 0.3; CI: 126.7; SI: 89.4; PSLI: 14.4; PPI: 47.5; PNI: 49.8; MTI: 86.2.

***Head.*** In full-face sub-oval, not widening posteriorly, with anterior and posterior sides slightly convex (Fig. 58B). In lateral view sub-oval; ventral and dorsal faces convex; inner hypostomal teeth visible. Sides of the head with moderately dense, moderately long, suberect pilosity; whole head with dense, long, decumbent to erect pilosity. Medial part of frons with thick, interrupted, dense, and longitudinal rugae with distinctly rugulate interspaces; lateral sides of frons with dense, thick, and longitudinally irregular rugae, interspaces with dense rugulae. Occipital lobes with irregular and thinner rugae and indistinctly rugulate interspaces. Area posterolateral from eyes with sparse, thick, irregular to longitudinal rugae with distinctly rugulate interspaces. Gena with relatively sparse, thick, and longitudinal rugae and distinctly rugulate interspaces. Centre of clypeus smooth and shiny, lateral sides with indistinct rugulae; median notch present, moderately wide, and shallow; median longitudinal carina present; lateral longitudinal carinae absent. Scape, when laid back, exceeding the midlength of head by two-fifths of its length; pilosity subdecumbent to erect (Fig. 58B, D). Inner hypostomal teeth distinct, moderately large, closely spaced, and triangular, with rounded apex directed inward; outer hypostomal teeth lobe-like, wider than and approximately as high as inner teeth, apex directed upward; inner and outer hypostomal teeth closely spaced and connected by indistinct concavity (Fig. 64P). ***Mesosoma.*** In lateral view, promesonotum short, angular, and moderately low, posterior mesonotum moderately steep, mesonotal process indistinct and tubercle-like; promesonotal groove absent; metanotal groove absent; propodeal spines moderate, with wide base and acute apex; humeral area weakly produced (Fig. 58D). Surface shiny and rugofoveolate; pronotum with additional thin, moderately dense, and transverse rugae. Pilosity moderately dense, long, and erect (Fig. 58D, F). ***Petiole.*** Shiny with dense foveolae; node finely foveolate, triangular, with rounded and thick apex, in rear view node dorsoventrally slightly convex; pilosity moderately sparse and erect (Fig. 58D, F). ***Postpetiole.*** Shiny and foveolate; dorsum with reduced sculpture and smooth notch; in dorsal view oval, lateral margins medially with two dentate projections; pilosity long, moderately sparse, and erect (Fig. 58D, F). ***Gaster.*** Shiny and smooth with slightly shagreened base; pilosity moderately dense, long, and erect (Fig. 58D, F). ***Colour.*** Ferruginous, gaster slightly darker, legs yellowish (Fig. 58D, F).

**Minor workers.** Measurements (*N* = 1): HL: 0.66; HW: 0.52; SL: 0.81; EL: 0.12; WL: 0.88; PSL: 0.08; MTL: 0.69; PNW: 0.37; PTW: 0.1; PPW: 0.14; CI: 128.9; SI: 157.5; PSLI: 11.7; PPI: 72.2; PNI: 71.8; MTI: 133.6.

***Head.*** Cephalic margin slightly convex (Fig. 58A). Pilosity relatively sparse, moderately long, subdecumbent to erect. Sculpture shiny and foveolate; median frons with short and indistinct longitudinal rugulae; area posterolateral from eyes with weaker sculpture; antennal sockets with few indistinct, curved outward rugae and foveolate interspaces. Clypeus with median longitudinal carina absent; two lateral longitudinal carinae absent. Scape, when laid back, exceeding the posterior head margin by one-third of its length; pilosity dense, subdecumbent to erect (Fig. 58A, C). ***Mesosoma.*** In lateral view, promesonotum low and moderately long, arched; promesonotal groove indistinct; metanotal groove distinct; propodeal spines minute and triangular (Fig. 58C). Sculpture shiny and foveolate. Pilosity very sparse, moderately long, and erect (Fig. 58C, E). ***Gaster.*** With sparse, erect pilosity (Fig. 58C, E). ***Colour.*** Brown, legs yellowish (Fig. 58C, E).

##### Etymology.

From the type locality.

##### Biology.

The species was collected at 1036 m in elevation, in rainforest. Nest was located in rotten log.

##### Comments.

*Pheidole tsaravoniana* sp. nov. is described from the vicinity of Tsaravoniana in Toamasina and is parapatric with *P. renirano* sp. nov. from several localities distributed in the Toamasina prefecture (from Réserve Spéciale Ambatovaky north to Antanambe). Both taxa have very similar morphology. Major workers of *P. tsaravoniana* can be distinguished from majors of *P. renirano* sp. nov. based on ferruginous body colouration and promesonotal dorsum with transverse and more irregular rugae with distinctly rugofoveolae interspaces; minors can be separated based on brown body, never reduced sculpture, and lack of smooth notches on medial parts of dorsal side of promesonotum and lateral sides of pronotum.

#### Pheidole vadum sp. nov.

Taxon classificationAnimalia

88D168D0-245B-5B6D-98C1-71AA188EEE32

http://zoobank.org/F85769B8-4A08-4652-8686-CFD9B7F3A620

**Figure 59.**
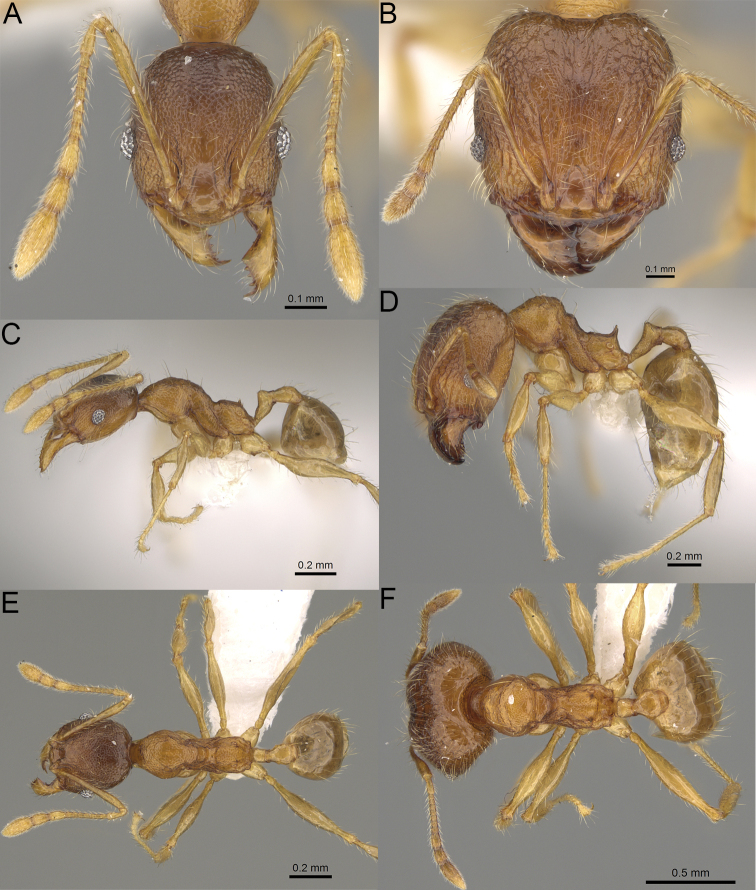
*Pheidole vadum* sp. nov., full-face view (**A**), profile (**C**), and dorsal view (**E**) of paratype minor worker (CASENT0427785) and full-face view (**B**), profile (**D**), and dorsal view (**F**) of holotype major worker (CASENT0427789).

Figs 59A–F , 64Q , 67I

##### Type material.

***Holotype.*** Madagascar. • 1 major worker; Antananarivo; 3 km 41°NE Andranomay, 11.5 km 147°SSE Anjozorobe; -18.47333, 47.96; alt. 1300 m; 5 Dec 2000; Fisher et al. leg.; montane rainforest, ex dead twig above ground; BLF02436; CASENT0427789 (CASC). ***Paratypes.*** • 4w., 1s.; same data as for holotype; CASENT0427788, CASENT0427791, CASENT0427785 (CASC, MHNG).

##### Diagnosis.

Moderately large species. ***Major workers.*** Head in full-face view oval, not widening posteriorly, with anterior and posterior sides convex, in lateral view sub-oval, ventral and dorsal faces convex, occipital cleft very shallow; sides of the head with dense, moderately long, suberect pilosity; medial frons with moderately dense, thin, longitudinal to irregular and interrupted rugae, rugae in posteromedial part slightly directed outward, interspaces shiny with sparse and distinct rugofoveolae; occipital lobes and area posterolateral from eyes never smooth; scape, when laid back, exceeding the midlength of head by two-fifths of its length; inner hypostomal teeth distinct, large, closely spaced, triangular, with rounded apex directed upward; outer hypostomal teeth lobe-like, wider than inner hypostomal teeth and approximately the same height, apex directed outward; inner and outer hypostomal teeth closely spaced and not connected by concavity; mesosoma with thick and sparse foveolae; lateral sides of pronotum and propodeum with smooth notches; body yellowish brown. ***Minor workers.*** Head foveolate; anteromedial part of frons with smooth notch; area posterolateral from eyes predominantly smooth; scape, when laid back, surpassing the posterior head margin by one-fifth of its length; promesonotum moderately high and short; promesonotal groove absent; propodeal spines minute and triangular; mesosoma with thick foveolae; body yellowish brown.

##### Description.

**Major workers.** Measurements (*N* = 2): HL: 0.96, 1.04; HW: 0.92, 1.01; SL: 0.64, 0.63; EL: 0.12, 0.13; WL: 0.94, 1.0; PSL: 0.17, 0.18; MTL: 0.61, 0.62; PNW: 0.38, 0.44; PTW: 0.13, 0.14; PPW: 0.23, 0.25; CI: 103.5, 103.0; SI: 69.7, 62.4; PSLI: 17.7, 17.4; PPI: 57.8, 53.6; PNI: 41.0, 43.4; MTI: 66.0, 61.2.

***Head.*** In full-face view oval, not widening posteriorly, with anterior and posterior sides convex (Fig. 59B); occipital cleft very shallow. In lateral view sub-oval; ventral and dorsal faces convex; inner hypostomal teeth visible. Sides of the head with dense, moderately long, suberect pilosity; whole head with dense, long, decumbent to erect pilosity. Medial part of frons with moderately dense, thin, longitudinal to irregular and interrupted rugae, rugae in posteromedial part slightly directed outward, interspaces shiny with sparse and distinct rugofoveolae; anterolateral sides with longitudinal, thin, and relatively dense rugae; posterolateral sides with irregular, thin, and relatively dense rugae; interspaces shiny with relatively dense and distinct rugofoveolae. Occipital lobes with sparse, thick, and irregular rugae; interspaces rugofoveolate. Gena with relatively sparse, thick, and longitudinal rugae and rugofoveolate interspaces. Area posterolateral from eyes shiny, with dense rugofoveolae. Centre of clypeus smooth and shiny, lateral sides with indistinct rugulae; median notch present, moderately wide, and shallow; median longitudinal carina absent; lateral longitudinal carinae absent. Scape, when laid back, exceeding the midlength of head by two-fifths of its length; pilosity subdecumbent to erect (Fig. 59B, D). Inner hypostomal teeth distinct, large, closely spaced, triangular, with rounded apex directed upward; outer hypostomal teeth lobe-like, wider than inner hypostomal teeth and approximately the same height, apex directed outward; inner and outer hypostomal teeth closely spaced and not connected by concavity (Fig. 64Q). ***Mesosoma.*** In lateral view, promesonotum short, angular, and moderately low, posterior mesonotum moderately steep, mesonotal process indistinct, tubercle-like; promesonotal groove absent; metanotal groove absent; propodeal spines moderately long, moderately narrow, with acute apex; humeral area laterally weakly produced (Fig. 59D). Surface shiny with thick and sparse foveolae; lateral sides of pronotum and propodeum with smooth notches. Pilosity sparse, long, and erect (Fig. 59D, F). ***Petiole.*** Shiny with fine and sparse foveolae; node smooth, low, triangular, with rounded and thin apex, in rear view node dorsoventrally slightly concave; pilosity moderately sparse and erect (Fig. 59D, F). ***Postpetiole.*** Shiny and smooth; in dorsal view oval, lateral margins medially with two dentate projections; pilosity long, moderately sparse, and erect (Fig. 59D, F). ***Gaster.*** Shiny and smooth; pilosity moderately dense, long, and erect (Fig. 59D, F). ***Colour.*** Yellowish brown; mandibles and gaster slightly darker; legs yellowish (Fig. 59D, F).

**Minor workers.** Measurements (*N* = 4): HL: 0.52–0.59 (0.56); HW: 0.46–0.51 (0.49); SL: 0.55–0.59 (0.56); EL: 0.1–0.11 (0.11); WL: 0.72–0.73 (0.73); PSL: 0.07–0.08 (0.08); MTL: 0.47–0.51 (0.49); PNW: 0.3–0.33 (0.31); PTW: 0.07–0.09 (0.08); PPW: 0.13–0.14 (0.13); CI: 112.7–116.7 (114.1); SI: 111.3–117.9 (114.9); PSLI: 11.3–15.9 (13.6); PPI: 53.0–62.8 (59.3); PNI: 57.6–65.0 (62.2); MTI: 98.8–101.2 (100.5).

***Head.*** Cephalic margin indistinctly convex or straight (Fig. 59A). Pilosity relatively sparse, long, decumbent to suberect. Sculpture foveolate; anteromedial part of frons with smooth notch; area posterolateral from eyes predominantly smooth. Clypeus with median longitudinal carina absent; two lateral longitudinal carinae absent. Scape, when laid back, surpassing the posterior head margin by one-fifth of its length; pilosity dense, subdecumbent to erect (Fig. 59A, C). ***Mesosoma.*** In lateral view, promesonotum moderately high and short, arched; promesonotal groove absent; metanotal groove distinct; propodeal spines minute, triangular (Fig. 59C). Sculpture shiny and with thick foveolae. Pilosity very sparse, moderately long, and erect (Fig. 59C, E). ***Postpetiole.*** Short, low, and relatively flat; with few short, erect setae (Fig. 59C, E). ***Gaster.*** With sparse, erect pilosity (Fig. 59C, E). ***Colour.*** Yellowish brown, vertex slightly darker (Fig. 59C, E).

##### Etymology.

Latin for shallow in reference to very shallow occipital cleft.

##### Biology.

The species was collected at 1300 m in elevation, in montane rainforest. Nest was located in a dead twig above ground.

##### Comments.

*Pheidole vadum* sp. nov. is a member of the group of species characterised by major workers with head in full-face view oval and not widening posteriorly and medial part of frons with thick, moderately sparse, irregular rugae or medial frons with moderately dense, thin, longitudinal anteriorly to irregular posteriorly, interrupted rugae and very shallow occipital cleft. Minor workers of this group have a short and moderately high promesonotum and dark body colouration ranging from orange to brown. The group consists of three species: *P. vadum* sp. nov., *P. analavelona* sp. nov., and *P. ambohimanga* sp. nov. *Pheidole vadum* sp. nov. is known from the vicinity of Antananarivo and is sympatric with *P. ambohimanga* sp. nov. Its majors can be easily separated from *P. ambohimanga* sp. nov. based on presence of moderately dense, thin, longitudinal anteriorly to irregular posteriorly, interrupted rugae on medial frons and very shallow occipital cleft; minor workers can be separated based on predominantly foveolate head sculpture lacking additional rugae. Additionally, minors of *P. vadum* sp. nov. can be confused with workers of other members of the *sikorae* group, especially with workers of *P. sava* sp. nov. and *P. sparsa* sp. nov., both known from the northern part of the island. However, minor workers of *P. vadum* sp. nov. can be separated based on the combination of the following characters: body yellowish brown, head and mesosoma predominantly foveolate with no additional sculpture, scape, when laid back, surpassing the posterior head margin by one-fifth of its length, promesonotum short and moderately high, and promesonotal groove absent.

#### Pheidole veteratrix

Taxon classificationAnimalia

Forel, 1891

34006D0F-7B85-5F59-AECF-C4BFCA686C0F

**Figure 60.**
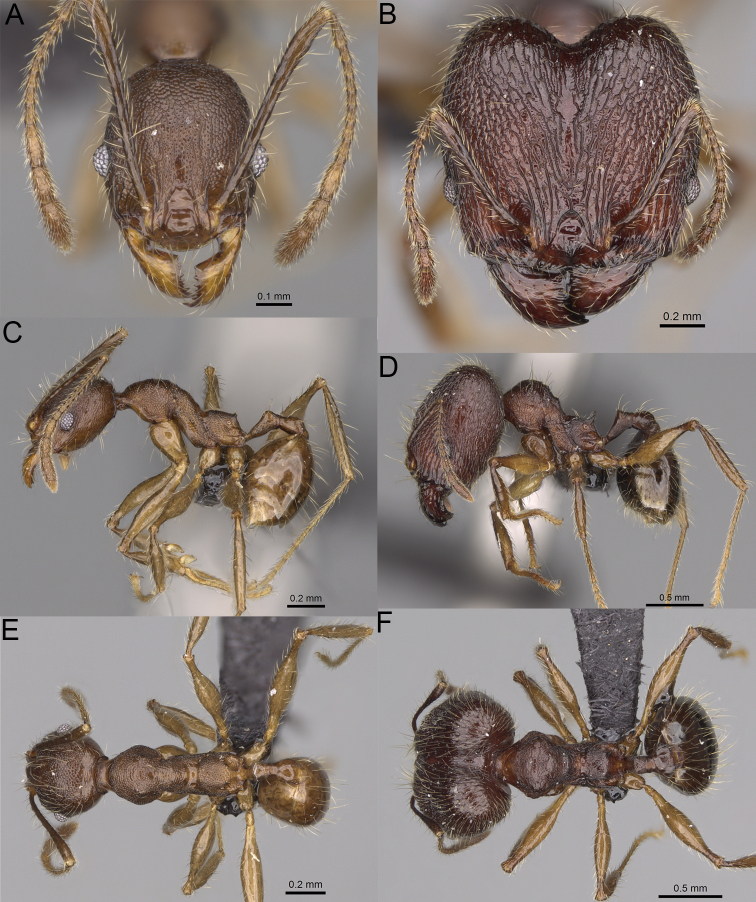
*Pheidole veteratrix* Forel, full-face view (**A**), profile (**C**), and dorsal view (**E**) of minor worker (CASENT0923259) and full-face view (**B**), profile (**D**), and dorsal view (**F**) of major worker (CASENT0148662).

Figs 60A–F , 64R , 67J

Pheidole veteratrix Forel, 1891: 225 (s.w.).
=Pheidole veteratrix angustinoda Forel, 1892: 526 (s.w.). **syn. nov.**

##### Type material.

*Pheidole veteratrix* Forel, 1891: 225 (s.w.).

Lectotype [designated here]: major worker (CASENT0101594): Madagascar, Antananarivo, Forêt d’Andrangoloaka, coll. Sikora (MHNG) [examined]. Paralectotypes: 1 minor worker (CASENT0101630) (MHNG) [examined], 1 minor worker (CASENT0923190) (MHNG) [examined], 1 major worker (CASENT0101935) (MHNG) [examined], 1 worker (CASENT0923191) (MHNG) [examined].

Lectotype [designated here]: major worker, bottom specimen on the pin (CASENT0101567): Madagascar, Toamasina, Amparafaravantsiv, Mangoro river, coll. Sikora; (MHNG) [examined]. Paralectotypes: 1 major worker, top specimen, the same pin as holotype (CASENT0876546) (MHNG) [examined], 2 minor workers (CASENT0101661) (MHNG) [examined].

##### Other material.

Madagascar. –**Antananarivo**: • 4w., 6s.; 3 km 41°NE Andranomay, 11.5 km 147°SSE Anjozorobe; -18.47333, 47.96; alt. 1300 m; 5 Dec 2000; B. L. Fisher et al. leg.; montane rainforest, ex rotten log; BLF02393 (CASC). • 1w.; 3 km 41°NE Andranomay, 11.5 km 147°SSE Anjozorobe; -18.47333, 47.96; alt. 1300 m; 5 Dec 2000; B. L. Fisher et al. leg.; montane rainforest, ex rotten log; BLF02395 (CASC). • 2s.; 3 km 41°NE Andranomay, 11.5 km 147°SSE Anjozorobe; -18.47333, 47.96; alt. 1300 m; 5 Dec 2000; B. L. Fisher et al. leg.; montane rainforest, ex rotten log; BLF02396 (CASC). • 1w., 3s.; 3 km 41°NE Andranomay, 11.5 km 147°SSE Anjozorobe; -18.47333, 47.96; alt. 1300 m; 5 Dec 2000; B. L. Fisher et al. leg.; montane rainforest, ex rotten log; BLF02401 (CASC). • 3w., 1s.; 3 km 41°NE Andranomay, 11.5 km 147°SSE Anjozorobe; -18.47333, 47.96; alt. 1300 m; 5 Dec 2000; B. L. Fisher et al. leg.; montane rainforest, under stone; BLF02417 (CASC). • 1w., 1s.; Réserve Naturelle Sohisika, Sohisika 24.6 km NNE Ankazobe; -18.10322, 47.18692; alt. 1464 m; 1 Jun 2008; B. L. Fisher et al. leg.; gallery montane forest, sifted litter (leaf mold, rotten wood); BLF20507 (CASC). • 1w., 1s.; Réserve Naturelle Sohisika, Sohisika 24.6 km NNE Ankazobe; -18.10322, 47.18692; alt. 1464 m; 1 Jun 2008; B. L. Fisher et al. leg.; gallery montane forest, ex rotten log; BLF20539 (CASC). • 1w., 1s.; Réserve Speciale d’Ambohitantely; -18.18762, 47.28576; alt. 1580 m; 8 Mar 2012; B. L. Fisher et al. leg.; montane forest, ex rotten log; BLF28217 (CASC). • 1w., 1s.; Réserve Speciale d’Ambohitantely; -18.22444, 47.2774; alt. 1490 m; 9 Mar 2012; B. L. Fisher et al. leg.; montane forest, ex rotten log; BLF28244 (CASC). • 1w., 1s.; Réserve Speciale d’Ambohitantely; -18.22444, 47.2774; alt. 1490 m; 9 Mar 2012; B. L. Fisher et al. leg.; montane forest, ex rotten log; BLF28268 (CASC). • 2w., 1s., 1q.; Réserve Speciale d’Ambohitantely; -18.22444, 47.2774; alt. 1490 m; 9 Mar 2012; B. L. Fisher et al. leg.; montane forest, ex rotten log; BLF28286 (CASC). • 1w., 1s.; Réserve Speciale d’Ambohitantely; -18.22444, 47.2774; alt. 1490 m; 9 Mar 2012; B. L. Fisher et al. leg.; montane forest, ex rotten log; BLF28296 (CASC). –**Fianarantsoa**: • 1w.; 29 km SSW Ambositra, Ankazomivady; -20.77667, 47.165; alt. 1700 m; 7 Jan 1998; B. L. Fisher et al. leg.; disturbed montane rainforest; BLF01593 (CASC). • 1w.; 29 km SSW Ambositra, Ankazomivady; -20.77667, 47.165; alt. 1700 m; 7 Jan 1998; B. L. Fisher et al. leg.; disturbed montane rainforest; BLF01594 (CASC). • 1w.; 38 km S Ambalavao, Rés. Andringitra; -22.2, 46.96667; alt. 1680 m; 22 Oct 1993; B. L. Fisher et al. leg.; montane rainforest; BLF00818 (CASC). • 1w., 1s., 1q.; 38 km S Ambalavao, Rés. Andringitra; -22.2, 46.96667; alt. 1680 m; 24 Oct 1993; B. L. Fisher et al. leg.; montane rainforest, ex rotten log; BLF00823 (CASC). •1w.; R.S. Ivohibe, 6.5 km ESE Ivohibe; -22.49667, 46.955; alt. 1575 m; 24 Oct 1997; B. L. Fisher et al. leg.; montane rainforest, sifted litter (leaf mold, rotten wood); BLF01751 (CASC). • 3w.; R.S. Ivohibe, 6.5 km ESE Ivohibe; -22.49667, 46.955; alt. 1575 m; 24 Oct 1997; B. L. Fisher et al. leg.; montane rainforest; BLF01752 (CASC). –**Toamasina**: • 1w., 1s., 1q.; 6.9 km NE Ambanizana, Ambohitsitondroina; -15.58506, 50.00952; alt. 825 m; 2 Dec 1993; B. L. Fisher et al. leg.; rainforest, sifted litter (leaf mold, rotten wood); BLF00976 (CASC). •1w., 1s.; F.C. Didy; -18.19833, 48.57833; alt. 960 m; 16 Dec 1998; H.J. Ratsirarson leg.; rainforest, ex rotten log; HJR095 (CASC). • 1w.; Forêt Ambatovy, 14.3 km 57° Moramanga; -18.85083, 48.32; alt. 1075 m; 21 Mar 2004; Malagasy ant team leg.; montane rainforest, sifted litter (leaf mold, rotten wood); BLF10501 (CASC). –**Toliara**: • 1w.; Parc National d’Andohahela, Col du Sedro, 3.8 km 113°ESE Mahamavo, 37.6 km 341°NNW Tolagnaro; -24.76389, 46.75167; alt. 900 m; 21 Jan 2002; B. L. Fisher et al. leg.; montane rainforest, BLF05012 (CASC). • 1w.; Parc National d’Andohahela, Col du Sedro, 3.8 km 113°ESE Mahamavo, 37.6 km 341°NNW Tolagnaro; -24.76389, 46.75167; alt. 900 m; 21 Jan 2002; B. L. Fisher et al. leg.; montane rainforest; BLF05013 (CASC). • 1w., 1q.; Réserve Spéciale Kalambatritra, Ambinanitelo; -23.4502, 46.45658; alt. 1325 m; 11 Feb 2009; B. L. Fisher et al. leg.; montane rainforest, ex rotten log; BLF21796 (CASC). • 2w., 1s., 1q.; Réserve Spéciale Kalambatritra, Ambinanitelo; -23.4502, 46.45658; alt. 1325 m; 11 Feb 2009; B. L. Fisher et al. leg.; montane rainforest, ex rotten log; BLF21825 (CASC). • 1w.; Réserve Spéciale Kalambatritra, Ampanihy; -23.4635, 46.4631; alt. 1270 m; 9 Feb 2009; B. L. Fisher et al. leg.; montane rainforest, sifted litter (leaf mold, rotten wood); BLF21566 (CASC). • 1w., 1s.; Réserve Spéciale Kalambatritra, Befarara; -23.4178, 46.4478; alt. 1390 m; 7 Feb 2009; B. L. Fisher et al. leg.; montane rainforest, ex rotten log; BLF21338 (CASC). • 2w., 1s., 1q.; Réserve Spéciale Kalambatritra, Befarara; -23.4178, 46.4478; alt. 1390 m; 7 Feb 2009; B. L. Fisher et al. leg.; montane rainforest, ex rotten log; BLF21343 (CASC). • 1w., 1s.; Réserve Spéciale Kalambatritra, Befarara; -23.4178, 46.4478; alt. 1390 m; 7 Feb 2009; B. L. Fisher et al. leg.; montane rainforest, ex rotten log; BLF21348 (CASC). • 1w., 1s.; Réserve Spéciale Kalambatritra, Befarara; -23.4178, 46.4478; alt. 1390 m; 7 Feb 2009; B. L. Fisher et al. leg.; montane rainforest, ex rotten log; BLF21359 (CASC). • 1w., 1s.; Réserve Spéciale Kalambatritra, Befarara; -23.4178, 46.4478; alt. 1390 m; 7 Feb 2009; B. L. Fisher et al. leg.; montane rainforest; sifted litter (leaf mold, rotten wood); BLF21370 (CASC). • 1w., 1s.; Réserve Spéciale Kalambatritra, Befarara; -23.4178, 46.4478; alt. 1390 m; 7 Feb 2009; B. L. Fisher et al. leg.; montane rainforest, ex rotten log; BLF21374;(CASC). • 1w., 1s.; Réserve Spéciale Kalambatritra, Befarara; -23.4178, 46.4478; alt. 1390 m; 7 Feb 2009; B. L. Fisher et al. leg.; montane rainforest; ex rotten log; BLF21383 (CASC). • 1w., 1s.; Réserve Spéciale Kalambatritra, Befarara; -23.4178, 46.4478; alt. 1390 m; 7 Feb 2009; B. L. Fisher et al. leg.; montane rainforest, ex rotten log; BLF21385 (CASC). • 1w., 1s.; Réserve Spéciale Kalambatritra, Befarara; -23.4178, 46.4478; alt. 1390 m; 7 Feb 2009; B. L. Fisher et al. leg.; montane rainforest, ex rotten log; BLF21399 (CASC). • 2w., 1s., 1q.; Réserve Spéciale Kalambatritra, Betanana; -23.4144, 46.459; alt. 1360 m; 8 Feb 2009; B. L. Fisher et al. leg.; montane rainforest, ex rotten log; BLF21412 (CASC). • 1w., 1s.; Réserve Spéciale Kalambatritra, Betanana; -23.4144, 46.459; alt. 1360 m; 8 Feb 2009; B. L. Fisher et al. leg.; montane rainforest, ex rotten log; BLF21418 (CASC). • 1w., 1s.; Réserve Spéciale Kalambatritra, Betanana; -23.4144, 46.459; alt. 1360 m; 8 Feb 2009; B. L. Fisher et al. leg.; montane rainforest, ex rotten log; BLF21424 (CASC). • 1w., 1s.; Réserve Spéciale Kalambatritra, Betanana; -23.4144, 46.459; alt. 1360 m; 8 Feb 2009; B. L. Fisher et al. leg.; montane rainforest, ex rotten log; BLF21435 (CASC). • 1w., 1s.; Réserve Spéciale Kalambatritra, Betanana; -23.4144, 46.459; alt. 1360 m; 8 Feb 2009; B. L. Fisher et al. leg.; montane rainforest, ex rotten log; BLF21435 (CASC). • 1w., 1s.; Réserve Spéciale Kalambatritra, Betanana; -23.4144, 46.459; alt. 1360 m; 8 Feb 2009; B. L. Fisher et al. leg.; montane rainforest, ex rotten log; BLF21443 (CASC). • 1w., 1s.; Réserve Spéciale Kalambatritra, Betanana; -23.4144, 46.459; alt. 1360 m; 8 Feb 2009; B. L. Fisher et al. leg.; montane rainforest, ex rotten log; BLF21444 (CASC). • 1w., 1s.; Réserve Spéciale Kalambatritra, Betanana; -23.4144, 46.459; alt. 1360 m; 8 Feb 2009; B. L. Fisher et al. leg.; montane rainforest, under moss on rotten log; BLF21449 (CASC). • 1w.; Réserve Spéciale Kalambatritra, Betanana; -23.4144, 46.459; alt. 1360 m; 8 Feb 2009; B. L. Fisher et al. leg.; montane rainforest, ex rotten log; BLF21453 (CASC). • 1w., 1s.; Réserve Spéciale Kalambatritra, Betanana; -23.4144, 46.459; alt. 1360 m; 8 Feb 2009; B. L. Fisher et al. leg.; montane rainforest, ex rotten log; BLF21481 (CASC).

##### Diagnosis.

Moderately large species. ***Major workers.*** Head in full-face view sub-oval and slightly widening posteriorly, with anterior and posterior sides slightly convex, in lateral view sub-oval; ventral and dorsal faces convex; sides of the head with very dense, long, suberect to erect pilosity; medial part of frons with thick, interrupted, dense, and longitudinally irregular rugae with indistinctly rugulate interspaces; occipital lobes and area posterolateral from eyes without smooth notches; scape, when laid back, exceeding the midlength of head by one-fifth of its length; inner hypostomal teeth distinct, moderately large to large and wide, closely spaced, triangular with apex directed slightly inward; outer hypostomal teeth lobe-like, narrower than and approximately as high as inner teeth, apex directed upward; inner and outer hypostomal teeth closely spaced and not connected by concavity; mesosoma rugofoveolate; katepisternum with reduced sculpture and sometimes with smooth notch; gaster smooth; body brown to dark brown. ***Minor workers.*** Head foveolate with additional sparse and longitudinal rugae at least on lateral sides of frons, sometimes longitudinal to irregular rugae occur also on medial frons, vertex sometimes with transverse rugulae; area posterolateral from eyes sometimes with weaker foveolae or smooth, sometimes with additional rugae; scape, when laid back, exceeding the posterior head margin by one-third of its length; promesonotum low and moderately long; promesonotal groove present; propodeal spines minute and triangular; mesosoma foveolate; katepisternum with sparser foveolae to partially smooth; body yellow brown to dark brown.

##### Description.

**Major workers.** Measurements (*N* = 10): HL: 1.17–1.52 (1.39); HW: 1.2–1.57 (1.42); SL: 0.83–0.91 (0.87); EL: 0.15–0.19 (0.18); WL: 1.15–1.29 (1.21); PSL: 0.16–0.21 (0.2); MTL: 0.8–0.89 (0.85); PNW: 0.48–0.62 (0.54); PTW: 0.16–0.21 (0.17); PPW: 0.31–0.44 (0.37); CI: 95.4–100.1 (97.9); SI: 56.6–72.5 (61.6); PSLI: 11.8–15.5 (14.1); PPI: 41.7–56.0 (47.7); PNI: 36.3–40.5 (38.1); MTI: 56.0–67.0 (60.1).

***Head.*** In full-face view sub-oval, widening posteriorly, with anterior and posterior sides convex (Fig. 60B). In lateral view sub-oval; ventral and dorsal faces convex; inner hypostomal teeth visible. Sides of the head with dense, long, suberect to erect pilosity; whole head with dense, long, decumbent to erect pilosity. Medial part of frons with thick, longitudinally irregular, interrupted, and dense rugae, interspaces indistinctly rugulate, rugae directed outward on posteromedial part; lateral sides with thick, dense and irregular rugae with distinctly rugulate interspaces. Occipital lobes with weaker and sparser rugae and predominantly smooth interspaces. Area posterolateral from eyes with longitudinal, moderately thick, dense rugae with rugulate interspaces, sculpture weakening posteriorly. Gena with relatively sparse, thick, and longitudinal rugae and smooth to indistinctly rugulate interspaces. Centre of clypeus smooth and shiny, lateral sides with indistinct rugulae; median notch present, moderately wide, and shallow; median longitudinal carina present; lateral longitudinal carinae absent. Scape, when laid back, exceeding the midlength of head by two-fifths of its length; pilosity subdecumbent to erect (Fig. 60B, D). Inner hypostomal teeth distinct, moderately large to large, closely spaced, triangular, with rounded apex directed inward; outer hypostomal teeth lobe-like, slightly higher than or as high as inner teeth; inner and outer hypostomal teeth closely spaced and not connected by concavity (Fig. 64R). ***Mesosoma.*** In lateral view, promesonotum short, angular, and moderately low, posterior mesonotum steep, mesonotal process indistinct, tubercle-like; promesonotal groove absent; metanotal groove absent; propodeal spines moderately long, with narrow base and acute apex; humeral area produced (Fig. 60D). Surface shiny and distinctly rugofoveolate; promesonotum with additional sparse and moderately thick, transverse rugae on dorsum; katepisternum with reduced sculpture or partially smooth. Pilosity moderately dense, long, and erect (Fig. 60D, F). ***Petiole.*** Shiny with dense foveolae; node finely foveolate, triangular, with rounded and thick apex, in rear view node dorsoventrally slightly convex; pilosity moderately sparse and erect (Fig. 60D, F). ***Postpetiole.*** Shiny and foveolate; dorsum with reduced sculpture and smooth notch; in dorsal view oval, lateral margins medially with two dentate projections; pilosity long, moderately sparse and erect (Fig. 60D, F). ***Gaster.*** Shiny and smooth; pilosity moderately dense, long, and erect (Fig. 60D, F). ***Colour.*** Ferruginous to dark brown, with yellowish legs (Fig. 60D, F).

**Minor workers.** Measurements (*N* = 10): HL: 0.61–0.72 (0.69); HW: 0.55–0.59 (0.57); SL: 0.78–0.85 (0.82); EL: 0.12–0.14 (0.13); WL: 0.86–0.95 (0.91); PSL: 0.07–0.1 (0.09); MTL: 0.66–0.7 (0.69); PNW: 0.38–0.43 (0.4); PTW: 0.09–0.11 (0.1); PPW: 0.17–0.2 (0.18); CI: 110.2–125.8 (120.2); SI: 136.9–149.8 (143.4); PSLI: 9.8–15.4 (12.8); PPI: 48.6–61.5 (54.5); PNI: 65.8–74.6 (69.6); MTI: 117.3–123.9 (120.4).

***Head.*** Cephalic margin slightly convex (Fig. 60A). Pilosity relatively sparse, moderately long, subdecumbent to erect. Sculpture shiny and foveolate with additional sparse and longitudinal rugae at least on lateral sides of frons, sometimes longitudinal to irregular rugae occur also on medial frons, vertex sometimes with transverse rugulae; area posterolateral from eyes sometimes with weaker foveolae or smooth, sometimes with additional rugae; antennal sockets with few thick, curved outward rugae and foveolate interspaces. Clypeus with median longitudinal carina absent; two lateral longitudinal carinae absent. Scape, when laid back, exceeding the posterior head margin by one-third of its length; pilosity dense, suberect to erect (Fig. 60A, C). ***Mesosoma.*** In lateral view, promesonotum low and moderately long, arched; promesonotal groove indistinct; metanotal groove distinct; propodeal spines minute and triangular (Fig. 60C). Sculpture shiny and foveolate; katepisternum with small smooth notch. Pilosity very sparse, moderately long, and erect (Fig. 60C, E). ***Gaster.*** With sparse, erect pilosity (Fig. 60C, E). ***Colour.*** Brown, legs yellowish (Fig. 60C, E).

##### Biology.

The species was collected between 500–1700 m in elevation, in rainforest, montane rainforest and montane forest. Nests were located in rotten logs, under stones and under moss.

##### Comments.

*Pheidole veteratrix* express high variability in body colouration and head sculpture in minor workers. Body colouration varies from brown to dark brown with intermediate forms but the type of body colouration is stable within colony. Head sculpture of minor workers is predominantly foveolate, most often with vertex and area posterolateral from eyes partially or entirely smooth, additionally foveolae can be covered by a network of rugae that are longitudinal on frons to transverse of vertex, rugae can be thin to thick, and most of the time interrupted. Dark brown populations can be confused with parapatric *P. mainty* sp. nov. but its major workers can be easily separated based on lack of shagreened first gastral tergite and body never brownish black; minor workers differ in sparser foveolae on mesosoma and at least partially smooth katepisternum. Populations of *P. veteratrix* with brighter body colouration are similar to *P. anomala* sp. nov., a species known from Parc National Montagne d’Ambre in Antsiranana. Major workers of *P. veteratrix* can be separated based on brown to dark brown body and frons with smooth to indistinctly rugulate interspaces; minor workers based on body colouration which is never yellow and presence of smooth notches on katepisternum. Minors of *P. veteratrix* also can be confused with minor workers of *P. joffreville* sp. nov., known from the northernmost part of the Antsiranana prefecture, but they differ in low and long promesonotum, absence of smooth notch on anteromedial frons, and minute propodeal spines.

*Pheidole veteratrix angustinoda* was distinguished from *P. veteratrix* based on smaller body size and shape of postpetiole, which according to the description is as wide as long. After study of the type specimens of both taxa and additional investigation of new material, we concluded that those two differences mentioned by Forel (1892) overlap with the intraspecific variability of *P. veteratrix*. Additionally, we were unable to find any additional characters allowing us to separate those two taxa. Thus, we consider *Pheidole veteratrix angustinoda* a junior synonym of *Pheidole veteratrix*.

#### Pheidole volontany sp. nov.

Taxon classificationAnimalia

6AC79A16-7563-596F-81F9-32D26E56B9E7

http://zoobank.org/007D00C1-ED9C-462D-B6F2-F566494EE806

**Figure 61.**
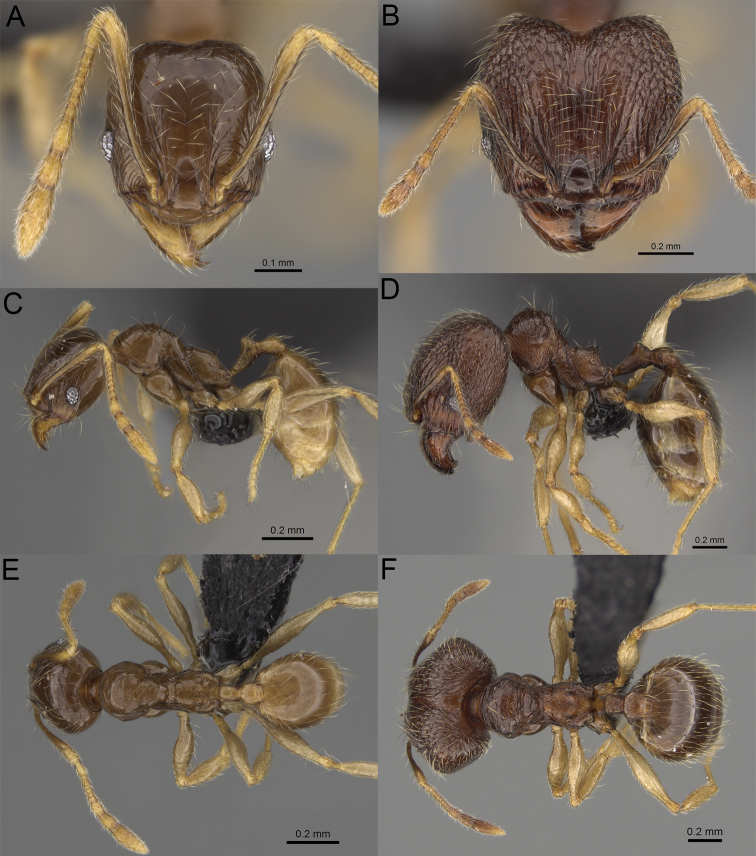
*Pheidole volontany* Forel, full-face view (**A**), profile (**C**), and dorsal view (**E**) of minor paratype worker (CASENT0491862) and full-face view (**B**), profile (**D**), and dorsal view (**F**) of holotype major worker (CASENT0491861).

Figs 61A–F , 64S , 67K

##### Type material.

***Holotype.*** Madagascar. • 1 major worker; Toliara; Forêt Classée d’Analavelona, 33.2 km 344°NNW Mahaboboka; -22.64333, 44.17167; alt. 1300 m; 22 Feb 2003; Fisher et al. leg.; montane rainforest, ex rotten log; BLF07935; CASENT0491861, bottom specimen on the pin (CASC). ***Paratypes.*** • 9w., 3s.; same data as for holotype; CASENT0491860, CASENT0491863, CASENT0491862, CASENT0491864, CASENT0872247 (CASC, MHNG, PBZT).

##### Other material.

Madagascar. –**Toliara**: • 3w., 2s.; Forêt Classée d’Analavelona, 33.2 km 344°NNW Mahaboboka; -22.64333, 44.17167; alt. 1300 m; 22 Feb 2003; Fisher et al. leg.; montane rainforest, ex rotten log; BLF07925 (CASC). • 3w.; Forêt Classée d’Analavelona, 33.2 km 344°NNW Mahaboboka; -22.64333, 44.17167; alt. 1300 m; 22 Feb 2003; Fisher et al. leg.; montane rainforest, ex rotten log; BLF07926 (CASC). • 3w., 2s.; Forêt Classée d’Analavelona, 33.2 km 344°NNW Mahaboboka; -22.64333, 44.17167; alt. 1300 m; 22 Feb 2003; Fisher et al. leg.; montane rainforest, ex rotten log; BLF07927 (CASC). • 1w.; Forêt Classée d’Analavelona, 33.2 km 344°NNW Mahaboboka; -22.64333, 44.17167; alt. 1300 m; 22 Feb 2003; Fisher et al. leg.; montane rainforest, ex rotten log; BLF07928 (CASC). • 6w., 3s.; Forêt Classée d’Analavelona, 33.2 km 344°NNW Mahaboboka; -22.64333, 44.17167; alt. 1300 m; 22 Feb 2003; Fisher et al. leg.; montane rainforest, ex rotten log; BLF07931 (CASC). • 6w.; Forêt Classée d’Analavelona, 33.2 km 344°NNW Mahaboboka; -22.64333, 44.17167; alt. 1300 m; 22 Feb 2003; Fisher et al. leg.; montane rainforest, ex rotten log; BLF07939 (CASC). • 3w., 1s.; Forêt Classée d’Analavelona, 33.2 km 344°NNW Mahaboboka; -22.64333, 44.17167; alt. 1300 m; 22 Feb 2003; Fisher et al. leg.; montane rainforest, ex rotten log; BLF07952 (CASC).

##### Diagnosis.

Minute species. ***Major workers.***HL < 1.05 mm and WL < 0.9 mm; head in full-face view sub-oval, slightly widening posteriorly, with anterior and posterior sides convex, in lateral view sub-oval; ventral and dorsal faces convex; body dark brown; sides of head with dense, very long, suberect to erect pilosity; entire head distinctly sculptured, medial part of frons with moderately dense, thick, longitudinal, and interrupted rugae, interspaces shiny and smooth; scape, when laid back, exceeding the midlength of head by one-fifth of its length; promesonotal dorsum with rugofoveolae and additional thick transverse to irregular rugae; lateral sides of pronotum, anepisternum, katepisternum, and propodeum with dense and moderately thick to thin rugoreticulae; inner hypostomal teeth distinct, large, closely spaced, triangular, with rounded apex directed slightly inward; outer hypostomal teeth lobe-like, wider than inner hypostomal teeth, and approximately the same height, apex directed outward; inner and outer hypostomal teeth closely spaced and connected by concavity; base of first gastral tergite smooth. ***Minor workers.***HL < 0.5 mm and WL < 0.6 mm, scape, when laid back, surpassing the posterior head margin by one-fifth of its length; propodeal spines minute, triangular; head relatively oval, smooth, and shiny with a few short, longitudinal rugae on the anterolateral sides of head; body brown; mesosoma smooth, indistinct foveolae can occur on propodeal dorsum and lateral sides of pronotum.

##### Description.

**Major workers.** Measurements (*N* = 10): HL: 0.91–1.05 (0.98); HW: 0.97–1.06 (1.02); SL: 0.55–0.6 (0.57); EL: 0.12–0.15 (0.14); WL: 0.82–0.87 (0.85); PSL: 0.13–0.18 (0.15); MTL: 0.51–0.56 (0.53); PNW: 0.43–0.5 (0.46); PTW: 0.14–0.16 (0.15); PPW: 0.26–0.34 (0.3); CI: 93.0–101.0 (96.2); SI: 51.4–61.6 (56.3); PSLI: 13.3–17.7 (14.9); PPI: 44.0–58.0 (50.2); PNI: 44.0–58.0 (50.2); MTI: 49.2–53.6 (51.9).

***Head.*** In full-face view sub-oval, slightly widening posteriorly, with anterior and posterior sides convex (Fig. 61B). In lateral view sub-oval; ventral and dorsal faces convex; inner hypostomal teeth visible. Sides of the head with dense, very long, suberect to erect pilosity; whole head with dense, long, decumbent to erect pilosity. Medial part of frons with moderately dense, thick, longitudinal and interrupted rugae, interspaces shiny and smooth; anterolateral sides with longitudinal, thick, and dense rugae; interspaces shiny with relatively dense and distinct rugofoveolae; posterolateral sides with thick, dense, and irregular rugae with relatively dense and distinct rugofoveolae. Occipital lobes with sparse, thick, and irregular rugae; interspaces rugofoveolate. Gena with relatively dense, thick, longitudinal rugae and rugofoveolate interspaces. Area posterolateral from eyes shiny, with dense, thick rugofoveolae and smooth interspaces. Centre of clypeus smooth and shiny, lateral sides with indistinct rugulae; median notch present, moderately wide, and shallow; median longitudinal carina present; lateral longitudinal carinae absent. Scape, when laid back, exceeding the midlength of head by one-fifth of its length; pilosity subdecumbent to erect (Fig. 61B, D). Inner hypostomal teeth distinct, large, closely spaced, triangular, with rounded apex directed slightly inward; outer hypostomal teeth lobe-like, wider than inner hypostomal teeth and approximately the same height, apex directed outward; inner and outer hypostomal teeth closely spaced and connected by concavity (Fig. 64S). ***Mesosoma.*** In lateral view, promesonotum short, angular, and moderately low, posterior mesonotum moderately steep, mesonotal process indistinct, tubercle-like or absent; promesonotal groove absent; metanotal groove absent; propodeal spines moderately short, with wide base and acute apex; humeral area laterally weakly produced (Fig. 61D). Surface shiny; promesonotal dorsum with rugofoveolae and additional thick transverse to irregular rugae; lateral sides of pronotum, anepisternum, katepisternum, and propodeum with dense and moderately thick to thin rugoreticulae. Pilosity sparse, long, and erect (Fig. 61D, F). ***Petiole.*** Shiny with fine foveolae; node smooth to finely foveolate, low, triangular, with rounded and thin apex, in rear view node dorsoventrally straight to slightly convex; pilosity moderately sparse and erect (Fig. 61D, F). ***Postpetiole.*** Shiny and foveolate; dorsum with reduced sculpture and smooth notch; in dorsal view oval, lateral margins medially with two dentate projections; pilosity long, moderately sparse, and erect (Fig. 61D, F). ***Gaster.*** Shiny and smooth; pilosity dense, long, and erect (Fig. 61D, F). ***Colour.*** Dark brown; legs and antennae yellow (Fig. 61D, F).

**Minor workers.** Measurements (*N* = 10): HL: 0.48–0.51 (0.5); HW: 0.44–0.46 (0.45); SL: 0.47–0.51 (0.49); EL: 0.09–0.11 (0.1); WL: 0.57–0.62 (0.59); PSL: 0.06–0.08 (0.07); MTL: 0.37–0.39 (0.38); PNW: 0.27–0.31 (0.29); PTW: 0.06–0.08 (0.07); PPW: 0.11–0.13 (0.12); CI: 108.8–116.0 (112.0); SI: 105.4–111.4 (108.4); PSLI: 11.2–14.6 (13.0); PPI: 56.9–70.2 (62.7); PNI: 61.4–68.6 (65.3); MTI: 81.3–88.2 (84.3).

***Head.*** Cephalic margin indistinctly concave or straight (Fig. 61A). Pilosity relatively sparse, long, decumbent to suberect. Sculpture smooth and shiny with a few short, longitudinal rugae on the anterolateral sides of head; antennal sockets with few thick, curved outward rugae and smooth interspaces. Clypeus with median longitudinal carina absent; two lateral longitudinal carinae absent. Scape, when laid back, surpassing the posterior head margin by one-fifth of its length; pilosity dense, subdecumbent to erect (Fig. 61A, C). ***Mesosoma.*** In lateral view, promesonotum moderately high and short, arched; promesonotal groove absent; metanotal groove distinct; propodeal spines minute, triangular (Fig. 61C). Sculpture shiny and smooth; indistinct foveolae can occur on propodeal dorsum and lateral sides of pronotum. Pilosity very sparse, moderately long, and erect (Fig. 61C, E). ***Postpetiole.*** Short, low, and relatively flat; with few short, erect setae (Fig. 61C, E). ***Gaster.*** With sparse, erect pilosity (Fig. 61C, E). ***Colour.*** Brown, legs and antenna yellow (Fig. 61C, E).

##### Etymology.

Malagasy for brown in reference to body colouration.

##### Biology.

The species was collected at 1300 m in elevation, in montane rainforest. Nests were located in rotten logs.

##### Comments.

*Pheidole volontany* sp. nov. belongs to the group of species characterised by small body size (major workers: HL < 1.05 mm, WL < 0.9 mm and minor workers HL < 0.5 mm, WL < 0.6 mm), sub-oval, slightly widening posteriorly head with anterior and posterior sides convex in major workers, and minor workers with yellow to brown body colouration and foveolate head or head predominantly smooth and relatively oval. The group includes six species: *P. havoana* sp. nov., *P. kely* sp. nov., *P. parvula* sp. nov., *P. parvulogibba* sp. nov., *P. volontany* sp. nov., and *P. midongy* sp. nov. Because of dark body colouration *P. volontany* sp. nov., known from Forêt Classée d’Analavelona in Toliara, is most similar to *P. midongy* sp. nov., described form Parc National Befotaka-Midongy in Fianarantsoa. Major workers of *P. volontany* sp. nov. differ from *P. midongy* sp. nov. in frons with moderately dense, thick, longitudinal, and interrupted rugae with shiny and smooth interspaces, and sides of the head with dense, very long, suberect to erect pilosity; minor workers differ in predominantly smooth head and darker brown body colouration.

#### Pheidole vony sp. nov.

Taxon classificationAnimalia

6F632810-1476-5DFA-804B-D19A2FD0C655

http://zoobank.org/D61A8A41-8E37-4B94-A723-9EABB9A024D6

**Figure 62.**
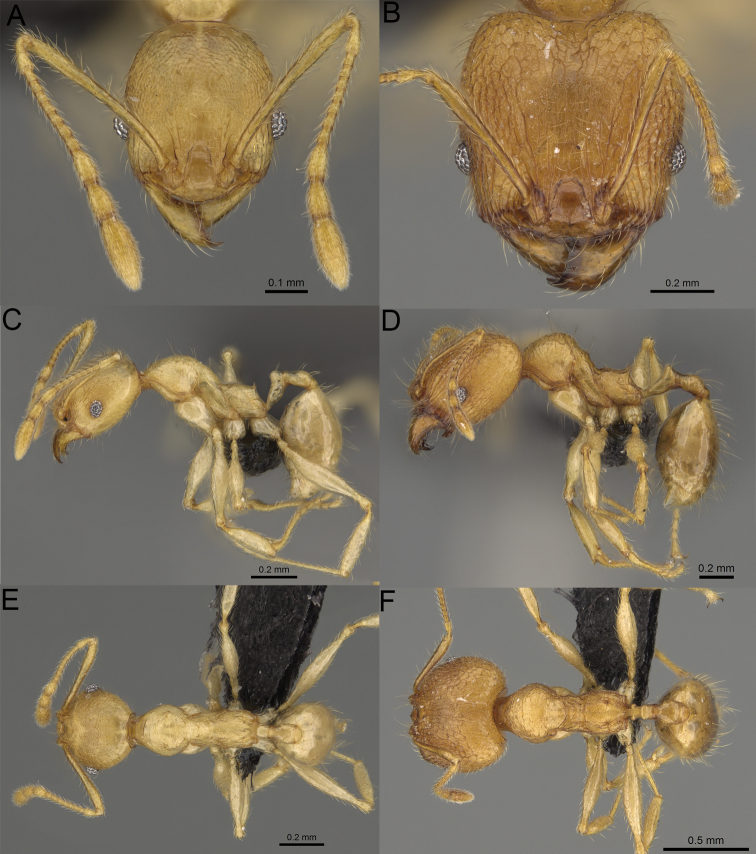
*Pheidole vony* Forel, full-face view (**A**), profile (**C**), and dorsal view (**E**) of minor paratype worker (CASENT0498053) and full-face view (**B**), profile (**D**), and dorsal view (**F**) of holotype major worker (CASENT0498055).

Figs 62A–F , 64T , 67L

##### Type material.

***Holotype.*** Madagascar. • 1 major worker; Toamasina; Montagne d’Anjanaharibe, 19.5 km 27°NNE Ambinanitelo; -15.17833, 49.635; alt. 1100 m; 12 Mar 2003; Fisher et al. leg.; montane rainforest, ex rotten tree stump; BLF08197; CASENT0498055, bottom specimen on the pin (CASC). ***Paratypes.*** • 3w., 5s.; same data as for holotype; CASENT0498053, CASENT0872250CASENT0498054 (CASC, MHNG, PBZT).

##### Other material.

Madagascar. –**Toamasina**: • 3w.; Montagne d’Akirindro 7.6 km 341°NNW Ambinanitelo; -15.28833, 49.54833; alt. 600 m; 17 Mar 2003; Fisher et al. leg.; rainforest, ex rotten stick on ground; BLF08317 (CASC). • 1s.; Montagne d’Akirindro 7.6 km 341°NNW Ambinanitelo; -15.28833, 49.54833; alt. 600 m; 17 Mar 2003; Fisher et al. leg.; rainforest, ex rotten log; BLF08377 (CASC). • 3w.; Montagne d’Anjanaharibe, 19.5 km 27°NNE Ambinanitelo; -15.17833, 49.635; alt. 1100 m; 12 Mar 2003; Fisher et al. leg.; montane rainforest, ex rotten log; BLF08172 (CASC). • 3s.; Montagne d’Anjanaharibe, 19.5 km 27°NNE Ambinanitelo; -15.17833, 49.635; alt. 1100 m; 12 Mar 2003; Fisher et al. leg.; montane rainforest, ex root mat, ground layer; BLF08186 (CASC).

##### Diagnosis.

Moderately large species. ***Major workers.*** Head in full-face view sub-oval, not widening posteriorly, with anterior and posterior sides slightly convex, in lateral view sub-oval, ventral and dorsal faces convex; sides of the head with moderately dense, moderately long, suberect to erect pilosity; medial frons with moderately dense, thin rugulae, anteriorly longitudinal and interrupted, posteromedial part with irregular rugae, interspaces shiny with sparse and indistinct irregular rugulae; lateral sides with longitudinal anteriorly to irregular posteriorly, thick, and relatively dense rugae with distinctly rugofoveolate interspaces; occipital lobes and area posterolateral from eyes never smooth; scape, when laid back, exceeding the midlength of head by three-fifths of its length; inner hypostomal teeth distinct, large, closely spaced, triangular, with rounded apex directed outward; outer hypostomal teeth lobe-like, lower and more narrow than inner hypostomal teeth, apex directed outward; inner and outer hypostomal teeth closely spaced and not connected by concavity; pronotum and mesonotum predominantly smooth with transverse rugae on dorsum and indistinct rugofoveolae on lateral sides; katepisternum and anepisternum predominantly smooth; propodeum rugofoveolate; body yellow. ***Minor workers.*** Head foveolate; area posterolateral from eyes smooth; scape, when laid back, surpassing the posterior head margin by two-fifths of its length; promesonotum moderately low and moderately short; promesonotal groove absent; propodeal spines very small and triangular; mesosoma smooth, sometimes with sparse and indistinct foveolae on lateral sides; body yellow.

##### Description.

**Major workers.** Measurements (*N* = 9): HL: 0.89–1.06 (0.95); HW: 0.83–1.02 (0.92); SL: 0.6–0.69 (0.65); EL: 0.12–0.14 (0.13); WL: 0.94–1.02 (0.98); PSL: 0.15–0.19 (0.16); MTL: 0.57–0.64 (0.6); PNW: 0.38–0.47 (0.42); PTW: 0.12–0.16 (0.13); PPW: 0.19–0.3 (0.24); CI: 100.5–106.4 (104.0); SI: 64.2–76.6 (71.5); PSLI: 15.5–20.0 (17.2); PPI: 47.3–61.5 (55.7); PNI: 44.7–48.5 (46.1); MTI: 59.4–71.7 (66.1).

***Head.*** In full-face view sub-oval, not widening posteriorly, with anterior and posterior sides slightly convex (Fig. 62B). In lateral view sub-oval; ventral and dorsal faces convex; inner hypostomal teeth visible. Sides of the head with moderately dense, moderately long, suberect to erect pilosity; whole head with dense, long, decumbent to erect pilosity. Medial part of frons with moderately dense, thin rugulae, anteriorly longitudinal and interrupted, posteromedial part with irregular rugae, interspaces shiny with sparse, indistinct, and irregular rugulae; lateral sides with longitudinal anteriorly to irregular posteriorly, thick, and relatively dense rugae with distinctly rugofoveolate interspaces. Occipital lobes with sparse, thick and irregular rugae; interspaces with fine rugofoveolae. Gena with relatively dense, thick, and longitudinal rugae and smooth to indistinctly rugofoveolae interspaces. Area posterolateral from eyes with thin and fine rugofoveolae, posteriormost parts with reduced sculpture and smooth notch. Centre of clypeus smooth and shiny, lateral sides with indistinct rugulae; median notch present, moderately wide, and shallow; median longitudinal carina present; lateral longitudinal carinae absent. Scape, when laid back, exceeding the midlength of head by three-fifths of its length; pilosity subdecumbent to erect (Fig. 62B, D). Inner hypostomal teeth distinct, large, closely spaced, triangular, with rounded apex directed outward; outer hypostomal teeth lobe-like, lower and more narrow than inner hypostomal teeth, apex directed outward; inner and outer hypostomal teeth closely spaced not connected by concavity (Fig. 64T). ***Mesosoma.*** In lateral view, promesonotum short, angular, and moderately low, posterior mesonotum moderately steep, mesonotal process indistinct, tubercle-like or absent; promesonotal groove absent; metanotal groove indistinct; propodeal spines moderately long, moderately wide, with acute apex; humeral area laterally weakly produced (Fig. 62D). Surface shiny; pronotum and mesonotum predominantly smooth with transverse rugae on dorsum and indistinct rugofoveolae on lateral sides; katepisternum and anepisternum predominantly smooth; propodeum rugofoveolate. Pilosity relatively dense, long, and erect (Fig. 62D, F). ***Petiole.*** Shiny with fine and dense rugofoveolae; node smooth, low, triangular, with rounded and thin apex, in rear view node dorsoventrally slightly convex; pilosity moderately sparse and erect (Fig. 62D, F). ***Postpetiole.*** Shiny and smooth; in dorsal view oval, lateral margins medially with two dentate projections; pilosity long, moderately sparse and erect (Fig. 62D, F). ***Gaster.*** Shiny and smooth; pilosity moderately dense, long, and erect (Fig. 62D, F). ***Colour.*** Yellow; mandibles and gaster slightly darker (Fig. 62D, F).

**Minor workers.** Measurements (*N* = 6): HL: 0.54–0.58 (0.56); HW: 0.45–0.48 (0.47); SL: 0.58–0.61 (0.6); EL: 0.1–0.11 (0.11); WL: 0.69–0.73 (0.71); PSL: 0.07–0.09 (0.07); MTL: 0.48–0.51 (0.49); PNW: 0.31–0.33 (0.32); PTW: 0.07–0.09 (0.08); PPW: 0.12–0.13 (0.13); CI: 117.4–121.1 (118.7); SI: 126.5–131.0 (128.4); PSLI: 12.0–15.0 (13.7); PPI: 58.7–71.1 (63.4); PNI: 65.7–69.0 (67.2); MTI: 101.7–109.2 (104.9).

***Head.*** Cephalic margin indistinctly convex or straight (Fig. 62A). Pilosity relatively sparse, long, decumbent to suberect. Sculpture foveolate; area posterolateral from eyes smooth. Clypeus with median longitudinal carina absent; two lateral longitudinal carinae absent. Scape, when laid back, surpassing the posterior head margin by two-fifths of its length; pilosity dense, subdecumbent to erect (Fig. 62A, C). ***Mesosoma.*** In lateral view, promesonotum moderately low and moderately short, arched; promesonotal groove absent; metanotal groove indistinct; propodeal spines very small and triangular (Fig. 62C). Sculpture smooth, sometimes with sparse and indistinct foveolae on lateral sides. Pilosity very sparse, moderately long, and erect (Fig. 62C, E). ***Postpetiole.*** Short, low, and relatively flat; with few short, erect setae (Fig. 62C, E). ***Gaster.*** With sparse, erect pilosity (Fig. 62C, E). ***Colour.*** Yellow, vertex slightly darker (Fig. 62C, E).

##### Etymology.

Malagasy for yellow in reference to the body colouration.

##### Biology.

The species was collected between 600–1100 m in elevation, in rainforest and montane rainforest. Nests were located in rotten logs, root mats, rotten tree stumps, and rotten sticks on the ground.

##### Comments.

*Pheidole vony* sp. nov. is a member of a group of species characterised by body colouration bright yellow to orange in majors and yellow in minors, head sub-oval, not widening posteriorly with sides not convex or convex indistinctly, and medial part of frons with longitudinal and interrupted rugae on the anterior part and distinctly irregular rugae on the posterior one. The group includes three taxa: *P. vony* sp. nov., *P. befotaka* sp. nov., and *P. mamiratra* sp. nov. *Pheidole vony* sp. nov. is known from two localities in Toamasina: Montagne d’Anjanaharibe and Montagne d’Akirindro, and its distribution doesn’t overlap with two remaining members of the group. Morphologically *P. vony* sp. nov. is most similar to *P. mamiratra* sp. nov., known only from Station Forestière Angavokely in Antananarivo. Major workers of *P. vony* sp. nov. differ from *P. mamiratra* sp. nov. in medial part of frons with moderately dense and thin rugae and inner hypostomal teeth large and triangular, with rounded apex directed outward and outer hypostomal teeth lobe-like, lower, and more narrow than inner hypostomal teeth; minors differ in more distinctly foveolate head sculpture. However, minor and major workers of *P. vony* sp. nov. can be also confused with *P. sparsa* sp. nov., known from Bemanevika in Mahajanga, and *P. hazo* sp. nov., described from the vicinity of Antananarivo. Majors of *P. vony* sp. nov. differ from *P. sparsa* sp. nov. and *P. hazo* sp. nov. in median frons with thinner and irregular rugae and more irregular sculpture on lateral sides of frons; minor workers differ in smaller propodeal spines, lack of promesonotal groove, strongly reduced sculpture of mesosoma, and head lacking additional, indistinct rugulae.

**Figure 63.**
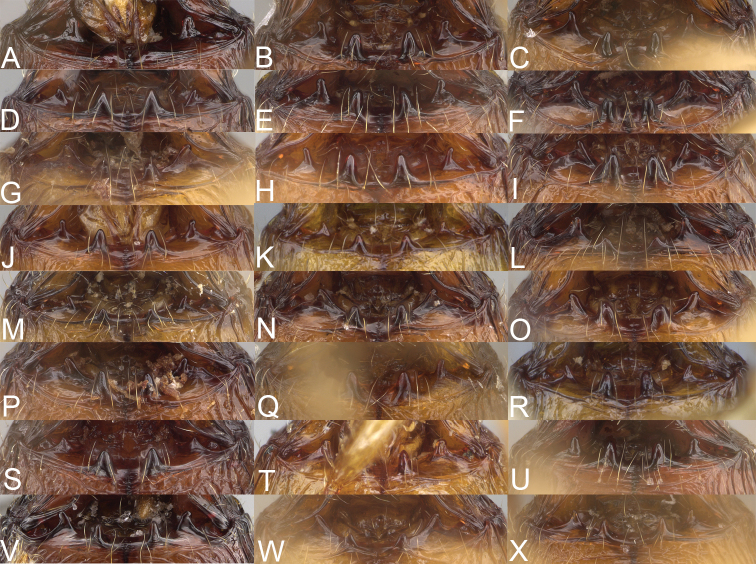
Major worker, hypostomal teeth. Pheidole alina sp. nov. (**A**). *Pheidole ambohimanga* sp. nov. (**B**). *Pheidole analavelona* sp. nov. (**C**). *Pheidole andohahela* sp. nov. (**D**). *Pheidole anomala* sp. nov. (**E**). *Pheidole anosyenne* sp. nov. (**F**). *Pheidole antranohofa* sp. nov. (**G**). *Pheidole beanka* sp. nov. (**H**). *Pheidole befotaka* sp. nov. (**I**). *Pheidole dasos* sp. nov. (**J**). *Pheidole flavominuta* sp. nov. (**K**). *Pheidole gracilis* sp. nov. (**L**). *Pheidole haboka* sp. nov. (**M**). *Pheidole havoana* sp. nov. (**N**). *Pheidole hazo* sp. nov. (**O**). *Pheidole itremo* sp. nov. (**P**). *Pheidole joffreville* sp. nov. (**Q**). *Pheidole kely* sp. nov. (**R**). *Pheidole lavasoa* sp. nov. (**S**). *Pheidole litigiosa* Forel (**T**). *Pheidole mahamavo* sp. nov. (**U**). *Pheidole mainty* sp. nov. (**V**). *Pheidole mamiratra* sp. nov. (**W**). *Pheidole manantenina* sp. nov. (**X**).

**Figure 64.**
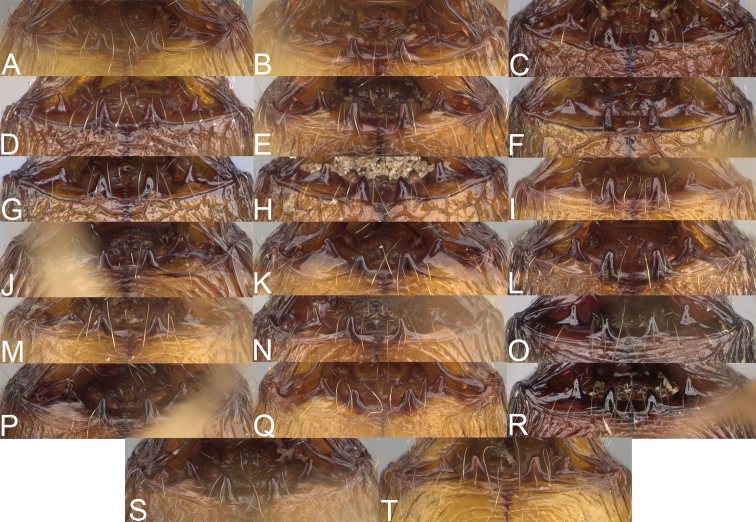
Major worker, hypostomal teeth. *Pheidole masoandro* sp. nov. (**A**). *Pheidole mavohavoana* sp. nov. (**B**). *Pheidole midongy* sp. nov. (**C**). *Pheidole mikros* sp. nov. (**D**). *Pheidole mivory* sp. nov. (**E**). *Pheidole nitidobruna* sp. nov. (**F**). *Pheidole parvula* sp. nov. (**G**). *Pheidole parvulogibba* sp. nov. (**H**). *Pheidole renirano* sp. nov. (**I**). *Pheidole sava* sp. nov. (**J**). *Pheidole sikorae* Forel (**K**). *Pheidole sofia* sp. nov. (**L**). *Pheidole sparsa* sp. nov. (**M**). *Pheidole*﻿ ﻿*tampony*﻿ sp. nov. (**N**). *Pheidole*﻿ ﻿*trichotos*﻿ sp. nov. (**O**). *Pheidole*﻿ ﻿*tsaravoniana*﻿ sp. nov. (**P**). *Pheidole*﻿ ﻿*vadum*﻿ sp. nov. (**Q**). *Pheidole*﻿ ﻿*veteratrix*﻿ Forel (**R**). *Pheidole*﻿ ﻿*volontany*﻿ sp. nov. (**S**). *Pheidole*﻿ ﻿*vony*﻿ sp. nov. (**T**).

**Figure 65.**
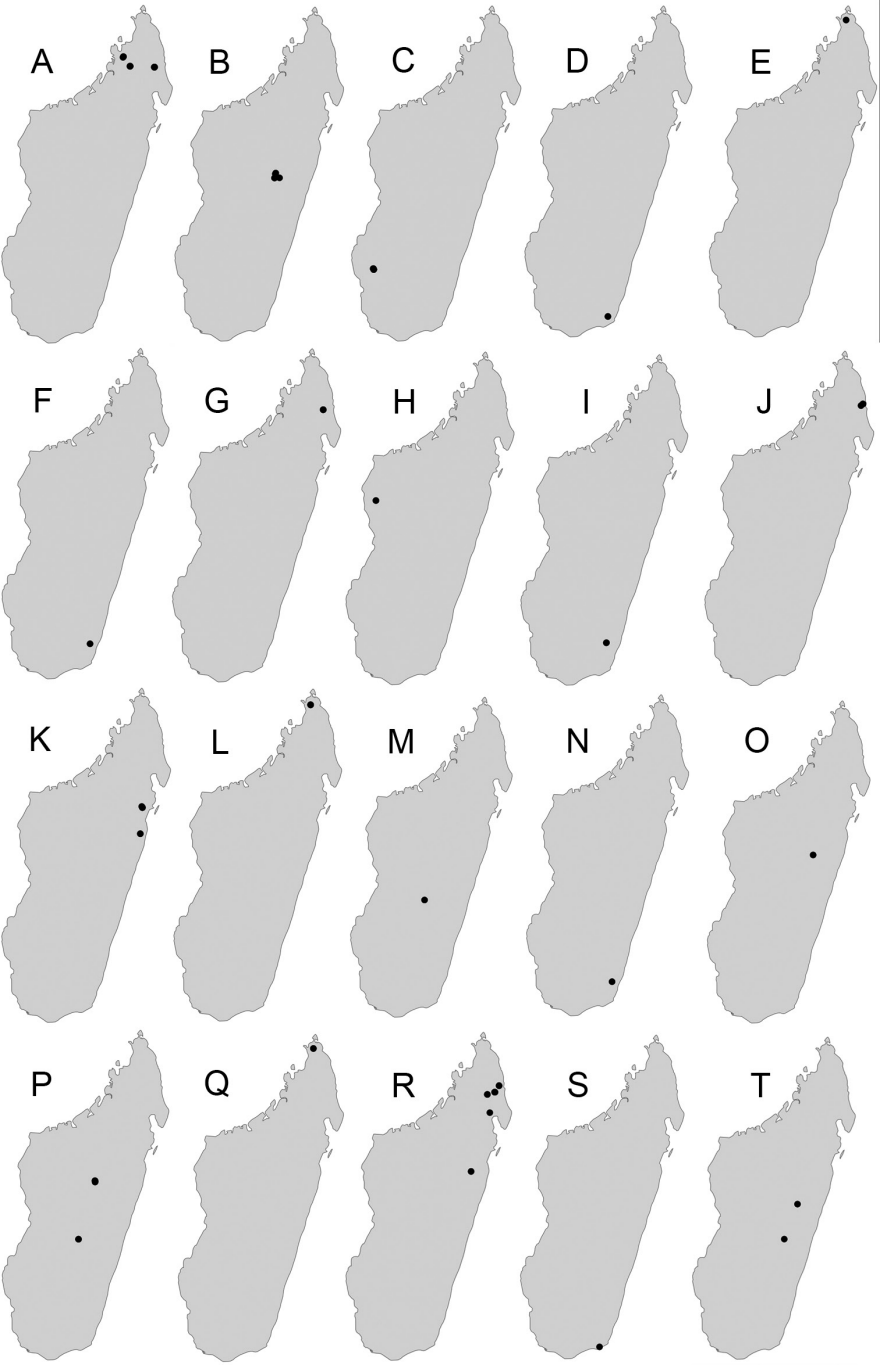
Distribution. *Pheidole alina* sp. nov. (**A**). *Pheidole ambohimanga* sp. nov. (**B**). *Pheidole analavelona* sp. nov. (**C**). *Pheidole andohahela* sp. nov. (**D**). *Pheidole anomala* sp. nov. (**E**). *Pheidole anosyenne* sp. nov. (**F**). *Pheidole antranohofa* sp. nov. (**G**). *Pheidole beanka* sp. nov. (**H**). *Pheidole befotaka* sp. nov. (**I**). *Pheidole dasos* sp. nov. (**J**). *Pheidole flavominuta* sp. nov. (**K**). *Pheidole gracilis* sp. nov. (**L**). *Pheidole haboka* sp. nov. (**M**). *Pheidole havoana* sp. nov. (**N**). *Pheidole hazo* sp. nov. (**O**). *Pheidole itremo* sp. nov. (**P**). *Pheidole joffreville* sp. nov. (**Q**). *Pheidole kely* sp. nov. (**R**). *Pheidole lavasoa* sp. nov. (**S**). *Pheidole litigiosa* Forel (**T**).

**Figure 66.**
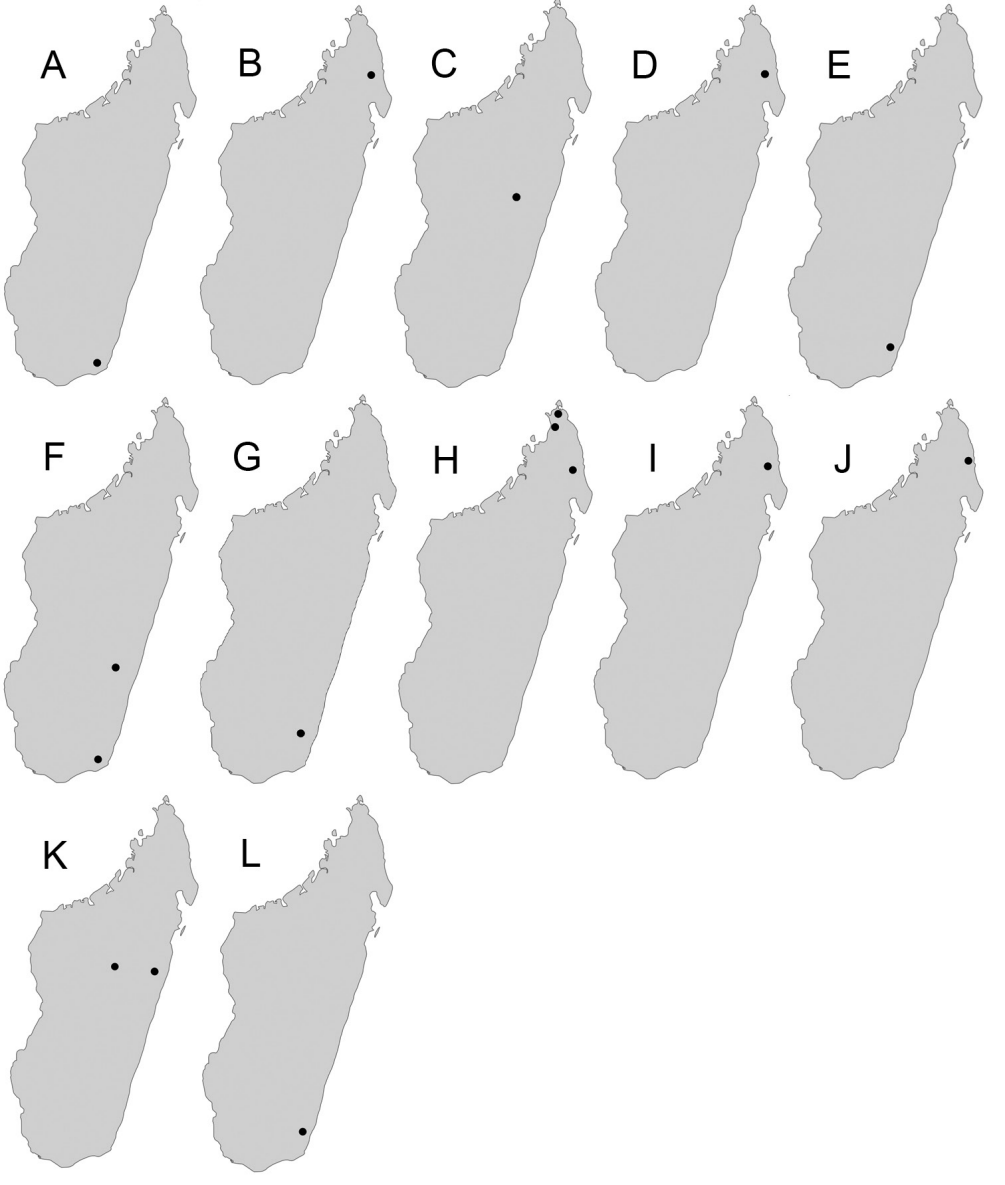
Distribution. *Pheidole*﻿ ﻿*mahamavo*﻿ sp. nov. (**A**). *Pheidole*﻿ *mainty* sp. nov. (**B**). *Pheidole*﻿ ﻿*mamiratra*﻿ sp. nov. (**C**). *Pheidole manantenina* sp. nov. (**D**). *Pheidole*﻿ ﻿*masoandro*﻿ sp. nov. (**E**). *Pheidole mavohavoana* sp. nov. (**F**). *Pheidole*﻿ ﻿*midongy*﻿ sp. nov. (**G**). *Pheidole mikros* sp. nov. (**H**). *Pheidole*﻿ ﻿*mivory*﻿ sp. nov. (**I**). *Pheidole*﻿ ﻿*nitidobruna*﻿ sp. nov. (**J**). *Pheidole*﻿ ﻿*parvula*﻿ sp. nov. (**K**). *Pheidole*﻿ ﻿*parvulogibba*﻿ sp. nov. (**L**).

**Figure 67.**
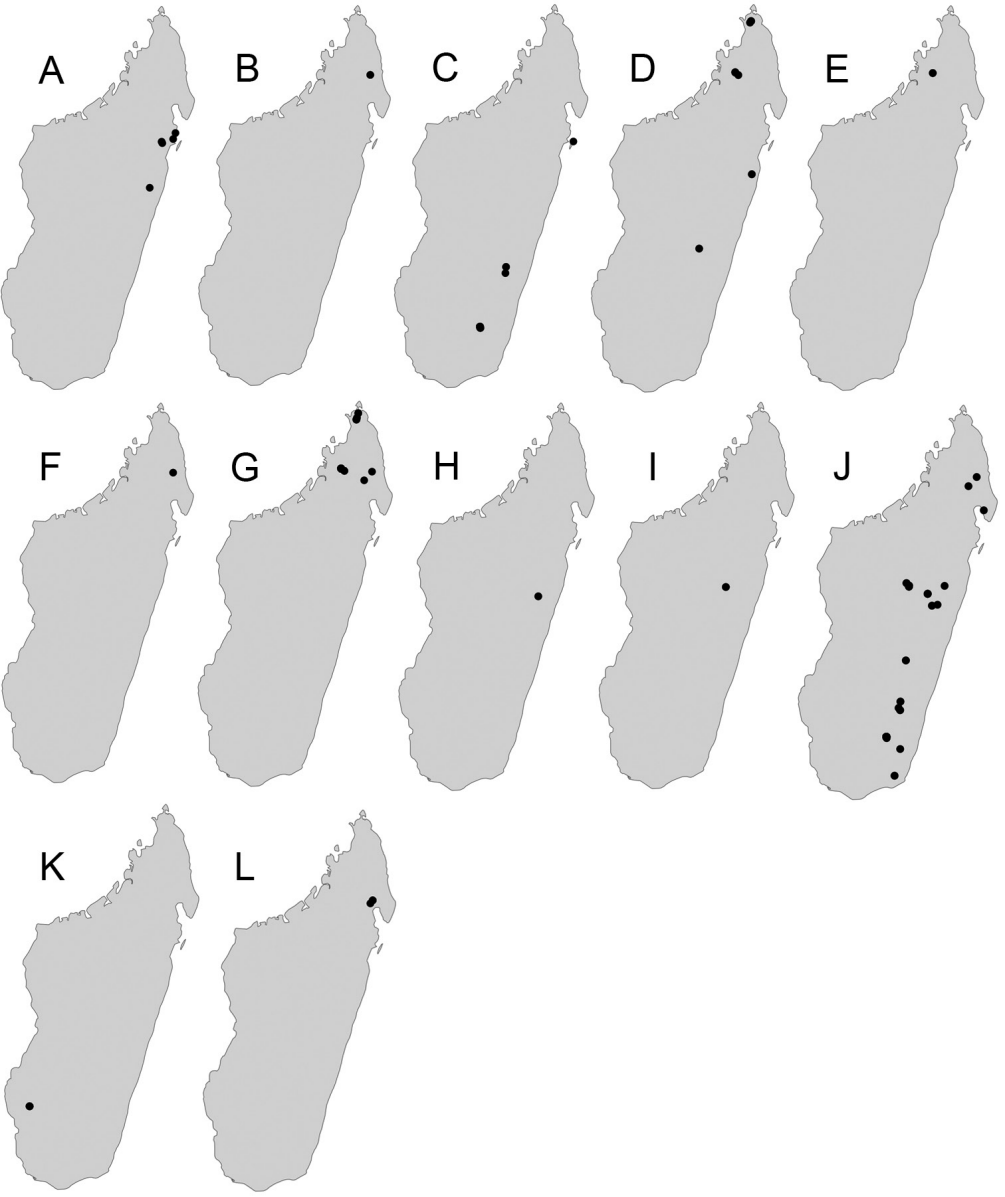
Distribution. *Pheidole renirano* sp. nov. (**A**). *Pheidole sava* sp. nov. (**B**). *Pheidole sikorae* Forel (**C**). *Pheidole sofia* sp. nov. (**D**). *Pheidole sparsa* sp. nov. (**E**). *Pheidole tampony* sp. nov. (**F**). *Pheidole trichotos* sp. nov. (**G**). *Pheidole tsaravoniana* sp. nov. (**H**). *Pheidole vadum* sp. nov. (**I**). *Pheidole veteratrix* Forel (**J**). *Pheidole volontany* sp. nov. (**K**). *Pheidole vony* sp. nov. (**L**).

## Supplementary Material

XML Treatment for Pheidole alina

XML Treatment for Pheidole ambohimanga

XML Treatment for Pheidole analavelona

XML Treatment for Pheidole andohahela

XML Treatment for Pheidole anomala

XML Treatment for Pheidole anosyenne

XML Treatment for Pheidole antranohofa

XML Treatment for Pheidole beanka

XML Treatment for Pheidole befotaka

XML Treatment for Pheidole dasos

XML Treatment for Pheidole flavominuta

XML Treatment for Pheidole gracilis

XML Treatment for Pheidole haboka

XML Treatment for Pheidole havoana

XML Treatment for Pheidole hazo

XML Treatment for Pheidole itremo

XML Treatment for Pheidole joffreville

XML Treatment for Pheidole kely

XML Treatment for Pheidole lavasoa

XML Treatment for Pheidole litigiosa

XML Treatment for Pheidole mahamavo

XML Treatment for Pheidole mainty

XML Treatment for Pheidole mamiratra

XML Treatment for Pheidole manantenina

XML Treatment for Pheidole masoandro

XML Treatment for Pheidole mavohavoana

XML Treatment for Pheidole midongy

XML Treatment for Pheidole mikros

XML Treatment for Pheidole mivory

XML Treatment for Pheidole nitidobruna

XML Treatment for Pheidole parvula

XML Treatment for Pheidole parvulogibba

XML Treatment for Pheidole renirano

XML Treatment for Pheidole sava

XML Treatment for Pheidole sikorae

XML Treatment for Pheidole sofia

XML Treatment for Pheidole sparsa

XML Treatment for Pheidole tampony

XML Treatment for Pheidole trichotos

XML Treatment for Pheidole tsaravoniana

XML Treatment for Pheidole vadum

XML Treatment for Pheidole veteratrix

XML Treatment for Pheidole volontany

XML Treatment for Pheidole vony

## Footnotes

Note. Minor workers of Pheidole kely sp. nov. share the same set of characters as Pheidole havoana sp. nov. and these species can be separated only based on major workers.

Note: Minor workers of P. sikorae, P. antranohofa sp. nov., and P. befotaka sp. nov. (couplet #17) are extremely similar to those of P. litigiosa, P. masoandro sp. nov., P. gracilis sp. nov., and P. tampony sp. nov. (couplet #14) and can be separated only based on major workers. For more details see Comments sections in the species accounts.

Note. At this point, species separation based on minor workers only is very impeded and, in some cases, impossible, therefore we strongly encourage using major workers or relying on nest samples.
